# Sense of coherence and religion/spirituality: A systematic review and meta-analysis based on a methodical classification of instruments measuring religion/spirituality

**DOI:** 10.1371/journal.pone.0289203

**Published:** 2023-08-03

**Authors:** Florian Jeserich, Constantin Klein, Benno Brinkhaus, Michael Teut

**Affiliations:** 1 Institute for Social Medicine, Epidemiology and Health Economics, Charité University Medical Center, Berlin, Germany; 2 Contilia Academy, Contilia GmbH, Essen, Germany; 3 Department of Practical Theology, University of Applied Sciences for Social Work, Education and Nursing, Dresden, Germany; UTAD: Universidade de Tras-os-Montes e Alto Douro, PORTUGAL

## Abstract

The coherence hypothesis assumes that sense of coherence (SOC) explains the positive link between religion/spirituality (R/S) and mental health. The aim of our meta-analysis is to evaluate the evidence for the association between SOC (sensu Antonovsky) and different aspects of R/S and thus to contribute to the verification of the coherence hypothesis. Eighty-nine English- and German-language primarily cross-sectional studies with 67,913 participants met the inclusion criteria. The R/S scales of all included studies were subjected to item-by-item qualitative content analysis in order to determine whether scales do actually measure religion or spirituality and which R/S aspects dominated the instrument. Based on this classification, overall and subgroup meta-analyses were conducted using a random effects model. The adjusted effect size between SOC and all positive R/S measures was *r+* = .120, 95% CI [.092, .149]. Particularly significant (*r+* < -.180 or > .180) were correlations with negative R/S scales (*r+* = -.405, 95% CI [-.476, -.333]), R/S instruments measuring primarily positive emotions (*r+* = .212, 95% CI [.170, .253]) or meaning-making (*r+* = .196, 95% CI [.126, .265]). Both sample characteristics (age, culture, gender, health status, religious affiliation) and study characteristics (e.g., publication year) had a moderating effect on the R/S-SOC connection. The correlation was particularly high in studies from Southern Asia (*r+* = .226, 95% CI [.156, .297]), the African Islamic cultural value zone (*r+* = .196, 95% CI [.106, .285]), and in a small subgroup of Iranian studies (*r+* = .194, 95% CI [.117, .271]). The results confirm that R/S and SOC are clearly associated and suggest that there are different religious/spiritual pathways to a strong SOC. The strength of the associations presumably depends not only on individual differences, but also on cultural embeddedness and social plausibility of R/S. **Trial registration. PROSPERO registration number**: CRD42021240380. https://www.crd.york.ac.uk/prospero/display_record.php?ID = CRD42021240380.

## Introduction

Based on prior systematic reviews and meta-analyses, it can be assumed that there is a beneficial relationship between R/S and health [1]. If the two parameters "religion/spirituality" (R/S) and "mental health" are defined very broadly, at least 20 meta-analyses can be found to date that together have a cumulative effect size (Pearson *r*) of .15 [2]. However, the overall association between R/S and mental health can only be considered modest [3]. In a recent meta-analysis of English-language longitudinal studies, Garssen and colleagues [4] report a cumulative effect size of *r+* = .08. This figure is remarkably consistent with previous analyses of the overall relationship between R/S and psychological adjustment: Bergin [5], nearly 40 years ago, found a mean correlation of *r+* = .09, and Hackney and Sanders [6], some 20 years ago, a mean correlation of *r+* = .10. In German-speaking countries, the weighted average correlation (*r+* = .03) is also positive but lower in comparison [7].

However, even in meta-analyses, which examine relatively homogeneous religious/spiritual populations within a defined cultural domain, effect sizes between R/S and mental health are of only limited value. This is because different aspects or dimensions of R/S can be expected to correlate differently with different aspects of mental health. For example, Garssen and colleagues [4] distinguish between eight R/S predictors and only two of them (public religious activities, importance of religion) correlate significantly with mental health. Another recent meta-analysis reports "mostly medium effect sizes (*r+* = .25 to *r+* = .30) for the associations of positive God representations with well-being" [8]. Because the association between God representations and psychological well-being was significantly stronger than the correlations in other systematic reviews, the authors argue that future research should focus primarily on this specific dimension of R/S. It is also known that negative measures of R/S (e.g., spiritual struggles or negative religious coping) are significantly more (negatively) associated with mental health [9]. Thus, to get a clearer picture of the R/S-mental health connection, it is essential to decompose the multidimensional phenomenon of R/S back into its individual dimensions and relate these dimensions to specific mental health variables.

The choice of mental health variables also influences the direction and strength of the association. First, a distinction can be made between positive (happiness, optimism, well-being, etc.) and negative indicators of psychological functioning (anxiety, addiction, depression, etc.). Now, it is quite conceivable that certain aspects of R/S correlate differently with certain positive/negative indicators of mental health. For example, the association between R/S and lower rates of depression is known to be particularly strong [10]. In principle, therefore, what was said for the R/S variable applies equally to the construct “mental health”: either this variable should be considered in a differentiated way for meta-analytic operations (Garssen and colleagues [4], for instance, divide the outcome measures of mental health into five categories), or one chooses a single indicator for mental health and examines how this indicator is related to different aspects of R/S (as Abdel-Khalek and colleagues [11] have done for religiosity and anxiety in Arab Muslim samples).

### Current meta-analysis

In the present meta-analysis, great emphasis is placed on a differentiated view and fine-grained analysis of the R/S variable. In order to obtain more robust results in R/S-mental health connection research, it is important to examine precisely whether and to what extent measurement instruments capture "religion" and/or "spirituality" and which of the multiple aspects of R/S play a dominant role in the (sub-)scales. Therefore, we conduct a qualitative analysis of all questionnaire items and classify the potential R/S measurement instruments on this basis.

For mental health, on the other hand, we selected a single indicator that (a) is widely used in empirical research, for which there is (b) a psychometrically valid measurement instrument, and that (c) is related to R/S from a theoretical point of view. In this systematic review, sense of coherence was chosen as an indicator of mental health because coherence—understood as a meaning- and trust-producing meta-resource—has been considered a mechanism capable of explaining the relationship between R/S and psychological health since the early dawn of R/S-health connection research.

Already 1987, sociologist of religion Ellen L. Idler introduced the coherence hypothesis into the field of study [12]. In the meantime, it is well theorized and researched that meaning-making is a key resource for psychological functioning [13] and that R/S can help people produce meaning privately or collectively [14]. Precisely because the coherence hypothesis emphasizes the meaning dimension of the R/S-health connection, it retains its explanatory power in view of the individualized appropriation of religious/spiritual offers of meaning on the “commodity market of transcendencies” [15] and in view of the increasing diversification of post-secular identities and worldviews [16].

Over time, Idler’s coherence hypothesis has evolved into the sense of coherence hypothesis (see [17] for a detailed treatment of the reception history). While Idler [12] understood coherence as a sense of meaning based on religiously reinforced optimism/fatalism, the sense of coherence hypothesis states that R/S and mental health are mediated by Antonovsky’s concept of sense of coherence (SOC), a basic life orientation characterized by a comprehensive and dynamic sense of trust [18]. Some twenty years ago, George and colleagues [19] asserted that the SOC hypothesis has received the most empirical support and claimed that SOC could explain 20–30% of the relationship between religious involvement and health. That SOC may be a particularly promising variable in the R/S-mental health connection has also been suggested in a recent meta-analysis: “The most important recommendation is to employ outcomes that are theoretically closer to R/S than distress and well-being, such as sense of coherence, […]” [4].

However, hitherto no systematic synthesis of this type of research has been published. Hence, the aim of our meta-analysis is to systematically examine the statistical associations between R/S and Antonovsky’s SOC. In this way, it makes an important contribution to testing the plausibility of the SOC hypothesis within R/S-mental health connection research. In connection with the SOC hypothesis, we are also interested in which form of transcendence reference, religion or spirituality, correlates more strongly with SOC. For this reason, in the following chapter we will heuristically define what we mean by "religion" and "spirituality" in the context of our meta-analysis and which mixed forms of R/S exist—a decision that will also determine how we will classify the R/S measurement instruments used in the included studies. Since we assume that both religion and spirituality are multidimensional constructs, our classification of the included R/S scales will not only decide whether the scale is an R scale, an S scale, a mixed scale, or no R/S scale according to our heuristic definition; we will also classify which dimension or aspect of R/S each R/S scale primarily measures. In this way, we aim to answer the question of which R/S aspects (faith, collective practice, God-relationship, etc.) are most strongly/weakly associated with SOC. However, since not only religion and spirituality can be understood as multidimensional constructs, but also the SOC scale generated by Antonovsky is conceived three-dimensionally, we also aim to answer the question with which SOC subscale (comprehensibility, manageablility, meaningfullness) the R/S scales correlate most clearly.

Another aim of the present meta-analysis is to identify moderator variables that have an effect on the statistical relationship between R/S and SOC. The selection of potential moderators is strongly determined by the type and breadth of data we can extract from the included studies. Nonetheless, we considered in advance which variables we would like to extract on theoretical grounds because we hypothesize that they might have a moderating effect on the R/S-SOC relationship. We formulated the following hypotheses regarding the moderator variables that we will consider in this meta-analysis:

- **Age group**: Since both religiosity and SOC increase with advancing age [20–22], it can be assumed that the R/S-SOC connection is stronger in older samples.
- **Gender**: Some studies have shown that the SOC of boys and men is higher than the SOC of girls and women [23,24]. Other studies, however, have found no gender differences in SOC strength among adolescents [25] and adults [26,27]. In the area of religious commitment, a gender gap has been noted for years: By and large, women appear to be more religious/spiritual [28,29]. We assume that these effects average out and no significant gender differences in the R/S-SOC connection come into play.
- **Health status**: According to Antonovsky’s theory of salutogenesis, people who are mentally or physically ill tend to be near the dis-ease pole of the health ease/dis-ease continuum [18]. Since, according to theory, the position on the continuum depends strongly on a person’s SOC strength, the SOC should generally be lower in clinical samples. On the other hand, R/S may increase in the context of illness, as religious/spiritual coping resources are often mobilized in difficult life situations [30]. Thus, we make the tentative hypothesis that the R/S-SOC connection is somewhat stronger in clinical samples.
- **Cultural value orientation**: Culture, especially cultural belonging and stability, is an important source for the development of a strong SOC [31,32]. Antonovsky [18] assumed that in countries or (sub-) cultures that have a shared canon of fixed yet flexible religious/spiritual beliefs, practices and values, the R/S-SOC connection is likely to be stronger. Therefore, drawing on the Inglehart-Welzel World Cultural Map (https://www.worldvaluessurvey.org), we hypothesize that the statistical association of R/S and SOC will be lower in countries with secular (post-religious) values than in countries committed to traditional (religious) values.
- **Religious affiliation:** It stands to reason that the R/S-SOC connection is more pronounced in religious and/or spiritual populations than in non-religious/non-spiritual or heterogeneously composed groups, since only for people of faith does R/S have relevance as a generalized resistance resource or SOC source.
- **Time period:** Garssen and colleagues [4] identified the publication year of the studies as a significant moderator in the R/S-health connection. In their meta-analysis, studies before 2000 and after 2009 show higher effect sizes. We hypothesize that further development and specification of R/S instruments will lead to stronger correlations being measured in more recent times.

## Preliminary conceptual understandings

### Conceptualizing and measuring sense of coherence

Antonovsky [18] formally defined SOC as

*a global orientation that expresses the extent to which one has a pervasive*, *enduring though dynamic feeling of confidence that (a) the stimuli deriving from one’s internal and external environments in the course of living are structured*, *predictable*, *and explicable*, *(b) the resources are available to one to meet the demands posed by these stimuli*, *and (c) these demands are challenges*, *worthy of investment and engagement*.

From the definition follows that Antonovsky conceptualized SOC as a three-dimensional construct. He named the three SOC components (a) comprehensibility, (b) manageability, and (c) meaningfulness. They represent the cognitive, instrumental, and motivational or emotional aspects of SOC.

Based on a qualitative pilot study, Antonovsky [18] developed a 29-item-long *Orientation to Life Questionnaire* (SOC-29) and a short version of this scale (SOC-13) to measure SOC. Each item is rated on a 7-point Likert scale. Thus, the total SOC score ranges from 29 to 203 (SOC-29) and from 13 to 91 (SOC-13) respectively. A high SOC score is associated with the presence of resistance resources, adequate tension and stress management skills and the enduring feeling of confidence that things will work out as well as reasonably expected.

Antonovsky supposed that one’s SOC is developed in the first third of one’s life and that it remains relatively stable over time. Some studies confirm Antonovsky’s stability hypothesis [33], while some other studies suggest that SOC is much more dynamic and changeable than initially assumed [34]. Schnyder and colleagues [35] have tried to propose a middle ground between the positions: “Antonovsky’s SOC can probably be seen as a relatively stable (trait) measure, showing some degree of (state) variability when a person is faced with a drastic life event.”

Even though Antonovsky differentiated between three SOC components on a theoretical basis, he emphasized the holistic idea of his construct [18]. On an empirical basis, too, he underlined that SOC should be dealt with as a measure of one global factor [36]. Confirmatory factor analysis in various studies of SOC confirm that a single-factor model best fits the data [37]. By the same token, other studies suggest that the factorial structure seems to be multi- rather than unidimensional [38].

Antonovsky’s SOC scales show fairly good to excellent reliability. For the SOC-29 scale the Cronbach’s *α* coefficient of internal consistency has ranged from .82 to .95 in 26 studies analyzed by Antonovsky [36] and from .70 to .95 in 124 studies reviewed by Eriksson and Lindström [39]. The *α* values for SOC-13 range from .70 to .92. Cronbach’s *α* is rarely reported for the SOC sub-scales.

High correlations between SOC and some aspects of mental health have led some scholars to argue that SOC is not a discrete construct but rather an inverse measure for psychological distress, negative affectivity or neuroticism. Geyer [40], for example, holds that the “[…] very high negative correlations between SOC and depression/anxiety suggest that the instruments used may assess the same phenomenon, but with inverse signs”. On the other hand, these findings could also be interpreted as validation of Antonovsky’s SOC scale [41], because it is expected that SOC will highly correlate with mental health measures (convergent validity) and that it will predict mental health outcomes (predictive validity).

In our context, it is of particular interest whether the content of some SOC items could be partly regarded as overlapping the content of items measuring R/S. Coming from a methodological point of view, the conceptual and contentual discrimination between SOC and R/S is a premise for our meta-analysis [42]. Antonovsky attested religion an important role as a macrosocial generalized resistance resource [43], but he did not incorporate items dealing with transcendent aspects in his SOC questionnaire [44]. In accordance with this finding, Piedmont and colleagues [45] conclude that “SOC appears more oriented towards a secular-type of meaning-making” (p. 7). This interpretation is backed up by factor analyses showing that R/S and SOC items load on different factors [46].

### Conceptualizing religion and spirituality

**Fig 1:**
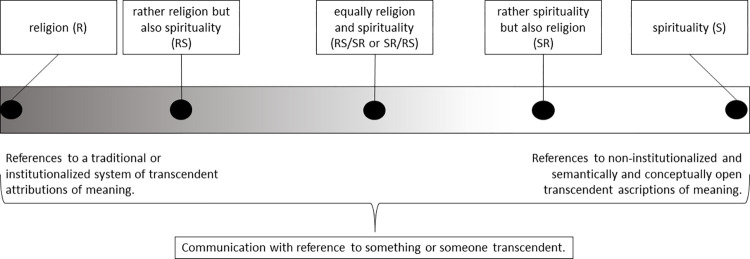
Continuum model of religion/spirituality.

In the context of this meta-analysis, we conceptualize religion and spirituality as endpoints of a continuum (Fig 1). Hence, the controversial question of whether religion or spirituality is the broader construct no longer arises [47]. Both are equal poles of a dynamic field and therefore in their clearest expression, extreme manifestations of one and the same human mode of perception. In this way, common polarizations invoked to conceptually distinguish religion from spirituality can still be used to make an analytical differentiation. Religion in its "pure form" refers to transcendent attributions of meaning that are usually communicated with recourse to a handed-down system of moral teachings, rituals, practices and precepts, which is supported by an often hierarchically organized system of specialists and is institutionally anchored in a society. Idealtypically, the concept of spirituality describes rather subjective transcendent attributions of meaning, whose communication does not necessarily have to be linked to a fixed religious system of beliefs and practices, but rather is based on the individual-creative appropriation of religious concepts and on personal search and experience. At the same time, the continuum model makes it clear that the separation of religion and spirituality is not conceived as a simple dichotomy, but that there are fluid transitions and various mixed forms between the poles. If one wants to punctuate the continuum for analytical reasons, some ideal-typical hybrids can be delineated between religion (R) and spirituality (S): rather religion but also spirituality (RS), equally religion and spirituality (RS/SR or SR/RS), and rather spirituality but also religion (SR).

The phenomenal domain covered by the R/S continuum differs from the domain of the non-religious/non-spiritual by communications with reference to something or someone transcendent. The distinction transcendence/immanence is prominently used as “guiding difference” (*Leitdifferenz*) in a systems theory approach to mark the boundary between the secular and the religious [48]. Luhmann, a prominent representative of systems theory, understood “transcendence” as a point of view by which an intrinsically indeterminate immanent world (e.g., a lightning strike) is observed at the same time as something determinable and interpretable (e.g., Thor’s hammer blow). Or in Luhmann’s words: “a communication is always then religious when it observes immanence from the point of view of transcendence […]. Only when viewed from transcendence do events in this world acquire a religious meaning” ([49]; translated and cited by [50]).

The recourse to Luhmann helped us to clarify our concept of transcendence and thus the distinction between R/S and non-religion/non-spirituality. Whether a transcendent interpretation is rather religious or rather spiritual depends (a) on the reference system used and (b) the degree of definiteness. Religious systems of meaning usually have certain cognitive schemes in order to interpret the immanent in a transcendent way, e.g., as God’s work or the work of the devil in the case of Christian tradition. These schemes of interpretation are often institutionalized (e.g., in the form of theologies), reinforced by collective practices, and perpetuated by a faith community. In comparison, spirituality is characterized by a greater scope of individual interpretation and by greater indeterminacy: the immanent is indeed interpreted transcendently, but the reference system chosen to describe what has been experienced remains rather vague (e.g., a higher power or an invisible energy), may vary, and/or is not based on institutionalized traditions.

## Materials and methods

### Search strategies

Literature search was designed to retrieve studies that report a statistical correlation between an original SOC questionnaire and an R/S measure. In this paper, two different literature search styles were used. For a start we made use of the classical “building blocks” technique [51]: The query was divided into facet A (salutogenesis), facet B (R/S), and facet C (quantitative design). According to this facet structure, Academic Search Ultimate, APA PsycINFO, APA PsycArticles, CINAHL, EMBASE, MEDLINE, PSYINDEX, PubMed, SocINDEX, and Web of Science were searched using the Boolean phrase depicted in S1 Table. In some cases, the search algorithm had to be adapted to the query boxes of the databases in order to obtain a reasonable number of results. The exact search queries for each database are compiled in S2 Table. The last search run was conducted by the first author on March 3, 2023.

In addition to database searches, we drew on the “berry picking” model of information retrieval proposed by Bates [52]. As Booth [51] points out, “berry picking” “is actually a meta-strategy, including six specific tactics”: footnote chasing, citation searching, journal run, area scanning, subject searches, and author searching. Most of the “berry picking” work was done using not only online databases but also online public access catalogs (OPACs), digital libraries, and Web search engines. The big advantage of “berry picking” is the opportunity to track down studies overlooked by systematic search strategies. Disadvantages include high time cost and the lack of replicability and transparency. The “berry picking” process was stopped on March 3, 2023.

### Inclusion and exclusion criteria

Each study found was screened for eligibility. All of the following five criteria (I1-I5) have to be met in order for studies to be included in the review:

1. The study must be written in English or German.
2. The study must be published in a journal, as a book, as a book chapter or as a dissertation/habilitation thesis.
3. The study must use one of Antonovsky’s original questionnaires (SOC-29 or SOC-13) to measure SOC quantitively.
4. The study must use at least one instrument/item to measure R/S.
5. The study must report at least one correlation coefficient between a R/S (sub-)scale and a SOC (sub-) scale.

A study was excluded from the meta-analysis if any of the following criteria (E1-E5) applied:

1. The authors were unable to obtain the full text of the study.
2. The study results were reported elsewhere and that work met the above inclusion criteria.
3. The sample size is less than or equal to ten.
4. The assessment of the study with our critical appraisal tool showed that the study was of limited suitability for the questions of this meta-analysis (“poor fit”).
5. The instrument/item used to assess R/S did not qualify as an R/S measure according to our classification. (Less than 67% of the questionnaire items have no relation to R/S, using our heuristic definition of R/S formulated above to classify the items. The exact methodological procedure is described below.)

A rationale for the choice of the inclusion and exclusion criteria is provided in S3 Table. If certain information (e.g., the correlation coefficient or the wording of a self-developed R/S questionnaire) were missing, that would be necessary for the study not to be excluded from the meta-analysis, the first author contacted the study authors via e-mail, if possible.

### Critical appraisal of studies

Each study that met the inclusion criteria and had not been excluded based on one of the first three exclusion criteria was critically appraised to determine whether it has a sufficient “fit” to the research questions of this meta-analysis. We did not use an already validated instrument to assess the “quality” of the studies for two reasons: (1) The Cochrane Collaboration was critical of the use of “quality” appraisal tools because the scales often mixed different concepts of “quality” and therefore the validity of a summary score was limited [53]. (2) Even if an assessed study was methodologically rigorous and met all standard “quality” criteria, such an critical appraisal would say little about the “quality” of the effect size, which is of particular relevance for our purposes. We include many studies with entirely different research contexts, study designs, and objectives that only "coincidentally" report a correlation coefficient between an R/S variable and SOC. Therefore, we did not need an appraisal tool that only asks for general non-specific “quality” criteria, but one that assesses the “fit” to the questions of our meta-analysis. For example, the question of whether the included study uses a valid R/S measurement instrument plays a special role for our assessment

To decide whether a study “fits” the context of our investigation, we developed an appraisal tool with three domains: (a) study population, (b) R/S measure, and (c) data analysis (S1 Tool). The tool incorporates criteria that have already been used [54,55], but also contains criteria that have been developed specifically for our purpose.

The first domain (study population) includes five criteria (e.g., sample size) with a score range from 0 to 10 points, the second domain (R/S measure) includes four criteria (e.g., Cronbach’s α) with a score range from 0 to 8, and the third domain (data analysis) includes three criteria (e.g., precise report of effect size) with a score ranging from 0 to 6. The overall result is recorded in a tripartite form (e.g., 6/5/2). Each study was assessed twice by the first author (intrarater reliability) and then reviewed by another author of the research team (interrater reliability). If there was a disagreement between the raters, it was discussed and a value was agreed upon.

The reviewed studies are classified into three categories according to the following criteria:

- **poor fit**: The second value (R/S measure) is less than 2 and/or the overall score is less than or equal to 7.
- **moderate fit**: The second value (R/S measure) is between 2 and 5 and the overall score is between 8 and 15.
- **good fit**: The second value (R/S measure) is above 5 and the total score is above 16.

Studies with a “poor fit” were excluded from the meta-analysis.

### Classification of measuring instruments according to the continuum model of religion and spirituality

The designation of a measuring instrument can be deceptive. Just because terms such as “spiritual” or “religious” are mentioned in the title does not necessarily mean that the questionnaire items also measure religion and/or spirituality. For instance, Koenig [56] has convincingly argued that Peterman and colleagues’ *Spiritual Well-Being* scale (FACIT-Sp) is a measure of positive feelings rather than a measure of spirituality in the strict sense. The reverse case also occurs: Scales that do not have terms such as “religious” or “spiritual” in the title may nevertheless contain items that measure R/S. Therefore, an item-specific content analysis of the questionnaire items is necessary to ensure that the measures that may be included in our meta-analysis actually capture the R/S variable.

We classified each item of all potential R/S measures used in the included studies according to our heuristic continuum model of R/S (Fig 1). For this purpose, the continuum is divided into the five categories introduced above: religion (R), rather religion but also spirituality (RS), equally religion and spirituality (RS/SR or SR/RS), rather spirituality but also religion (SR), and spirituality (S). Each item is assigned one of these five labels or, alternatively, it is classified with the symbol X, which means that this item has no reference to someone or something transcendent. To determine whether a (sub-) scale as a whole should be considered an R-measure, RS-measure, RS/SR-measure, SR/RS-measure, SR-measure, or S-measure, the percentages of S-items and R-items within the total questionnaire items are calculated. If, in the course of content analysis, a (sub-)scale is found not to include enough R/S-related items (less than 67%) to be classified as an R/S measure in our sense, the study (insofar as it does not include at least one other valid R/S instrument) was excluded from the meta-analysis.

In order to make the classification process intersubjectively more comprehensible, the first author developed a coding aid with “trigger words” (S4 Table). Each potential R/S measure was classified twice by the first author and then reviewed by another author. If different opinions prevailed in the classification, the research team discussed until a consensus was reached. The methodological intricacies of the classification process are explained in more detail in S1 Text.

### Classification of measuring instruments according to their religious/spiritual aspects

Previous meta-analyses have made distinctions between different dimensions of R/S and categorized the included R/S measures accordingly. The used coding schemes ranged from three [6] to 21 categories [7]. Garssen and colleagues [4] assigned the R/S scales to eight categories. In the categorization process, their “aim was to stay as close as possible to the names and categories used in the reviewed articles” (p. 4). Zwingmann, Klein and Büssing [57] undertook a more theory-driven classification of R/S instruments according to their primary measurement intention. In this meta-analysis we made use of a qualitative content analysis [58] of the items of all R/S measures used in the reviewed studies as the basis for classification. In accordance with the logic of qualitative coding, no categories were predetermined in advance; instead, the category system emerged stepwise from the analysis of the questionnaire items themselves. In this way, over time, certain categories are consolidated, revised and made more precise, and finally provided with anchor examples and descriptions. Each item was coded twice by the first and then reviewed by the second author. In case of a disagreement, the item in question was discussed by the author team.

To calculate the predominance of certain R/S aspects within a measurement instrument, a formula was developed that determines how often an R/S aspect must occur relative to the number of items in order to be considered dominant: If the scale included 1–4 items, 50% of the items had to address the same R/S aspect for that R/S aspect to be classified as dominant. For longer measuring instruments, the percentage was reduced accordingly (33% of 5–13 items; 25% of 14–25 items; 20% of 26 or more items). The percentages chosen are not absolute, of course, but have emerged as sensible benchmarks after a lengthy trial and error process. If more than one-third (33%) of the items addressed negative aspects of R/S (and were not reverse coded), the (sub-) scale was classified as a negative measurement instrument (symbolized with the abbreviation N).

### Coding matrix of the included studies

Each included study was tabulated according to the following coding system: (a) serial number (numbering is according to alphabetical sorting by author surname), (b) author/s, (c) year of publication, (d) time period (1988–1999; 2000–2011; 2012–2023), (e) country (usually the nationality of the sample, but if the researchers are from another country, this is added in brackets after it), (f) cultural map (assignment of the country according to the Inglehart-Welzel World Cultural Map [59]: African-Islamic; Catholic Europe; Confucian; English-Speaking; Orthodox Europe; Protestant Europe; Latin America; West & South Asia), (g) UN geoscheme (17 subregional groups), (h) study type (article; book; chapter; dissertation), (i) study design (cross-sectional; longitudinal), (j) survey method (one or several of these codes: administered; in-person; mail; online; telephone), (k) response rate (usually in % and/or in numbers), (l) sample description, (m) sample type (mentally ill; migrants; population; refugees; religious officials; somatic patients; specific group; specific profession; students), (n) major life event (yes; no), (o) health status (clinical; non-clinical), (p) type of disease, (q) sample size, (r) size group (small [10–99]; middle [100–299]; large [>299]), (s) number of women, (t) number of men, (u) number of diverse people, (v) gender group (women; mostly women [at least two thirds of the sample are women]; mixed; mostly men [at least two thirds of the sample are men]; men), (w) average age of the sample, (x) standard deviation age, (y) age range, (z) age group (classification is based on the average age of the sample: <18; 18–30; 31–60; >60), (aa) religious affiliation (If possible, the percentage of the sample belonging to a particular worldview is indicated. Options include not only the “major” religions, but also indicators such as “atheist”, “non-religious” or “spiritual”), (ab) religion group (If 100% of the sample belongs to a belief system, the sample is coded as, for example, “Christian” or “Muslim”. If two thirds, i.e., over 66.6%, belong to a belief system, the sample is coded as, for example, “mostly Christian” or “mostly Muslim”. In the other cases, the code “heterogenous” is used), (ac) R/S measure (The name of the R/S measuring instrument is listed here. If more than one instrument was used in a study, the study is listed in the table a corresponding number of times. However, each study is only included once in every meta-analytic procedure), (ad) classification of the R/S measure (The code corresponds to the classification of the respective R/S instrument using our R/S continuum model, i.e., the codes are: R; RS; RS/SR; SR/RS; SR; S), (ae) dominant R/S aspects (Listed here are which R/S aspects dominate in each R/S measure according to our content analysis), (af) SOC measure (SOC-13; SOC-29), (ag) SOC item mean score (range, 1 to 7), (ah) SOC strength (weak: SOC item mean score < 4.1; moderate: SOC item mean score between 4.1 and 5.3; strong: SOC item mean score > 5.3), (ai) total SOC mean score (SOC-13 range, 13 to 91; SOC-29 range, 29 to 203), (aj) SOC range, (ak) *SD* SOC, (al) R/S mean score, (am) R/S range, (an) *SD* R/S score, (ao) correlation SOC-C (comprehensibility) and R/S measure, (ap) correlation SOC-MA (manageability) and R/S measure, (aq) correlation SOC-ME (meaningfulness) and R/S measure, (ar) correlation of SOC total mean score and R/S measure, (as) correlation method (Pearson; Spearman), (at) level of significance (*ns*; p < .05; p < .01; p < .005; p < .001; p < .0005; or exact *p* value), (au) score of the 1^st^ domain of the appraisal tool (range, 0 to 10), (av) score of the 2^nd^ domain of the appraisal tool (range, 0 to 8), (aw) score of the 3^rd^ domain of the appraisal tool (range, 0 to 6), (ax) total score of the appraisal tool (range, 0 to 24), (ay) fit assessment (poor; moderate; good), (az) personal communication (yes; no).

Extraction of data was performed twice for each study by the first author and have been checked by authors of the team.

### Meta-analytical procedures

Basis for all statistical calculations is a Microsoft® Excel-sheet, on which the extracted data of all included studies are listed. Bivariate correlation coefficients (Pearson’s product-moment correlations or Spearman’s rank correlations) between the R/S (sub-) scales and total SOC are set as effect sizes (ESs) and listed in column AR of the Excel table. If correlations are also reported between R/S measures and one or more of the SOC sub-components (comprehensibility, manageability, meaningfulness), these ESs are also listed. For each subgroup analysis, an extra Excel table (reduced to necessary information) is created from the total Excel sheet to ensure that each study sample (even if reporting multiple effect sizes) is included only once in each analysis. This explains the difference between the number of studies and the number of ESs. A subgroup must consist of at least five different studies and therefore also of at least five ESs. If a study included multiple ESs relevant to a subgroup analysis (e.g., correlations between total SOC and two different S measures), these ESs were combined into an aggregate ES for each study in the respective meta-analysis. However, due to the askew distribution of correlations, we could not simply take the arithmetic mean of the ESs. Thus, we transformed the ESs into Fisher z-values, weighted them based on the number of cases, and then transformed the mean back into an *r*-value.

Subgroups were analyzed using R statistical software (RStudio, Version 1.4.1106) with the packages robumeta, metafor, and dplyr. The program converts the list of (aggregated) *r*-values of the uploaded Excel table to z-values and calculates a cumulative ES *r+* (including standard error, level of significance, and 95% confidence intervals) using random effects models.

To test whether the difference in ESs between two subgroups is statistically significant, *z-*tests for two independent samples have been calculated [60].

Heterogeneity was examined using Baujat plots, *τ*^*2*^, *I*^*2*^ statistics, *H*^*2*^ index, and *Q* test. For the detection of any outlier studies, primarily the Baujat plot was used. Studies that strongly contributed to heterogeneity (horizontal axis) and that strongly biased the overall result (vertical axis) were omitted gradually from the subgroup until no study was visible as an extreme outlier in the upper right corner of the Baujat plot. Each of these omission steps is recorded and the subgroup analysis is recalculated to keep the procedure transparent and the change in values due to the leaving-out of studies traceable. A maximum of three studies were excluded in a subgroup analysis, and these were often the same studies despite different subgroups.

Publication bias was examined using funnel plots. We tested for funnel plot asymmetry using a mixed-effects meta-regression model with standard error as predictor [61] and a rank correlation test (Kendall’s *τ*).

### Transparency and openness

This meta-analysis follows the standards developed by the Transparency and Openness Committee [62]. All materials are either publicly available on the Open Science Framework (https://osf.io/2smz5/) or are available on request from the first author. With the supporting information, all methodological steps can be traced in detail. Tables for sub-group analyses can be created from the Excel spreadsheet that captures all extracted data (S5 Table). One such sub-table created by us is also available as an example (S6 Table). All other sub-tables used for subgroup analyses will be provided by the first author upon request. Using the R code, which is also publicly available (S1 Code), all our calculations can be reproduced.

In presenting our approach as well as our results, we followed the American Psychological Association (APA) Meta-Analysis Reporting Standards (MARS) [63] and the PRISMA 2020 statement [64]. This meta-analysis was registered with PROSPERO on 2 April 2021 (registration number: CRD42021240380). After discussion of an early draft manuscript, suggestions from commentators were incorporated, resulting in minor modifications of the preregistration plan.

## Results

### Summary of the included and excluded studies

**Fig 2:**
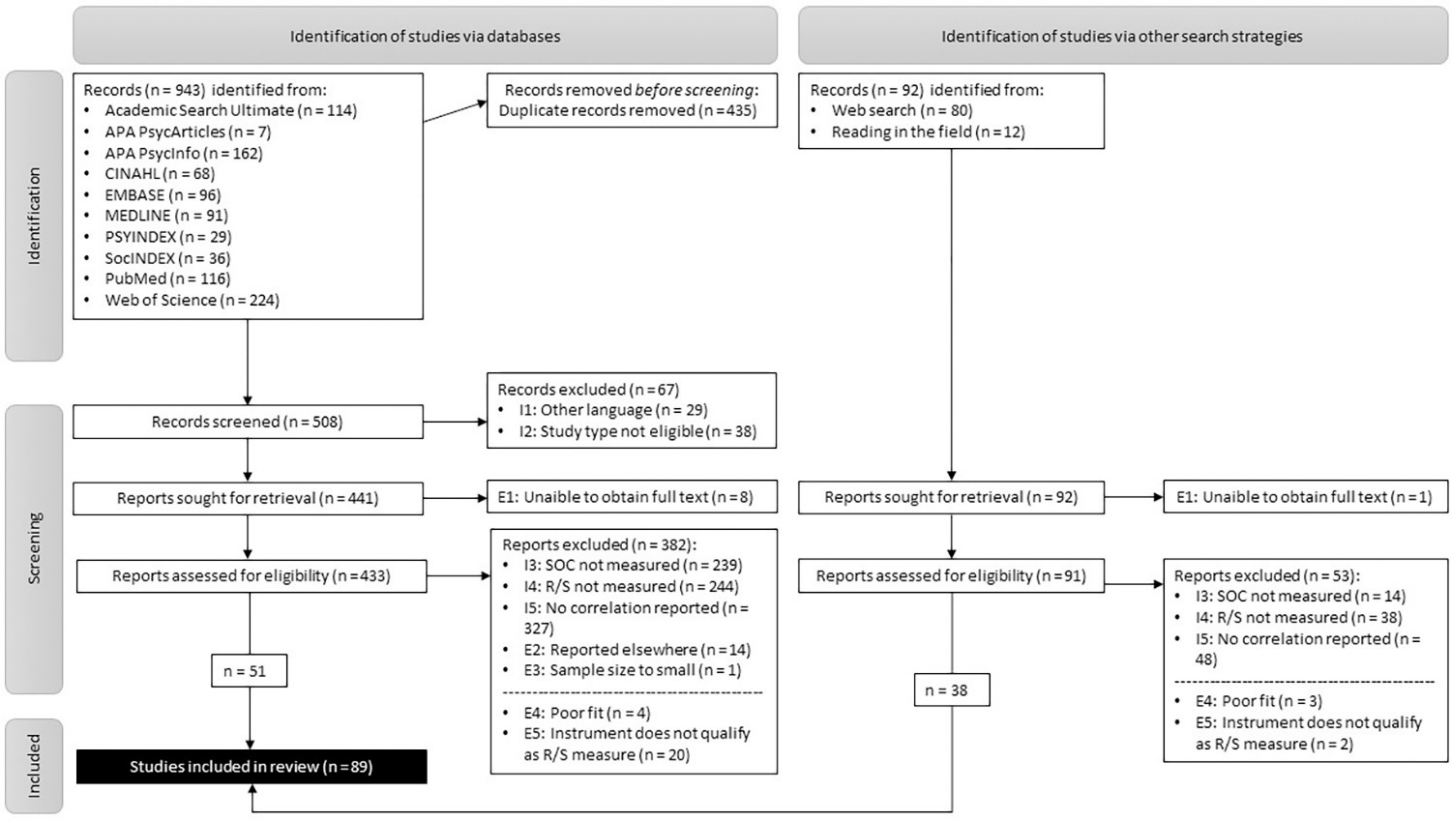
PRISMA flow diagram.

By searching databases, we identified 943 studies (cleared from duplicates: 508) that may report a statistical correlation between R/S and SOC. Of these studies, 51 met our inclusion and exclusion criteria. The targeted search for additional studies that fell through the grid of the database search (“berry picking”) revealed a further 92 studies. According to our list of criteria, 38 additional studies were included. Thus, in total, our meta-analysis is based on 89 studies with 67,913 participants. Of these 89 studies, 17 studies could only be included in the meta-analysis because missing information could be obtained through email contact (S7 Table). A list of the 444 studies that we sought for retrieval, assessed for eligibility and that were subsequently excluded on the basis of different criteria (I3-I5 and E1-E5) is included as supporting information (S8 Table). The search and selection process is illustrated in a PRISMA flow diagram (Fig 2).

In the methods sub-section "Coding matrix of the included studies", we defined 52 codes that are extracted for each included study. The result of this extraction process is listed in its entirety in an Excel spreadsheet provided as S5 Table. Our codes from (a) to (az) correspond to the column labels of the Excel table, ensuring easy traceability. Table 1 briefly presents the 89 included studies.

**Table 1:** Measurement and effect size information of study samples included in the meta-analysis.

Study	Sample	R/S measure (classification)	Dominant R/S aspects	*r*	Appraisal
Abel et al. (2014) [65]	63 undergraduate students and 61 persons recruited from social networking sites	Intrinsic Religiosity (R)	Emotion, individual practice, way of life	NA	5/5/1
Aderhold et al. (2019) [66]	251 cancer patients	Religious/Spiritual Coping (RS)	Coping, God relationship, power, trust	.000^ns^	8/6/3
Ahmad (2012) [67]	121 South Asian American women	Religion-Spirituality (R)	Coping, efficacy	.150^ns^	10/6/3
Arya & Davidson (2015) [68]	100 trauma victims	Spiritual Change (RS/SR)	Belief, development, understanding	.334**	4/4/2
Basson and Rothmann (2002) [69]	67 pharmacists	Turning to Religion (R)	Coping, effort, frequency, God relationship, trust	.130^ns^	1/6/2
Berger et al. (2016) [70]	190 students from universities in Guadalajara	General Religiosity (RS)	Emotion, God concept, God relationship	.150	8/8/3
Berger et al. (2016)	190 students from universities in Guadalajara	Connectedness (S)	Afterlife, belief, experience, mysticism	-.050	8/8/3
Bossick (2008) [71]	131 college students, 128 community members and 17 persons who did not report this information	Quest Scale (RS)	Development, intellect, quest	-.040^ns^	7/7/3
Bradbury et al. (2009) [46]	130 non-undergraduate students	Intrinsic/Extrinsic Religiosity Scales (R)	Centrality, individual practice, way of life	-.020^ns^	5/6/2
Bradbury et al. (2009) [46]	130 non-undergraduate students	Revised Paranormal Beliefs Scale (N-S)	Negative aspects	-.310***	5/6/2
Bradbury et al. (2009) [46]	130 non-undergraduate students	Traditional Religious Beliefs (R)	Afterlife, belief	-.220*	5/6/2
Brockhouse et al. (2011) [72]	118 registered therapists that had worked with trauma clients	Spiritual Change (RS/SR)	Belief, development, understanding	-.150^ns^	5/5/2
Büssing et al. (2013) [73]	425 Catholic priests	Spiritual Dryness (N-RS)	Negative aspects	-.483**	8/8/1
Büssing et al. (2016) [74]	3,824 Catholic priests	Daily Spiritual Experiences (RS)	Awareness, emotion, experience, frequency, God concept, God relationship	.317**	9/8/4
Büssing et al. (2016)	3,824 Catholic priests	Spiritual Dryness (N-RS)	Negative aspects	-.339**	9/8/4
Chamberlain and Zika (1988) [75]	188 women having at least one child (≤5) and no paid employment	Intrinsic Religiosity (R)	Centrality, frequency, God relationship, way of life	.135	4/8/4
Conway-Phillips and Janusek (2014) [76]	134 African American women	Spiritual Perspective Scale (S)	Centrality, frequency, individual practice, way of life	.360***	7/7/2
Curtis (2000) [77]	277 prostrate cancer patients	Duke University Religion Index (R)	Centrality, frequency, way of life	.180^ns^	7/7/3
Curtis (2000)	277 prostrate cancer patients	Intrinsic Religiosity (R)	Centrality, way of life	.200**	7/7/3
DeBruyn (2001) [78]	463 undergraduate students	Degree of Spirituality (S)	Centrality, identity, meaning	.156*	7/2/4
Delgado (2007) [79]	181 COPD outpatients	Spiritual Transcendence Scale (S)	Emotion, individual practice, universality	.27^1^**	9/8/3
Dezutter et al. (2010) [80]	207 chronic pain patients	Centrality of Religious Meaning System Scale (RS)	Centrality, way of life	.310***	9/6/3
Diaconescu et al. (2021) [81]	1,243 female medical students	Turning to Religion, shortened (RS/SR)	Coping, frequency, individual practice	-.001^ns^	8/5/4
Diržyte et al. (2003) [82]	132 undergraduate students	Belief in punishing God (N-R)	Negative aspects	-.426**	3/3/2
Dyer (2022) [83]	20,991 students	Religiosity (R)	Affiliation	.020	9/4/2
Ebrahimi Meymand et al. (2021) [84]	156 patients with multiple sclerosis	Spiritual Intelligence Self-Report Inventory (S)	Awareness, intellect, meaning	.080	4/8/4
Ebrahimi Meymand et al. (2021) [84]	156 patients with multiple sclerosis	Critical Existential Thinking (S)	Individual practice, intellect	-.001^ns^	4/8/4
Ebrahimi Meymand et al. (2021) [84]	156 patients with multiple sclerosis	Personal Meaning Production (S)	Meaning, way of life	.131	4/8/4
Ebrahimi Meymand et al. (2021) [84]	156 patients with multiple sclerosis	Transcendental Awareness (S)	Awareness, extraordinary perception, self	.193	4/8/4
Ebrahimi Meymand et al. (2021) [84]	156 patients with multiple sclerosis	Conscious State Expansion (S)	Awareness, control, individual practice	-.005^ns^	4/8/4
Edwards and Besseling (2001) [85]	51 workers at a sawmill in a small rural South African community	Religious Practice (R)	Affiliation, churchiness, collective practice, frequency, organized religion	.410**	9/5/4
Encarnação et al. (2013) [86]	251 people diagnosed with multiple sclerosis	Spiritual Assessment Scale (R)	Belief, emotion, God concept, God relationship, optimism	.194**	6/6/4
Encarnação et al. (2013) [86]	251 people diagnosed with multiple sclerosis	Personal Faith (R)	Belief, emotion, God concept, God relationship, optimism, trust, universality	.025^ns^	6/6/4
Encarnação et al. (2013) [86]	251 people diagnosed with multiple sclerosis	Religious Practice (R)	Collective Practice, coping, emotion, God relationship, individual practice, organized religion, social support	-.009^ns^	6/6/4
Encarnação et al. (2013) [86]	251 people diagnosed with multiple sclerosis	Spiritual Contentment (R)	God concept, God relationship, optimism, trust	.469**	6/6/4
Encarnação et al. (2013) [86]	251 people diagnosed with multiple sclerosis	Spiritual Suffering (S)	Connectedness, coping, emotion	.177*	6/6/4
Farhadi et al. (2022) [87]	260 mothers with disabled children in rehabilitation centers	Spiritual Change (RS/SR)	Belief, development, understanding	.295**	6/5/4
Fernández-Martínez et al. (2019) [88]	463 students taking a degree in nursing	Turning to Religion (R)	Coping, effort, frequency, God relationship, trust	.019^ns^	4/5/3
Ferreira et al. (2020) [89]	1,095 adults aged 20 years or older	Spiritual Involvement and Beliefs Scale (S)	Frequency, individual practice	.168	6/6/0
Forstmeier et al. (2009) [90]	103 former child soldiers of World War II	Spiritual Change (RS/SR)	Belief, development, understanding	.060^ns^	5/6/2
Gabrielsen et al. (2012) [91]	244 pupils from high schools	Religion (RS)	Afterlife, belief, centrality, God concept	.051^ns^	6/6/2
Gabrielsen et al. (2013) [92]	244 pupils and 54 young people with mental health problems	Religion (RS)	Afterlife, belief, centrality, God concept	.050	4/5/1
Gavulic (2018) [93]	460 adults who experienced suffering	Awareness (R)	Awareness, communication, experience, God concept, God relationship, influence	.271	7/6/1
Gavulic (2018) [93]	460 adults who experienced suffering	Disappointment (N-R)	Negative aspects	-.482	7/6/1
Gavulic (2018) [93]	460 adults who experienced suffering	Grandiosity (R)	Efficacy, God concept, God relationship, grandiosity, individual practice, self-image	-.078	7/6/1
Gavulic (2018) [93]	460 adults who experienced suffering	Impression Management (R)	Centrality, churchiness, emotion, frequency, grandiosity, individual practice, organized religion, prosociality, self-image	.224	7/6/1
Gavulic (2018) [93]	460 adults who experienced suffering	Instability (N-R)	Negative aspects	-.562	7/6/1
Gavulic (2018) [93]	460 adults who experienced suffering	Realistic Acceptance (R)	Effort, emotion, God concept, God relationship, quest, trust	.334	7/6/1
Ghazinour et al. (2004) [94]	50 Iranian refugees resettled in Sweden treated in a psychiatric hospital and 50 not in any treatment	Spirituality (SR)	Belief, coping, meaning	.730***	5/6/1
Gibson (2000) [95]	162 African American breast cancer survivors	Spiritual Perspective Scale (S)	Centrality, frequency, individual practice, way of life	.160*	9/8/4
Goulding (2004) [96]	88 undergraduate students	Australian Sheep-Goat Scale (S)	Experience, extraordinary perception, paranormal beliefs	.183^ns^	5/4/4
Goulding (2005) [97]	129 paranormal believers and experients	Australian Sheep-Goat Scale (S)	Experience, extraordinary perception, paranormal beliefs	.013^ns^	4/4/2
Hammer et al. (2013) [98]	448 Atheist military personnel and veterans and 789 Atheist civilians and 293 Christian civilians	Original Spiritual Fitness Scale (S)	Belief, meaning	.373	10/8/2
Herbst et al. (2007) [99]	120 married working mothers	Turning to Religion (R)	Coping, effort, frequency, God relationship, trust	.090^ns^	6/6/1
Hossack (1997) [100]	169 religiously homogenous undergraduate students	Extrinsic Orientation (R)	Churchiness, coping, individual practice, social interaction, organized religion, social support	-.100^ns^	6/6/4
Hossack (1997) [100]	169 religiously homogenous undergraduate students	Intrinsic Orientation (R)	Centrality, churchiness, collective practice, intellect	.110**	6/6/4
Hossack (1997) [100]	169 religiously homogenous undergraduate students	Religion as a Means (R)	Centrality, churchiness, development, socialization	.120**	6/6/4
Hossack (1997) [100]	169 religiously homogenous undergraduate students	Religion as a Quest (R)	Autonomy, development, quest	-.160**	6/6/4
Hossack (1997) [100]	169 religiously homogenous undergraduate students	Religion as an End (R)	Centrality, identity	.020^ns^	6/6/4
Hossack (1997) [100]	169 religiously homogenous undergraduate students	Religious Maturity Scale (RS)	Autonomy, development, intellect, quest, truth	-.030^ns^	6/6/4
Israelashvili et al. (2011) [101]	93 Druze and 100 Jewish students at Israeli universities	Religiosity (R)	NA	-.126	6/6/2
Johnson-Migalski (2006) [102]	21 medically stable and 15 medically unstable elderly	Beliefs and Values (RS)	Belief, emotion, God concept, God relationship, idealism, prosociality	.290^ns^	4/4/3
Johnson-Migalski (2006) [102]	21 medically stable and 15 medically unstable elderly	Daily Spiritual Experiences (SR)	Afterlife, belief, experience, mysticism	.300*	4/4/3
Johnson-Migalski (2006) [102]	21 medically stable and 15 medically unstable elderly	Religious Commitment (R)	Centrality, churchiness	.110^ns^	4/4/3
Kerksieck et al. (2016) [103]	8,594 participants from all Catholic pastoral vocational groups	Daily Spiritual Experiences (RS)	Afterlife, belief, experience, mysticism	.310**	10/6/4
Kerksieck et al. (2016) [103]	8,594 participants from all Catholic pastoral vocational groups	Reliance on God’s help (RS)	Coping, God concept, God relationship, optimism, trust	.237**	10/6/4
Khanjari et al. (2012) [104]	115 family caregivers of patients with breast cancer	Negative Religious Coping (N-R)	Negative aspects	-.310**	10/7/3
Khanjari et al. (2012) [104]	115 family caregivers of patients with breast cancer	Positive Religious Coping (R)	Coping, effort, God concept, God relationship, individual practice	.070^ns^	10/7/3
Khanjari et al. (2012) [104]	115 family caregivers of patients with breast cancer	Spiritual Perspective Scale (S)	Centrality, frequency, individual practice, way of life	.230*	10/7/3
Kibour (2002) [105]	91 Ethiopians living in the United States	Religiosity (R)	Collective practice, frequency, identity, organized religion	-.126^ns^	6/2/2
Kim et al. (2020) [106]	315 caregiving partners of MS patients	Spirituality (RS)	Coping, efficacy, frequency	.147^ns^	7/5/2
Kohls et al. (2008) [107]	711 persons recruited from spiritually interested groups 711 persons recruited from spiritually interested groups	Positive Spiritual Experiences (S)	Awareness, experience, extraordinary perception	.070^ns^	7/7/2
Kohls et al. (2008) [107]	711 persons recruited from spiritually interested groups	Transpersonal Trust (SR)	Connectedness, self-transcendence, trust	.120**	7/7/2
Kohls et al. (2008) [107]	711 persons recruited from spiritually interested groups	Psychopathology (N-S)	Negative aspects	-.290**	7/7/2
Krok (2016) [108]	212 Polish older adults	Religious Meaning Questionnaire (R)	Centrality, meaning, intellect, way of life	.200**	10/6/4
Lee (1998) [109]	49 male cocaine dependent outpatients	Religious Well-Being (R)	Belief, coping, God concept, God relationship, meaning	.130^ns^	4/8/3
Littlejohn (1994) [110]	213 women undergraduate students	Extrinsic Orientation (R)	Churchiness, coping, individual practice, social interaction, organized religion, social support	-.190*	4/8/3
Littlejohn (1994) [110]	213 women undergraduate students	Intrinsic Orientation (R)	Centrality, churchiness, collective practice, way of life	.130^ns^	4/8/3
Littlejohn (1994) [110]	213 women undergraduate students	Spiritual Orientation Inventory (S)	Emotion, experience, idealism, meaning	.200*	4/8/3
López et al. (2015) [111]	103 widowed and non-widowed older adults	Beliefs and Practices (RS)	Belief, coping, God concept	.140	6/6/1
López et al. (2015) [111]	103 widowed and non-widowed older adults	Social Support (RS)	Coping, frequency, social interaction, social support	.053	6/6/1
Magnano (2003) [112]	1,122 members of the Light and Life movement in Poland	Religious Hope (R)	Coping, emotion, future, God concept, God relationship, trust, way of life	.250***	9/4/3
Meghani et al. (2018) [113]	18 outpatients with cancer	Faith (SR)	Belief, coping, emotion	.491	5/6/4
Mirsoleymani et al. (2021) [114]	104 family caregivers of cancer patients	Seeking Spiritual Support (R)	Churchiness, collective practice, coping, family resource, frequency, organized religion	.320*	5/6/3
Nahlén & Saboonchi (2010) [115]	80 patients living with chronic heart failure	Turning to Religion, shortened (RS/SR)	Coping, frequency, individual practice	-.190^ns^	8/5/1
Nishi et al. (2010) [116]	118 motor vehicle accident survivors	Spiritual Change (RS/SR)	Belief, development, understanding	.170**	6/4/4
Nolvi et al. (2022) [117]	93 ambulant persons with verified late effects of polio	Turning to Religion, shortened (RS/SR)	Coping, frequency, individual practice	-.160^ns^	6/4/3
Ozaki (2005) [118]	1,397 college students in the Tokyo metropolitan area	Spiritual Sensitivity (S)	Emotion	.036^ns^	5/7/1
Pakenham (2007) [119]	296 persons with MS and 140 caregivers	Spiritual Perspective (SR)	Coping, future, meaning	NA	8/4/2
Pakenham and Cox (2008) [120]	232 caregivers of persons with MS	Spiritual Growth (SR)	Belief, development	NA	5/2/2
Piedmont et al. (2014) [45]	298 adults living in the USA	Prayer Fulfillment (SR)	Coping, efficacy, emotion, individual practice	.270***	7/7/3
Piedmont et al. (2014) [45]	298 adults living in the USA	Religious Involvement (R)	Frequency, individual practice, intellect	.000^ns^	7/7/3
Piedmont et al. (2014) [45]	298 adults living in the USA	Universality (S)	Emotion, universality	.160**	7/7/3
Post-White et al. (1996) [121]	32 cancer patients	Spirituality Index (SR/RS)	Coping, frequency, emotion, individual practice	.150^ns^	10/5/3
Racklin (1999) [122]	210 ethnically-diverse adults	Religious Importance Scale (R)	Coping, meaning, organized religion	.060^ns^	8/8/5
Racklin (1999) [122]	210 ethnically-diverse adults	Spiritual Orientation Inventory (S)	Emotion, experience, idealism, meaning	.200**	8/8/5
Ragger et al. (2019) [123]	266 emergency service members	Spiritual Change (RS/SR)	Belief, development, understanding	.040^ns^	4/5/2
Reguera-García et al. (2020) [124]	84 MS patients	Turning to Religion, shortened (RS/SR)	Coping, frequency, individual practice	-.039^ns^	5/5/3
Renner et al. (2004) [125]	421 Austrian adults	Religiosity (R)	Belief, God concept, God relationship	.120**	7/6/1
Rohani et al. (2010) [126]	298 healthy employees	Negative Religious Coping (N-R)	Negative aspects	-.370	8/6/1
Rohani et al. (2010) [126]	298 healthy employees	Positive Religious Coping (R)	Coping, effort, God concept, God relationship, individual practice	.110	8/6/1
Rohani et al. (2010) [126]	298 healthy employees	Spiritual Perspective Scale (S)	Centrality, frequency, individual practice, way of life	.250	8/6/1
Rothmann and Van Rensburg (2002) [127]	287 uniformed police personnel	Turning to Religion (R)	Coping, effort, frequency, God relationship, trust	.090^ns^	4/6/1
Schonder (2016) [128]	1,623 German and 643 Polish students	Non-Organizational Religious Activity (RS)	Individual practice, intellect, frequency	.166	10/8/5
Schonder (2016) [128]	1,623 German and 643 Polish students	Organizational Religious Activity (R)	Churchiness, collective practice, frequency, organized religion	.189	10/8/5
Schonder (2016) [128]	1,623 German and 643 Polish students	Santa Clara Strength of Religious Faith Questionnaire (RS)	Centrality	.165	10/8/5
Skalski-Bednarz et al. (2022) [129]	600 refugees in Germany	Negative Religious Coping (N-R)	Negative aspects	-.470***	9/8/5
Skalski-Bednarz et al. (2022) [129]	600 refugees in Germany	Positive Religious Coping (N-R)	Coping, effort, God concept, God relationship, individual practice	-.060^ns^	9/8/5
Skowroński & Talik (2021) [130]	390 men imprisoned in penitentiary institutions	Intensity of Religious Attitude Scale (NA)	NA	.244**	6/6/3
Strümpfer (1997) [131]	149 coloured farm w [132] orkers	Extrinsic Religious Orientation (RS)	Churchiness, coping, efficacy, individual practice, social interaction, social support	.080^ns^	5/7/6
Strümpfer (1997) [131]	149 coloured farm workers	Intrinsic Religious Motivation (R)	Centrality, effort, God relationship, way of life	-.130^ns^	5/7/6
Tagay et al. (2006) [133]	251 psychosomatic outpatients and 138 healthy blood donors	Importance of Religion (R)	Centrality, way of life	-.020^ns^	9/2/4
Tagay et al. (2006) [133]	251 psychosomatic outpatients and 138 healthy blood donors	Subjective Religiosity (R)	Centrality, identity	.010^ns^	9/2/4
Torinomi et al. (2022) [134]	1,938 university students	Turning to Religion, shortened (RS/SR)	Coping, frequency, individual practice	-.006^ns^	8/6/3
Unterrainer and Ladenhauf (2008) [132]	241 persons from four different patient groups and 263 healthy people	Connectedness (S)	Afterlife, belief, experience, mysticism	-.030^ns^	9/6/5
Unterrainer and Ladenhauf (2008) [132]	241 persons from four different patient groups and 263 healthy people	General Religiosity (RS)	Emotion, God concept, God relationship	.240**	9/6/5
Unterrainer and Ladenhauf (2008) [132]	241 persons from four different patient groups and 263 healthy people	Global Religiosity (*NA*)	NA	.240**	9/6/5
Unterrainer and Ladenhauf (2008) [132]	241 persons from four different patient groups and 263 healthy people	Religiosity and Search for Meaning (SR)	Coping, effort, meaning	.080^ns^	9/6/5
Unterrainer et al. (2010) [135]	263 Austrian adults	Connectedness (S)	Afterlife, belief, experience, mysticism	-.060	7/8/2
Unterrainer et al. (2010) [135]	263 Austrian adults	General Religiosity (RS)	Emotion, God concept, God relationship	.220**	7/8/2
Unterrainer et al. (2010) [135]	263 Austrian adults	Global Religiosity (NA)	*NA*	.230**	7/8/2
Unterrainer et al. (2013) [136]	389 detoxified patients with substance use disorder	Connectedness (S)	Afterlife, belief, experience, mysticism	.050^ns^	9/8/3
Unterrainer et al. (2013) [136]	389 detoxified patients with substance use disorder	General Religiosity (RS)	Emotion, God concept, God relationship	.230^ns^	9/8/3
Uren and Wastell (2002) [137]	109 females who experienced perinatal bereavement	Spiritual Orientation Scale (NA)	NA	-.010^ns^	6/6/1
Van der Colff and Rothmann (2009) [138]	818 registered nurses in private and public hospitals	Turning to Religion (R)	Coping, effort, frequency, God relationship, trust	.110*	7/5/3
Verouli et al. (2016) [139]	94 employees in the Primary Health Care	Turning to Religion, shortened (RS/SR)	Coping, frequency, individual practice	.081^ns^	6/5/1
Vosloo et al. (2009) [140]	508 black and white undergraduate students	Religious Well-Being (R)	Belief, coping, God concept, God relationship, meaning	.300**	6/7/1
Wenzl et al. (2021) [141]	1,011 Swedish students	General Religiosity (RS)	Emotion, God concept, God relationship	.060^ns^	8/8/2
Wenzl et al. (2021) [141]	1,011 Swedish students	Connectedness (S)	Afterlife, belief, experience, mysticism	.000^ns^	8/8/2
Wilkins et al. (2012) [142]	585 adults	Prayer Fulfillment (SR)	Coping, efficacy, emotion, individual practice	.300**	6/8/2
Wilkins et al. (2012) [142]	585 adults	Religious Involvement (R)	Frequency, individual practice, intellect	-.020^ns^	6/8/2
Wilkins et al. (2012) [142]	585 adults	Universality (S)	Emotion, universality	.160^ns^	6/8/2
Wissing et al. (2008) [143]	384 white students and adults and 130 black students and adults	Religious Well-Being (R)	Belief, coping, God concept, God relationship, meaning	.247*	3/5/1
Zafar et al. (2019) [144]	332 students from Public sector universities	Daily Spiritual Experiences (RS)	Emotion, experience, frequency, God relationship	.300**	5/6/1
Zamanian et al. (2021) [145]	221 outpatient women with breast cancer	Turning to Religion, shortened (RS/SR)	Coping, frequency, individual practice	.120^ns^	7/3/4
Zarzycka and Rydz (2014a) [146]	636 Polish adults	Orthodoxy (R)	Belief, compliance	.030	6/8/3
Zarzycka and Rydz (2014a) [146]	636 Polish adults	External Critique (N-RS)	Negative aspects	-.264	6/8/3
Zarzycka and Rydz (2014a) [146]	636 Polish adults	Relativism (R)	Intellect, plurality	-.173	6/8/3
Zarzycka and Rydz (2014a) [146]	636 Polish adults	Second Naivité (R)	God relationship, intellect, quest	.070	6/8/3
Zarzycka and Rydz (2014b) [147]	636 Polish adults	Centrality of Religiosity Scale (RS)	Centrality, frequency, individual practice	.166	7/8/5
Zarzycka and Rydz (2014b) [147]	636 Polish adults	Intellect (R)	Frequency, intellect	.146	7/8/5
Zarzycka and Rydz (2014b) [147]	636 Polish adults	Ideology (RS/SR)	Belief	.144	7/8/5
Zarzycka and Rydz (2014b) [147]	636 Polish adults	Public Practice (R)	Centrality, churchiness, collective practice, organized religion	.132	7/8/5
Zarzycka and Rydz (2014b) [147]	636 Polish adults	Private Practice (RS)	Frequency, individual practice	.128	7/8/5
Zarzycka and Rydz (2014b) [147]	636 Polish adults	Experience (RS)	Emotion, experience, frequency, God concept, God relationship	.163	7/8/5
Zehnder Grob (2015) [148]	750 Swiss youths	Centrality of Religiosity Scale (RS)	Centrality, frequency	-.064^ns^	10/8/6
Zehnder Grob (2015) [148]	750 Swiss youths	Everyday Relevance of Religion (R)	Centrality, compliance, ethics, way of life	-.085^ns^	10/8/6
Zehnder Grob (2015) [148]	750 Swiss youths	God concepts (R)	God concept	.016^ns^	10/8/6
Zehnder Grob (2015) [148]	750 Swiss youths	Religious Coping (RS)	Coping, God concept, God relationship, higher being, power	-.081*	10/8/6
Zehnder Grob (2015) [148]	750 Swiss youths	Religious Identity (R)	Affiliation, churchiness, identity, organized religion	.002^ns^	10/8/6
Zehnder Grob (2015) [148]	750 Swiss youths	Religious Pluralism (R)	Compliance, ethics, plurality	-.050^ns^	10/8/6
Zehnder Grob (2015) [148]	750 Swiss youths	Religious Socialization (R)	Family resource, frequency, socialization	.035^ns^	10/8/6
Zerach (2013) [149]	221 child-care workers	Daily Spiritual Experiences (RS)	Emotion, experience, frequency, God relationship	.170	8/8/2
Zerach and Levin (2018) [150]	192 Jewish male volunteers	Spiritual Connection (S)	Self-transcendence, workplace	.170	9/7/5

The correlation coefficient *r* refers to the association between the R/S measurement instrument and the total score of the respective sense of coherence questionnaire (SOC-29 or SOC-13) used in the study. *ns* = not statistically significant

* *p* < .05

** *p* < .01

*** *p* < .001; if no * or no ns is indicated, the significance level was not reported in the study.

### Results of the critical appraisal process

Studies that met the inclusion criteria and did not fulfill any of the first three exclusion criteria were reviewed using our critical appraisal tool (S1 Tool) to determine how well they fit the focus of this meta-analysis. Seven studies [151–157] were excluded according to our fourth exclusion criterion because of "poor fit". Nevertheless, the data of these studies was entered in the overall Excel sheet (S5 Table) so that they could be considered in later analyses. The average total appraisal score of the 89 included studies was 15.13 points (range 9–24). Individual appraisal scores can be found in Table 1.

### Results of the classification of the religion/spirituality measures

Studies that did not use at least one item to measure R/S were excluded from the meta-analysis (criterion I4). In a further step, the question was whether the R/S questionnaires used in the studies actually measured the variable R/S according to our heuristic definition of R/S (criterion E5), namely as communication with reference to something or someone transcendent (Fig 1). Using the coding aid (S4 Table), each individual questionnaire item was analyzed and given a label of R, RS, RS/SR or SR/RS, SR, S, or X (no reference to transcendence). More than a third (> 67%) of the items had to have a reference to transcendence for us to speak of an R/S measure. A total of 1,318 questionnaire items from 128 different potential R/S (sub-) scales were subjected to qualitative content analysis. The fact that 17 total scales, along with some of their sub-scales, were not considered as R/S measures according to our definition/coding (a detailed overview and rationale for the exclusion process can be found in S9 Table) resulted in 22 studies being excluded from the meta-analysis.

In the 89 studies that finally remained, 111 different positive R/S (sub-) scales were used with a total of 925 codable items. Of the 925 items coded, 45.2% were coded with R (418 items), 23.2% with S (215 items), 15.8% with RS (146 items), 7.4% with SR (68 items), 5.0% with X (46 items), and 3.5% with RS/SR (32 items). Based on the item codings, the scales were classified as a whole: Of the 111 (sub-) scales included, 47.7% were classified as R measures (53 scales), 22.5% as RS measures (25 scales), 17.1% as S measures (19 scales), 6.3% as SR measures (7 scales), 3.6% as RS/SR or SR/RS measures (4 scales), and 3 scales (2.7%) could not be classified. Eleven (sub-) scales were classified as negative R/S measures. Detailed documentation of the item-by-item analysis and classification of all those (sub-) scales that have been used in the included studies can be found in S10 Table.

### Dominant aspects of the religion/spirituality measures

Each questionnaire item was not only assigned to a label (R, RS, RS/SR or SR/RS, SR, S, X), but was furthermore examined to determine which aspects of R/S it emphasized. By analyzing the 925 items, a scheme of 61 codes and nine superordinate categories was gradually developed (S11 Table). Over time, each code that emerged from the item-by-item analysis could be provided with a code description, trigger words, and an anchor example. The codes are at an intermediate level of abstraction and each designates an aspect of R/S (e.g., belief, individual practice or God relationship). A formula was then used to decide which R/S aspects of the (sub-)scale stood out as dominant. Which of the included R/S measures emphasize which R/S aspects can be seen in both Tables 1 and S10.

To give an example: The *Intellect* sub-scale of the *Centrality of Religiosity Scale* [158] consists of three items. Each item was individually analyzed using our coding scheme. The result was the following: Centrality (1), **frequency** (2), individual practice (1), **intellect** (3). This means that all items measure the intellectual aspect of R/S (which, by the way, speaks for the conceptually clean construction of the sub-scale *Intellect*), two items refer to the frequency of religious/spiritual activities, and one item asks for the centrality of R/S and the individual R/S practice, respectively. The codes in bold indicate that they are R/S aspects that are dominantly represented in this sub-scale.

### Sense of coherence

Of the 89 included studies, 47 studies (52.8%) used the short version of Antonovsky’s SOC questionnaire (SOC-13), 38 studies (42.7%) used the long version (SOC-29), and four studies (4.5%) used only a SOC sub-scale or have not indicated which SOC-questionnaire version has been used. Across the 85 studies which used one of the two complete questionnaires, the overall SOC score was reported or could be calculated in 64 cases. In these studies, the total SOC score averaged *M* = 4.40 on a seven-point Likert scale. The highest average total SOC score measured was 6.02 points in a sample of 192 Jewish male volunteers [150]. The lowest total SOC score was 3.78 points in a group of 1,243 Romanian female medical students [81]. Based on the mean and *SD* of SOC scores, a three-level categorization was performed. The following tripartite was used: 1.00 to 4.25 points = weak SOC; 4.26 to 5.14 points = moderate SOC; 5.15 to 7.00 points = strong SOC. The majority of the studies (38) measured a moderate SOC, 14 studies measured a weak SOC and 11 studies measured a strong SOC. For the remainder of the studies (26), classification could not be made due to lack of information.

### Correlations between religion/spirituality and sense of coherence

#### Overall and subgroup meta-analyses

Based on our coding matrix (S5 Table), subgroup meta-analyses were conducted using a random effects model. The adjusted effect size between SOC and all positive R/S measures was *r+* = .120, 95% CI [.092, .149]. In our research context, particularly significant (*r+* < -.180 or > .180) were correlations between SOC and negative R/S scales (*r+* = -.405, 95% CI [-.476, -.333]), R/S instruments measuring primarily positive emotions (*r+* = .212, 95% CI [.170, .253]) or meaning-making (adjusted *r+* = .196, 95% CI [.126, .265]). Both sample characteristics (age, culture, gender, health status, religious affiliation) and study characteristics (e.g., publication year) had a moderating effect on the R/S-SOC connection. The correlation was particularly high in eight studies from Southern Asia (adjusted *r+* = .226, 95% CI [.156, .297]), the African Islamic cultural value zone (adjusted *r+* = .196, 95% CI [.106, .285]), and in a small subgroup of six Iranian studies (adjusted *r+* = .194, 95% CI [.117, .271]). The entire results of the subgroup analyses and the heterogeneity tests are summarized in Table 2.

**Table 2:** Subgroup meta-analyses and heterogeneity tests.

Variable	Quantity			Cumulative effect size			Heterogeneity				
	*k*	*ESs*	*n* ^*+*^	*r*^*+*^ *(SE)*	*p*	*95% CI*	*T*^*2*^ *(SE)*	*I* ^*2*^	*H* ^*2*^	*Q*	*p*
**R/S measures and SOC**											
Negative R/S measures	10	11	7,331	-.405 (.037)	< .001	[-.476, -.333]	.010 (.006)	85.050	6.690	54.506	< .001
All positive R/S measures	82	134	62,320	.128 (.017)	< .001	[.095, .160]	.017 (.003)	91.890	12.320	1090.853	< .001
All positive R/S measures; w/o Ghazinour et al. (2004) [94]	81	133	62,220	.120 (.015)	< .001	[.092, .149]	.012 (.003)	89.040	9.120	1027.724	< .001
R measures	35	52	33,422	.094 (.022)	< .001	[.051, .138]	.012 (.004)	87.930	8.280	250.674	< .001
Mixed measures (RS, RS/SR, SR/RS, SR)	43	53	28,032	.139 (.027)	< .001	[.087, .191]	.025 (.007)	93.390	15.130	513.695	< .001
Mixed measures (RS, RS/SR, SR/RS, SR); w/o Ghazinour et al. (2004) [94]	42	52	27,932	.124 (.021)	< .001	[.082, .165]	.014 (.004)	89.000	9.090	460.609	< .001
S measures	24	27	10,565	.138 (.026)	< .001	[.088, .189]	.012 (.005)	83.540	6.080	192.079	< .001
**R/S measures and SOC dimensions**											
Positive R/S and SOC-Comprehensibility	23	46	5,624	.082 (.048)	.088	[-.012, .177]	.043 (.016)	91.000	11.110	194.058	< .001
Positive R/S and SOC-Comprehensibility; w/o Ghazinour et al. (2004) [94]	22	45	5,524	.043 (.032)	.180	[-.020, .107]	.015 (.007)	78.010	4.550	114.079	< .001
Positive R/S and SOC-Manageability	23	46	5,624	.115 (.043)	.007	[.032, .199]	.032 (.012)	88.350	8.580	120.533	< .001
Positive R/S and SOC-Manageability; w/o Ghazinour et al. (2004) [94]	22	45	5,524	.071 (.023)	.002	[.026, .116]	.005 (.003)	54.790	2.210	42.913	.002
Positive R/S and SOC-Meaningfulness	26	49	6,416	.178 (.037)	< .001	[.106, .251]	.027 (.010)	86.690	7.510	124.492	< .001
Positive R/S and SOC-Meaningfulness; w/o Ghazinour et al. (2004) [94]	25	48	6,316	.144 (.022)	< .001	[.101, .187]	.005 (.003)	56.910	2.320	54.715	< .001
**Religious affiliation**											
Heterogenous	6	10	25,154	.071 (.043)	.095	[-.012, .155]	.009 (.007)	92.940	14.170	66.346	< .001
Muslim & mostly Muslim	7	12	1,650	.243 (.118)	.039	[.012, .474]	.091 (.056)	95.100	20.400	92.264	< .001
Muslim & mostly Muslim; w/o Ghazinour et al. (2004) [94]	6	11	1,550	.131 (.056)	.020	[.020, .241]	.014 (.012)	76.430	4.240	28.653	< .001
Christian & mostly Christian	23	49	19,960	.152 (.031)	< .001	[.092, .212]	.017 (.006)	92.510	13.350	245.475	< .001
Christian & mostly Christian; w/o Hammer et al. (2013) [98]	22	48	19,667	.136 (.027)	< .001	[.084, .189]	.012 (.005)	89.690	9.700	223.153	< .001
**Dominant aspects of R/S**											
Afterlife	10	10	11,659	.036 (.049)	.462	[-.060, .132]	.020 (.011)	91.740	12.110	249.897	< .001
Intellect	9	11	4,618	.062 (.032)	.051	[-.000; .124]	.005 (.004)	69.920	3.320	30.107	< .001
Individual Practice	27	37	10,202	.063 (.025)	.010	[.015, .112]	.011 (.004)	80.190	5.050	112.305	< .001
Mysticism	7	7	10.987	.064 (.061)	.291	[-.055, .183]	.021 (.015)	93.610	15.650	202.418	< .001
Organized R/S	11	11	5,221	.079 (.053)	.137	[-.025, .182]	.025 (.014)	90.870	10.950	75.503	< .001
Effort	11	11	3,618	.080 (.039)	.041	[.003, .157]	.012 (.007)	79.260	4.820	53.704	< .001
Development	8	10	1,410	.091 (.059)	.123	[-.025, .206]	.021 (.015)	78.080	4.560	31.246	< .001
Coping	35	36	21,423	.114 (.032)	< .001	[.050, .177]	.031 (.009)	93.970	16.590	399.751	< .001
Coping; w/o Ghazinour et al. (2004) [94]	34	35	21,323	.093 (.025)	< .001	[.044, .142]	.016 (.005)	89.010	9.100	340.836	< .001
Frequency	34	40	17,900	.122 (.023)	< .001	[.077, .168]	.013 (.004)	86.430	7.370	313.858	< .001
Belief	27	28	16,754	.153 (.044)	< .001	[.068, .239]	.043 (.014)	95.310	21.310	391.920	< .001
Belief; w/o Ghazinour et al. (2004) [94]	26	27	16,654	.123 (.034)	< .001	[.057, .189]	.022 (.008)	91.630	11.950	345.853	< .001
Churchiness	10	13	4,854	.135 (.040)	< .001	[.057, .213]	.010 (.007)	79.970	4.990	39.858	< .001
Collective Practice	8	8	3,801	.137 (.048)	.004	[.044, .230]	.012 (.009)	79.770	4.940	24.227	.001
Centrality	22	26	7,826	.137 (.027)	< .001	[.083, .191]	.011 (.005)	78.510	4.650	93.975	< .001
Way of life	15	16	4,502	.141 (.038)	< .001	[.066, .216]	.017 (.008)	82.890	5.850	97.125	< .001
Experience	17	17	17,811	.143 (.035)	< .001	[.075, .212]	.017 (.007)	92.980	14.250	264.260	< .001
God relationship	30	32	23,144	.147 (.023)	< .001	[.101, .193]	.012 (.004)	89.120	9.190	275.210	< .001
God concept	22	25	20,672	.155 (.024)	< .001	[.107, .203]	.010 (.004)	88.030	8.360	201.561	< .001
Trust	11	12	12,765	.162 (.035)	< .001	[.094, .231]	.010 (.006)	86.820	7.590	54.848	< .001
Meaning	12	16	4,406	.242 (.064)	< .001	[.117, .367]	.044 (.021)	93.460	15.290	101.351	< .001
Meaning; w/o Ghazinour et al. (2004) [94]	11	15	4,306	.196 (.036)	< .001	[.126, .265]	.010 (.006)	77.260	4.400	59.144	< .001
Emotion	21	25	12,173	.212 (.021)	< .001	[.170, .253]	.006 (.003)	74.530	3.930	129.958	< .001
**Cultural value zone**											
Protestant Europe	16	28	20,161	.066 (.034)	.053	[-.001, .133]	.015 (.007)	93.840	16.220	352.584	< .001
English speaking	24	43	27,065	.133 (.029)	< .001	[.077, .190]	.013 (.006)	86.020	7.150	271.657	< .001
West & South Asia	11	12	3,120	.141 (.042)	< .001	[.058, .224]	.014 (.009)	78.830	4.720	43.969	< .001
Catholic Europe	16	32	5,954	.151 (.023)	< .001	[.105, .197]	.005 (.003)	65.370	2.890	42.642	< .001
African Islamic	10	15	1,777	.262 (.084)	.002	[.098, .425]	.063 (.033)	91.430	11.670	71.116	< .001
African Islamic; w/o Ghazinour et al. (2004) [94]	9	14	1,677	.196 (.046)	< .001	[.106, .285]	.013 (.009)	68.960	3.220	23.727	.003
**Geographic zone**											
Northern Europe	9	11	2,191	-.014 (.034)	.673	[-.081, .052]	.004 (.005)	45.780	1.840	14.865	.062
Southern Europe	5	7	995	.075 (.043)	.084	[-.010, .160]	.003 (.007)	35.500	1.550	5.709	.222
Western Europe	15	32	20,268	.120 (.031)	< .001	[.058, .181]	.012 (.006)	93.160	14.620	299.375	< .001
Northern America	20	38	26,520	.163 (.029)	< .001	[.107, .219]	.010 (.005)	84.120	6.300	260.698	< .001
Northern America; w/o Hammer et al. (2013) [98]	19	37	24,990	.139 (.024)	< .001	[.093, .186]	.005 (.003)	67.780	3.100	83.194	< .001
Eastern Europe	6	15	4,246	.163 (.045)	< .001	[.075, .251]	.010 (.008)	87.060	7.730	50.328	< .001
Southern Africa	8	9	2,514	.167 (.046)	< .001	[.077, .257]	.011 (.009)	75.750	4.120	27.746	< .001
Southern Asia	9	14	1,686	.301 (.081)	< .001	[.142, .460]	.052 (.030)	90.360	10.380	58.006	< .001
Southern Asia; w/o Ghazinour et al. (2004) [94]	8	13	1,586	.226 (.036)	< .001	[.156, .297]	.005 (.005)	47.070	1.890	13.221	.067
**Country**											
Sweden	5	6	1,401	-.013 (.057)	.815	[-.125, .099]	.009 (.012)	57.890	2.370	8.792	.067
German speaking	14	29	20,061	.109 (.031)	< .001	[.047, .170]	.012 (.005)	93.130	14.560	297.781	< .001
Germany	7	11	17,365	.122 (.054)	.024	[.016, .229]	.019 (.012)	97.440	39.130	232.575	< .001
Austria	5	10	1,843	.124 (.023)	< .001	[.078, .170]	.000 (.002)	0.000	1.000	2.431	.657
South Africa	8	9	2,555	.171 (.045)	< .001	[.084, .258]	.011 (.008)	75.340	4.060	27.615	< .001
USA	19	32	26,351	.171 (.029)	< .001	[.114, .228]	.010 (.005)	84.030	6.260	260.137	< .001
Iran	7	12	1,254	.295 (.107)	.006	[.086, .505]	.073 (.046)	92.610	13.530	56.538	< .001
Iran; w/o Ghazinour et al. (2004) [94]	6	11	1,154	.194 (.039)	< .001	[.117, .271]	.004 (.006)	40.150	1.670	8.404	.135
**Gender**											
Mixed	53	95	42,584	.129 (.022)	< .001	[.086, .173]	.021 (.005)	92.690	13.670	544.180	< .001
Mixed; w/o Ghazinour et al. (2004) [94]	52	94	42,484	.118 (.018)	< .001	[.082, .153]	.012 (.003)	88.440	8.650	474.260	< .001
Mixed; w/o Ghazinour et al. (2004) [94], Hammer et al. (2013) [98]	51	93	40,954	.111 (.017)	< .001	[.078, .144]	.010 (.003)	85.610	6.950	318.904	< .001
Women & Mostly women	26	43	8,956	.150 (.029)	< .001	[.093, .206]	.015 (.006)	83.390	6.020	132.660	< .001
Women & Mostly women; w/o Hammer et al. (2013) [98]	25	42	8,663	.128 (.024)	< .001	[.081, .176]	.008 (.004)	73.850	3.820	87.447	< .001
Men & Mostly men	16	28	16,479	.162 (.039)	< .001	[.085, .239]	.019 (.009)	93.270	14.860	143.138	< .001
**Age group**											
18–30 years	23	36	34,397	.095 (.031)	.002	[.034, .157]	.019 (.007)	94.910	19.640	248.400	< .001
18–30 years; w/o Hammer et al. (2013) [98]	22	35	34,104	.077 (.026)	.003	[.026, .127]	.011 (.004)	91.990	12.480	186.184	< .001
31–60 years	48	76	17,101	.163 (.023)	< .001	[.118, .209]	.019 (.005)	86.800	7.580	319.658	< .001
31–60 years; w/o Ghazinour et al. (2004) [94]	47	75	17,001	.150 (.019)	< .001	[.114, .186]	.010 (.003)	78.050	4.560	268.641	< .001
> 60 years	9	17	1,396	.056 (.051)	.272	[-.044, .157]	.014 (.011)	70.000	3.330	21.671	.003
**Health status**											
Non-clinical samples	69	115	60,545	.132 (.018)	< .001	[.097, .168]	.018 (.004)	93.060	14.400	1060.941	< .001
Non-clinical samples; w/o Ghazinour et al. (2004) [94]	68	114	60,445	.123 (.016)	< .001	[.093, .154]	.012 (.003)	90.330	10.340	997.935	< .001
Non-clinical samples; w/o Ghazinour et al. (2004) [94], Hammer et al. (2013) [98]	67	113	58,915	.118 (.015)	< .001	[.089, .148]	.011 (.003)	88.970	9.060	883.916	< .001
Clinical samples	15	21	2,530	.112 (.038)	.003	[.038, .186]	.013 (.008)	67.350	3.060	38.858	< .001
**Time period**											
1988–1999	7	16	1,010	.070 (.032)	.027	[.008, .133]	.000 (.004)	.000	1.000	4.153	.0656
2000–2011	33	45	10,331	.130 (.033)	< .001	[.065, .194]	.029 (.009)	89.820	9.820	196.656	< .001
2000–2011; w/o Ghazinour et al. (2004) [94]	32	44	10,231	.110 (.024)	< .001	[.063, .156]	.013 (.005)	79.550	4.890	134.730	< .001
2012–2023	43	71	51,493	.137 (.020)	< .001	[.097, .177]	.013 (.004)	92.920	14.120	894.687	< .001

Subgroup analysis was performed only when at least *k* = 5 studies were available. *k* = number of studies; ESs = number of effect sizes; *n*+ = total number of study participants; *r*+ = cumulative effect size; *p* = CI = confidence interval; R/S = religion/spirituality; R = religion; RS = religion, but also spirituality; SR = spirituality, but also religion; S = spirituality; w/o = without; SOC = sense of coherence.

#### Heterogeneity

Heterogeneity in the subgroups was generally high, but varied widely in some cases (Table 2). Taking *I*^*2*^, for instance, 50 of 61 subgroups (not adjusted for outliers) show a heterogeneity score higher than 75%. Seven of 61 subgroups show substantial heterogeneity (50% ≤ *I*^*2*^ ≤ 75%), while only two subgroups each show moderate (25% ≤ *I*^*2*^ ≤ 50%) or low heterogeneity (*I*^*2*^ ≤ 25%).

#### Detection of outliers

Baujat plots were produced to identify influential outliers in a subgroup that biased both heterogeneity and the cumulative ES. Based on Baujat diagrams, it was decided that individual studies would be excluded from a subgroup and a new meta-analysis without outliers would be calculated. Recurrent outliers include the studies by Ghazinour et al. [94] and Hammer et al. [98]. The first study is characterized by a very high positive ES (*r* = .730), and the second study is characterized by a moderate to high ES (*r* = .373) and a relatively large sample (*n* = 1,530).

The subgroup analysis of all positive R/S measures can serve as an example. Based on a Baujat plot (Fig A in S2 Fig), the study by Ghazinour and colleagues was detected as an outlier and a second subgroup analysis was performed without this study. A comparison of the two subgroup analyses shows that the cumulative ES decreases from *r+* = .128 (95% CI [.095, .160]) to .120 (95% CI [.092, .149]). At the same time, the very high heterogeneity (*I*^*2*^ = 91.89%) in the subgroup with Ghazinour et al. only slightly decreases in the subgroup adjusted for the Ghazinour study (*I*^*2*^ = 89.04%). However, after excluding Ghazinour et al., another study shows up in the upper right corner, which no longer extremely skews the overall result, but still has a relatively strong effect on the pooled ES (Fig B in S2 Fig). If we also exclude this study (Hammer et al.), the overall field moves closer together, but the basic picture of the Baujat plot no longer changes significantly (Fig C in S2 Fig). For this reason, we have chosen to consider the calculation of the subset of all positive R/S measures without Ghazinour et al. (but with Hammer et al.) as the authoritative variant.

**Fig 3:**
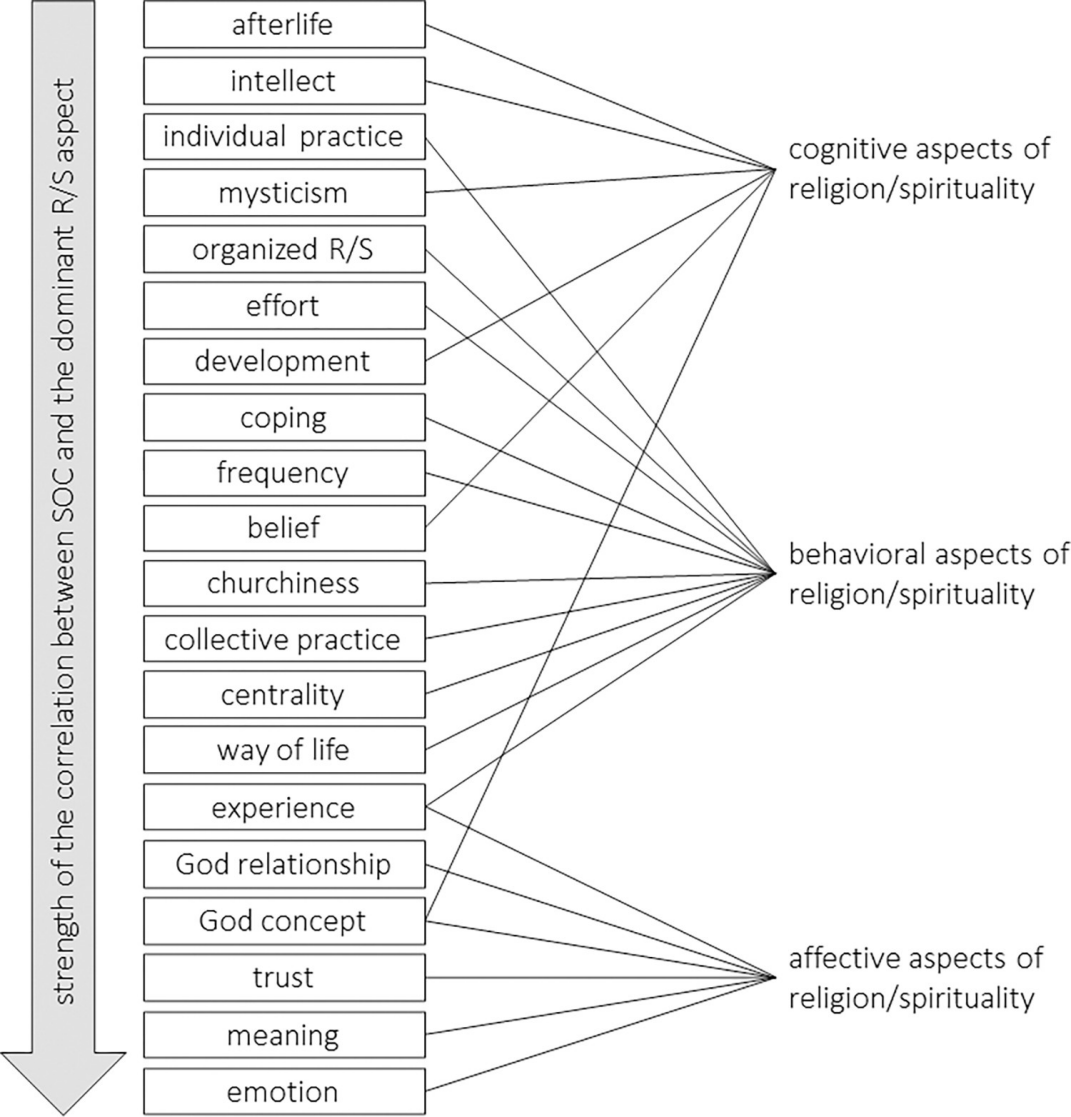
Forest plot for the subgroup of all positive R/S measures.

The decision is further supported by the forest plot (Fig 3), which makes Ghazinour and colleagues’ study visible as a clear outlier, while Hammer’s and colleagues’ study—although further to the right of the overall effect estimate than most other studies—does not show major discrepancies. Furthermore, the long confidence intervals of the studies by Edwards and Besseling and Meghani and colleagues are remarkable, which, however, are not so important because of their rather small point estimates.

In all cases, individual studies (at maximum two in each subgroup) were omitted gradually from the subgroup analyses until no study is visible as an extreme outlier in the upper right corner of the Baujat plot. The changes in values caused by each step of the leaving-out process can be seen in Table 2, so that every decision made is transparent. Despite the exclusion of outliers, the level of heterogeneity has generally remained high.

#### Publication bias

The analysis of the Baujat plots had already given an indication of outlier studies which have a biasing effect on the overall ESs. However, in addition to the detection of single outlier studies, it is important to examine whether there is also a systematic distortion due to publication bias. The linear increase of ESs across time (time period 1988–1999: *r+* = .070; time period 2000–2011: adj. *r+* = .110; time period 2012–2022: adj. *r+* = .137) could hint at a possible publication bias. Therefore, we generated funnel plots for the subgroup of all positive R/S measures (Fig A in S3 Fig) and for the subgroup of all positive R/S measures adjusted for outliers (Fig B and Fig C in S3 Fig). However, tests for funnel plot asymmetry did not give evidence for systematic publication bias.

## Discussion

### Religion/Spirituality and sense of coherence are significantly correlated

Our meta-analysis shows a positive significant relationship between positive R/S measures and SOC. If the outlier study identified by means of Baujat and funnel plot is excluded from the group, the overall ES is *r+* = .120. Comparing the adjusted overall ES in our meta-analysis with results of other systematic reviews in the field of R/S-mental health-connection research, the association seems to be slightly higher (see the introductory paragraph above). The impression is strengthened when taking into account that German-language studies were also included in our meta-analysis, since the association between R/S and psychological health has been shown to be lower in samples from the German-speaking area than, for example, in meta-analyses based primarily on U.S. samples [7].

In addition, it appears that the breadth of our inclusion criteria led to a decrease in the cumulative ES. With slightly stricter criteria (only studies from the year 2000 onward; only studies with a sample size ≤ 100; no measures of extrinsic religious orientation), the result would have been even more significant: In the remaining 65 studies the cumulative ES is *r+* = .139, 95% CI [.103, .175] based on an overall sample size of 60,608. If Ghazinour et al. [94] is omitted from this subgroup as an outlier study, the overall ES is still *r+* = .130, 95% CI [.099, .160], with the calculation based on 64 studies with 60,508 participants. The result supports the timeliness of the SOC hypothesis and confirms the assumption of Garssen and colleagues [4] that SOC is a particularly well-suited indicator of psychological adjustment in the context of R/S-mental health connection research.

The relationship between SOC and positive R/S is also moderated by personal factors (e.g., age, gender, health status). In our analysis, a strong correlation between SOC and R/S was measured in the subgroup of middle-aged people (adj. *r+* = .150), whereas the ES was significantly lower in younger (adj. *r+* = .077) and older people (*r+* = .056). In addition, samples composed exclusively or primarily of men have significantly higher ES than samples composed exclusively or primarily of women (*z* = 2.617, *p* = .004). Finally, in non-clinical samples, the ES turns out to be higher (adj. *r+* = .118) than in clinical samples (*r+* = .112), although the difference is not significant (*z* = .299, *p* = .382). In short, the SOC-R/S association is particularly positive in middle-aged men. Given that in most countries men rate themselves as less religious/spiritual than women [159,160], we attribute our result more to the influence of the SOC variable. At least, some studies suggest that the SOC of men is stronger than the SOC of women [24]. Since Antonovsky [18] emphasized that many salutary experiences of comprehensibility, manageability, and meaningfulness are gained in work contexts and are closely related to social recognition, it can be surmised that the stronger ES between SOC and R/S in the subgroup of “men and mostly men” is related to the still higher socio-economic status of middle-aged men (31–60 years) in many countries and cultures. For example, several studies show that there is a clear relationship between the level of income and the level of SOC [161–163]. In addition, religious power and prestige [164] might play a moderating role in the SOC-R/S association because in most traditional religions (e.g., Buddhism, Catholicism or Islam) men play a prominent role in religious organizations and communities, which might be a source of their SOC.

As was to be expected, there is also a clear significant inverse correlation between negative R/S measures and SOC of *r+* = -.405. Hodapp and Zwingmann [7] argued that such a pronounced negative association is particularly found in German-speaking countries. The fact that three studies with large German-speaking samples (*n+* = 4,960) are part of our subgroup might suggest that this bias partly explains the high negative value. However, the negative ES in our subgroup analysis is twice as high as the ES of *r+* = -.200 in Hodapp and Zwingmann’s meta-analysis of 37 negative correlations from the German-speaking area. The strikingly high correlation, although not very robust due to the small number of ESs in the subgroup (11), can probably be attributed to the high fit between religious samples and R/S measures. Two German studies in our subgroup used the *Spiritual Dryness Scale* [73] in church-active Catholics, and three studies used the *Negative Religious Coping* subscale [165] in two Iranian Muslim samples and a refugee sample. If we take a closer look at the items of the two scales, we notice that both negative R measures focus on a God relationship that is burdened by negative emotions. SOC as an outcome parameter might be particularly sensitive to the loss of transpersonal trust in these religious samples because, by and large, SOC is by definition a measure of a “pervasive, enduring though dynamic feeling of confidence” [18].

### Emotional and meaning aspects of religion/spirituality are most associated with sense of coherence

**Fig 4:**
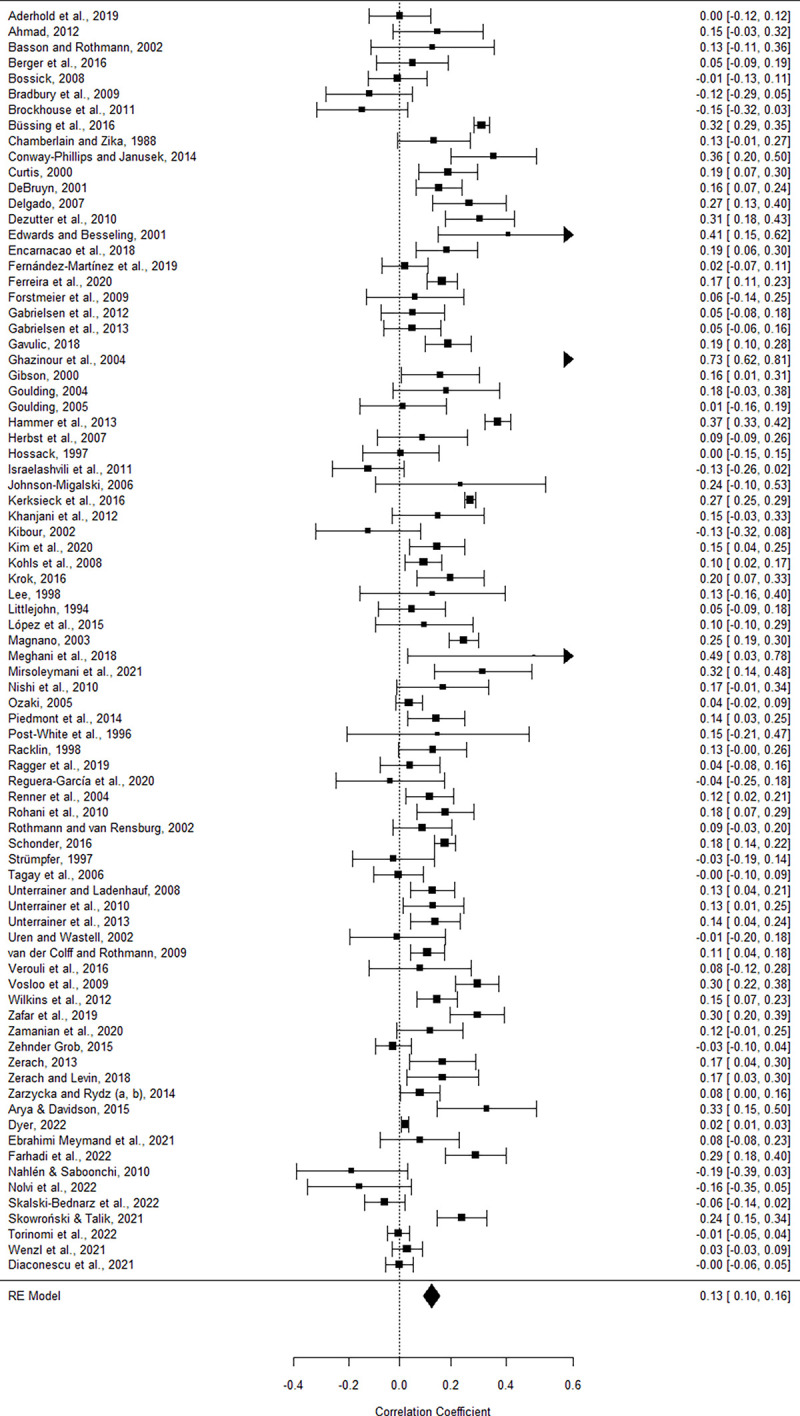
Three-dimensional model of aspects of religion/spirituality.

Salsman and colleagues [166] used a taxonomy of four categories (affective, behavioral, cognitive, other) to classify the R/S measures in their meta-analysis of the R/S and mental health association in cancer patients. They concluded that the affective R/S dimension (with the conceptually problematic *Spiritual Well-Being* scales excluded) is significantly more strongly associated with mental health (z = .290) than the other dimensions: cognitive R/S (z = .100), other R/S (z = .080), and behavioral R/S (z = .030) [167]. If we assign the 20 dominant aspects of R/S that we were able to subject to subgroup meta-analysis (Table 2) to the higher-order categories “affective”, “cognitive”, and “behavioral”, a strikingly consistent picture emerges (Fig 4). In our meta-analysis, the emotional aspect of R/S exhibits the strongest ES (*r+* = .212), whereas the intellectual aspect of R/S is hardly related to SOC (*r+* = .062). Other R/S aspects that have a strong affective component (meaning, trust, God concept, God relationship, experience) also have consistently higher *r*-values than more intellectual aspects of R/S (afterlife, development, belief). In the middle range of the ESs, R/S aspects are found that can largely be categorized as behavioral (e.g., frequency, churchiness, collective practice, centrality, way of life).

That emotion and meaning turned out to be the R/S aspects most associated with SOC becomes particularly plausible when one considers this definition of the SOC component “meaningfulness” inspired by Antonvosky: "Meaningfulness, the motivational dimension, refers to the *extent to which one feels that life has an emotional meaning*, that at least some of the problems faced in life are worth commitment and dedication, and are seen as challenges rather than only as burdens" [168]. It is therefore hardly surprising that the cumulative ES between positive R/S measures and the SOC-subscale *Meaningfulness*, adjusted for outlier studies, is *r+* = .144. This association is strikingly stronger than the non-significant relationship between R/S and the cognitive SOC-subscale *Comprehensibility*, which, when adjusted, is only *r+* = .043. The behavioral SOC component *Manageability* is also much less correlated with positive R/S measures (*r+* = .071), so that it can be assumed that the R/S-SOC connection can be explained primarily by the emotionally stabilizing and meaning-giving function of R/S as a resource of SOC as an “enduring though dynamic feeling of confidence” [18]. This observation echoes the conceptual ideas of German theologian Hans Küng, who defined “faith” as “basic trust in life” (*Lebensvertrauen*) and which he described as the “cornerstone of a healthy personality” [169] and the “foundation of a global ethic” [170].

### Sense of coherence is more correlated with spirituality than religion

As religion and spirituality become more socially and scientifically differentiated, the question arises as to which form of reference to transcendence is more associated with SOC. From a theoretical perspective, Antonovsky viewed the societal processes that accompanied the emergence of spirituality in modernity, namely an increasing de-traditionalization and individualization of faith, rather critically. Because religion, in his view, would rather contribute to group cohesion and worldview congruence, he assumed that institutionalized religion would be a stronger biographical SOC source than individualized spirituality (for a detailed analysis, see [43]).

However, the results of our meta-analysis refute Antonovsky’s hypothesis and show that S measures (*r+* = .138) are significantly stronger (*z* = 3.996, *p* < .001) associated with SOC than R measures (*r+* = .094). As expected, mixed measures, i.e., instruments that include approximately the same number of S as R items, show a correlation strength that lies between the other two subgroups (adj. *r+* = .124). The result can thus be reduced to the formula: The more items an R/S scale includes that measure spirituality (as reference to non-institutionalized and conceptually/semantically open transcendent ascriptions of meaning), the higher the correlation with SOC.

The result is probably due to a complex amalgamation of sociocultural developments, academic trends, and methodological factors. One of the main reasons might be the impact of changing study populations (e.g., the growing group of the non-religious or the “spiritual but not religious” [SBNR]) that respond differently to the (older) R and the (newer) S measures. Measurement instruments are constructed not only with certain questions in mind, but also with certain samples. This (to a greater or lesser extent) time, culture, and sample boundedness of many R/S measures can lead to results being biased. For example, it is likely that participants who would rate themselves as non-religious or SBNR would not score well on questionnaires that we classified as R measures. For instance, the study by Bradbury et al. [46] supports this explanation. The significant negative association between the R measure (*Traditional Religious Beliefs*) and SOC of *r* = -.220 is most likely due to the sample of a primarily non-religious population of undergraduate students in England. Since the medieval-looking four-item sub-scale of the *Revised Paranormal Belief Scale* [171] asks primarily for agreement with metaphysical Christian concepts (soul, devil, God, heaven, hell), it will presumably correlate positively with SOC only in a country or at a time when Christian dogmas are culturally strongly anchored and still have credibility. Thus, a strong positive relationship between religion and SOC might depend on whether and to what extent certain concepts of transcendence are supported by socio-cultural plausibility structures.

### Religion/Spirituality as social value is strongly associated with sense of coherence

The subgroup analysis results compiled in Table 2 under the sub-headings “Country”, “Geographic zone”, “Cultural value zone”, and “Religious affiliation” suggest: If the religious/spiritual worldviews, values and practices of the subgroup under study are supported by the cultural environment, i.e., if social plausibility structures are broadly intact, the R/S-SOC relationship turns out stronger. In agreement with the findings of Hodapp and Zwingmann [7], a transatlantic comparison shows that the R/S-SOC connection was found to be significantly weaker (*z* = -6.751, *p* < .001) in German-speaking samples (*r+* = .109) than in populations from the United States (*r+* = .171). This may be related not only to the different levels of religiosity of people [172], but also to the massive loss of social relevance of traditional Christian religion in German-speaking countries. However, an intra-European comparison shows that studies carried out in the geographic zone Western Europe (to which the German-speaking countries geographically belong) performed better (*r+* = .120) than studies from Southern Europe (*r+* = .075) or Northern Europe (*r+* = -.014).

The country or geographic differences could be attributed to varying cultural value zones. According to The Inglehart-Welzel World Cultural Map [59], most northern European countries belong to the cultural value zone "Protestant Europe", which emphasizes secular-rational and self-expression values and is more critical of religion, so R/S does not have the same plausibility strength as in cultures where R/S still plays a vital role in the societal value system. In many Western countries with Christian background, people of faith face the question of whether to leave or remain in the constituted church, which leads to inner insecurity, religious/spiritual doubts, and an unstable religious identity. While a stable religious affiliation is usually associated with better mental health, those who are undecided suffer more from depressive symptoms, according to an American study [173]. Thus, it is hardly surprising that a strong ES (adj. *r+* = .194) was measured in a small subgroup of six Iranian studies, which belongs geographically to Southern Asia (adj. *r+* = .226) and to the so-called “African Islamic” cultural value zone (adj. *r+* = .196). On the World Cultural Map, the “African Islamic” zone is located in the lower left corner, diametrically opposite to the “Protestant Europe” zone, which means that the countries in this cultural zone emphasize traditional (religious) values. Many countries from this cultural value zone have a binding state religion (e.g., Shiite Islam in Iran or Sunni Islam in Pakistan) and/or the people feel they belong to a relatively stable, political powerful and socially valued majority religion [174]. Sample populations that consist entirely or predominantly of Muslims (adj. *r+* = .131) or Christians (adj. *r+* = .136) have a significantly stronger R/S-SOC relationship than heterogonous samples (*r+* = .071). If this result is not due to a bias caused by a too small subgroup size (e.g., the adjusted Muslim subgroup consists only of 1,550 participants and 11 ESs), it can be surmised that R/S is a stronger SOC source in homogenous religious/spiritual samples.

Overall, our results are thus compatible with the "religiosity as social value hypothesis" (RASV), which states that in religious cultures religiosity is more likely to be a social resource for self-esteem than in secular cultures [175]. By analogy with the RASV hypothesis, we can summarize our assumption as follows: If the culture in which a (religious/spiritual) person lives supports his/her (religious/spiritual) worldview/practice, i.e., if this worldview/practice is culturally embedded, socially plausibilized, and socially valued, then the likelihood that this person will have a strong SOC increases. In technical terms, the variable "cultural value orientation" seems to have a moderating effect on the relationship between R/S and SOC. However, our hypothesis does not make any statement about the causality of the relationship: A strong SOC may lead a person to follow a religion/spirituality that is socio-culturally accepted. Or, conversely, a culturally embedded and socially valued R/S may be a source of a strong SOC. However, since there are many individual pathways to a strong SOC, it must remain open what role R/S plays in a person’s life and whether there are not other, more central socio-cultural and personal SOC sources.

### Strengths

Our meta-analysis focused on a well-established mechanism within R/S-health research, namely the SOC hypothesis, which promised robust results from a theoretical perspective [17].

The multitude of possible combinations between the multidimensional construct R/S and negative/positive indicators for mental health quickly leads to an unmanageable number of options (e.g., spiritual struggle/anxiety, religious affiliation/life satisfaction, religious coping/locus of control, etc.) or to simplifying generalizations (e.g., religion/mental health). However, by selecting SOC as a single, widely used and psychometrically valid indicator of psychological adjustment, we avoided the complexity of one variable (mental health) in order to deal more precisely with the intricacy and richness of aspects of the other variable (R/S).

Perhaps the greatest strength of our meta-analysis is that all items of all R/S measures were subjected to a qualitative content analysis (S10 Table). In this way, it was possible to decide in a transparent and systematic way whether a scale measures religion, spirituality, a mixed form, or (despite a scale name that promises otherwise) neither. At the same time, all included R/S instruments were analyzed item-by-item to determine which aspects of R/S they (actually) measure, which in individual cases may mean that a scale on spiritual well-being turns out to be a measure of the relationship with God. The categorization of R/S scales reduced the likelihood that the meta-analysis will be biased by measurement-specific issues and led to more valid and more fine-grained results.

Publication bias was further reduced by the inclusion of dissertations and the use of a self-developed critical appraisal tool to ensure that inappropriate and methodologically problematic studies (“poor fit”) were excluded from the meta-analysis.

Another advantage of our meta-analysis was that both English- and German-language studies were included. This not only expanded the data material, but also allowed cross-cultural comparisons and the analysis of cultural factors as potential moderators of the R/S-SOC connection.

### Limitations

Since, strictly speaking, the SOC hypothesis conceptualizes SOC as a mediator between R/S and health, the results of the meta-analysis can only be considered as a partial confirmation of the hypothesis. While the SOC hypothesis assumes an indirect causal effect of R/S (predictor) on health (outcome) interlinked by SOC (mediator), the present meta-analysis does not make any statements about causality. Because primary research on the R/S-SOC connection has used almost exclusively correlational study designs, the causal direction of variables remained unclear. R/S may be a biographical source for a strong SOC. At the same time, a strong SOC may be a source for the mobilization of religious/spiritual resources in coping with stressful events. Thus, a beneficial relationship is presumably bi-directional.

While our decision to choose SOC as the sole indicator for the complex construct of mental health necessarily reduces the complexity of the meta-analysis to a manageable level, it also has some disadvantages. Only tentative conclusions can be drawn about the association between R/S and mental health in general, as SOC is only one positive variable that may interact with the phenomenon of R/S in specific ways. And even though SOC is often considered a transcultural construct [176,177], it is conceivable that there are cultures that would not prioritize SOC as a crucial indicator of mental health, which could limit the generalizability of our results.

The broadness of our inclusion criteria led to a high heterogeneity of the studies (in terms of methodology as well as sample and study characteristics), which in turn made it difficult to identify the exact religious/spiritual factors affecting the R/S-health connection. The systematic differentiation of R/S scales according to our heuristic continuum model of R/S (Fig 1) and the inductively developed taxonomy of R/S aspects (S11 Table) did help in a more precise analysis. But this very approach involved preliminary decisions (e.g., the definition of the constructs “religion” and “spirituality”) and many further choices (e.g., the coding of each individual item) that affected the outcome. For this reason, we tried to increase intersubjective comprehensibility through intra- and interrater agreement and explained in detail our methodological procedures, ensuring transparency (S1 Text).

The validity of our results, as with almost any meta-analysis in this line of research, is limited by the fact that both the SOC and the various R/S measures are self-report instruments. Self-assessments are, of course, highly subjective and susceptible to bias due to social desirability [178,179]. With regard to SOC measurement, there is also the fact that extremely high SOC scores and possibly very high R/S-SOC correlations may be indicative of rigid SOC [180]. Antonovsky has also referred to rigid SOC as inauthentic SOC, as it is based on a simplistic, fundamentalist worldview that can be (but need not be) fragile when subjected to a reality check [18].

## Conclusions

As hypothesized by Garssen and colleagues [4], SOC has been confirmed to be a particularly sensitive indicator for psychological adjustment in the context of R/S-health connection research. In line with the coherence hypothesis, the results confirm that R/S and SOC are closely associated and suggest that there are different religious/spiritual pathways to a strong SOC. Which pathway is more salutogenic presumably depends not only on individual differences (e.g., age, gender, health status, level of religiosity/spirituality), but also on the cultural embeddedness, societal value, and social plausibility of R/S in a given context and at a certain time period.

In our meta-analysis, the affective dimension of R/S was found to play a particularly determining role in the strength and direction of the R/S-SOC association: Negative R/S-related emotions were highly inversely correlated with SOC, and positive R/S-related emotions were highly positively correlated with SOC. High positive effect sizes were also found for R/S as a meaning-making resource or as transpersonal trust (trust, God concept, God relationship).

The continuous decrease in measures (only) of religion and simultaneous increase in measures of religion and spirituality is associated with an increase in the effect size of the R/S-SOC link over time. The more items an R/S scale includes that measure spirituality (as reference to non-institutionalized and conceptually/semantically open transcendent ascriptions of meaning), the higher the correlation with SOC. In less methodical and simplified terms: In our meta-analysis, spirituality correlates more strongly with SOC (as an important indicator of mental health) than religion.

Because some measurement instruments were found not to be suitable as measures of R/S or to measure aspects of R/S for which they have not been designated (S9 Table), differentiated and valid results could only be presented on the basis of an item-by-item qualitative content analysis, inductive coding, and classification of all included R/S (sub-) scales (S10 Table). Future R/S-health connection research should investigate specific aspects of R/S using conceptually cleanly constructed and context-sensitive (e.g., culture- and religion-specific) measurement instruments.

## Supporting information

S1 Checklist PRISMA checklist. (DOCX)

S1 Fig PRISMA flow diagram. (TIF)

S2 Fig Baujat plots for the subgroup of all positive R/S measures. (PDF)

S3 Fig Funnel plots for the subgroup of all positive R/S measures. (PDF)

S1 Table Boolean search strategy. (PDF)

S2 Table Search queries. Exact representation of the search algorithms in each of the databases used.
(PDF)

S3 Table Rationale for the inclusion and exclusion criteria. (PDF)

S4 Table Coding aid. Coding aid used to assign questionnaire items to the categories R, RS, RS/SR, SR, S, or X (item without religious/spiritual reference) in an intersubjectively comprehensible manner.
(PDF)

S5 Table Coding matrix. An Excel spreadsheet that includes the extracted data of all included studies according to the parameters we established in the paragraph “Coding of the included studies”.
(XLSX)

S6 Table Example subgroup. An exemplary Excel table containing only the study data of the subgroup “Negative R/S measures” relevant for the meta-analysis with our R code.
(XLSX)

S7 Table Personal communication. Overview of the first author’s personal communication and documentation of the decision whether to include studies after obtaining additional information.
(PDF)

S8 Table List of excluded studies. List of all studies excluded from meta-analysis and indication of the respective exclusion criteria.
(PDF)

S9 Table List of excluded scales. List of all measurement instruments that cannot be considered as measures of R/S according to our (heuristic) definition of R/S including documentation of the item-wise classification process and a rationale for the decision.
(PDF)

S10 Table Characteristics of included scales. List of all scales that can be considered as measures of R/S according to our (heuristic) definition. The table contains information about the item-wise classification of each scale, the dominant R/S aspects, Cronbach’s alpha, and a sample item.
(PDF)

S11 Table Code and category system. The inductive coding system for coding the dominant aspects of R/S captured by the individual questionnaire items. For intersubjective comprehensibility, the table also includes trigger words, code descriptions, and anchor examples. Parallel to the codes, the more abstract deductive category system resulting from the inductive coding of the items is also presented.
(PDF)

S1 Tool Critical appraisal tool. Self-developed critical appraisal tool to check how well a study meets the methodological requirements or how well it fits the context of our research question.
(PDF)

S1 Text Methodological intricacies. A text explaining the methodological subtleties of classifying measures to be considered as measurement instruments for R/S in the context of our meta-analysis.
(PDF)

S1 Code R Code. The code used for the meta-analytical procedures for the RStudio software, which can be copied and used to replicate the calculations we perform.
(PDF)

## References

[1] Oman D, Syme SL. Weighing the evidence: What is revealed by 100+ meta-analyses and systematic reviews of religion/spirituality and health? In: Oman D, editor. Why religion and spirituality matter for public health: Evidence, Implications, and Resources: Springer; 2018. p. 261–81.

[2] Lefevor GT, Davis EB, Paiz JY, Smack ACP. The relationship between religiousness and health among sexual minorities: A meta-analysis. Psychological Bulletin. 2021;147(7):647–66. doi: 10.1037/bul0000321 33793286

[3] Masters KS, Hooker SA. Spirituality/religiosity and health. In: Sweeny K, Robbins ML, Cohen LM, editors. The Wiley Encyclopedia of Health Psychology: John Wiley & Sons; 2020. p. 709–16.

[4] Garssen B, Visser A, Pool G. Does spirituality or religion positively affect mental health? Meta-analysis of longitudinal studies. The International Journal for the Psychology of Religion. 2021;31(1):4–20. doi: 10.1080/10508619.2020.1729570

[5] Bergin AE. Religiosity and mental health: A critical reevaluation and meta-analysis. Professional Psychology: Research and Practice. 1983;14(2):170–84. doi: 10.1037/0735-7028.14.2.170

[6] Hackney CH, Sanders GS. Religiosity and mental health: A meta–analysis of recent studies. Journal for the Scientific Study of Religion. 2003;42(1):43–55. doi: 10.1111/1468-5906.t01-1-00160

[7] Hodapp B, Zwingmann C. Religiosity/spirituality and mental health: a meta-analysis of studies from the German-speaking area. Journal of Religion and Health. 2019;58(6):1970–98. doi: 10.1007/s10943-019-00759-0 30632002

[8] Stulp HP, Koelen J, Schep-Akkerman A, Glas GG, Eurelings-Bontekoe L. God representations and aspects of psychological functioning: A meta-analysis. Cogent Psychology. 2019;6(1):Article 1647926. doi: 10.1080/23311908.2019.1647926

[9] Bockrath MF, Pargament KI, Wong S, Harriott VA, Pomerleau JM, Homolka SJ, et al. Religious and spiritual struggles and their links to psychological adjustment: A meta-analysis of longitudinal studies. Psychology of Religion and Spirituality. 2021:Advance online publication. doi: 10.1037/rel0000400

[10] Oman D, Lukoff D. Mental health, religion, and spirituality. In: Oman D, editor. Why religion and spirituality matter for public health: Evidence, Implications, and Resources: Springer; 2018. p. 225–43.

[11] Abdel-Khalek AM, Nuño L, Gómez-Benito J, Lester D. The relationship between religiosity and anxiety: A meta-analysis. Journal of Religion and Health. 2019;58(5):1847–56. doi: 10.1007/s10943-019-00881-z 31309442

[12] Idler EL. Religious involvement and the health of the elderly: Some hypotheses and an initial test. Social Forces. 1987;66(1):226–38.

[13] Park CL. Making sense of the meaning literature: an integrative review of meaning making and its effects on adjustment to stressful life events. Psychological Bulletin. 2010;136(2):257–301. doi: 10.1037/a0018301 20192563

[14] Park CL. Religion as a meaning‐making framework in coping with life stress. Journal of Social Issues. 2005;61(4):707–29. doi: 10.1111/j.1540-4560.2005.00428.x

[15] Luckmann T. Die unsichtbare Religion = The invisbile religion: Suhrkamp; 1991.

[16] Taves A, Asprem E, Ihm E. Psychology, meaning making, and the study of worldviews: Beyond religion and non-religion. Psychology of Religion and Spirituality. 2018;10(3):207–17. 10.1037/rel0000201.

[17] Jeserich F. The coherence hypothesis: Critical reconsideration, reception history and development of a theoretical model. Archiv für Religionspsychologie. 2014;36(1):1–51. doi: 10.1163/15736121-12341281

[18] Antonovsky A. Unraveling the mystery of health: How people manage stress and stay well: Jossey-Bass; 1987.

[19] George LK, Larson DB, Koenig HG, McCullough ME. Spirituality and health: What we know, what we need to know. Journal of Social and Clinical Psychology. 2000;19(1):102–16. doi: 10.1521/jscp.2000.19.1.102

[20] Bleidorn W, Lenhausen MR, Schwaba T, Gebauer JE, Hopwood CJ. Secularization trends obscure developmental changes in religiosity. Social Psychological and Personality Science. 2022:1–10. doi: 10.1177/19485506221076684

[21] Koelen M, Eriksson M, Cattan M. Older people, sense of coherence and community. In: Mittelmark MB, Bauer GF, Vaandrager L, Pelikan JM, Sagy S, Eriksson M, et al., editors. The handbook of salutogenesis: Springer; 2017. p. 137–49.

[22] Nilsson KW, Leppert J, Simonsson B, Starrin B. Sense of coherence and psychological well-being: Improvement with age. Journal of Epidemiology & Community Health. 2010;64(4):347–52. doi: 10.1136/jech.2008.081174 19692734

[23] Braun-Lewensohn O, Idan O, Lindström B, Margalit M. Salutogenesis: Sense of coherence in adolescence. In: Mittelmark MB, Bauer GF, Vaandrager L, Pelikan JM, Sagy S, Eriksson M, et al., editors. The handbook of salutogenesis: Springer; 2017. p. 123–36. 28590649

[24] Larsson G, Kallenberg KO. Sense of coherence, socioeconomic conditions and health: Interrelationships in a nation-wide Swedish sample. The European Journal of Public Health. 1996;6(3):175–80. doi: 10.1093/eurpub/6.3.175

[25] Garcia-Moya I, Rivera F, Moreno C, Lindström B, Jimenez-Iglesias A. Analysis of the importance of family in the development of sense of coherence during adolescence. Scandinavian Journal of Public Health. 2012;40(4):333–9. doi: 10.1177/1403494812449924 22786917

[26] Roothman B, Kirsten DK, Wissing MP. Gender differences in aspects of psychological well-being. South African Journal of Psychology. 2003;33(4):212–8.

[27] Volanen S-M, Lahelma E, Silventoinen K, Suominen S. Factors contributing to sense of coherence among men and women. The European Journal of Public Health. 2004;14(3):322–30. doi: 10.1093/eurpub/14.3.322 15369042

[28] Klein C, Keller B, Traunmüller R. Sind Frauen tatsächlich grundsätzlich religiöser als Männer? Internationale und interreligiöse Befunde auf Basis des Religionsmonitors 2008 = Are women really more religious than men? International and interreligious findings based on the Religion Monitor 2008. In: Sammet K, Benthaus-Apel F, Gärtner C, editors. Religion und Geschlechterordnungen = Religion and gender orders: Springer; 2017. p. 99–131.

[29] Vardy T, Moya C, Placek CD, Apicella CL, Bolyanatz A, Cohen E, et al. The religiosity gender gap in 14 diverse societies. Religion, Brain & Behavior. 2022;12(1–2):18–37. doi: 10.1080/2153599X.2021.2006292

[30] Ano GG, Vasconcelles EB. Religious coping and psychological adjustment to stress: A meta‐analysis. Journal of Cinical Psychology. 2005;61(4):461–80. doi: 10.1002/jclp.20049 15503316

[31] Benz C, Bull T, Mittelmark M, Vaandrager L. Culture in salutogenesis: The scholarship of Aaron Antonovsky. Global Health Promotion. 2014;21(4):16–23. doi: 10.1177/1757975914528550 24814861 PMC4242901

[32] Braun-Lewensohn O, Sagy S. Salutogenesis and culture: Personal and community sense of coherence among adolescents belonging to three different cultural groups. International Review of Psychiatry. 2011;23(6):533–41. doi: 10.3109/09540261.2011.637905 22272591

[33] Feldt T, Lintula H, Suominen S, Koskenvuo M, Vahtera J, Kivimäki M. Structural validity and temporal stability of the 13-item sense of coherence scale: Prospective evidence from the population-based HeSSup study. Quality of Life Research. 2007;16(3):483–93. doi: 10.1007/s11136-006-9130-z 17091360

[34] Smith PM, Breslin FC, Beaton DE. Questioning the stability of sense of coherence. Social Psychiatry and Psychiatric Epidemiology. 2003;38(9):475–84. 14504729 10.1007/s00127-003-0654-z

[35] Schnyder U, Büchi S, Sensky T, Klaghofer R. Antonovsky’s sense of coherence: Trait or state? Psychotherapy and Psychosomatics. 2000;69(6):296–302. doi: 10.1159/000012411 11070441

[36] Antonovsky A. The structure and properties of the sense of coherence scale. Social Science & Medicine. 1993;36(6):725–33. doi: 10.1016/0277-9536(93)90033-Z 8480217

[37] Frenz AW, Carey MP, Jorgensen RS. Psychometric evaluation of Antonovsky’s sense of coherence scale. Psychological Assessment. 1993;5(2):145–53. doi: 10.1037/1040-3590.5.2.145

[38] Gana K, Garnier S. Latent structure of the sense of coherence scale in a French sample. Personality and Individual Differences. 2001;31(7):1079–90. doi: 10.1016/S0191-8869(00)00205-1

[39] Eriksson M, Lindström B. Validity of Antonovsky’s sense of coherence scale: A systematic review. Journal of Epidemiology & Community Health. 2005;59(6):460–6. doi: 10.1136/jech.2003.018085 15911640 PMC1757043

[40] Geyer S. Some conceptual considerations on the sense of coherence. Social Science & Medicine. 1997;44(12):1771–9. doi: 10.1016/s0277-9536(96)00286-9 9194239

[41] Eriksson M, Lindström B. Antonovsky’s sense of coherence scale and the relation with health: a systematic review. Journal of Epidemiology & Community Health. 2006;60(5):376–81. doi: 10.1136/jech.2005.041616 16614325 PMC2563977

[42] Garssen B, Visser A, de Jager Meezenbroek E. Examining whether spirituality predicts subjective well-being: How to avoid tautology. Psychology of Religion and Spirituality. 2016;8(2):141–8.

[43] Jeserich F. Antonovskys Religionsverständnis und dessen Verhältnis zu Konzepten von Spiritualität-eine gesundheitswissenschaftliche und sozialethische Herausforderung = Antonovsky’s understanding of religion and its relationship to concepts of spirituality: A challenge in health science and social ethics. In: Hensen P, Kölzer C, editors. Die gesunde Gesellschaft: Sozioökonomische Perspektiven und sozialethische Herausforderungen = The healthy society: Socioeconomic perspectives and socioethical challenges: Springer; 2011. p. 181–205.

[44] Jeserich F. Kohärenzgefühl und Dimensionen des Vertrauens = Sense of coherence and dimensions of trust. In: Petzold TD, editor. Vertrauensbuch zur Salutogenese = Book on trust and salutogenesis. Bad Gandersheim: Verlag Gesunde Entwicklung; 2012. p. 209–44.

[45] Piedmont RL, Magyar-Russell G, DiLella N, Matter S. Sense of coherence: Big five correlates, spirituality, and incremental validity. Current Issues in Personality Psychology. 2014;2(1):1–9. doi: 10.5114/cipp.2014.43096

[46] Bradbury DA, Stirling J, Cavill J, Parker A. Psychosis-like experiences in the general population: An exploratory factor analysis. Personality and Individual Differences. 2009;46(7):729–34. doi: 10.1016/j.paid.2009.01.035

[47] Streib H, Klein C. Religion and spirituality. In: Stausberg M, Engler S, editors. Oxford Handbook of Religious Studies. Oxford: Oxford University Press; 2016. p. 73–83.

[48] Kleine C. Religion and the secular in premodern Japan from the viewpoint of systems theory. Journal of Religion in Japan. 2013;2(1):1–34. doi: 10.1163/22118349-12341246

[49] Luhmann N. Die Religion der Gesellschaft = The religion of society: Suhrkamp; 2000.

[50] Laermans R, Verschraegen G. ‘The late Niklas Luhmann’ on religion: An overview. Social Compass. 2001;48(1):7–20. doi: 10.1177/003776801048001002

[51] Booth A. Unpacking your literature search toolbox: on search styles and tactics. Health information and Libraries Journal. 2008;25(4):313. doi: 10.1111/j.1471-1842.2008.00825.x 19076679

[52] Bates MJ. The design of browsing and berrypicking techniques for the online search interface. Online Review. 1989;13(5):407–24. doi: 10.1108/eb024320

[53] Boutron I, Page MJ, Higgins JPT, Altman DG, Lundh A, Hróbjartsson A, et al. Considering bias and conflicts of interest among the included studies. In: Higgins JPT, Thomas J, Chandler J, Cumpston M, Li T, Page MJ, et al., editors. Cochrane handbook for systematic reviews of interventions. 2nd ed: John Wiley & Sons; 2019. p. 177–204.

[54] Downes MJ, Brennan ML, Williams HC, Dean RS. Development of a critical appraisal tool to assess the quality of cross-sectional studies (AXIS). BMJ Open. 2016;6(12):e011458. doi: 10.1136/bmjopen-2016-011458 27932337 PMC5168618

[55] Wirsching J, Graßmann S, Eichelmann F, Harms LM, Schenk M, Barth E, et al. Development and reliability assessment of a new quality appraisal tool for cross-sectional studies using biomarker data (BIOCROSS). BMC Medical Research Methodology. 2018;18(1):Article number 122. doi: 10.1186/s12874-018-0583-x 30400827 PMC6219097

[56] Koenig HG. Concerns about measuring “spirituality” in research. The Journal of Nervous and Mental Disease. 2008;196(5):349–55. doi: 10.1097/NMD.0b013e31816ff796 18477877

[57] Zwingmann C, Klein C, Büssing A. Measuring religiosity/spirituality: Theoretical differentiations and categorization of instruments. Religions. 2011;2(3):345–57.

[58] Hsieh H-F, Shannon SE. Three approaches to qualitative content analysis. Qualitative Health Research. 2005;15(9):1277–88. doi: 10.1177/1049732305276687 16204405

[59] World Values Survey. The Inglehart-Welzel World Cultural Map 2023 [cited 2023 Feb 23]. Available from: https://www.worldvaluessurvey.org.

[60] Eid M, Gollwitzer M, Schmitt M. Statistik und Forschungsmethoden Lehrbuch: Beltz; 2011.

[61] Egger M, Smith GD, Schneider M, Minder C. Bias in meta-analysis detected by a simple, graphical test. BMJ. 1997;315(7109):629–34. doi: 10.1136/bmj.315.7109.629 9310563 PMC2127453

[62] Nosek BA, Alter G, Banks GC, Borsboom D, Bowman SD, Breckler SJ, et al. Promoting an open research culture. Science. 2015;348(6242):1422–5. doi: 10.1126/science.aab23 26113702 PMC4550299

[63] Cooper H. Reporting quantitative research in psychology: How to meet APA Style Journal Article Reporting Standards. 2nd ed: American Psychological Association; 2020.

[64] Page MJ, McKenzie JE, Bossuyt PM, Boutron I, Hoffmann TC, Mulrow CD, et al. The PRISMA 2020 statement: An updated guideline for reporting systematic reviews. BMJ. 2021;372:n71. doi: 10.1136/bmj.n71 33782057 PMC8005924

[65] Abel L, Walker C, Samios C, Morozow L. Vicarious posttraumatic growth: Predictors of growth and relationships with adjustment. Traumatology: An International Journal. 2014;20(1):9–18. doi: 10.1037/h0099375

[66] Aderhold C, Morawa E, Paslakis G, Erim Y. Entwicklung und Validierung eines Fragebogens zur Patientenkompetenz im Umgang mit einer Krebserkrankung (PUK) = Development and validation of a questionnaire on patient competency in coping with cancer (PUK). Zeitschrift für Psychosomatische Medizin und Psychotherapie. 2019;65(3):239–56. doi: 10.13109/zptm.2019.65.3.239 31476991

[67] Ahmad S. Coping with cultural conflict: Strategies used by South Asian American women [Doctoral dissertation]: University of La Verne; 2012.

[68] Arya B, Davidson C. Sense of coherence as a predictor of post traumatic growth. Indian Journal of Health & Wellbeing. 2015;6(6):634–6.

[69] Basson M, Rothmann S. Sense of coherence, coping and burnout of pharmacists. South African Journal of Economic and Management Sciences. 2002;5(1):35–62.

[70] Berger D, Fink A, Perez Gomez MM, Lewis A, Unterrainer H-F. The validation of a Spanish version of the multidimensional inventory of religious/spiritual well-being in Mexican college students. The Spanish Journal of Psychology. 2016;19(e3):1–11. doi: 10.1017/sjp.2016.9 26887859

[71] Bossick BE. An empirical examination of the relationship between posttraumatic growth and the personality traits of hardiness, sense of coherence, locus of control, self-efficacy, resilience, and optimism [Doctoral dissertation]: University of Akron; 2008.

[72] Brockhouse R, Msetfi RM, Cohen K, Joseph S. Vicarious exposure to trauma and growth in therapists: The moderating effects of sense of coherence, organizational support, and empathy. Journal of Traumatic Stress. 2011;24(6):735–42. doi: 10.1002/jts.20704 22147494

[73] Büssing A, Günther A, Baumann K, Frick E, Jacobs C. Spiritual dryness as a measure of a specific spiritual crisis in catholic priests: Associations with symptoms of burnout and distress. Evidence-Based Complementary and Alternative Medicine. 2013;2013:Article ID 246797. doi: 10.1155/2013/246797 23843867 PMC3703410

[74] Büssing A, Baumann K, Jacobs C, Frick E. Spiritual dryness in Catholic priests: Internal resources as possible buffers. Psychology of Religion and Spirituality. 2016;9(1):46–55. https://psycnet.apa.org/doi/10.1037/rel0000063.

[75] Chamberlain K, Zika S. Religiosity, Life Meaning and Wellbeing: Some Relationships in a Sample of Women. J Sci Stud Relig. 1988;27(3):411. doi: 10.2307/1387379

[76] Conway-Phillips R, Janusek L. Influence of sense of coherence, spirituality, social support and health perception on breast cancer screening motivation and behaviors in African American women. The ABNF Journal. 2014;25(3):72–9. 25181785

[77] Curtis RC. Coping with prostrate cancer: The effects of family strength, sense of coherence, and spiritual resources [Doctoral dissertation]: University of North Carolina at Greensboro; 2000.

[78] DeBruyn JC. Binge drinking and salutogenesis: Sense of coherence, stress, religiousness and spirituality [Doctoral dissertation]: Western Michigan University Kalamazoo; 2001.

[79] Delgado C. Sense of coherence, spirituality, stress and quality of life in chronic illness. Journal of Nursing Scholarship. 2007;39(3):229–34. doi: 10.1111/j.1547-5069.2007.00173.x 17760795

[80] Dezutter J, Robertson LA, Luyckx K, Hutsebaut D. Life satisfaction in chronic pain patients: The stress-buffering role of the centrality of religion. Journal for the Scientific Study of Religion. 2010;49(3):507–16. doi: 10.1111/j.1468-5906.2010.01525.x 20886698

[81] Diaconescu LV, Gheorghe IR, Chesches T, Popa-Velea O. Psychological variables associated with HPV vaccination intent in Romanian academic settings. Int J Environ Res Public Health. 2021;18:8938. doi: 10.3390/ijerph18178938 34501527 PMC8430846

[82] Diržytė A, Patapas A, Limantaitė E. Religious beliefs, sense of coherence and well being of Lithuanian students. Socialinis Darbas. 2003;1(3):15–22.

[83] Dyer WJ. Refining research on the intersection between sexual orientation, suicide, and religiosity. Psychol Relig Spiritual. 2022;14(2):179–88. doi: 10.1037/rel0000451

[84] Ebrahimi Meymand HA, Askarizadeh G, Bagheri M, Arabnejad M. The role of spiritual intelligence, sense of coherence, and cognitive flexibility as internal resources in predicting perceived stress in patients with multiple sclerosis. Quarterly Horizon of Medical Sciences. 2021;27(1):114–29. doi: 10.32598/hms.27.1.2986.2

[85] Edwards D, Besseling E. Relationship between depression, anxiety, sense of coherence, social support and religious involvement in a small rural community affected by industrial relations conflict. South African Journal of Psychology. 2001;31(4):62–71. doi: 10.1177/008124630103100408

[86] Encarnação P, Oliveira CC, Martins T. Psychometric properties of the suffering assessment questionnaire in adults with chronic diseases or life‐threatening illness. Scandinavian Journal of Caring Sciences. 2018;32(4):1279–87. doi: 10.1111/scs.12569 29578241

[87] Farhadi A, Bahreini M, Moradi A, Mirzaei K, Nemati R. The predictive role of coping styles and sense of coherence in the post-traumatic growth of mothers with disabled children: A cross-sectional study. BMC Psychiatry. 2022;22(1):708. doi: 10.1186/s12888-022-04357-5 36380328 PMC9667566

[88] Fernandez-Martinez E, Lopez-Alonso AI, Marques-Sanchez P, Martinez-Fernandez MC, Sanchez-Valdeon L, Liebana-Presa C. Emotional intelligence, sense of coherence, engagement and coping: A cross-sectional study of university students’ health. Sustainability. 2019;11(24):6953. doi: 10.3390/su11246953

[89] Ferreira DC, Gonçalves TR, Celeste RK, Olinto MTA, Pattussi MP. Psychosocial aspects and the impact of oral health on quality of life of Brazilian adults. Brazilian Journal of Epidemiology. 2020;23(e200049):1–13. doi: 10.1590/1980-549720200049 32520100

[90] Forstmeier S, Kuwert P, Spitzer C, Freyberger HJ, Maercker A. Posttraumatic growth, social acknowledgment as survivors, and sense of coherence in former German child soldiers of World War II. The American Journal of Geriatric Psychiatry. 2009;17(12):1030–9. doi: 10.1097/JGP.0b013e3181ab8b36 20104060

[91] Gabrielsen LE, Ulleberg P, Watten RG. The Adolescent Life Goal Profile Scale: Development of a new scale for measurements of life goals among young people. Journal of Happiness Studies. 2012;13(6):1053–72. doi: 10.1007/s10902-011-9306-2

[92] Gabrielsen LE, Watten RG, Ulleberg P. Differences on Adolescent Life Goal Profile Scale between a clinical and non-clinical adolescent sample. International Journal of Psychiatry in Clinical Practice. 2013;17(4):244–52. doi: 10.3109/13651501.2012.745573 23116240

[93] Gavulic AM. Examining the effect of parental attachment, emotional maturity, spiritual maturity, and view of suffering on sense of coherence [Doctoral dissertation]: Liberty University; 2018.

[94] Ghazinour M, Richter J, Eisemann M. Quality of life among Iranian refugees resettled in Sweden. Journal of Immigrant Health. 2004;6(2):71–81. doi: 10.1023/B:JOIH.0000019167.04252.58 15014224

[95] Gibson LM. Inner resources (sense of coherence, hope, and spiritual perspective) as predictors of psychological well-being in African American breast cancer survivors [Doctoral dissertation]: University of South Carolina; 2000.

[96] Goulding A. Schizotypy models in relation to subjective health and paranormal beliefs and experiences. Personality and Individual Differences. 2004;37(1):157–67. doi: 10.1016/j.paid.2003.08.008

[97] Goulding A. Healthy schizotypy in a population of paranormal believers and experients. Personality and Individual Differences. 2005;38(5):1069–83. doi: 10.1016/j.paid.2004.07.006

[98] Hammer JH, Cragun RT, Hwang K. Measuring spiritual fitness: Atheist military personnel, veterans, and civilians. Military Psychology. 2013;25(5):438–51. doi: 10.1037/mil0000010

[99] Herbst L, Coetzee S, Visser D. Personality, sense of coherence and the coping of working mothers. SA Journal of Industrial Psychology. 2007;33(3):57–67. https://hdl.handle.net/10520/EJC89274.

[100] Hossack RC. Salutogenic and pathogenic orientations to life: Attachment, personality, sense of coherence and well-being in late adolescence: A structural equation model [Doctoral dissertation]: University of Manitoba; 1997.

[101] Israelashvili M, Taubman-Ben-Ari O, Hochdorf Z. A multidimensional approach to explore cross-cultural differences in coping behavior: comparing Druze and Jews in Israel. The Journal of Social Psychology. 2011;151(1):31–50. doi: 10.1080/00224540903366651 21375124

[102] Johnson-Migalski L. Levels and correlations of sense of coherence, depression and spirituality/religiousness in the medically stable and unstable elderly [Doctoral dissertation]: Adler School of Professional Psychology; 2006.

[103] Kerksieck P, Büssing A, Frick E, Jacobs C, Baumann K. Reduced sense of coherence due to neuroticism: Are transcendent beliefs protective among Catholic pastoral workers? Journal of Religion and Health. 2016;56(6):1956–70. 10.1007/s10943-016-0322-8 27787694

[104] Khanjari S, Oskouie F, Langiu-Eklöf A. Lower sense of coherence, negative religious coping, and disease severity as indicators of a decrease in quality of life in Iranian family caregivers of relatives with breast cancer during the first 6 months after diagnosis. Cancer Nursing. 2012;35(2):148–56. doi: 10.1097/NCC.0b013e31821f1dda 21760493

[105] Kibour Y. The moderating role of sense of coherence between migration stress and ajustment among Ethiopian immigrants [Doctoral dissertation]: Howard University; 2002.

[106] Kim S, Zemon V, Foley FW. Measuring personal growth in partners of persons with multiple sclerosis: A new scale. Rehabilitation Psychology. 2020;65(3):219–30. doi: 10.1037/rep0000299 31855017

[107] Kohls N, Walach H. Validating four standard scales in spiritually practicing and nonpracticing samples using propensity score matching. European Journal of Psychological Assessment. 2008;24(3):165–73.

[108] Krok D. Sense of coherence mediates the relationship between the religious meaning system and coping styles in Polish older adults. Aging & Mental Health. 2016;20(10):1002–9. doi: 10.1080/13607863.2015.1056772 26091064

[109] Lee LE. Internal coping resources as predictive of individual outcome in outpatient drug rehabilitation treatment [Doctoral dissertation]: City University of New York; 1998.

[110] Littlejohn EM. The relationship between the components of Black feminism and psychological health in African American women [Doctoral dissertation]: Ohio State University; 1994.

[111] López J, Camilli C, Noriega C. Posttraumatic growth in widowed and non-widowed older adults: Religiosity and sense of coherence. Journal of Religion and Health. 2015;54(5):1612–28. doi: 10.1007/s10943-014-9876-5 24839098

[112] Magnano PA. Hope: Building a schema [Doctoral dissertation]: University of Washington; 2003.

[113] Meghani SH, Peterson C, Kaiser DH, Rhodes J, Rao H, Chittams J, et al. A pilot study of a mindfulness-based art therapy intervention in outpatients with cancer. American Journal of Hospice & Palliative Medicine. 2018;35(9):1195–200. doi: 10.1177/1049909118760304 29514486

[114] Mirsoleymani S, Matbouei M, Vasli P, Marzaleh MA, Rohani C. The role of family caregiver’s sense of coherence and family adaptation determinants in predicting distress and caregiver burden in families of cancer patients. Indian Journal of Palliative Care. 2021;27(1):47–53. doi: 10.4103/IJPC.IJPC_112_20 34035617 PMC8121216

[115] Nahlén C, Saboonchi F. Coping, sense of coherence and the dimensions of affect in patients with chronic heart failure. European Journal of Cardiovascular Nursing. 2010;9(2):118–25. doi: 10.1016/j.ejcnurse.2009.11.006 20022304

[116] Nishi D, Matsuoka Y, Kim Y. Posttraumatic growth, posttraumatic stress disorder and resilience of motor vehicle accident survivors. Biopsychosocial Medicine. 2010;4(7):1–6. doi: 10.1186/1751-0759-4-7 20573276 PMC2914073

[117] Nolvi M, Brogårdh C, Jacobsson L, Lexell J. Sense of coherence and coping behaviours in persons with late effects of polio. Annals of Physical and Rehabilitation Medicine. 2022;65(3):101577. doi: 10.1016/j.rehab.2021.101577 34624546

[118] Ozaki M. Development of an assessment tool on spirituality explained by three domains, Will, joy and sense: From a holistic educational approach. Journal of International Society of Life Information Science. 2005;23(2):364–9.

[119] Pakenham KI. Making sense of caregiving for persons with multiple sclerosis (MS): The dimensional structure of sense making and relations with positive and negative adjustment. International Journal of Behavioral Medicine. 2007;15(3):241–52. doi: 10.1080/10705500802222345 18696319

[120] Pakenham KI, Cox S. Development of the benefit finding in multiple sclerosis (MS) caregiving scale: A longitudinal study of relations between benefit finding and adjustment. British Journal of Health Psychology. 2008;13(4):583–602. doi: 10.1348/135910707X250848 18854061

[121] Post-White J, Ceronsky C, Kreitzer MJ, Nickelson K, Drew D, Mackey KW, et al. Hope, spirituality, sense of coherence, and quality of life in patients with cancer. Oncology Nursing Forum. 1996;23(10):1571–9. 9348591

[122] Racklin JM. The roles of sense of coherence, spirituality, and religion in responses to trauma [Doctoral dissertation]: California School of Professional Psychology at Alameda; 1999.

[123] Ragger K, Hiebler-Ragger M, Herzog G, Kapfhammer H-P, Unterrainer H-F. Sense of coherence is linked to post-traumatic growth after critical incidents in Austrian ambulance personnel. BMC Psychiatry. 2019;19(89):1–11. doi: 10.1186/s12888-019-2065-z 30866860 PMC6417083

[124] Reguera-García MM, Liébana-Presa C, Álvarez-Barrio L, Alves Gomes L, Fernández-Martínez E. Physical activity, resilience, sense of coherence and coping in people with multiple sclerosis in the situation derived from COVID-19. International Journal of Environmental Research and Public Health. 2020;17(21):1–13. doi: 10.3390/ijerph17218202 33172022 PMC7664264

[125] Renner W, Salem I, Alexandrowicz R. Human values as predictors for political, religious and health-related attitudes: A contribution towards validating the Austrian Value Questionnaire (AVQ) by structural equation modeling. Social Behavior and Personality. 2004;32(5):477–90. doi: 10.2224/sbp.2004.32.5.477

[126] Rohani C, Khanjari S, Abedi H-A, Oskouie F, Langius-Eklöf A. Health index, sense of coherence scale, brief religious coping scale and spiritual perspective scale: Psychometric properties. Journal of Advanced Nursing. 2010;66(12):2796–806. doi: 10.1111/j.1365-2648.2010.05409.x 20722813

[127] Rothmann S, Van Rensburg P. Psychological strengths, coping and suicide ideation in the South African Police Services in the North West Province. SA Journal of Industrial Psychology. 2002;28(3):39–49. https://hdl.handle.net/10520/EJC88918.

[128] Schonder M. Sense of coherence among religious and non-religious students from Germany and Poland [Doctoral dissertation]: Technische Universität Carola-Wilhelmina zu Braunschweig; 2016.

[129] Skalski‐Bednarz SB, Konaszewski K, Muszyńska J, Maier K, Surzykiewicz J. Negative situation appraisal and mental well‐being among refugees in Germany: Serial mediation by religious coping and sense of coherence. Int Migr. 2022;00:1–13. doi: 10.1111/imig.13087

[130] Skowronski B, Talik E. Psychosocial quality of life and its’ correlates in people serving prison sentences in penitentiary institutions. Int J Offender Ther Comp Criminol. 2021;65(5):590–612. doi: 10.1177/0306624X20944685 32697119

[131] Strümpfer DJW. The relation between religious motivation and work-related variables amongst agricultural workers. South African Journal of Psychology. 1997;27(3):134–42. https://psycnet.apa.org/doi/10.1177/008124639702700302.

[132] Unterrainer H-F, Ladenhauf KH. Religiös-spirituelles Befinden im Kontext seelischer Gesundheit und Krankheitsverarbeitung: Ergebnisse eines interdisziplinären Forschungsprojekts = Religious-spiritual well-being in the context of mental health and coping with illness: Results of an interdisciplinary research project. Psychologie in Österreich. 2008;28(1):54–61.

[133] Tagay S, Erim Y, Brähler E, Senf W. Religiosity and sense of coherence–Protective factors of mental health and well-being? Zeitschrift für Medizinische Psychologie. 2006;15(4):165–71.

[134] Torinomi C, Lindenberg K, Möltner A, Herpertz SC, Holm-Hadulla RM. Predictors of students’ mental health during the COVID-19 pandemic: The impact of coping strategies, sense of coherence, and social support. Int J Environ Res Public Health. 2022;19(24):16423. doi: 10.3390/ijerph192416423 36554304 PMC9778699

[135] Unterrainer H-F, Huber H-P, Ladenhauf KH, Wallner-Liebmann SJ, Liebmann PM. MI-RSB 48. Die Entwicklung eines multidimensionalen Inventars zum religiös-spirituellen Befinden = The development of a multidimensional inventory of religious-spiritual well-being. Diagnostica. 2010;56(2):82–93. doi: 10.1026/0012-1924/a000001

[136] Unterrainer H-F, Lewis A, Collicutt J, Fink A. Religious/spiritual well-being, coping styles, and personality dimensions in people with substance use disorders. International Journal for the Psychology of Religion. 2013;23(3):204–13. doi: 10.1080/10508619.2012.714999

[137] Uren TH, Wastell CA. Attachment and meaning-making in perinatal bereavement. Death Studies. 2002;26(4):279–308. doi: 10.1080/074811802753594682 11980450

[138] Van der Colff JJ, Rothmann S. Occupational stress, sense of coherence, coping, burnout and work engagement of registered nurses in South Africa. SA Journal of Industrial Psychology. 2009;35(1):1–10. doi: 10.4102/sajip.v35i1.423

[139] Verouli P, Siafaka V, Ageli A. Association between the fear of pain, the response strategies and the sense of coherence in workers in primary health care. International Journal of Caring Sciences. 2016;9(3):1106–16.

[140] Vosloo C, Wissing MP, Temane QM. Gender, spirituality and psychological well-being. Journal of Psychology in Africa. 2009;19(2):153–9. doi: 10.1080/14330237.2009.10820274

[141] Wenzl M, Fuchshuber J, Podolin-Danner N, Silani G, Unterrainer H-F. The Swedish version of the Multidimensional Inventory for Religious/Spiritual Well-Being: First results from Swedish students. Front Psychol. 2021;12:783761. doi: 10.3389/fpsyg.2021.783761 34858301 PMC8630782

[142] Wilkins TA, Piedmont RL, Magyar-Russell GM. Spirituality or religiousness: Which serves as the better predictor of elements of mental health? Research in the Social Scientific Study of Religion. 2012;23:53–73.

[143] Wissing JAB, Wissing MP, du Toit MM, Temane QM. Psychometric properties of various scales measuring psychological well-being in a South African context: The FORT 1 Project. Journal of Psychology in Africa. 2008;18(4):511–20. doi: 10.1080/14330237.2008.10820230

[144] Zafar H, Khan SH, Bhatti MI, Hussain MM. Spirituality, sense of coherence, resilience, and stress among earthquake survivors of Azad Jammu and Kashmir. Pakistan Journal of Social Sciences. 2019;39(4):1511–9.

[145] Zamanian H, Amini‐Tehrani M, Mahdavi Adeli A, Daryaafzoon M, Arsalani M, Enzevaei A, et al. Sense of coherence and coping strategies: How they influence quality of life in Iranian women with breast cancer. Nursing Open. 2021;8:1731–40. doi: 10.1002/nop2.814 33608988 PMC8186695

[146] Zarzycka B, Rydz E. Centrality of religiosity and sense of coherence: A cross-sectional study with Polish young, middle and late adults. International Journal of Social Science Studies. 2014;2(2):126–36. doi: 10.11114/ijsss.v2i2.346

[147] Zarzycka B, Rydz E. Explaining the relationship between post-critical beliefs and sense of coherence in Polish young, middle, and late adults. Journal of Religion & Health. 2014;53(3):834–48. doi: 10.1007/s10943-013-9680-7 23370860 PMC3982208

[148] Zehnder Grob S. Religiosität, psychische Gesundheit und Kohärenzsinn: Eine empirische Befragungsstudie Adoleszenter [Doctoral dissertation]: Technische Universität Dortmund; 2015.

[149] Zerach G. Compassion fatigue and compassion satisfaction among residential child care workers: The role of personality resources. Residential Treatment for Children & Youth. 2013;30(1):72–91. doi: 10.1080/0886571X.2012.761515

[150] Zerach G, Levin Y. Posttraumatic stress symptoms, burn-out, and compassion satisfaction among body handlers: The mediating role of sense of coherence and spirituality at workplace. Journal of Interpersonal Violence. 2018;33(12):1931–57. doi: 10.1177/0886260515621065 26685213

[151] Cassel L, Suedfeld P. Salutogenesis and autobiographical disclosure among Holocaust survivors. The Journal of Positive Psychology. 2006;1(4):212–25. doi: 10.1080/17439760600952919

[152] Cohen O. On the origins of a sense of coherence: Sociodemographic characteristics, or narcissism as a personality trait. Social Behavior and Personality. 1997;25(1):49–57. doi: 10.2224/sbp.1997.25.1.49

[153] Kimhi S, Eshel Y, Lahad M, Leykin D. National Resilience: A new self-report assessment scale. Community Mental Health Journal. 2019;55(4):721–31. doi: 10.1007/s10597-018-0362-5 30600401

[154] Marciano H, Kimhi S, Eshel Y. Predictors of individual, community and national resiliencies of Israeli Jews and Arabs. International Journal of Psychology 2019;55(4):553–61. doi: 10.1002/ijop.12636 31792956

[155] Nanín JE. Burnout, sense of coherence, and health status in New York City HIV service providers [Doctoral dissertation]: Columbia University; 2001.

[156] Senka J. Coping processes in groups at risk in the context of psychological health aspects. Studia Psychologica. 1995;37(3):154–6.

[157] von Humboldt S, Leal I, Pimenta F. Sense of coherence, sociodemographic, lifestyle, and health-related factors in older adults’ subjective well-being. International Journal of Gerontology. 2015;9(1):15–9.

[158] Huber S, Huber OW. The centrality of religiosity scale (CRS). Religions. 2012;3(3):710–24. doi: 10.3390/rel3030710

[159] Stark R. Physiology and faith: Addressing the “universal” gender difference in religious commitment. Journal for the Scientific Study of Religion. 2002;41(3):495–507. doi: 10.1111/1468-5906.00133

[160] Moon JW, Tratner AE, McDonald MM. Men are less religious in more gender-equal countries. Proceedings of the Royal Society B. 2022;289(1968):20212474. doi: 10.1098/rspb.2021.2474 35105234 PMC8808095

[161] Joan D, Reutter L. Socioeconomic status, sense of coherence and health in Canadian women. Canadian Journal of Public Health. 2003;94(3):224–8. doi: 10.1007/BF03405071 12790499 PMC6979888

[162] Barnard A. The role of socio-demographic variables and their interaction effect on sense of coherence. SA Journal of Industrial Psychology. 2013;39(1):1–9. https://hdl.handle.net/10520/EJC144496

[163] Barnard A. Sense of coherence: A distinct perspective on financial well-being. South African Journal of Economic and Management Sciences. 2016;19(4):647–60.

[164] Paden WE. The prestige of the gods: Evolutionary continuities in the formation of sacred objects. In: Geertz AW, editor. Origins of religion, cognition and culture: Routledge; 2014. p. 96–111.

[165] Pargament KI, Koenig HG, Perez LM. The many methods of religious coping: Development and initial validation of the RCOPE. Journal of Clinical Psychology. 2000;56(4):519–43. doi: 10.1002/(sici)1097-4679(200004)56:4&lt;519::aid-jclp6&gt;3.0.co;2-1 10775045

[166] Salsman JM, Fitchett G, Merluzzi TV, Sherman AC, Park CL. Religion, spirituality, and health outcomes in cancer: A case for a meta‐analytic investigation. Cancer. 2015a;121(21):3754–9. doi: 10.1002/cncr.29349 26258400 PMC4618242

[167] Salsman JM, Pustejovsky JE, Jim HSL, Munoz AR, Merluzzi TV, George L, et al. A meta‐analytic approach to examining the correlation between religion/spirituality and mental health in cancer. Cancer. 2015b;121(21):3769–78. doi: 10.1002/cncr.29350 26258536 PMC4618157

[168] Eriksson M, Mittelmark MB. The sense of coherence and its measurement. In: Mittelmark MB, Bauer GF, Vaandrager L, Pelikan JM, Sagy S, Eriksson M, et al., editors. The handbook of salutogenesis: Springer; 2017. p. 97–103.

[169] Küng H. What I believe: Bloomsbury Publishing; 2010.

[170] Küng H. Basic trust as the foundation of a global ethic. International Review of Psychiatry. 2001;13(2):94–100.

[171] Tobacyk JJ. A revised paranormal belief scale. The International Journal of Transpersonal Studies. 2004;23(23):94–8.

[172] Pollack D. Religion und gesellschaftliche Differenzierung: Studien zum religiösen Wandel in Europa und den USA III: Mohr Siebeck; 2016.

[173] May M. Should I stay or should I go? Religious (dis) affiliation and depressive symptomatology. Society and Mental Health. 2018;8(3):214–30. doi: 10.1177/2156869317748

[174] Ghorbani N, Chen ZJ, Rabiee F, Watson PJ. Religious fundamentalism in Iran: Religious and psychological adjustment within a Muslim cultural context. Archive for the Psychology of Religion. 2019;41(2):73–88. doi: 10.1177/0084672419878832

[175] Gebauer JE, Sedikides C, Schönbrodt FD, Bleidorn W, Rentfrow PJ, Potter J, et al. The religiosity as social value hypothesis: A multi-method replication and extension across 65 countries and three levels of spatial aggregation. Journal of Personality and Social Psychology. 2017;113(3):e18–e39. doi: 10.1037/pspp0000104 27442765

[176] Cederblad M, Ruksachatkunakorn P, Boripunkul T, Intraprasert S, Höök B. Sense of coherence in a Thai sample. Transcultural Psychiatry. 2003;40(4):585–600. doi: 10.1177/1363461503404007 14979469

[177] Bonanato K, Branco DBT, Mota JPT, Ramos-Jorge ML, Paiva SM, Pordeus IA, et al. Trans-cultural adaptation and psychometric properties of the Sense of Coherence Scale in mothers of preschool children. Revista Interamericana de Psicología/Interamerican Journal of Psychology. 2009;43(1):144–53.

[178] Jones AE, Elliott M. Examining social desirability in measures of religion and spirituality using the bogus pipeline. Review of Religious Research. 2017;59(1):47–64.

[179] van de Mortel TF. Faking it: Social desirability response bias in self-report research. Australian Journal of Advanced Nursing. 2008;25(4):40–8.

[180] Jeserich F, Zwingmann C, Klein C. Religiosität/Spiritualität und rigides Kohärenzgefühl = Religiosity/spirituality and rigid sense of coherence. In: Zwingmann C, Klein C, Jeserich F, editors. Religiosität: die dunkle Seite = Religiosity: The dark side: Waxmann; 2017. p. 91–113.

