# Supplementary material for: Sense of coherence and religion/spirituality: A systematic review and meta-analysis based on a methodical classification of instruments measuring religion/spirituality
Source: PLoS One. 2023 Aug 3;18(8):e0289203. doi: 10.1371/journal.pone.0289203 (PMC10399782; doi:10.1371/journal.pone.0289203)

Fig 17. Baujat Plots for the Subgroup of Positive R/S Measures.

Fig A. Baujat Plot of All Included Studies with a Positive R/S Measure.

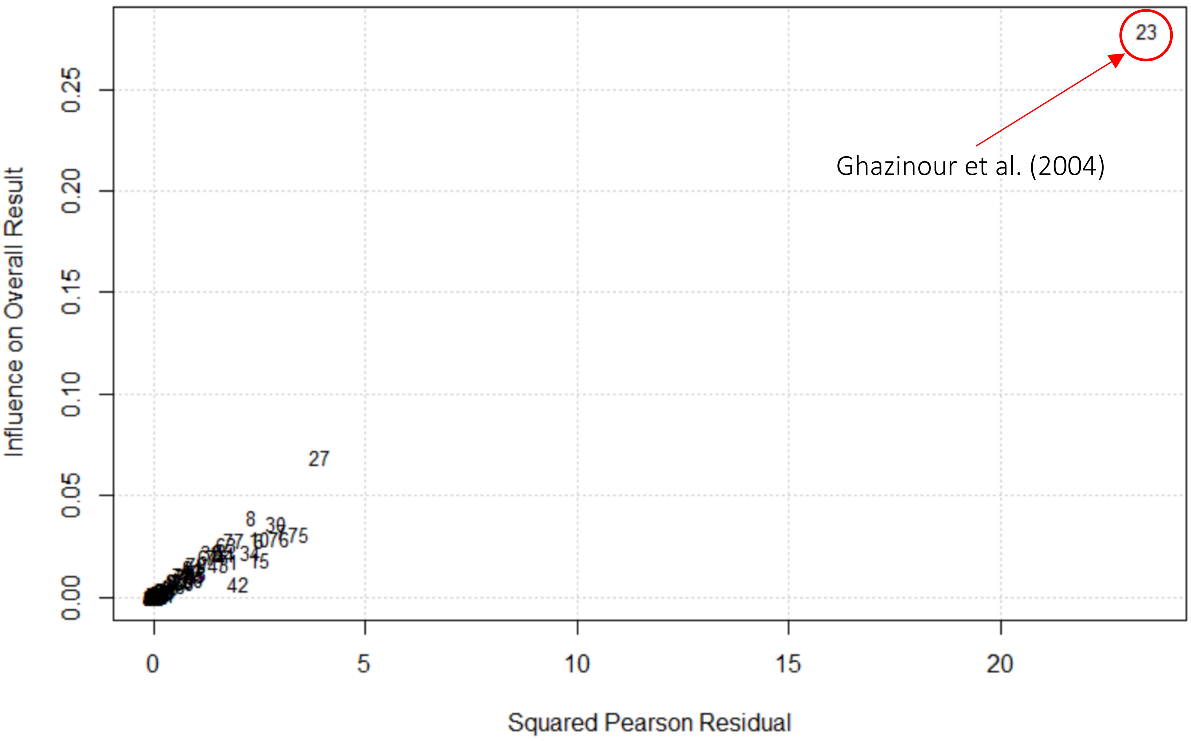

Fig B. Baujat Plot of All Included Studies with a Positive R/S Measure Without the Study of Ghazinour et al. (2004)

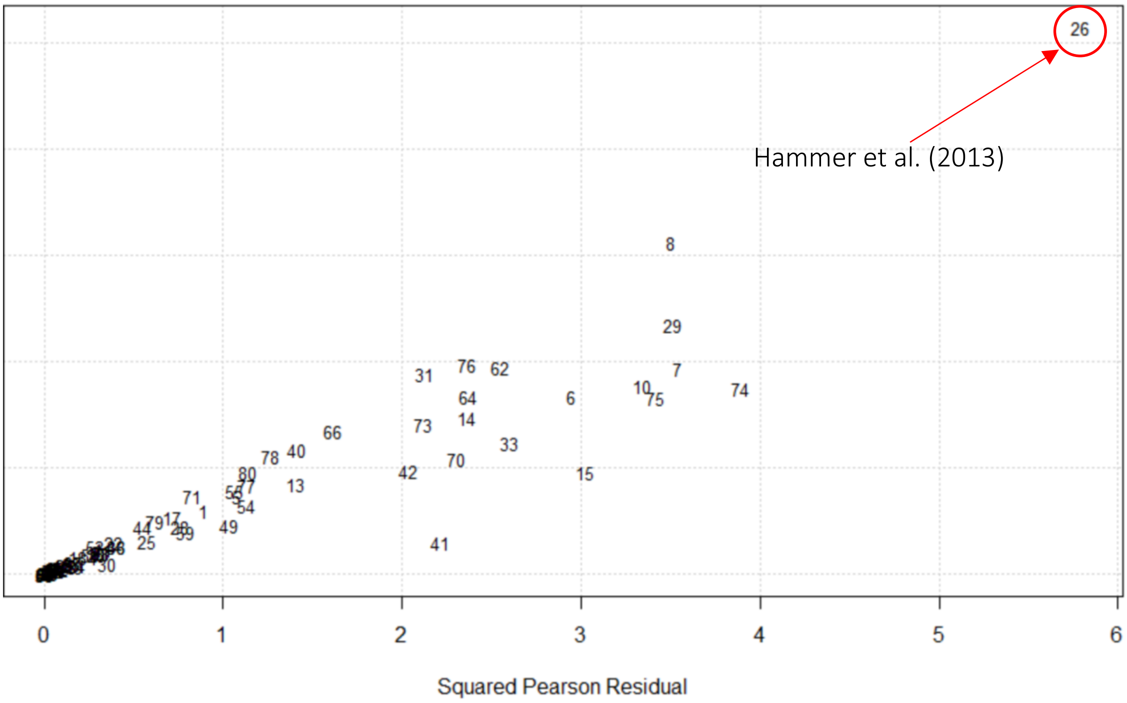

Fig C. Baujat Plot of All Included Studies with a Positive R/S Measure Without the Studies of Ghazinour et al. (2004) and Hammer et al. (2013)

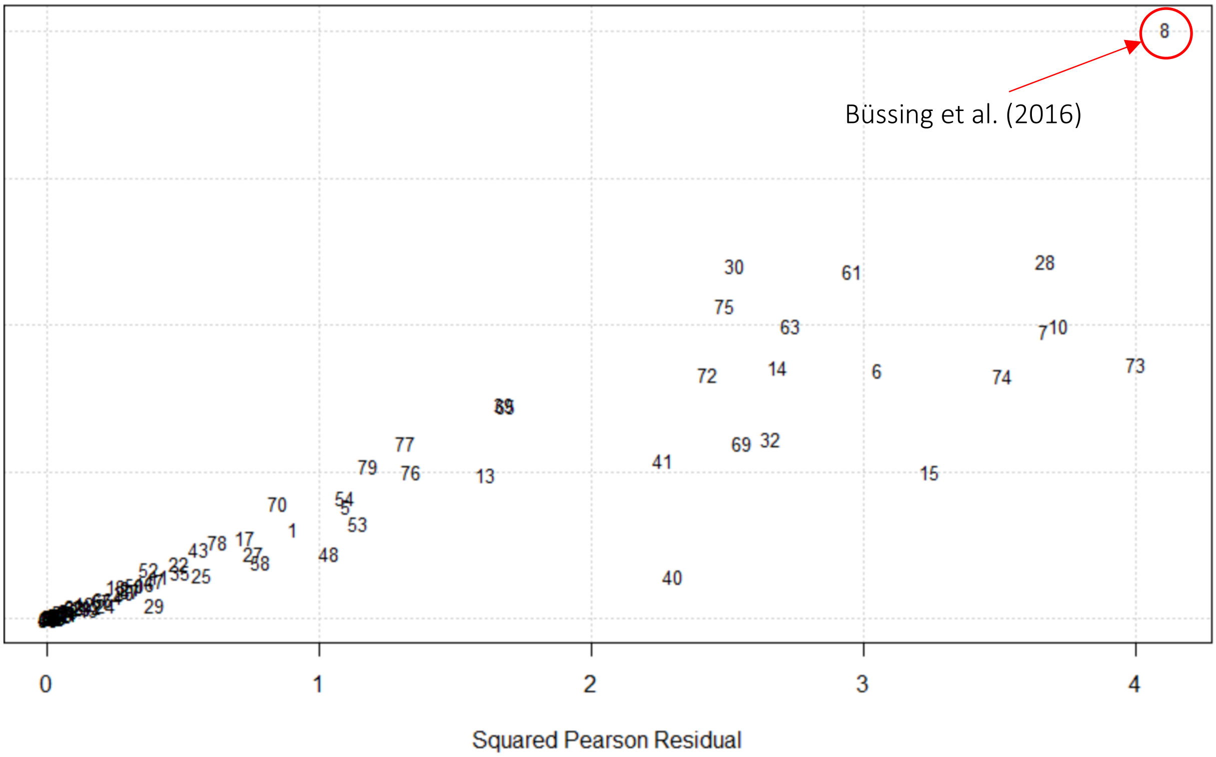

Supplement: S2 Fig — (PDF) [file pone.0289203.s003.pdf]
