# Supplementary material for: Sense of coherence and religion/spirituality: A systematic review and meta-analysis based on a methodical classification of instruments measuring religion/spirituality
Source: PLoS One. 2023 Aug 3;18(8):e0289203. doi: 10.1371/journal.pone.0289203 (PMC10399782; doi:10.1371/journal.pone.0289203)
Supplement: S3 Fig — (PDF) [file pone.0289203.s004.pdf]

S19 Fig. Funnel Plots for the Subgroup of Studies With a Positive R/S Measure.

Fig A. Funnel Plots for All Included Studies With a Positive R/S Measure.

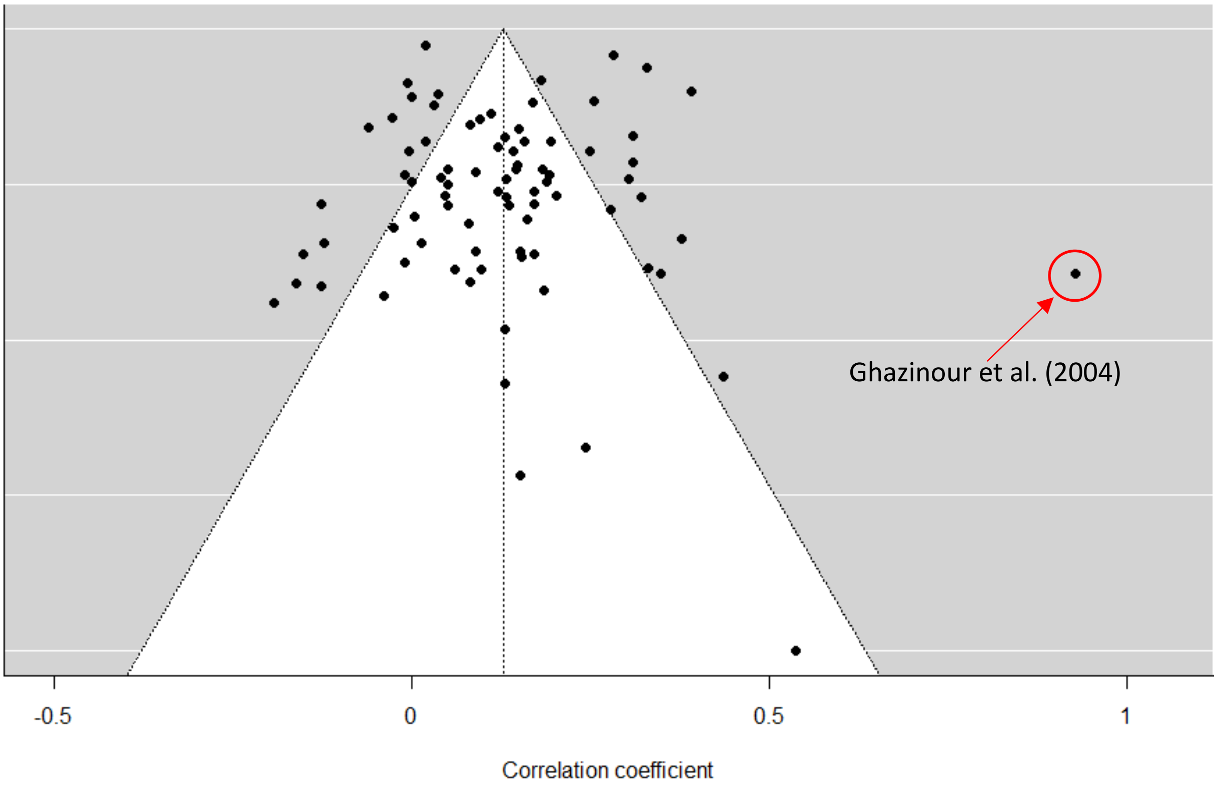

Regression Test for Funnel Plot Asymmetry

Model: mixed-effects meta-regression model

Predictor: standard error

Test for Funnel Plot Asymmetry: z = 0.3730, p = 0.7092

Limit Estimate (as sei-> 0): b = 0.1157 (CI: 0.0437, 0.1876)

Rank Correlation Test for Funnel Plot Asymmetry: Kendall's tau =-0.0062, p = 0.9350

Fig B. Funnel Plots for All Included Studies With a Positive R/S Measure Without Ghazinour et al. (2004)

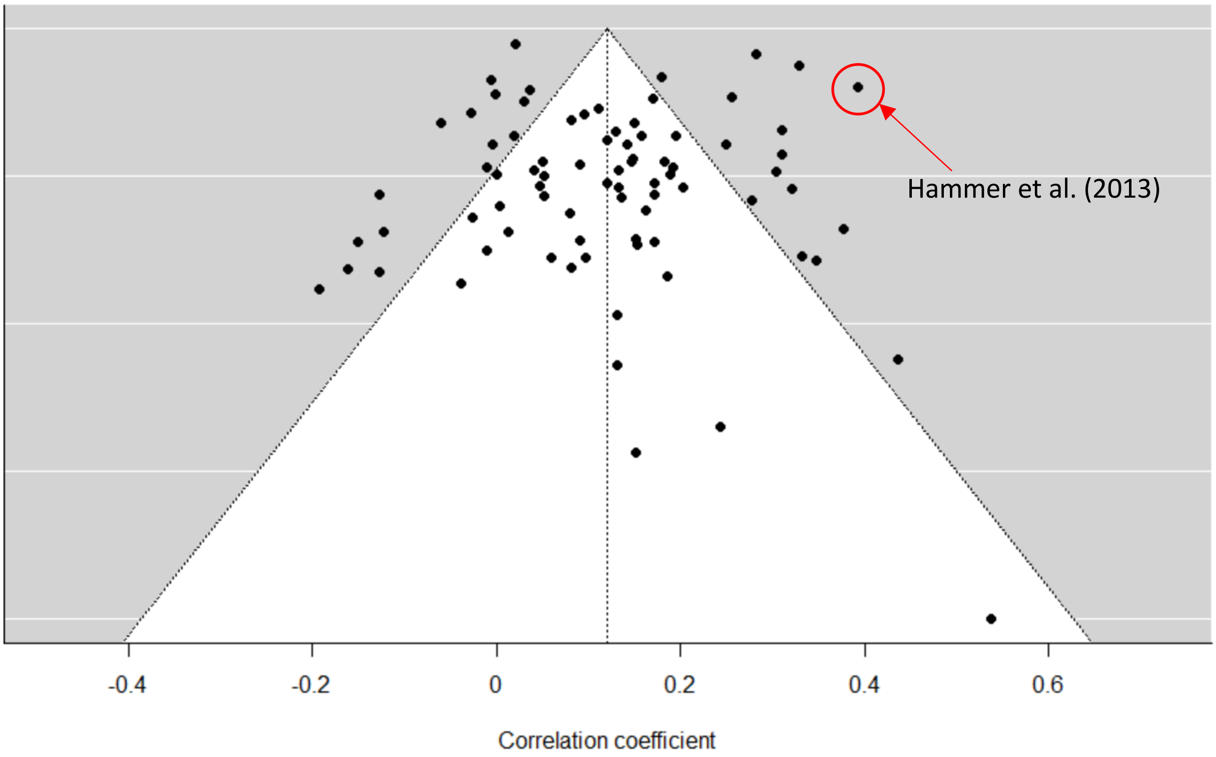

Regression Test for Funnel Plot Asymmetry

Model: mixed-effects meta-regression model

Predictor: standard error

Test for Funnel Plot Asymmetry: z =-0.3716, p = 0.7102

Limit Estimate (as sei-> 0): b = 0.1307 (CI: 0.0680, 0.1934)

Rank Correlation Test for Funnel Plot Asymmetry: Kendall's tau =-0.0250, p = 0.7427

Fig C. Funnel Plots for All Included Studies With a Positive R/S Measure Without Ghazinour et al. (2004) and Hammer at al. (2013)

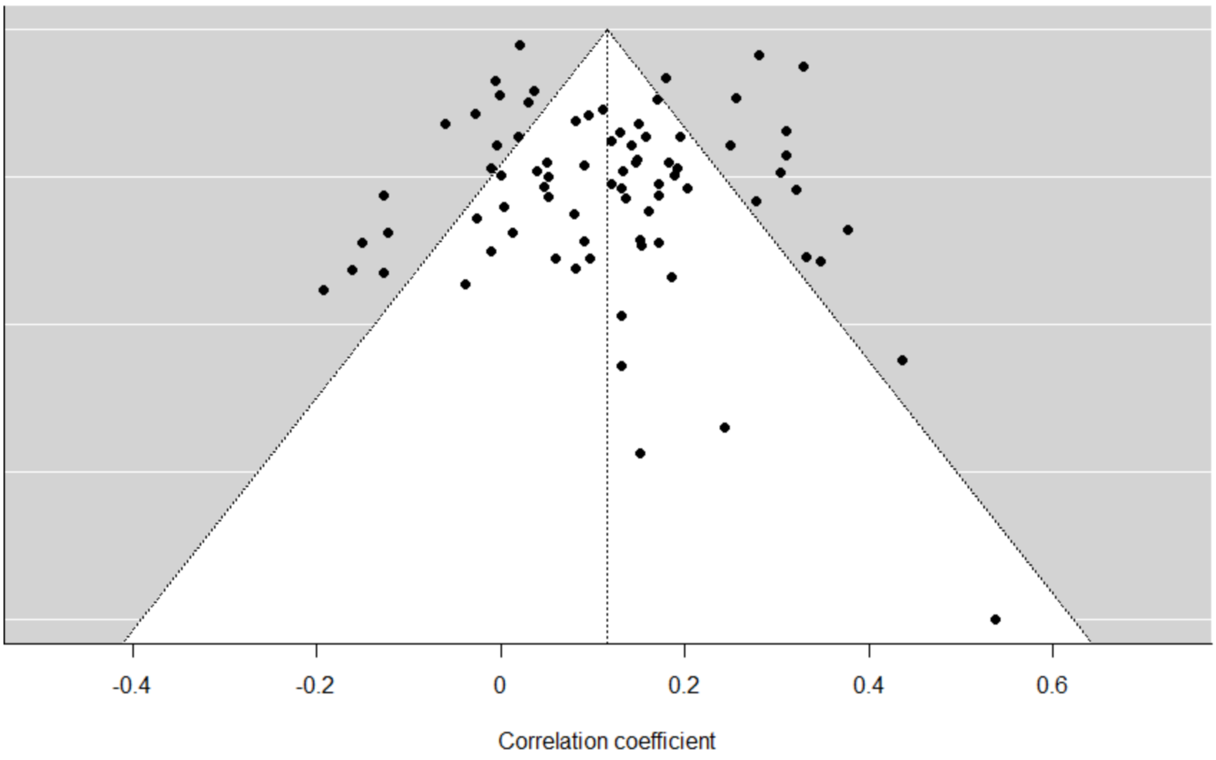

Regression Test for Funnel Plot Asymmetry

Model: mixed-effects meta-regression model

Predictor: standard error

Test for Funnel Plot Asymmetry: z =-0.0529, p = 0.9578

Limit Estimate (as sei-> 0): b = 0.1171 (CI: 0.0558, 0.1784)

Rank Correlation Test for Funnel Plot Asymmetry: Kendall's tau =-0.0150, p = 0.8456
