# Supplementary material for: Sense of coherence and religion/spirituality: A systematic review and meta-analysis based on a methodical classification of instruments measuring religion/spirituality
Source: PLoS One. 2023 Aug 3;18(8):e0289203. doi: 10.1371/journal.pone.0289203 (PMC10399782; doi:10.1371/journal.pone.0289203)
Supplement: S1 Table — (PDF) [file pone.0289203.s005.pdf]

S3 Table. Boolean Search Strategy.

| Facet A: salutogenesis | Facet B: religion/spirituality | Facet C: quantitative design |
|---|---|---|
| Antonovsky* | religio* | association* |
| Kohärenz* | spiritu* | correlation* |
| salutogen* | | Korrelation* |
| “sense of coherence” | | Pearson* |
| | | quantitativ* |
| | | regression |
| | | relation* |
| | | Spearman* |
| **Boolean phrase**: (Antonovsky* OR Kohärenz* OR salutogen* OR “sense of coherence”) AND (religio* OR spiritu*) AND (association* OR correlation* OR Korrelation* OR Pearson* OR quantitativ* OR regression OR relation* OR Spearman*) | | |

*Note.* * = Words match if they begin with the word preceding the asterisk; “ “ = A phrase that is double-quoted will only be found if it is word-for-word the same.
