# Supplementary material for: Sense of coherence and religion/spirituality: A systematic review and meta-analysis based on a methodical classification of instruments measuring religion/spirituality
Source: PLoS One. 2023 Aug 3;18(8):e0289203. doi: 10.1371/journal.pone.0289203 (PMC10399782; doi:10.1371/journal.pone.0289203)
Supplement: S2 Table — Exact representation of the search algorithms in each of the databases used. (PDF) [file pone.0289203.s006.pdf]

S4 Table. Search Queries in the Databases.

Database	Boolean phrase	Search fields	Time period
Academic Search Ultimate	AB (Antonovsky* OR Kohärenz* OR salutogen* OR “sense of coherence”) AND AB (religio* OR spiritu*) AND TX (association* OR correlation* OR Korrelation* OR Pearson* OR quantitativ* OR regression OR relation* OR Spearman*)	Facet A: abstract<br>Facet B: abstract<br>Facet C: all text	No limit
APA PsycArticles	AB (Antonovsky* OR Kohärenz* OR salutogen* OR “sense of coherence”) AND AB (religio* OR spiritu*) AND TX (association* OR correlation* OR Korrelation* OR Pearson* OR quantitativ* OR regression OR relation* OR Spearman*)	Facet A: abstract<br>Facet B: abstract<br>Facet C: all text	No limit
APA PsycInfo	AB (Antonovsky* OR Kohärenz* OR salutogen* OR “sense of coherence”) AND AB (religio* OR spiritu*) AND TX (association* OR correlation* OR Korrelation* OR Pearson* OR quantitativ* OR regression OR relation* OR Spearman*)	Facet A: abstract<br>Facet B: abstract<br>Facet C: all text	No limit
CINAHL	AB (Antonovsky* OR Kohärenz* OR salutogen* OR “sense of coherence”) AND AB (religio* OR spiritu*) AND TX (association* OR correlation* OR Korrelation* OR Pearson* OR quantitativ* OR regression OR relation* OR Spearman*)	Facet A: abstract<br>Facet B: abstract<br>Facet C: all text	No limit
EMBASE	coherence AND religion OR spirituality	Abstract	No limit
MEDLINE	AB (Antonovsky* OR Kohärenz* OR salutogen* OR “sense of coherence”) AND AB (religio* OR spiritu*) AND TX (association* OR correlation* OR Korrelation* OR Pearson* OR quantitativ* OR regression OR relation* OR Spearman*)	Facet A: abstract<br>Facet B: abstract<br>Facet C: all text	No limit
PubMed	(Antonovsky* OR Kohärenz* OR salutogen* OR “sense of coherence”) AND (religio* OR spiritu*) AND (association* OR correlation* OR Korrelation* OR Pearson* OR quantitativ* OR regression OR relation* OR Spearman*)	All fields	No limit
PSYINDEX	AB (Antonovsky* OR Kohärenz* OR salutogen* OR “sense of coherence”) AND AB (religio* OR spiritu*) AND TX (association* OR correlation* OR Korrelation* OR Pearson* OR quantitativ* OR regression OR relation* OR Spearman*)	Facet A: abstract<br>Facet B: abstract<br>Facet C: all text	No limit
SocINDEX	AB (Antonovsky* OR Kohärenz* OR salutogen* OR “sense of coherence”) AND AB (religio* OR spiritu*) AND TX (association* OR correlation* OR Korrelation* OR Pearson* OR quantitativ* OR regression OR relation* OR Spearman*)	Facet A: abstract<br>Facet B: abstract<br>Facet C: all text	No limit
Web of Science	((ALL=("sense of coherence" OR Antonovsky* OR Kohärenz* OR salutogen*)) AND ALL=(religio* OR spiritu*))	All fields	No limit

Note. * = Words match if they begin with the word preceding the asterisk; “ “ = A phrase that is double-quoted will only be found if it is word-for-word the same.
