# Supplementary material for: Sense of coherence and religion/spirituality: A systematic review and meta-analysis based on a methodical classification of instruments measuring religion/spirituality
Source: PLoS One. 2023 Aug 3;18(8):e0289203. doi: 10.1371/journal.pone.0289203 (PMC10399782; doi:10.1371/journal.pone.0289203)
Supplement: S3 Table — (PDF) [file pone.0289203.s007.pdf]

S5 Table. Rationale for the Inclusion and Exclusion Criteria.

No.	Criteria	Rationale
I1	The study must be written in English or German.	The language limitation of the studies is due to the language skills of the author team.
I2	The study must be published in a journal, as a book, as a book chapter or as a dissertation/habilitation thesis.	The criterion is used for quality assurance. Work that has been published as an article, book contribution or book is usually peer-reviewed. Conference papers or case reports do not count as articles and are therefore not included. Unpublished theses such as dissertations or post-doctoral theses are nevertheless included, as the review committee guarantees the scientific quality of the work. This does not apply to the same extent to lower qualification theses (bachelor's or master's thesis), which are therefore also not included.
I3	The study must use one of Antonovsky's original questionnaires (SOC-29 or SOC-13) to measure SOC quantitively.	The review will be limited to studies utilizing one of Antonovsky's original scales (SOC-29 or SOC-13) for two pragmatic reasons: (1) there is hardly a handful of studies using an alternative SOC scale to explore the R/S-SOC connection; (2) the inclusion of these studies would have diminished comparability of the results.
I4	The study must use at least one instrument/item to measure R/S.	Because the meta-analysis focuses on the correlation of R/S and SOC, only those studies that measure the variable R/S with at least one item can be included.
I5	The study must report at least one correlation coefficient between a R/S (sub-)scale and a SOC (sub-)scale.	The correlation coefficient between R/S and SOC was chosen as the effect size for the meta-analysis. Studies reporting effect sizes between R/S and at least one of the three SOC sub-scales can also be included, as this relationship is the subject of sub-analyses. Each eligible study, at the least, must specify sufficient data to calculate a correlation quotient by ourselves.
E1	The authors were unable to obtain the full text of the study (in time).	This criterion has solely research pragmatic background. Sometimes it is not possible to obtain the full text of a study within a reasonable period of time for the work process.
E2	The study results were reported elsewhere and that work met the above inclusion criteria.	This criterion prevents some data sets from being included more than once in the meta-analysis and thus distorting the results. As a rule, the later publication is excluded and the earlier one included.
E3	The sample size is less than or equal to ten.	If the sample of a study is too small, this weakens its validity. Nevertheless, we only automatically exclude studies with an extremely low number of cases, as sample quality is also part of our critical appraisal tool. The number 10 is merely an artificial limit that has proven useful from a research pragmatics point of view.

E4	The assessment of the study with our critical appraisal tool showed that the study was of limited suitability for the questions of this meta-analysis (“poor fit”).	Each study is assessed using our critical appraisal tool developed specifically for this meta-analysis. The tool assesses not so much the quality as the fit of the study. If a study is found not to fit our research question, it is excluded from the meta-analysis.
E5	The instrument/item used to assess R/S did not qualify as an R/S measure according to our classification.	Some measuring instruments do not measure R/S, although their designation suggests this. For this reason, this meta-analysis is based on a systematic item-specific qualitative content analysis of all putative R/S measures. If a study does not contain an R/S measure that qualified as a sufficient R/S measurement instrument according to our classification, the study will be excluded.

Note. The numbering of the criteria follows the listing in the text. Inclusion criteria are preceded by the letter I, exclusion criteria by the letter E.
