# Supplementary material for: Sense of coherence and religion/spirituality: A systematic review and meta-analysis based on a methodical classification of instruments measuring religion/spirituality
Source: PLoS One. 2023 Aug 3;18(8):e0289203. doi: 10.1371/journal.pone.0289203 (PMC10399782; doi:10.1371/journal.pone.0289203)
Supplement: S4 Table — Coding aid used to assign questionnaire items to the categories R, RS, RS/SR, SR, S, or X (item without religious/spiritual reference) in an intersubjectively comprehensible manner. (PDF) [file pone.0289203.s008.pdf]

S7 Table. Coding Aid for Classifying R/S Questionnaire Items.

Religion (R)	Rather religion but also spirituality (RS)	Equally religion and spirituality (RS/SR or SR/RS)	Rather spirituality but also religion (SR)	Spirituality (S)
religion, religious, religiously		religion/spirituality, religious/spiritual		spirituality, spiritual, spiritually
God, Heavenly Father, Allah, etc.	divine being		higher being, supreme being, greater being	higher power, transcendence, larger reality, higher plane, higher level
relationship to God, child of God				bond, connectedness, connection, interconnection
experience of God, religious experience	divine experience		mystical experience, mysticism, unity	spiritual experience, experience of transcendence
parental religiosity, religious socialization (upbringing, education)				search, seekers, quest
shared religious meaning-making framework	fate			personal meaning of life (mission, purpose, calling, ultimate goal)
organization, institution, church, religious community				individualism, non-organizational, non-institutionalized
“external”, material symbols (e.g., crucifix)				“internal”, inward, higher self, inner core
tradition, “static”, structure				development, growth, change
rules, norms, order, truth claims				openness, tolerance, eclecticism, choice
religious behavior, rules of conduct, behavior guidelines				holistic lifestyle
authority, religious experts (e.g., minister, pastoral worker)				spiritual self-sufficiency
private religious practice	prayer		meditation, contemplation	individual spiritual practice
church service, public worship, collective religious practice	ritual, ritualistic			spiritual events, loose group settings
Bible, sacred texts, canonical writings				spiritual literature

religious beliefs, doctrines, dogma, specific religious concepts (e.g., sin, sinner)	beliefs, believe, faith			spiritual beliefs, New Age or esoteric beliefs (e.g., aura, PSI powers, vital force)
religious thought, theological reflection, “rational”				magical thinking, “irrational”
specific religious persons, places or objects considered “holy”	holy, holiness, sacred, sacredness			entirety, wholeness, rather no clear distinction sacred vs. profane
hell, heaven, Nirvana, etc.		afterlife, life after death		spiritism, contact with the dead
morality, religious precepts, religious virtues, godly life	ethics	values		awareness, mindfulness, personal responsibility
creation (of God)				nature, universe, cosmos, greater world, all life, humanity
