# Supplementary material for: Sense of coherence and religion/spirituality: A systematic review and meta-analysis based on a methodical classification of instruments measuring religion/spirituality
Source: PLoS One. 2023 Aug 3;18(8):e0289203. doi: 10.1371/journal.pone.0289203 (PMC10399782; doi:10.1371/journal.pone.0289203)
Supplement: S7 Table — Overview of the first author’s personal communication and documentation of the decision whether to include studies after obtaining additional information. (PDF) [file pone.0289203.s011.pdf]

S12 Table. Personal Communication.

Study	Answer	Decision
Aderhold, C., Morawa, E., Paslakis, G., & Erim, Y. (2019). Entwicklung und Validierung eines Fragebogens zur Patientenkompetenz im Umgang mit einer Krebserkrankung (PUK). *Zeitschrift für Psychosomatische Medizin und Psychotherapie, 65*(3), 239-256. https://doi.org/https://doi.org/10.13109/zptm.2019.65.3.239	answer too late	nevertheless included
Akbari, T., & Ahadi Sagavaz, S. (2021). Predicting students' psychological well-being based on the components of spiritual intelligence and sense of coherence. *Journal of Modern Psychology, 1*(2), 40-49.	no answer	excluded
Anyfantakis, D., Symvoulakis, E. K., Panagiotakos, D. B., Tsetis, D., Castanas, E., Shea, S., . . . Lionis, C. (2013). Impact of religiosity/spirituality on biological and preclinical markers related to cardiovascular disease. Results from the SPILI III study. *Hormones*, *12*(3), 386-396.	no answer	excluded
Anyfantakis, D., Symvoulakis, E. K., Linardakis, M., Shea, S., Panagiotakos, D., & Lionis, C. (2015). Effect of religiosity/spirituality and sense of coherence on depression within a rural population in Greece: The Spili III project. *BMC Psychiatry, 15,* Article no 173. https://doi.org/10.1186/s12888-015-0561-3	no answer	excluded
Arévalo, S., Prado, G., & Amaro, H. (2008). Spirituality, sense of coherence, and coping responses in women receiving treatment for alcohol and drug addiction. *Evaluation & Program Planning, 31*(1), 113-123. https://doi.org/10.1016/j.evalprogplan.2007.05.009	no answer	excluded
Avaznejad, N., Ravanipour, M., Motamed, N., & Bahreini, M. (2017). Comparative study of the relationship between spiritual well-being and sense of coherence in mothers with chronically ill children in Kerman, Iran, in 2016. *Evidence Based Care, 7*(2), 78-83.	undelivered mail returned to sender	excluded
Braun-Lewensohn, O., & Sagy, S. (2010). Sense of coherence, hope and values among adolescents under missile attacks: A longitudinal study. *International Journal of Children's Spirituality, 15*(3). https://doi.org/10.1080/1364436X.2010.520305	negative (Pearson's *r* not provided)	excluded
Chamberlain, K., & Zika, S. (1988). Religiosity, Life meaning and wellbeing: Some relationships in a sample of women. *Journal for the Scientific Study of Religion, 27*(3), 411-420. https://doi.org/10.2307/1387379	negative (Pearson's *r* for total SOC not provided)	nevertheless included
Cowlishaw, S., Niele, S., Teshuva, K., Browning, C., & Kendig, H. (2013). Older adults' spirituality and life satisfaction: A longitudinal test of social support and sense of coherence as mediating mechanisms. *Ageing & Society, 33*(7), 1243-1262. https://doi.org/10.1017/S0144686X12000633	negative (Pearson's *r* not provided)	excluded
Dåderman, A. M., & De Colli, D. (2014). The significance of the sense of coherence for various coping resources in stress situations used by police officers in on-the-beat service. *International Journal of Occupational Medicine and Environmental Health, 27*(1), 3-15. https://doi.org/10.2478/s13382-014-0227-2	positive (Pearson's *r* provided)	included, later excluded

Study	Answer	Decision
Diaconescu, L. V., Gheorghe, I. R., Cheşcheş, T., & Popa-Velea, O. (2021). Psychological variables associated with HPV vaccination intent in Romanian academic aettings. *International Journal of Environmental Research and Public Health, 18(*17), 8938.	positive (Pearson's *r* provided)	included
Diržytė, A., Patapas, A., & Limantaitė, E. (2003). Religious beliefs, sense of coherence and well-being of Lithuanian students. *Socialinis Darbas, 1*(3), 15-22.	undelivered mail returned to sender	nevertheless included
Encarnação, P., Oliveira, C. C., & Martins, T. (2018). Psychometric properties of the suffering assessment questionnaire in adults with chronic diseases or life-threatening illness. *Scandinavian Journal of Caring Sciences, 32*(4), 1279-1287. https://doi.org/https://doi.org/10.1111/scs.12569	positive (Pearson's *r* provided)	included
Everson, R. B., Darling, C. A., & Herzog, J. R. (2013). Parenting stress among US Army spouses during combat-related deployments: the role of sense of coherence. *Child & Family Social Work, 18*(2), 168-178.	negative (Pearson's *r* not provided)	excluded
Ferreira, D. C., Gonçalves, T. R., Celeste, R. K., Olinto, M. T. A., & Pattussi, M. P. (2020). Psychosocial aspects and the impact of oral health on quality of life of Brazilian adults. *Brazilian Journal of Epidemiology, 23*, 1-13. https://doi.org/https://doi.org/10.1590/1980-549720200049	positive (Pearson's *r* provided)	included
Findler, L., Dayan-Sharabi, M., & Yaniv, I. (2014). The overlooked side of the experience: Personal growth and quality of life among grandparents of children who survived cancer. *Journal of Family Social Work, 17*(5), 418-437. https://doi.org/10.1080/10522158.2014.945675	negative (Pearson's *r* not provided)	excluded
Gabrielsen, L. E., Ulleberg, P., & Watten, R. G. (2012). The Adolescent Life Goal Profile Scale: Development of a new scale for measurements of life goals among young people. *Journal of Happiness Studies, 13*(6), 1053-1072. https://doi.org/https://doi.org/10.1007/s10902-011-9306-2	negative (Pearson's *r* for the clinical sample not provided)	nevertheless included
Gavulic, A. M. (2018). *Examining the effect of parental attachment, emotional maturity, spiritual maturity, and view of suffering on sense of coherence.* [Doctoral dissertation, Liberty University]. EBSCOhost Psyh Database.	positive (Pearson's *r* and R/S measure provided)	included
Gilhooly, M., Hanlon, P., Cullen, B., Macdonald, S., & Whyte, B. (2007). Successful ageing in an area of deprivation: part 2--a quantitative exploration of the role of personality and beliefs in good health in old age. *Public Health, 121*(11), 814-821.	no answer	excluded
Goulding, A. (2004). Schizotypy models in relation to subjective health and paranormal beliefs and experiences. *Personality and Individual Differences, 37*(1), 157-167.	positive (Pearson's *r* provided)	included
Goulding, A. (2005). Healthy schizotypy in a population of paranormal believers and experients. *Personality and Individual Differences, 38*(5), 1069-1083. https://doi.org/10.1016/j.paid.2004.07.006	positive (Pearson's *r* provided)	included

Study	Answer	Decision
Hammer, J. H., Cragun, R. T., & Hwang, K. (2013). Measuring spiritual fitness: Atheist military personnel, veterans, and civilians. *Military Psychology, 25*(5), 438-451. https://doi.org/https://doi.org/10.1037/mil0000010	negative (R/S measure not provided)	included
Khanjari, S., Oskouie, F., & Langius-Eklöf, A. (2012). Psychometric testing of the Caregiver Quality of Life Index-Cancer scale in an Iranian sample of family caregivers to newly diagnosed breast cancer women. *Journal of Clinical Nursing, 21*(3-4), 573-584.	negative (Pearson's *r* not provided)	excluded
Kimhi, S., & Eshel, Y. (2019). Measuring national resilience: A new short version of the scale (NR-13). *Journal of Community Psychology, 47*(3), 517-528. https://doi.org/10.1002/jcop.22135	negative (Pearson's *r* not provided)	excluded
Kimhi, S., Eshel, Y., Leykin, D., & Lahad, M. (2017). Individual, community, and national resilience in peace time and in the face of terror: A longitudinal study. *Journal of Loss & Trauma, 22*(8), 698-713. https://doi.org/10.1080/15325024.2017.1391943	Negative (Pearson's *r* not provided)	excluded
Kimhi, S., Eshel, Y., Lahad, M., & Leykin, D. (2019). National Resilience: A new self-report assessment scale. *Community Mental Health Journal, 55*(4), 721-731. https://doi.org/http://dx.doi.org/10.1007/s10597-018-0362-5	positive (Pearson's *r* provided)	included, but later excluded
Krok, D. (2016). Sense of coherence mediates the relationship between the religious meaning system and coping styles in Polish older adults. *Aging & Mental Health, 20*(10), 1002-1009. https://doi.org/https://doi.org/10.1080/13607863.2015.1056772	positive (R/S measure provided)	included
Lerner, M., & Lyvers, M. (2006). Values and beliefs of psychedelic drug users: A cross-cultural study. *Journal of Psychoactive Drugs, 38*(2), 143-147. https://doi.org/10.1080/02791072.2006.10399838	no answer	excluded
Lloyd, C. S., af Klinteberg, B., & DeMarinis, V. (2015). Psychological and existential vulnerability among clinical young women: a quantitative comparison of depression-related subgroups. *Mental Health, Religion & Culture, 18*(4), 259-272. https://doi.org/10.1080/13674676.2015.1021313	negative (Pearson's *r* not provided)	excluded
López, J., Camilli, C., & Noriega, C. (2015). Posttraumatic growth in widowed and non-widowed older adults: Religiosity and sense of coherence. *Journal of Religion and Health, 54*(5), 1612-1628. https://doi.org/10.1007/s10943-014-9876-5	positive (Pearson's *r* provided)	included
Marciano, H., Kimhi, S., & Eshel, Y. (2019). Predictors of individual, community and national resiliencies of Israeli Jews and Arabs. *International Journal of Psychology 55*(4), 553-561. https://doi.org/http://dx.doi.org/10.1002/ijop.12636	positive (Pearson's *r* provided)	included, but later excluded
Marsh, S. C., Clinkinbeard, S. S., Thomas, R. M., & Evans, W. P. (2007). Risk and protective factors predictive of sense of coherence during adolescence. *Journal of Health Psychology, 12*(2), 281-284.	undelivered mail returned to sender	excluded
Masters, K. S., & Knestel, A. (2011). Religious motivation and cardiovascular reactivity among middle aged adults: Is being pro-religious really that good for you? *Journal of Behavioral Medicine, 34*(6), 449-461. https://doi.org/10.1007/s10865-011-9352-6	no answer	excluded

Study	Answer	Decision
*Meghani, S. H., Peterson, C., Kaiser, D. H., Rhodes, J., Rao, H., Chittams, J., & Chatterjee, A. (2018). A pilot study of a mindfulness-based art therapy intervention in outpatients with cancer. *American Journal of Hospice & Palliative Medicine, 35*(9), 1195-1200. https://doi.org/https://doi.org/10.1177/1049909118760304	positive (Pearson's *r* provided)	included
Mowla, F., Khanjari, S., & Haghani, S. (2020). Effect of the combination of Benson's relaxation technique and brief psychoeducational intervention on religious coping, sense of coherence, and quality of life of family caregivers. *Journal of Education and Health Promotion, 9*(1), 7, Article 117. https://doi.org/10.4103/jehp.jehp_653_19	negative (Pearson's *r* not provided)	excluded
Nahlén, C., & Saboonchi, F. (2010). Coping, sense of coherence and the dimensions of affect in patients with chronic heart failure. *European Journal of Cardiovascular Nursing, 9*(2), 118-125.	positive (Pearson's *r* provided)	included
Oluwole, A. (2014). Spirituality and optimism as buffer of sense of coherence among imminent retirees in Lagos State, Nigeria. *African Journal for the Psychological Studies of Social Issues, 17*(1), 74-83.	no answer	excluded
Ozaki, M. (2005). Development of an assessment tool on spirituality explained by three domains, Will, joy and sense: From a holistic educational approach. *Journal of International Society of Life Information Science, 23*(2), 364-369.	negative (no information on test scores and SOC scale provided)	nevertheless included
Piedmont, R. L., Magyar-Russell, G., DiLella, N., & Matter, S. (2014). Sense of coherence: Big five correlates, spirituality, and incremental validity. *Current Issues in Personality Psychology, 2*(1), 1-9. https://doi.org/https://doi.org/10.5114/cipp.2014.43096	positive (R/S measure provided)	included
Pienaar, J. M., Beukes, R. B., & Esterhuyse, K. G. (2006). The relationship between conservatism and psychological well-being in adolescents. *South African Journal of Psychology, 36*(2), 391-406.		
Postolică, R., Enea, V., Dafinoiu, I., Petrov, I., & Azoicăi, D. (2019). Association of sense of coherence and supernatural beliefs with death anxiety and death depression among Romanian cancer patients. *Death Studies, 43*(1), 9-19. https://doi.org/10.1080/07481187.2018.1430083	no answer	excluded
Post-White, J., Ceronsky, C., Kreitzer, M. J., Nickelson, K., Drew, D., Mackey, K. W., . . . Gutknecht, S. (1996). Hope, spirituality, sense of coherence, and quality of life in patients with cancer. *Oncology Nursing Forum, 23*(10), 1571-1579.	positive (R/S measure and Pearson's *r* provided)	included
Punyoo, J., Pothiban, L., Jintrawet, U., Mesukko, J., & Reungrongrat, S. (2020). Factors associated with psychological well-being among parents of a critically ill child in pediatric intensive care unit. *Walailak Journal of Science & Technology, 17*(5), 437-449.	no answer	excluded

Study	Answer	Decision
Ragger, K., Hiebler-Ragger, M., Herzog, G., Kapfhammer, H.-P., & Unterrainer, H.-F. (2019). Sense of coherence is linked to post-traumatic growth after critical incidents in Austrian ambulance personnel. *BMC Psychiatry, 19*(89), 1-11. https://doi.org/https://doi.org/10.1186/s12888-019-2065-z	positive (Pearson's *r* provided)	included
Rigo, D. C. A., Ferreira, J. B. D. S., Costa, L. R., & Freire, M. D. C. M. (2022). Religiosity is associated with caregivers' perception of preschool children's dental health. *Brazilian Oral Research, 36*, e0121. https://doi.org/10.1590/1807-3107bor-2022.vol36.0121	no answer	excluded
Rohani, C., Khanjari, S., Abedi, H.-A., Oskouie, F., & Langius-Eklöf, A. (2010). Health index, sense of coherence scale, brief religious coping scale and spiritual perspective scale: Psychometric properties. *Journal of Advanced Nursing, 66*(12), 2796-2806. https://doi.org/https://doi.org/10.1111/j.1365-2648.2010.05409.x	undelivered mail returned to sender	excluded
Rokach, A., Findler, L., Chin, J., Lev, S., & Kollender, Y. (2013). Cancer patients, their caregivers and coping with loneliness. *Psychology, Health & Medicine, 18*(2), 135-144. https://doi.org/10.1080/13548506.2012.689839	negative (Pearson's *r* not provided)	excluded
Sadati, A. K., Salehzade, H., Hemmati, S., Darvish, M., Heydari, S. T., & Tabrizi, R. (2015). The causal factors associated with the loving care of the mothers of children with multiple disabilities. *International Journal of Community Based Nursing and Midwifery, 3*(4), 309.	no answer	excluded
Salehi, N., Afrashteh, M. Y., Majzoobi, M. R., Ziapour, A., Janjani, P., & Karami, S. (2022). Mediating role of pain self-efficacy in the relationship between sense of coherence, spiritual well-being and self-compassion with quality of life in Iranian elderly with cardiovascular disease. *Preprint*. https://doi.org/10.21203/rs.3.rs-2312272/v1	no answer	excluded
Sarenmalm, E. K., Browall, M., Persson, L. O., Fall-Dickson, J., & Gaston-Johansson, F. (2013). Relationship of sense of coherence to stressful events, coping strategies, health status, and quality of life in women with breast cancer. *Psycho-Oncology, 22*(1), 20-27. https://doi.org/10.1002/pon.2053	no answer	excluded
Siqueira, J., Fernandes, N. M., & Moreira-Almeida, A. (2019). Association between religiosity and happiness in patients with chronic kidney disease on hemodialysis. *Jornal Brasileiro de Nefrologia: 'Orgao Oficial de Sociedades Brasileira e Latino-Americana de Nefrologia, 41*(1), 22-28. https://doi.org/10.1590/2175-8239-JBN-2018-0096	no answer	excluded
Soares, T. R. C., Lenzi, M. M., Leite, I. M., Muniz Loureiro, J., Leão, A. T. T., Pomarico, L., . . . Maia, L. C. (2020). Oral status, sense of coherence, religious-spiritual coping, socio-economic characteristics, and quality of life in young patients. *International journal of Paediatric Dentistry, 30*(2), 171-180. https://doi.org/10.1111/ipd.12594	no answer	excluded
Stefanaki, I. N., Shea, S., Linardakis, M., Symvoulakis, E. K., Wynyard, R., & Lionis, C. (2014). Exploring the association of sense of coherence, and spiritual and religious beliefs in a rural population group on the Island of Crete, Greece. *International Journal of Psychiatry in Medicine, 47*(3), 207-230. https://doi.org/10.2190/PM.47.3.c	no answer	excluded
Suraj, S., & Singh, A. (2011). Study of sense of coherence health promoting behavior in north Indian students. *The Indian Journal of Medical Research, 134*(5), 645-652. https://doi.org/10.4103/0971-5916.90989	negative (Pearson's *r* not provided)	excluded

Study	Answer	Decision
Tagay, S., Senf, W., Schöpper, N., Mewes, R., Bockisch, A., & Görges, R. (2007). Protektive Faktoren für Angst und Depression bei Schilddrüsenkarzinompatienten. *Zeitschrift für Psychosomatische Medizin und Psychotherapie, 53*(1), 62-74.	no answer	excluded
Temane, Q. M., & Wissing, M. P. (2006). The role of spirituality as a mediator for psychological well-being across different contexts. *South African Journal of Psychology, 36*(3), 582-597. https://doi.org/10.1177/008124630603600309	no answer	excluded
Temane, L., Khumalo, I. P., & Wissing, M. P. (2014). Validation of the Meaning in Life Questionnaire in a South African context. *Journal of Psychology in Africa, 24*(1), 51-60.	no answer	excluded
Teut, M., Besch, F., Witt, C. M., & Stöckigt, B. (2019). Perceived outcomes of spiritual healing: Results from a prospective case series. *Complementary Medicine Research, 26*(4), 265-275.	positive (Pearson's *r* provided)	included, later excluded
Torinomi, C., Lindenberg, K., Möltner, A., Herpertz, S. C., & Holm-Hadulla, R. M. (2022). Predictors of Students' Mental Health during the COVID-19 Pandemic: The Impact of Coping Strategies, Sense of Coherence, and Social Support. *International Journal of Environmental Research and Public Health, 19*(24), 16423.	positive (Pearson's *r* provided)	included
Unterrainer, H.-F., Ladenhauf, K. H., Wallner-Liebmann, S. J., & Fink, A. (2011). Different types of religious/spiritual well-being in relation to personality and subjective well-being. *International Journal for the Psychology of Religion, 21*(2), 115-126. https://doi.org/10.1080/10508619.2011.557003	negative (Pearson's *r* not provided)	excluded
Uren, T. H., & Wastell, C. A. (2002). Attachment and meaning-making in perinatal bereavement. *Death Studies, 26*(4), 279-308. https://doi.org/https://doi.org/10.1080/074811802753594682	negative (R/S measure not provided)	nevertheless included
von Humboldt, S., Leal, I., & Pimenta, F. (2015). Sense of coherence, sociodemographic, lifestyle, and health-related factors in older adults' subjective well-being. *International Journal of Gerontology, 9*(1), 15-19.	positive (Pearson's *r* and single-item R/S measure provided)	included, but later excluded
Wissing, J. A. B., Wissing, M. P., du Toit, M. M., & Temane, Q. M. (2008). Psychometric properties of various scales measuring psychological well-being in a South African context: The FORT 1 Project. *Journal of Psychology in Africa, 18*(4), 511-520. https://doi.org/https://doi.org/10.1080/14330237.2008.10820230	positive (Pearson's *r* provided)	included
Zerach, G., & Levin, Y. (2018). Posttraumatic stress symptoms, burn-out, and compassion satisfaction among body handlers: The mediating role of sense of coherence and spirituality at workplace. *Journal of Interpersonal Violence, 33*(12), 1931-1957. https://doi.org/https://doi.org/10.1177/0886260515621065	positive (Pearson's *r* provided)	included
Ziarko, M., Mojs, E., Kaczmarek, L. D., Warchol-Biedermann, K., Malak, R., Lisinski, P., & Samborski, W. (2015). Do urban and rural residents living in Poland differ in their ways of copingwith chronic diseases? *European Review for Medical and Pharmacological Sciences, 19*(22), 4227-4234.	no answer	excluded

Note. R/S = religion/spirituality; SOC = sense of coherence.
