# Supplementary material for: Sense of coherence and religion/spirituality: A systematic review and meta-analysis based on a methodical classification of instruments measuring religion/spirituality
Source: PLoS One. 2023 Aug 3;18(8):e0289203. doi: 10.1371/journal.pone.0289203 (PMC10399782; doi:10.1371/journal.pone.0289203)
Supplement: S8 Table — List of all studies excluded from meta-analysis and indication of the respective exclusion criteria. (PDF) [file pone.0289203.s012.pdf]

S13 Table. List of Excluded Studies (*n* = 444 reports).

Number	Reference	Criteria
001	Adams, T. B. (1995). *The conceptualization and measurement of wellness*. [Doctoral dissertation, The University of Texas at Austin]. ProQuest Dissertations and Theses Global.	E5
002	Adams, T. B., Bezner, J. R., Drabbs, M. E., Zambarano, R. J., & Steinhardt, M. A. (2000). Conceptualization and measurement of the spiritual and psychological dimensions of wellness in a college population. *Journal of American College Health, 48*(4), 165-173. https://doi.org/10.1080/07448480009595692	E5
003	Aiko Bruce, A., Witol, A., Alvadj-Korenic, T., Mayan, M., Greenslade, H., Plaha, M., & Venner, M. A. (2018). A complex interface: Exploring sickle cell disease from a parent's perspective, after moving from Sub-Saharan Africa to North America". *Pediatric Hematology and Oncology*, *35(7-8)*, 373-384. https://doi.org/https://dx.doi.org/10.1080/08880018.2018.1541949	I3
004	Agarkov, V. A., Alexandrov, Y. I., Bronfman, S. A., Chernenko, A. M., Kapfhammer, H. P., & Unterrainer, H. F. (2018). A Russian adaptation of the Multidimensional Inventory for Religious/Spiritual Well-Being: Psychometric properties for young adults and associations with personality and psychiatric symptoms. *Archiv für Religionspsychologie, 40*(1), 104-115. https://doi.org/10.1163/15736121-12341347	I3, I5
005	Alivia, M., Guadagni, P., & Roberti di Sarsina, P. (2011). Towards salutogenesis in the development of personalised and preventive healthcare. *The EPMA journal, 2*(4), 381-384. https://doi.org/10.1007/s13167-011-0131-9	I3, I4, I5
006	Allart, P., Soubeyran, P., & Cousson-Gélie, F. (2013). Are psychosocial factors associated with quality of life in patients with haematological cancer? A critical review of the literature. *Psycho-Oncology, 22*(2), 241-249. https://doi.org/10.1002/pon.3026	I4, I5
007	Almedom, A. (2005). Resilience, hardiness, sense of coherence, and posttraumatic growth: All paths leading to "light at the end of the tunnel"? *Journal of Loss & Trauma, 10*(3), 253-265. https://doi.org/10.1080/15325020590928216	I3, I4, I5
008	Alon, R. (2022). Predicting typically-developing siblings? Acceptance of their sibling with ASD during emerging adulthood. *Research in Autism Spectrum Disorders, 99*, 12, Article 102065. https://doi.org/10.1016/j.rasd.2022.102065	I5
009	Anderson, C., & Potts, L. (2022). Physical health conditions of the Amish and intervening social mechanisms: An exhaustive narrative review. *Ethnicity & Health, 27*(8), 1952-1978. https://doi.org/https://dx.doi.org/10.1080/13557858.2021.1968351	I4, I5
010	Anderson, M. R. (2020). The spiritual heart. *Religions, 11*(10), 506-506. https://doi.org/10.3390/rel11100506	I4, I5
011	Andersson, N., & Ledogar, R. J. (2008). The CIET Aboriginal youth resilience studies: 14 Years of capacity building and methods development in Canada. *Pimatisiwin, 6*(2), 65-88.	I4, I5
012	Andreatta, M.-P. (2004). Die Erschütterung des Selbst- und Weltverständnisses durch primäre und sekundäre Traumatisierung: Auswirkungen von Traumaexposition auf kognitive Schemata [Doctoral dissertation, Universität Innsbruck].	I4
013	Anson, O., Carmel, S., Levenson, A., Bonneh, D. Y., & Maoz, B. (1993). Coping with recent life events: The interplay of personal and collective resources. *Behavioral Medicine, 18*(4), 159-166.	I4, I5
014	Anyan, F., & Knizek, B. L. (2018). The coping mechanisms and strategies of hypertension patients in Ghana: The role of religious faith,	I3, I4, I5

Number	Reference	Criteria
	beliefs and practices. *Journal of Religion and Health, 57*(4), 1402-1412. https://doi.org/10.1007/s10943-017-0517-7	
015	Anyfantakis, D., Symvoulakis, E. K., Linardakis, M., Shea, S., Panagiotakos, D., & Lionis, C. (2015). Effect of religiosity/spirituality and sense of coherence on depression within a rural population in Greece: The Spili III project. *BMC Psychiatry, 15*. https://doi.org/10.1186/s12888-015-0561-3	I5
016	Anyfantakis, D., Symvoulakis, E. K., Panagiotakos, D. B., Tsetis, D., Castanas, E., Shea, S., . . . Lionis, C. (2013). Impact of religiosity/spirituality on biological and preclinical markers related to cardiovascular disease. Results from the SPILI III study. *Hormones, 12*(3), 386-396.	I5
017	Arens, C. R., White, T. L., & Massengill, N. (2014). Attitudinal factors protective against youth smoking: An integrative review. *Journal of Nursing Scholarship, 46*(3), 167-175. https://doi.org/10.1111/jnu.12065	I3, I4, I5
018	Arévalo, S., Prado, G., & Amaro, H. (2008). Spirituality, sense of coherence, and coping responses in women receiving treatment for alcohol and drug addiction. *Evaluation & Program Planning, 31*(1), 113-123. https://doi.org/10.1016/j.evalprogplan.2007.05.009	I5
019	Arnold, R. W. (2007). *Sense of coherence, spiritual maturity, and psychological well-being among united methodist clergy.* [Doctoral dissertation, University of Florida]. ProQuest Dissertations and Theses Global.	I5
020	Artinian, B. M. (1991). The development of the Intersystem Model. *Journal of Advanced Nursing, 16*(2), 194-205.	I3, I4, I5
021	Arvidsdotter, T., Marklund, B., Taft, C., & Kylén, S. (2015). Quality of life, sense of coherence and experiences with three different treatments in patients with psychological distress in primary care: A mixed-methods study. *BMC Complementary and Alternative Medicine, 15*, 132. https://doi.org/10.1186/s12906-015-0654-z	I4, I5
022	Astedt-Kurki, P., Friedemann, M. L., Paavilainen, E., Tammentie, T., & Paunonen-Ilmonen, M. (2001). Assessment of strategies in families tested by Finnish families. *International Journal of Nursing Studies, 38*(1), 17-24.	I3, I4, I5
023	Atwell, R., Gifford, S. M., & McDonald-Wilmsen, B. (2009). Resettled refugee families and their children's futures: Coherence, hope and support. *Journal of Comparative Family Studies, 40*(5), 677-697. https://doi.org/10.3138/jcfs.40.5.677	I3, I4, I5
024	Avaznejad, N., Ravanipour, M., Motamed, N., & Bahreini, M. (2017). Comparative study of the relationship between spiritual well-being and sense of coherence in mothers with chronically ill children in Kerman, Iran, in 2016. *Journal of Evidence-based Care, 7*(2), 78-83. https://doi.org/10.22038/EBCJ.2017.22985.1500	I5
025	Azevedo, A. (2020). A new theoretical framework for therapeutic landscapes: Coastal (blue), forest (green), spiritual “power spots” (gold) and wildernerss (dark/white). *Journal of Spatial and Organizational Dynamics, 8*(1), 29-51.	I3, I4, I5
026	Baker, S. J. (2010). Bedside shift report improves patient safety and nurse accountability. *Journal of Emergency Nursing, 36*(4), 355-358.	I3, I4, I5
027	Bauer, R., Sterzinger, L., Koepke, F., & Spiessl, H. (2013). Rewards of caregiving and coping strategies of caregivers of patients with mental illness. *Psychiatric Services, 64*(2), 185-188. https://doi.org/10.1176/appi.ps.001212012	I5, I3
028	Bell, I. R., Lewis, I. D. A., Lewis, S. E., Brooks, A. J., Schwartz, G. E., & Baldwin, C. M. (2004). Strength of vital force in classical homeopathy: Bio-psycho-social-spiritual correlates within a complex systems context. *Journal of Alternative and Complementary Medicine, 10*(1), 123-131. https://doi.org/http://dx.doi.org/10.1089/107555304322849048	I5

Number	Reference	Criteria
029	Bennett, C. C. (1993). *Factors which impact coping and health outcome of significant others of persons with AIDS*. [Doctoral dissertation, University of California].	I4
030	Bhattacharya, S., Pradhan, K. B., Bashar, M. A., Tripathi, S., Thiyagarajan, A., Srivastava, A., & Singh, A. (2020). Salutogenesis: A bona fide guide towards health preservation. *Journal of Family Medicine and Primary Care, 9*(1), 16-19. https://doi.org/10.4103/jfmpc.jfmpc_260_19	I3, I4, I5
031	Bias, E. S. (1998). Mediating the stress-outcome relationship in Alzheimer's caregiving: The reciprocal influences of Sense of Coherence, coping, and boundary ambiguity. [Doctoral dissertation, The California School of Professional Psychology at Alameda]. ProQuest Dissertations and Theses Global.	I3, I4, I5
032	Bjarnason, T. (1998). Parents, religion and perceived social coherence: A Durkheimian framework of adolescent anomie. *Journal for the Scientific Study of Religion, 37*(4), 742-754. https://doi.org/10.2307/1388154	I3, I5
033	Bjørkløf, G. H., Engedal, K., Selbaek, G., Kouwenhoven, S. E., & Helvik, A. S. (2013). Coping and depression in old age: A literature review. *Dementia and Geriatric Cognitive Disorders, 35*(3-4), 121-154. https://doi.org/http://dx.doi.org/10.1159/000346633	I3, I4, I5
034	Błażek, M., & Besta, T. (2012). Self-concept clarity and religious orientations: Prediction of purpose in life and self-esteem. *Journal of Religion and Health, 51*(3), 947-960. https://doi.org/10.1007/s10943-010-9407-y	I3, I5
035	Blume, N. (1999). *'For better or worse': Exploration of positive appraisal in the Alzheimer's caregiving situation.* [Doctoral dissertation, University of Kansas]. ProQuest Dissertations and Theses Global.	I5
036	Bonmatí-Tomás, A., Malagón-Aguilera Mdel, C., Bosch-Farré, C., Gelabert-Vilella, S., Juvinyà-Canal, D., & Garcia Gil Mdel, M. (2016). Reducing health inequities affecting immigrant women: a qualitative study of their available assets. *Global Health, 12*(1), 37. https://doi.org/10.1186/s12992-016-0174-8	I3, I4, I5
037	Bontempo, A. M. (2004). Making meaning out of mayhem: A grounded theory of resiliency, relationship, and women coping with breast cancer. [Doctoral dissertation, James Madison University]. ProQuest Dissertations and Theses Global.	I3, I4, I5
038	Borwick, S., Schweitzer, R. D., Brough, M., Vromans, L., & Shakespeare-Finch, J. (2013). Well-being of refugees from Burma: A salutogenic perspective. *International Migration, 51*(5), 91-105. https://doi.org/10.1111/imig.12051	I3, I4, I5
039	Boscaglia, N., & Clarke, D. M. (2007). Sense of coherence as a protective factor for demoralisation in women with a recent diagnosis of gynaecological cancer. *Psycho-Oncology, 16*, 189-195. https://doi.org/10.1002/pon.1044	I3, I4, I5
040	Botha, K. F. H., Du Plessis, W. F., Van Rooyen, J. M., & Wissing, M. P. (2002). Biopsychosocial determinants of self-management in culturally diverse South african patients with essential hypertension. *Journal of health psychology, 7*(5), 519-531. https://doi.org/10.1177/1359105302007005672	I4, I5
041	Bowman, B. J. (1995). *Differences in the development and expression of a sense of coherence between Euro Americans and native Americans.* [Doctoral dissertation, University of North Dakota]. ProQuest Dissertations and Theses Global.	I5
042	Bowman, B. J. (1997). Cultural pathways toward Antonovsky's sense of coherence. *Journal of Clinical Psychology, 53*(2), 139-142. https://doi.org/http://dx.doi.org/10.1002/%28SICI%291097-4679%28199702%2953:2%3C139::AID-JCLP7%3E3.0.CO;2-O	I5

Number	Reference	Criteria
043	Braun-Lewensohn, O. (2016). Sense of Coherence, values, youth involvement, civic efficacy and hope: Adolescents during social protest. *Social Indicators Research, 128*(2), 661-673. https://doi.org/10.1007/s11205-015-1049-8	I3, I4, I5
044	Braun-Lewensohn, O., & Sagy, S. (2010). Sense of Coherence, hope and values among adolescents under missile attacks: A longitudinal study. *International Journal of Children's Spirituality, 15*(3), 247-260. https://doi.org/10.1080/1364436X.2010.520305	I5
045	Braun-Lewensohn, O., Abu-Kaf, S., & Kalagy, T. (2017). Are "Sense of Coherence" and "Hope" related constructs? Examining these concepts in three cultural groups in Israel. *The Israel Journal of Psychiatry and Related Sciences, 54*(2), 17-23.	I3, I4, I5
046	Braun-Lewensohn, O., Sagy, S., Sabato, H., & Galili, R. (2013). Sense of coherence and sense of community as coping resources of religious adolescents before and after the disengagement from the Gaza Strip. *Israel Journal of Psychiatry and Related Sciences, 50*(2), 110-117.	I4, I5
047	Braun-Lewensohn, O., & Kalagy, T. (2019). Between the inside and the outside world: Coping of ultra-orthodox individuals with their work environment after academic studies. *Community Mental Health Journal, 55*(5), 894-905. https://doi.org/10.1007/s10597-019-00392-x	I3
048	Bringmann, H. C., Bringmann, N., Jeitler, M., Brunnhuber, S., Michalsen, A., & Sedlmeier, P. (2021). Meditationsbasierte Lebensstilmodifikation (MBLM) bei ambulanten Patienten mit leichter bis mittelschwerer Depression: Eine Mixed-Methods-Machbarkeitsstudie [Meditation Based Lifestyle Modification (MBLM) in outpatients with mild to moderate depression: A mixed-methods feasibility study]. *Complementary Therapies in Medicine, 56*, 102598. https://doi.org/10.1016/j.ctim.2020.102598	I3
049	Britt, T. W., Millard, M. R., Sundareswaran, P. T., & Moore, D. (2009). Personality variables predict strength-related attitude dimensions across objects. *Journal of Personality, 77*(3), 859-882. https://doi.org/10.1111/j.1467-6494.2009.00567.x	E5
050	Bronikowski, M., Laudanska-Krzeminska, I., Tomczak, M., & Morina, B. (2016). Sense of coherence, physical activity and its associations with gender and age among Kosovar adolescents: A cross-sectional study. *Journal of Sports Medicine & Physical Fitness, 57*(7-8), 1023-1032. https://doi.org/10.23736/S0022-4707.16.06394-5	I4, I5
051	Brook, U. (2006). Psychosocial impact and personality predictors of coherence feeling among girls with acne. *The Indian Journal of Pediatrics, 73*(3), 213-215.	I4, I5
052	Büssing, A., Frick, E., Jacobs, C., & Baumann, K. (2016). Spiritual dryness in non-ordained Catholic pastoral workers. *Religions, 7*(12), 9, Article 141. https://doi.org/10.3390/rel7120141	E2
053	Carlsson, I.-M., Ziegert, K., & Nissen, E. (2015). The relationship between childbirth self-efficacy and aspects of well-being, birth interventions and birth outcomes. *Midwifery, 31*(10), 1000-1007. https://doi.org/10.1016/j.midw.2015.05.005	I4, I5
054	Cassel, L., & Suedfeld, P. (2006). Salutogenesis and autobiographical disclosure among Holocaust survivors. *The Journal of Positive Psychology, 1*(4), 212-225. https://doi.org/https://doi.org/10.1080/17439760600952919	E4
055	Cederblad, M., Dahlin, L., Hagnell, O., & Hansson, K. (1995). Coping with life span crises in a group at risk of mental and behavioral disorders: From the Lundby study. *Acta Psychiatrica Scandinavica, 91*(5), 322-330.	I4, I5
056	Chase, M. W. (2001). Spirituality as a salutogenic factor in African American adolescents: Understanding the relationships among religion,	I3, I5

Number	Reference	Criteria
	health, and well-being. [Doctoral dissertation, California School of Professional Psychology]. ProQuest Dissertations and Theses Global.	
057	Chawak, S., Chittem, M., S, A., Varghese, D., & Epton, T. (2020). Predictors of health behaviours among Indian college students: an exploratory study. *Health Education, 120*(2), 179-195. https://doi.org/10.1108/HE-11-2019-0049	I4, I5
058	Chen, G. (2006b). Social support, spiritual program, and addiction recovery. *International Journal of Offender Therapy and Comparative Criminology, 50*(3), 306-323. https://doi.org/10.1177/0306624X05279038	I3, I4, I5
059	Cheng, Y.-C. (2006). Caregiver burnout: A critical review of the literature. [Doctoral dissertation, Alliant International University San Diego]. ProQuest Dissertations and Theses Global.	I3, I4, I5
060	Cilliers, F., & Terblanche, L. (2014). The role of spirituality in coping with the demands of the hospital culture amongst fourth-year nursing students. *International Review of Psychiatry, 26*(3), 279-288. https://doi.org/10.3109/09540261.2014.890922	I3, I5
061	Cobb, R. K. (2012). How well does spirituality predict health status in adults living with HIV-disease: A Neuman systems model study. *Nursing Science Quarterly, 25*(4), 347-355. https://doi.org/10.1177/0894318412457051	I5
062	Cofini, V., Cecilia, M. R., Petrarca, F., Bernardi, R., Mazza, M., & Di Orio, R. (2014). Factors associated with post-traumatic growth after the loss of a loved. *Minerva Psichiatrica, 55*(4), 207-214.	I3, I5
063	Cohen, O. (1997). On the origins of a sense of coherence: Sociodemographic characteristics, or narcissism as a personality trait. *Social Behavior and Personality, 25*(1), 49-57. https://doi.org/http://doi.org/10.2224/sbp.1997.25.1.49	E4
064	Cohen, O., & Dekel, R. (2000). Sense of coherence, ways of coping, and well being of married and divorced mothers. *Contemporary Family Therapy: An International Journal, 22*(4), 467. https://doi.org/10.1023/A:1007853002549	I3, I5
065	Conway-Phillips, R. (2011). A salutogenic framework to understand disparity in breast cancer screening behavior in African American women [Doctoral dissertation, Loyola University Chicago].	E2
066	Costin, V., & Vignoles, V. L. (2022). What do people find most meaningful? How representations of the self and the world provide meaning in life. *Journal of Personality, 90*(4), 541-558. https://doi.org/10.1111/jopy.12682	I3, I4, I5
067	Cowlishaw, S., Niele, S., Teshuva, K., Browning, C., & Kendig, H. (2013). Older adults' spirituality and life satisfaction: A longitudinal test of social support and sense of coherence as mediating mechanisms. *Ageing & Society, 33*(7), 1243-1262. https://doi.org/10.1017/S0144686X12000633	I5
068	Crawford, M. R., & Holder, M. D. (2013). Promoting happiness through urban planning. In F. Sarracino (Ed.), *The happiness compass: Theories, actions and perspectives for well-being*. (pp. 177-201). Nova Science Publishers.	I3, I4, I5
069	Crego, A., Yela, J. R., Gomez-Martinez, M. A., & Karim, A. A. (2020). The contribution of meaningfulness and mindfulness to psychological well-being and mental health: A structural equation model. *Journal of Happiness Studies, 21*(8), 2827-2850. https://doi.org/10.1007/s10902-019-00201-y	I3, I5
070	Crespi, B., Dinsdale, N., Read, S., & Hurd, P. (2019). Spirituality, dimensional autism, and schizotypal traits: The search for meaning. *Plos One, 14*(3), Article e0213456. https://doi.org/https://dx.doi.org/10.1371/journal.pone.0213456	I3, I5

Number	Reference	Criteria
071	Crowther, S., & Lau, A. (2019). Migrant Polish women overcoming communication challenges in Scottish maternity services: A qualitative descriptive study. *Midwifery, 72*, 30-38. https://doi.org/10.1016/j.midw.2019.02.004	I3, I4, I5
072	Czekóová, K., Shaw, D. J., & Urbánek, T. (2018). Personality systems, spirituality, and existential well-being: A person-centered perspective. *Psychology of Religion and Spirituality, 10*(4), 307-317. https://doi.org/10.1037/rel0000109	I3, I5
073	Dåderman, A. M., & De Colli, D. (2014). The significance of the sense of coherence for various coping resources in stress situations used by police officers in on-the-beat service. *International Journal of Occupational Medicine and Environmental Health, 27*(1), 3-15. https://doi.org/10.2478/s13382-014-0227-2	I5, E5
074	Dahlberg, U., Persen, J., Skogås, A.-K., Selboe, S.-T., Torvik, H. M., & Aune, I. (2016). How can midwives promote a normal birth and a positive birth experience? The experience of first-time Norwegian mothers. *Sexual & Reproductive Healthcare, 7*, 2-7. https://doi.org/10.1016/j.srhc.2015.08.001	I3, I5
075	Darling, C. A., Hill, E. W., & McWey, L. M. (2004). Understanding stress and quality of life for clergy and clergy spouses. *Stress and health, 20*(5), 261-277.	E5
076	De La Paz, M. (2004). The role of spirituality in how Filipino immigrants conceptualize and cope with crisis. [Doctoral dissertation, Alliant International University, San Francisco Bay]. ProQuest Dissertations and Theses Global.	I3, I4, I5
077	De Siqueira, S. R. D. T. (2018). Existential meaning of patients with chronic facial pain. *Journal of Religion and Health, 57*(3), 1125-1132. https://doi.org/10.1007/s10943-018-0583-5	I3, I5
078	Delgado, C. L. (2006). The impact of sense of coherence and spirituality on perceived stress and quality of life in persons with chronic obstructive pulmonary disease. [Doctoral dissertation, Loyola University Chicago]. ProQuest Dissertations and Theses Global.	E2
079	Demmrich, S., & Akgul, S. (2020). Bullying experience among adolescents with a Turkish migration background in Germany: Ethnic class composition, integration, and religiosity as protective factors?. *International Journal of Environmental Research and Public Health, 17*(13), 16, Article 4776. https://doi.org/10.3390/ijerph17134776	I3
080	Dengah, H. J. F., 2nd, Bingham Thomas, E., Hawvermale, E., & Temple, E. (2019). "Find that Balance:" The impact of cultural consonance and dissonance on mental health among Utah and Mormon women. *Medical Anthropology Quarterly, 33*(3), 439-458. https://doi.org/10.1111/maq.12527	I3, I4, I5
081	Diehl, V. (2009). The bridge between patient and doctor: The shift from CAM to integrative medicine. *Hematology.,1*, 320-325. https://doi.org/10.1182/asheducation-2009.1.320	I3, I4, I5
082	Dilani, A. (2001). Psychosocially supportive design--Scandinavian health care design. *World Hospitals and Health Services, 37*(1), 20.	I3, I4, I5
083	Du Toit, B. M. (1977). Historical and cultural factors influencing Cannabis use among Indians in South Africa. *Journal of Psychedelic Drugs, 9*(3), 235-246.	I3
084	Edwards, S. D. (2014). Evaluation of heart rhythm coherence feedback training on physiological and psychological variables. *South African Journal of Psychology, 44*(1), 73-82. https://doi.org/10.1177/0081246313516255	I5, I3
085	Edwards, S. D., & Edwards, D. J. (2017). Contemplative investigation into Christ consciousness with Heart Prayer and HeartMath practices.	I3

Number	Reference	Criteria
	Hts Teologiese Studies-Theological Studies, 73(3), 5, Article 4537. https://doi.org/10.4102/hts.v73i3.4537	
086	Eli, K., Sorjonen, K., Mokoena, L., Pietrobelli, A., Flodmark, C.-E., Faith, M. S., & Nowicka, P. (2016). Associations between maternal sense of coherence and controlling feeding practices: The importance of resilience and support in families of preschoolers. *Appetite, 105*, 134-143. https://doi.org/10.1016/j.appet.2016.05.012	I4, I5
087	Elliott, M., & Hayward, R. D. (2007). Religion and well-being in a church without a creed. *Mental Health, Religion and Culture, 10*(2), 109-126. https://doi.org/https://dx.doi.org/10.1080/13694670500386069	I3
088	El-Shahawy, O., Sun, P., Tsai, J. Y.-K., Rohrbach, L. A., & Sussman, S. (2015). Sense of coherence and tobacco use myths among adolescents as predictors of at-risk youth cigarette use. *Substance Use & Misuse, 50*(1), 8-14. https://doi.org/10.3109/10826084.2014.957767	I4, I5
089	Encarnacao, P., Oliveira, C. C., & Martins, T. (2017). A generalized resistance resource: faith. A nursing view. *Health Promotion International, 32*(3), 577-580. https://doi.org/10.1093/heapro/dav114	I3, I4, I5
090	Esch, T., & Stefano, G. B. (2004). The neurobiology of pleasure, reward processes, addiction and their health implications. *Neuro Endocrinology Letters, 25*(4), 235-251.	I3, I4, I5
091	Everson, R. B., Darling, C. A., & Herzog, J. R. (2013). Parenting stress among US Army spouses during combat-related deployments: the role of sense of coherence. *Child & Family Social Work, 18*(2), 168-178. https://doi.org/10.1111/j.1365-2206.2011.00818.x	I5
092	Everson, R. B., Darling, C., Herzog, J. R., Figley, C. R., & King, D. (2017). Quality of life among U.S. Army spouses during the Iraq war. *Journal of Family Social Work, 20*(2), 124-143. https://doi.org/10.1080/10522158.2017.1279578	I4, I5
093	Eychmueller, S. (2009). Management of depression in the last month of life. *Current Opinion in Supportive and Palliative Care, 3*(3), 186-189. https://doi.org/10.1097/SPC.0b013e32832f00d7	I3, I4, I5
094	Falkenberg, T. (2014). Spiritual phenomena as public goods: Exploring meditation beyond the standard model. In S. Schmidt & H. Walach (Eds.), *Meditation — Neuroscientific approaches and philosophical implications*. (Vol. 2, pp. 261-270). Springer International Publishing.	I3, I4, I5
095	Faull, K., & Hills, M. D. (2007). A spiritually-based measure of holistic health for those with disabilities: Development, preliminary reliability and validity assessment. *Disability and Rehabilitation: An International, Multidisciplinary Journal, 29*(13), 999-1010. https://doi.org/10.1080/09638280600926637	E5
096	Feingold, J. H., Hurtado, A., Feder, A., Peccoralo, L., Southwick, S. M., Ripp, J., & Pietrzak, R. H. (2022). Posttraumatic growth among health care workers on the frontlines of the COVID-19 pandemic. *Journal of Affective Disorders, 296*, 35-40. https://doi.org/10.1016/j.jad.2021.09.032	I3, I5
097	Fenton, C., Brooks, F., Spencer, N. H., & Morgan, A. (2010). Sustaining a positive body image in adolescence: An assets-based analysis. *Health & Social Care in the Community, 18*(2), 189-198. https://doi.org/10.1111/j.1365-2524.2009.00888.x	I4, I5
098	Fernandez-Martinez, E., Lopez-Alonso, A. I., Marques-Sanchez, P., Martinez-Fernandez, M. C., Sanchez-Valdeon, L., & Liebana-Presa, C. (2019). Emotional intelligence, sense of coherence, engagement and coping: A cross-sectional study of university students'	I5

Number	Reference	Criteria
	health. *Sustainability, 11*(24), Article 6953. https://doi.org/10.3390/su11246953	
099	Fernros, L. (2022). *Improving quality of life using mind-body therapies: The evaluation of a course intervention for personal self-awareness and development* [Doctoral dissertation, Karolinska Institutet]. ProQuest Dissertations and Theses Global.	I4, I5
100	Findl, H. (2012). *Religiosität und Spiritualität in psychotherapeutischen Behandlungen: Erfahrungen, Einstellungen und (be)handlungsleitende Theorien von PsychotherapeutInnen* [Doctoral dissertation].	I3, I4, I5
101	Frick, E., Baumann, K., Büssing, A., Jacobs, C., & Sautermeister, J. (2018). Spirituelle Trockenheit - Krise oder Chance? Am Beispiel der römisch-katholischen Priesterausbildung. *Wege zum Menschen, 70*(1), 61-77.	I3, I4, I5, E2
102	Fries, C. J. (2020). Healing health care: From sick care towards salutogenic healing systems. *Social Theory & Health, 18*(1), 16-32. https://doi.org/10.1057/s41285-019-00103-2	I3, I4, I5
103	Gaston-Johansson, F., Haisfield-Wolfe, M. E., Reddick, B., Goldstein, N., & Lawal, T. A. (2013). The relationships among coping strategies, religious coping, and spirituality in African American women with breast cancer receiving chemotherapy. *Oncology Nursing Forum, 40*(2), 120-131. https://doi.org/10.1188/13.ONF.120-131	I5
104	Généreux, M., Schluter, P. J., Hung, K. K., …, & Roy, M. (2020). One virus, four continents, eight countries: An interdisciplinary and international study on the psychosocial impacts of the COVID-19 pandemic among adults. *International Journal of Environmental Research on Public Health, 17*(22). https://doi.org/10.3390/ijerph17228390	I3, I4, I5
105	Généreux, M., Schluter, P. J., Landaverde, E., …, & Roy, M. (2021). The evolution in anxiety and depression with the progression of the pandemic in adult populations from eight countries and four Continents. *International Journal of Environmental Research on Public Health*, *18*(9). https://doi.org/10.3390/ijerph18094845	I3, I4, I5
106	George, L. K., Ellison, C. G., & Larson, D. B. (2002). Explaining the relationships between religious involvement and health. *Psychological Inquiry, 13*(3), 190-200. https://doi.org/10.1207/S15327965PLI1303_04	I3, I4, I5
107	George, L. K., Larson, D. B., Koenig, H. G., & McCullough, M. E. (2000). Spirituality and health: What we know, what we need to know. *Journal of Social and Clinical Psychology, 19*(1), 102-116. https://doi.org/10.1521/jscp.2000.19.1.102	I3, I4, I5
108	Ghafoori, B., Hierholzer, R. W., Howsepian, B., & Boardman, A. (2008). The role of adult attachment, parental bonding, and spiritual love in the adjustment to military trauma. *Journal of Trauma & Dissociation, 9*(1), 85-106. https://doi.org/10.1080/15299730802073726	I5, I3
109	Gilbar, O. (1998). Relationship between burnout and sense of coherence in health social workers. *Social Work in Health Care, 26*(3), 39-49.	I4, I5
110	Gilbar, O. (2003). Do attitude toward cancer, sense of coherence and family high risk predict more psychological distress in women referred for a breast cancer examination? *Women & Health, 38*(2), 35-46.	I4, I5
111	Gilhooly, M., Hanlon, P., Cullen, B., Macdonald, S., & Whyte, B. (2007). Successful ageing in an area of deprivation: part 2--a quantitative exploration of the role of personality and beliefs in good health in old age. *Public Health, 121*(11), 814-821.	I5
112	Giordano, J., & Engebretson, J. (2006). Neural and cognitive basis of spiritual experience: Biopsychosocial and ethical implications for	I3, I4, I5

Number	Reference	Criteria
	clinical medicine. *Explore, 2*(3), 216-225. https://doi.org/10.1016/j.explore.2006.02.002	
113	Glandon, D. M., Muller, J., & Almedom, A. M. (2008). Resilience in post-Katrina New Orleans, Louisiana: A preliminary study. *African Health Sciences, 8*(Special Edition), S21-S27.	I4, I5
114	Głaz, S. (2019). The relationship of forgiveness and values with meaning in life of Polish students. *Journal of Religion and Health, 58*(5), 1886-1907. https://doi.org/10.1007/s10943-019-00860-4	I3, I5
115	Gliko, I., & Braun-Lewensohn, O. (2022). Teachers' coping with integrated classes that include special-needs children: A comparison between teachers in different education sectors. *Mifgash: Journal of Social Educational Work, 30*(54), IV.	E1
116	Goetzmann, L., Wagner-Huber, R., Klaghofer, R., Muellhaupt, B., Clavien, P. A., Buddeberg, C., & Scheuer, E. (2006). Waiting for a liver transplant: Psychosocial well-being, spirituality, and need for counselling. *Transplantation Proceedings, 38*(9), 2931-2936. https://doi.org/http://dx.doi.org/10.1016/j.transproceed.2006.08.171	I4, I5
117	Gomes, A. C., Rebelo, M. A. B., de Queiroz, A. C., de Queiroz Herkrath, A. P. C., Herkrath, F. J., Rebelo Vieira, J. M., Pereira, J. V., & Vettore, M. V. (2020). Socioeconomic status, social support, oral health beliefs, psychosocial factors, health behaviours and health-related quality of life in adolescents. *Quality of Life Research, 29*(1), 141-151. https://doi.org/10.1007/s11136-019-02279-6	I4, I5
118	Götze, H., Brähler, E., Gansera, L., Schnabel, A., & Köhler, N. (2015). Erschöpfung und Überlastung pflegender Angehöriger von Krebspatienten in der palliativen Situation. [Exhaustion and overload of caring relatives of cancer patients in the palliative situation]. *Psychotherapie, Psychosomatik, Medizinische Psychologie, 65*(2), 66-72. https://doi.org/10.1055/s-0034-1385933	I4, I5
119	Gowan, M. E., Kirk, R. C., & Sloan, J. A. (2014). Building resiliency: A cross-sectional study examining relationships among health-related quality of life, well-being, and disaster preparedness. *Health and Quality of Life Outcomes, 12*(1), Article 85. https://doi.org/http://dx.doi.org/10.1186/1477-7525-12-85	I3, I5
120	Graham, K. (1998). Alcohol abstention among older adults: reasons for abstaining and characteristics of abstainers. *Addiction Research, 6*(6), 473-487.	I5
121	Grassi, L. (2012). Quam bene vivas refert: Curing and caring in psycho-oncology. *Asia-Pacific Journal of Clinical Oncology, 8*, 215. https://doi.org/http://dx.doi.org/10.1111/ajco.12029	E1
122	Greeff, A. P., & Van Der Merwe, S. (2004). Variables associated with resilience in divorced families. *Social Indicators Research, 68*(1), 59-75. https://doi.org/10.1023/B:SOCI.0000025569.95499.b5	I3
123	Gregersen, A. M., & Afdal, G. (2022). An affective religious boundary tool. *Poetics, 93*, 11, Article 101685. https://doi.org/10.1016/j.poetic.2022.101685	I3, I4, I5
124	Gregg, J., & O'Hara, L. (2007). The Red Lotus Health Promotion Model: A new model for holistic, ecological, salutogenic health promotion practice. *Health Promotion Journal of Australia, 18*(1), 12-19.	I3, I4, I5
125	Grodzinsky, E., Walter, S., Viktorsson, L., Carlsson, A.-K., Jones, M. P., & Faresjö, Å. (2015). More negative self-esteem and inferior coping strategies among patients diagnosed with IBS compared with patients without IBS--a case-control study in primary care. *BMC Family Practice, 16*, Article 6. https://doi.org/10.1186/s12875-015-0225-x	I4, I5

Number	Reference	Criteria
126	Gull, M., & Husain, A. (2020). Reliability, validity, and factor structure of religious coping scale. *Iranian Rehabilitation Journal, 18*(3), 301-308. https://doi.org/10.32598/irj.18.3.942.1	I3, I5
127	Gupta, E., Robinson, P. G., Marya, C. M., & Baker, S. R. (2015). Oral health inequalities: Relationships between environmental and Individual factors. *Journal of Dental Research, 94*(10), 1362-1368. https://doi.org/10.1177/0022034515592880	I4, I5
128	Gururatana, O., Baker, S. R., & Robinson, P. G. (2014). Determinants of children's oral-health-related quality of life over time. *Community Dentistry and Oral Epidemiology, 42*(3), 206-215. https://doi.org/10.1111/cdoe.12080	I4, I5
129	Gwiaździński, P., Fedyk, O., Krawczyk, M., & Szymański, M. (2017). Practicing Hatha-Yoga, sense of coherence and sense of agency: Neurophenomenological approach. *Psychiatria Danubina, 29*(Suppl 3), 530-535.	I4, I5
130	Hadid, L. R. E. A., Becker, C., Hamden-Mansour, A., Al-Shuabi, J. T. M., Tharwat, H., & Glascoff, N. (2013). Salutogenic Wellness Promotion Scale: Validation of the Arabic version. *American Journal of Health Education, 44*(4), 229-234. https://doi.org/10.1080/19325037.2013.798210	I3, I4, I5
131	Hag Hamed, D., & Daniel, M. (2019). The influence of fatalistic beliefs on health beliefs among diabetics in Khartoum, Sudan: A comparison between Coptic Christians and Sunni Muslims. *Global Health Promotion, 26*(3), 15-22. https://doi.org/http://dx.doi.org/10.1177/1757975917715884	I3, I4, I5
132	Hagoel, L., Neter, E., Dishon, S., Barnett, O., & Rennert, G. (2003). BRCA1/2 mutation carriers: Living with susceptibility. *Community Genetics, 6*(4), 242-248. https://doi.org/http://dx.doi.org/10.1159/000079386	I4, I5
133	Haraoka, T., Ojima, T., Murata, C., & Hayasaka, S. (2012). Factors influencing collaborative activities between non-professional disaster volunteers and victims of earthquake disasters. *PLoS ONE, 7*(10). https://doi.org/10.1371/journal.pone.0047203	I4, I5
134	Harper, L., Alshammari, D., Ferdynus, C., & Kalfa, N. (2020). Burnout amongst members of the French-speaking Society of Pediatric and Adolescent Urology (SFUPA). Are there specific risk factors? *Journal of Pediatric Urology, 16*(4), 482-486. https://doi.org/https://dx.doi.org/10.1016/j.jpurol.2020.05.014	I3, I5
135	Haugan, G., Rinnan, E., Espnes, G. A., Drageset, J., Rannestad, T., & André, B. (2019). Development and psychometric properties of the Joy-of-Life Scale in cognitively intact nursing home patients. *Scandinavian Journal of Caring Sciences, 33*(4), 801-814. https://doi.org/10.1111/scs.12676	E5
136	Haugan, G. (2021). Nurse-patient interaction: A vital salutogenic resource in nursing home care. In G. Haugan & M. Eriksson (Eds.), *Health promotion in health care – Vital theories and research* (pp. 117-136). Springer https://doi.org/10.1007/978-3-030-63135-2_10	E5
137	Haugan, G., & Dezutter, J. (2021). Meaning-in-life: A vital salutogenic resource for health. In G. Haugan & M. Eriksson (Eds.), *Health promotion in health care – Vital theories and research* (pp. 85-101). Springer. https://doi.org/10.1007/978-3-030-63135-2_8	I3, I4, I5
138	Hazel, K. L., & Mohatt, G. V. (2001). Cultural and spiritual coping in sobriety: Informing substance abuse prevention for Alaska Native communities. *Journal of Community Psychology, 29*(5), 541-562.	I3, I4, I5
139	Heggdal, K., Mendelsohn, J. B., Stepanian, N., Oftedal, B. F., & Larsen, M. H. (2021). Health-care professionals' assessment of a person-centred intervention to empower self-management and health across chronic illness: Qualitative findings from a process	I3, I4, I5

Number	Reference	Criteria
	evaluation study. *Health Expect, 24*(4), 1367-1377. https://doi.org/10.1111/hex.13271	
140	Hemberg, J., Nasman, M., & Nyqvist, F. (2022). Meaningfulness among frail older adults receiving home-based care in Finland. *Health Promotion International, 37*(2), 10. https://doi.org/10.1093/heapro/daab087	I3, I4, I5
141	Henery, N. (2003). Constructions of spirituality in contemporary nursing theory. *Journal of Advanced Nursing, 42*(6), 550-557.	I3, I4, I5
142	Heszen, I., & Mroziak, B. (2006). Human health resources: Contribution of psychology to health sciences. *Polish Psychological Bulletin, 37*(2), 61-64.	I4, I5
143	Hinterberger, T., Auer, J., Schmidt, S., & Loew, T. (2013). Evaluation of a salutogenetic concept for inpatient psychosomatic treatment. *Evidence-Based Complementary and Alternative Medicine, 15*, Article 735731. https://doi.org/10.1155/2013/735731	I3, I5
144	Hjelm, K., & Bard, K. (2013). Beliefs about health and illness in Latin-American migrants with diabetes living in Sweden. *Open Nursing Journal, 7*, 57-65. https://doi.org/10.2174/1874434601307010057	I3, I4, I5
145	Hollis, J. (2019). The psychosocial experience of UK immigration detention. *International Journal of Migration, Health & Social Care, 15*(1), 76-89. https://doi.org/10.1108/IJMHSC-04-2018-0024	I3, I4, I5
146	Hoppe, C., Buntzel, J., von Weikersthal, L. F., Junghans, C., Zomorodbakhsch, B., Stoll, C., Prott, F. J., Fuxius, S., Micke, O., Richter, A., Sallmann, D., & Hubner, J. (2023). Usage of complementary and alternative methods, lifestyle, and psychological variables in cancer care. *In Vivo, 37*(1), 106-114. https://doi.org/https://dx.doi.org/10.21873/invivo.13059	I3, I4, I5
147	Houle, J., Meunier, S., Coulombe, S., Mercerat, C., Gaboury, I., Tremblay, G., . . . Lavoie, B. (2017). Peer positive social control and men's health-promoting behaviors. *American Journal of Men's Health, 11*(5), 1569-1579. https://doi.org/10.1177/1557988317711605	I3, I4, I5
148	Husmann, B. (2009). Besser als nur rumsitzen und nichts tun? Über Achtsamkeitsarbeit, Autogenes Training und Salutogenese [Mindfulness work, autogenic training, and salutogenesis]. *Entspannungsverfahren, 26*, 36-91.	I3, I4, I5
149	Iani, L., Quinto, R. M., Porcelli, P., & Abeni, D. (2019). The effect of sense of coherence and positivity on spiritual well-being and distress in individuals with skin diseases. *Psychotherapy and Psychosomatics, 88*(Supplement 1), 58. https://doi.org/http://dx.doi.org/10.1159/000502467	E5
150	Iden, K. R., Ruths, S., & Hjørleifsson, S. (2015). Residents’ perceptions of their own sadness-a qualitative study in Norwegian nursing homes. *BMC Geriatrics, 15*(1), 1-7.	I3, I4, I5
151	Ikizer, G., & Ozel, E. P. (2021). Examining psychological resilience and posttraumatic growth following terrorist attacks in Turkey. *Traumatology, 27*(2), 236-243. https://doi.org/10.1037/trm0000255	I3, I5
152	Isham, L., Sheng Loe, B., Hicks, A., Wilson, N., Bird, J. C., Bentall, R. P., & Freeman, D. (2022). The meaning in grandiose delusions: measure development and cohort studies in clinical psychosis and non-clinical general population groups in the UK and Ireland. *The Lancet Psychiatry, 9*(10), 792-803. https://dx.doi.org/10.1016/S2215-0366%2822%2900236-X	I3, I4, I5
153	Jähnig, U. (2001). *Zusammenhänge von subjektiven Gottesbildern und Merkmalen psychischer Gesundheit/Krankheit in biographischer Perspektive* [Doctoral dissertation].	E1
154	Jaspal, R., & Cinnirella, M. (2010). Coping with potentially incompatible identities: accounts of religious, ethnic, and sexual identities from	I3, I4, I5

Number	Reference	Criteria
	British Pakistani men who identify as Muslim and gay. *The British Journal of Social Psychology, 49*(Pt 4), 849-870. https://doi.org/10.1348/014466609X485025	
155	Jeserich, F. (2014). The coherence hypothesis: Critical reconsideration, reception history and development of a theoretical model. *Archiv für Religionspsychologie, 36*(1), 1-51. https://doi.org/10.1163/15736121-12341281	I3, I4, I5
156	Jeserich, F., & Stausberg, M. (2015). Making sense of July 22nd: Outcomes of secular and religious/spiritual reappraisal and coping processes from a meaning-making perspective. *German Journal for Young Researchers / Zeitschrift für Nachwuchswissenschaftler, 8*(1), 34-45.	I5
157	Johnsen, G. A. (1992). *Sense of coherence, perceived health, and the performance of health-promoting behaviors*. [Doctoral dissertation, Boston College]. ProQuest Dissertations and Theses Global.	I5
158	Johnson, R. A. (1992). *Account-making and the meaning of translocation for elders*. [Doctoral dissertation, University of Iowa].	I4, I5
159	Jones, K. F., Pryor, J., Care-Unger, C., & Simpson, G. K. (2018). Spirituality and its relationship with positive adjustment following traumatic brain injury: a scoping review. *Brain Injury, 32*(13/14), 1612-1622. https://doi.org/10.1080/02699052.2018.1511066	I3, I4, I5
160	Jorna, T. (2012). The longing for human contact: Why it is crucial that we foster awareness for the inner self in ourselves and in others. *Journal of Spirituality in Mental Health, 14*(1), 23-37. https://doi.org/10.1080/19349637.2012.642665	I3, I4, I5
161	Kalagy, T., Braun-Lewensohn, O., & Abu-Kaf, S. (2017). Youth from fundamentalist societies: What are their attitudes toward war and peace and their relations with anxiety reactions? *Journal of Religion & Health, 56*(3), 1064-1080. https://doi.org/10.1007/s10943-017-0358-4	I4, I5
162	Kalkstein, S. (2007). The Daily Spiritual Experiences Scale and psychological and physical well-being: Demographic comparisons, scale validation, and outcome measures. [Doctoral dissertation, Columbia University]. ProQuest Dissertations and Theses Global.	I3, I5
163	Kállay, É. (2008). Investigation of the relationship between religious growth, positive affect, and meaning in life in a sample of female cancer patients. *Cogniţie Creier Comportament, 12*(2), 161-182.	I3, I5
164	Kark, J. D., Carmel, S., Sinnreich, R., Goldberger, N., & Friedlander, Y. (1996). Psychosocial factors among members of religious and secular kibbutzim. *Israel Journal of Medical Sciences, 32*(3/4), 185-194.	I4, I5
165	Kastbom, Å. A., Sydsjö, G., Bladh, M., Priebe, G., & Svedin, C. G. (2015). Sexual debut before the age of 14 leads to poorer psychosocial health and risky behaviour in later life. *Acta Paediatrica, 104*(1), 91-100.	I4, I5
166	Kelley, M. M., & Chan, K. T. (2012). Assessing the role of attachment to God, meaning, and religious coping as mediators in the grief experience. *Death Studies, 36*(3), 199-227. https://doi.org/10.1080/07481187.2011.553317	I3, I5
167	Khanjari, S., Oskouie, F., & Langius-Eklöf, A. (2012). Psychometric testing of the Caregiver Quality of Life Index-Cancer scale in an Iranian sample of family caregivers to newly diagnosed breast cancer women. *Journal of Clinical Nursing, 21*(3-4), 573-584. https://doi.org/10.1111/j.1365-2702.2011.03850.x	I5
168	Kim, M. J. (2003). Spirituality and meaning in Korean immigrants exposed to cumulative trauma. [Doctoral dissertation, Fuller Theological Seminary]. ProQuest Dissertations and Theses Global.	I5

Number	Reference	Criteria
169	Kimhi, S., Eshel, Y., Lahad, M., & Leykin, D. (2019). National Resilience: A New Self-Report Assessment Scale. *Community Mental Health Journal*, *55*(4), 721-731. https://doi.org/http://dx.doi.org/10.1007/s10597-018-0362-5	E4
170	Kindermann, D., Zeyher, V., Nagy, E., Friederich, H. C., Bozorgmehr, K., & Nikendei, C. (2020). Predictors of asylum seekers' health care utilization in the early phase of resettlement. *Frontiers in Psychiatry, 11*, Article 475. https://doi.org/http://dx.doi.org/10.3389/fpsyt.2020.00475	I3, I4
171	King, G. A., Zwaigenbaum, L., King, S., Baxter, D., Rosenbaum, P., & Bates, A. (2006). A qualitative investigation of changes in the belief systems of families of children with autism or Down syndrome. *Child: Care, Health & Development, 32*(3), 353-369. https://doi.org/10.1111/j.1365-2214.2006.00571.x	I3, I4, I5
172	King, U. (2013). The spiritual potential of childhood: Awakening to the fullness of life. *International Journal of Childrens Spirituality, 18*(1), 4-17. https://doi.org/10.1080/1364436x.2013.776266	I3, I4, I5
173	Kizilhan, J. I., & Klett, C. (2021). *Psychologie für die Arbeit mit Migrant*innen [Psychology for the work with migrants*].Beltz Juventa.	I3, I4, I5
174	Klapheck, K., Nordmeyer, S., Cronjäger, H., Naber, D., & Bock, T. (2012). Subjective experience and meaning of psychoses: the German Subjective Sense in Psychosis Questionnaire (SUSE). *Psychological Medicine, 42*(1), 61-71.	I3, I5
175	Klein, C., & Albani, C. (2011). Die Bedeutung von Religion für die psychische Befindlichkeit: Mögliche Erklärungsansätze und allgemeines Wirkmodell. In C. Klein, H. Berth, & F. Balck (Eds.), *Religiosität und psychische Gesundheit - empirische Befunde und Erklärungsansätze [Religiosity and mental health - Empirical results and explanations*] (pp. 215-245). Juventa.	I5
176	Kloosterhouse, V., & Ames, B. D. (2002). Families' use of religion/spirituality as a psychosocial resource. *Holistic Nursing Practice, 16*(5), 61-76. https://doi.org/10.1097/00004650-200210000-00011	I5, I3
177	Kohls, N. B. (2004). *Außergewöhnliche Erfahrungen - Blinder Fleck der Psychologie?: Eine historische, empirische und methodische Auseinandersetzung mit außergewöhnlichen Erfahrungen und ihrem Zusammenhang mit geistiger Gesundheit*. [Extraordinary experiences - Blind spot of psychology?: A historical, empirical, and methodological examination of extraordinary experiences and their relationship to mental health]. Lit-Verlag.	E2
178	Kohls, N., & Walach, H. (2006). Exceptional experiences and spiritual practice: A new measurement approach. *Spirituality & Health International, 7*(3), 125-150. https://doi.org/10.1002/shi.296	I5, E2
179	Kohls, N., & Walach, H. (2008). Validating four standard scales in spiritually practicing and nonpracticing samples using propensity score matching. *European Journal of Psychological Assessment, 24*(3), 165-173.	I5, I3
180	Kohls, N., Walach, H., & Wirtz, M. (2009). The relationship between spiritual experiences, transpersonal trust, social support, and sense of coherence and mental distress: A comparison of spiritually practising and non-practising samples. *Mental Health, Religion & Culture, 12*(1), 1-23. https://doi.org/10.1080/13674670802087385	I5, E2
181	Kohls, W., & Walach, H. (2007). Psychological distress, experiences of ego loss and spirituality: Exploring the effects of spiritual practice. *Social Behavior and Personality, 35*(10), 1301-1316.	I3, I5
182	Kolstad, A., & Gjesvik, N. (2013). Perceptions of minor mental health problems in China. *Mental Health, Religion & Culture, 16*(4), 335-	I3, I4, I5

Number	Reference	Criteria
	351. https://doi.org/10.1080/13674676.2012.664816	
183	Koons, D. (2013). *The impact of spiritual coping on depression for caregivers of patients diagnosed with Alzheimer's disease.* [Doctoral dissertation, Walden University]. ProQuest Dissertations and Theses Global.	I3, I5
184	Körükcü, Ö., & Kabukcuoğlu, K. (2021). Health promotion among home-dwelling elderly individuals in Turkey. In G. Haugan & M. Eriksson (Eds.), *Health promotion in health care – Vital theories and research* (pp. 313-327). Springer.	I3, I4, I5
185	Koslander, T., Ronning, S., Magnusson, S., & Wiklund Gustin, L. (2021). A 'near-life experience': Lived experiences of spirituality from the perspective of people who have been subject to inpatient psychiatric care. *Scandinavian Journal of Caring Sciences, 35*(2), 512-520. https://doi.org/https://dx.doi.org/10.1111/scs.12863	I3, I4, I5
186	Krägeloh, C. U., Henning, M. A., Billington, R., & Hawken, S. J. (2015). The relationship between quality of life and spirituality, religiousness, and personal beliefs of medical students. *Academic psychiatry: the journal of the American Association of Directors of Psychiatric Residency Training and the Association for Academic Psychiatry, 39*(1), 85-89. https://doi.org/10.1007/s40596-014-0158-z	I3, I5
187	Krampe, H., Goerling, U., Spies, C. D., Gerhards, S. K., Enge, S., Salz, A.-L., Kerper, L. F., & Schnell, T. (2020). Sense of coherence, mental well-being and perceived preoperative hospital and surgery related stress in surgical patients with malignant, benign, and no neoplasms. *BMC Psychiatry, 20*(1), N.PAG-N.PAG. https://doi.org/10.1186/s12888-020-02953-x	I3, I4, I5
188	Krause, N. (2011). Religion and health: Making sense of a disheveled literature. *Journal of Religion and Health, 50*(1), 20-35. https://doi.org/10.1007/s10943-010-9373-4	I3, I4, I5
189	Krause, N., Pargament, K. I., Ironson, G., & Hill, P. (2017). Religious involvement, financial strain, and poly-drug use: Exploring the moderating role of meaning in life. *Substance Use & Misuse, 52*(3), 286-293. https://doi.org/http://dx.doi.org/10.1080/10826084.2016.1225096	I3, I5
190	Kroik, L., Tishelman, C., Stoor, K., & Edin-Liljegren, A. (2021). A salutogenic perspective on end-of-life care among the indigenous Sámi of Northern Fennoscandia. *Healthcare, 9*(6). https://doi.org/10.3390/healthcare9060766	I3, I4, I5
191	Kröz, M., Reif, M., Büssing, A., Zerm, R., Feder, G., Bockelbrink, A., . . . Girke, M. (2011). Does self-regulation and autonomic regulation have an influence on survival in breast and colon carcinoma patients? results of a prospective outcome study. *Health and Quality of Life Outcomes, 9*, Article 85. https://doi.org/10.1186/1477-7525-9-85	I3, I4, I5
192	Kulik, L., & Heine-Cohen, E. (2011). Coping resources, perceived stress and adjustment to divorce among Israeli women: Assessing effects. *Journal of Social Psychology, 151*(1), 5-30. https://doi.org/10.1080/00224540903366453	I4, I5
193	Kumar, A., & Kumar, R. (2022). A study and evaluation of yoga and physical activity. *NeuroQuantology, 20*(5), 3809-3815. https://doi.org/https://dx.doi.org/10.14704/nq.2022.20.5.NQ22675	E1
194	Lake, D. M., & Rhynders, P. (2019). Preliminary evaluation of an adolescent positive health measurement scale: A salutogenic health promotion approach. *Global Health Promotion, 26*(4), 44-51. https://doi.org/10.1177/1757975918757703	I3, I4, I5
195	Langius, A., & Björvell, H. (2001). The applicability of the Antonovsky Sense of Coherence Scale to a group of Pentecostalists. *Scandinavian*	I4, I5

Number	Reference	Criteria
	Journal of Caring Sciences, 15(2), 190-192. https://doi.org/10.1046/j.1471-6712.2001.00025.x	
196	Latham, K., & Clarke, P. J. (2018). Neighborhood disorder, perceived social cohesion, and social participation among older Americans: Findings from the National Health & Aging Trends Study. *Journal of Aging and Health, 30*(1), 3-26. https://doi.org/http://dx.doi.org/10.1177/0898264316665933	I3, I4, I5
197	Laufer, A., & Solomon, Z. (2006a). Posttraumatic symptoms and posttraumatic growth among Israeli youth exposed to terror incidents. *Journal of Social and Clinical Psychology, 25*(4), 429-447. https://doi.org/10.1521/jscp.2006.25.4.429	I3, I5
198	Lee, E., Zahn, A., & Baumann, K. (2011). Religion in psychiatry and psychotherapy? A pilot study: The meaning of religiosity/spirituality from staff's perspective in psychiatry and psychotherapy. *Religions, 2*(4), 525-535. https://doi.org/10.3390/rel2040525	I3, I4, I5
199	Lethborg, C., Aranda, S., Bloch, S., & Kissane, D. (2006). The role of meaning in advanced cancer-integrating the constructs of assumptive world, sense of coherence and meaning-based coping. *Journal of Psychosocial Oncology, 24*(1), 27-42.	I3, I4, I5
200	Levin, J. (2003). Spiritual determinants of health and healing: An epidemiologic perspective on salutogenic mechanisms. *Alternative Therapies in Health and Medicine, 9*(6), 48-57.	I3, I4, I4
201	Levin, J. S. (1996). How religion influences morbidity and health: Reflections on natural history, salutogenesis and host resistance. *Social Science & Medicine, 43*(5), 849-864. https://doi.org/10.1016/0277-9536(96)00150-5	I3, I4, I5
202	Levine, S. Z., Laufer, A., Stein, E., Hamama-Raz, Y., & Solomon, Z. (2009). Examining the relationship between resilience and posttraumatic growth. *Journal of Traumatic Stress, 22*(4), 282-286. https://doi.org/10.1002/jts.20409	I3, I5
203	Ley, C., & Barrio, M. R. (2013). Evaluation of a psychosocial health programme in the context of violence and conflict. *Journal of Health Psychology, 18*(10), 1371-1381. https://doi.org/10.1177/1359105312462435	I4, I5
204	Lindmark, U., & Abrahamsson, K. H. (2015). Oral health-related resources-a salutogenic perspective on Swedish 19-year-olds. *International Journal of Dental Hygiene, 13*(1), 56-64.	I4, I5
205	Lindmark, U., Hakeberg, M., & Hugoson, A. (2011). Sense of coherence and oral health status in an adult Swedish population. *Acta Odontologica Scandinavica, 69*(1), 12-20. https://doi.org/10.3109/00016357.2010.517553	I4, I5
206	Lloyd, C. S., af Klinteberg, B., & DeMarinis, V. (2015). Psychological and existential vulnerability among clinical young women: a quantitative comparison of depression-related subgroups. *Mental Health, Religion & Culture, 18*(4), 259-272. https://doi.org/10.1080/13674676.2015.1021313	I5
207	Löffler, S., Knappe, R., Joraschky, P., & Pöhlmann, K. (2010). Individuelle Sinnkonstruktionen und seelische Gesundheit: Ein Vergleich von Psychotherapeuten und Psychotherapiepatienten [Individual constructions of meaning and mental health: A comparison of psychotherapists and psychotherapy patients]. *Zeitschrift fur Psychosomatische Medizin und Psychotherapie, 56*(4), 358-372.	I4
208	López, J., Camilli, C., & Noriega, C. (2015). Posttraumatic growth in widowed and non-widowed older adults: Religiosity and sense of coherence. *Journal of Religion and Health, 54*(5), 1612-1628. https://doi.org/10.1007/s10943-014-9876-5	I5
209	López-Pérez, B., Hanoch, Y., Holt, K., & Gummerum, M. (2017). Cognitive and affective empathy, Personal belief in a just world, and bullying among offenders. *Journal of Interpersonal Violence, 32*(17), 2591-2604. https://doi.org/10.1177/0886260515593300	I3, I4, I5

Number	Reference	Criteria
210	Ludik, D., & Greeff, A. P. (2020). Exploring factors that helped adolescents adjust and continue with life after the death of a parent. *Omega*. Advance online publication. https://doi.org/http://dx.doi.org/10.1177/0030222820923905	I3, I4, I5
211	Lumby, J., & English, F. (2009). From simplicism to complexity in leadership identity and preparation: exploring the lineage and dark secrets [Article]. International Journal of Leadership in Education, 12(2), 95-114. https://doi.org/10.1080/13603120802449678	I3, I4, I5
212	Lundman, B., Alex, L., Jonsen, E., Norberg, A., Nygren, B., Santamaki Fischer, R., & Strandberg, G. (2010). Inner strength: A theoretical analysis of salutogenic concepts. *International Journal of Nursing Studies, 47*(2), 251-260. https://doi.org/http://dx.doi.org/10.1016/j.ijnurstu.2009.05.020	I3, I4, I5
213	Lundman, B., Arestedt, K., Norberg, Å., Norberg, C., Santamaki Fischer, R., & Lövheim, H. (2015). Psychometric properties of the Swedish version of the Self-Transcendence Scale among very old people. *Journal of Nursing Measurement, 23*(1), 96-111. https://doi.org/10.1891/1061-3749.23.1.96	E5
214	Lustig, D. C. (2005). The adjustment process for individuals with spinal cord injury: The effect of perceived premorbid sense of coherence. *Rehabilitation Counseling Bulletin, 48*(3), 146-156. https://doi.org/10.1177/00343552050480030301	I4, I5
215	Lustig, D. C., & Strauser, D. R. (2002). The relationship between sense of coherence and career thoughts. *Career Development Quarterly, 51*(1), 2-11. https://doi.org/10.1002/j.2161-0045.2002.tb00587.x	I4, I5
216	Mackenzie, E. R., Rajagopal, D. E., Meibohm, M., & Lavizzo-Mourey, R. (2000). Spiritual support and psychological well-being: older adults' perceptions of the religion and health connection. *Alternative therapies in health and medicine, 6*(6), 37-45.	I3, I4, I5
217	Macys, M. J. E. (1996). *Stressful life events and a sense of coherence as predictors of women's spiritual development* [Doctoral dissertation].	E1
218	Maercker, A., & Herrle, J. (2003). Long-term effects of the Dresden bombing: Relationships to control beliefs, religious belief, and personal growth. *Journal of Traumatic Stress, 16*(6), 579-587. https://doi.org/10.1023/B:JOTS.0000004083.41502.2d	I3, I5
219	Maffioletti, F., Vettore, M. V., Rebelo, M., Herkrath, F., Queiroz, A., Herkrath, A. P., . . . Rebelo Vieira, J. (2020). Predisposing, enabling, and need characteristics of dental services utilization among socially deprived schoolchildren. *Journal of Public Health Dentistry, 80*(2), 97-106. https://doi.org/10.1111/jphd.12349	I4, I5
220	Mak, W. W. S., Ng, I. S. W., Wong, C. C. Y., & Law, R. W. (2019). Resilience Style Questionnaire: Development and validation among college students and cardiac patients in Hong Kong. *Assessment, 26*(4), 706-725. https://doi.org/10.1177/1073191116683798	I4, E5
221	Makkar, J. S. (2005). *Positive coping in individuals with prostate cancer: The effects of prostate support groups, social comparison and religious resources.* [Doctoral dissertation, Simon Fraser University]. ProQuest Dissertations and Theses Global.	I5
222	Mana, A., Sagy, S., & Srour, A. (2016). Sense of community coherence and inter-religious relations. *The Journal of Social Psychology, 156*(5), 469-482. https://doi.org/10.1080/00224545.2015.1129302	I4, I5, I3
223	Mana, A., Srour, A., & Sagy, S. (2019). A sense of national coherence and openness to the 'other's' collective narrative: The case of the Israeli–Palestinian conflict. *Peace and Conflict: Journal of Peace Psychology, 25*(3), 226-233. https://doi.org/10.1037/pac0000391	I3
224	Manor-Binyamini, I. (2012). Parental coping with developmental disorders in adolescents within the ultraorthodox Jewish community in	I4, I5

Number	Reference	Criteria
	Israel. *Journal of Autism & Developmental Disorders, 42*(5), 815-826. https://doi.org/10.1007/s10803-011-1313-y	
225	Manor-Binyamini, I., & Nator, M. (2016). Parental coping with adolescent developmental disabilities in terms of stress, sense of coherence and hope within the Druze community of Israel. *Research in Developmental Disabilities, 55*, 358-367. https://doi.org/10.1016/j.ridd.2016.05.003	I4, I5
226	Maoz, B., Hadar, S., & Asher, S. (2011). Culture-sensitive therapy and salutogenesis: Treating Israeli Bedouin of the Negev. *International Review of Psychiatry, 23*(6), 550-554. https://doi.org/http://dx.doi.org/10.3109/09540261.2011.637904	I3, I4, I5
227	Marciano, H., Kimhi, S., & Eshel, Y. (2019). Predictors of individual, community and national resiliencies of Israeli Jews and Arabs. *International Journal of Psychology, 55*(4), 553-561. https://doi.org/http://dx.doi.org/10.1002/ijop.12636	E4
228	Markus, W. (2020). Social dimensions of health: Ritual practice, moral orders, and worlds of meaning in Brazilian Candomblé and Umbanda temples. *Anthropology of Consciousness, 31*(2), 153-173. https://doi.org/10.1111/anoc.12123	I3, I4, I5
229	Marlock, G., & Weiss, H. (2015). *Das Handbuch der Körperpsychotherapie und somatischen Psychologie [The handbook of body psychotherapy and somatic psychology*] North Atlantic Books.	I3, I4, I5
230	Masters, K. S., & Knestel, A. (2011). Religious motivation and cardiovascular reactivity among middle aged adults: Is being pro-religious really that good for you? *Journal of behavioral medicine, 34*(6), 449-461. https://doi.org/10.1007/s10865-011-9352-6	I5
231	Mathias, L. A., Davis, D., & Ferguson, S. (2020). Salutogenic qualities of midwifery care: A best-fit framework synthesis. *Women and Birth.* https://doi.org/http://dx.doi.org/10.1016/j.wombi.2020.03.006	I3, I4, I5
232	Matousek, R. H., & Dobkin, P. L. (2010). Weathering storms: A cohort study of how participation in a mindfulness-based stress reduction program benefits women after breast cancer treatment. *Current Oncology, 17*(4), 62-70.	I3, I4, I5
233	Maus, K., Peusquens, F., Rabe, L. M., & Radbruch, L. (2021). Resilienz, Kohärenz, Lebenssinn sowie andere Konzepte und Begriffe in der Palliativversorgung - Eine Standortbestimmung [Resilience, sense of coherence, meaning in life and other concepts in palliative care - Definitions and perspectives]. *Spiritual Care, 10*(2), 145-155. https://doi.org/10.1515/spircare-2020-0121	I3, I4, I5
234	Mayer, C. H., & Viviers, R. (2014). 'Following the word of God': Empirical insights into managerial perceptions on spirituality, culture and health. *International Review of Psychiatry, 26*(3), 302-314. https://doi.org/http://dx.doi.org/10.3109/09540261.2014.914473	I3, I4, I5
235	Mayer, C.-H. (2011). *The meaning of sense of coherence in transcultural management: A salutogenetic perspective on interactions in a selected South African business organization*. Waxmann.	I4, I5
236	Mayer, C. H., & Boness, C. (2011). Concepts of health and well-being in managers: An organizational study. *International Journal of Qualitative Studies on Health and Well-Being, 6*(4), 12, Article 7143. https://doi.org/10.3402/qhw.v6i4.7143	I3, I4, I5
237	Mayer, C.-H., Surtee, S., & Barnard, A. (2015). Women leaders in higher education: A psycho-spiritual perspective. *South African Journal of Psychology, 45*(1), 102-115. https://doi.org/10.1177/0081246314548869	I3, I4, I5
238	Mayer, C.-H., Viviers, R., Flotman, A.-P., & Schneider-Stengel, D. (2016). Enhancing Sense of Coherence and mindfulness in an ecclesiastical, intercultural group training context. *Journal of Religion & Health, 55*(6), 2023-2038. https://doi.org/10.1007/s10943-016-0301-0	I4, I5

Number	Reference	Criteria
239	Mayer, C.-H., von der Ohe, H., & Viviers, R. (2017). The development of a sense of coherence in family therapy trainees in Germany: A three-year investigation. *Journal of Family Psychotherapy, 28*(3), 237-256. https://doi.org/10.1080/08975353.2017.1294966	I4, I5
240	Mayer, C. H., George, W. M., & Nass, E. (2020). "Care for the Common Home": Responses to Pope Francis's encyclical letter. *Journal of Religion & Health, 59*(1), 416-427. https://doi.org/10.1007/s10943-019-00957-w	I3, I4, I5
241	Mayer, C. H. (2022). Elizabeth Schuyler Hamilton in psychobiography: Sense of coherence and faith across her lifetime. *Frontiers of Psychology, 13*, 948167. https://doi.org/10.3389/fpsyg.2022.948167	I3, I4, I5
242	Mayer, G. S., & Scott, K. J. (1988). An exploration of heterogeneity in an inpatient male alcoholic population. *Journal of Personality Disorders, 2*(3), 243-255. https://doi.org/http://dx.doi.org/10.1521/pedi.1988.2.3.243	I3, I4, I5
243	McEachron, G. (2014). Compassion for the young experiencing the trauma of death. *Journal of Child & Adolescent Trauma, 7*(1), 63-72. https://doi.org/10.1007/s40653-014-0005-0	I3, I4, I5
244	McNeil, D. W. (2023). Behavioural and cognitive-behavioural theories in oral health research: Current state and future directions. *Community Dentistry and Oral Epidemiology, 51*(1), 6-16. https://doi.org/10.1111/cdoe.12840	I3, I4, I5
245	Meddin, J. R. (1998). Dimensions of spiritual meaning and well-being in the lives of ten older Australians. *International Journal of Aging and Human Development, 47*(3), 163-175. https://doi.org/http://dx.doi.org/10.2190/1LXA-K5TN-BGY4-FAXV	I3, I4, I5
246	Meghani, S. H., Peterson, C., Kaiser, D. H., Rhodes, J., Rao, H. Y., Chittams, J., & Chatterjee, A. (2018). A pilot study of a mindfulness-based art therapy intervention in outpatients with cancer. *American Journal of Hospice & Palliative Medicine, 35*(9), 1195-1200. https://doi.org/10.1177/1049909118760304	E5
247	Meleth, S., Allen, L., Kvale, E., Meredith, R., Spencer, S., Heslin, M., . . . Partridge, E. (2009). A qualitative study of exceptional survivors of cancer. *Journal of Clinical Oncology, 27*, e17522-e17522.	I3, I5
248	Melin, R., & Fugl-Meyer, A. R. (2003). On prediction of vocational rehabilitation outcome at a Swedish employability institute. *Journal of Rehabilitation Medicine, 35*(6), 284-289.	I4, I5
249	Merakou, K., Kyklou, E., Antoniadou, E., Karageorgos, G., Doufexis, E., & Barbouni, A. (2016). Salutogenesis and the monks of Athos, Greece: A spiritual health promotion? *Advances in Mind-Body Medicine, 30*(2), 11-18.	I4, I5
250	Merakou, K., Kyklou, E., Antoniadou, E., Theodoridis, D., Doufexis, E., & Barbouni, A. (2017). Health-related quality of life of a very special population: monks of Holy Mountain Athos, Greece. *Quality of Life Research, 26*(11), 3169-3175. https://doi.org/http://dx.doi.org/10.1007/s11136-017-1622-5	I4, I5
251	Merakou, K., Taki, S., Barbouni, A., Antoniadou, E., Theodoridis, D., Karageorgos, G., & Kourea-Kremastinou, J. (2017). Sense of coherence (SOC) in Christian Orthodox monks and nuns in Greece. *Journal of Religion and Health, 56*(2), 521-529. https://doi.org/10.1007/s10943-016-0244-5	I4, I5
252	Merakou, K., Xefteri, E., & Barbouni, A. (2017c). Sense of coherence in religious Christian Orthodox women in Greece. *Community Mental Health Journal, 53*(3), 353-357. https://doi.org/10.1007/s10597-016-0051-1	I4, I5
253	Merluzzi, T. V., Salamanca-Balen, N., Philip, E. J., & Salsman, J. M. (2023). 'Letting go'—Relinquishing control of illness outcomes to God	I3, I5

Number	Reference	Criteria
	and quality of life: Meaning/peace as a mediating mechanism in religious coping with cancer. *Social Science & Medicine, 317*, 1-9. https://doi.org/10.1016/j.socscimed.2022.115597	
254	Messecar, D. C. (2002). Older people perceived health as going and doing something meaningful. *Evidence Based Nursing, 5*(3), 96-96.	I3, I4, I5
255	Meyer-Parsons, B., Van Etten, S., & Shaw, J. R. (2017). The healer's art (HART): Veterinary students connecting with self, peers, and the profession. *Journal of Veterinary Medical Education, 44*(1), 187-197. https://doi.org/http://dx.doi.org/10.3138/jvme.0116-022R	I3, I4, I5
256	Midling, A., & Czigany, L. (2008). "Rede-spuren": Untersuchung der Bindung von spirituellen Leitern in verschiedenen religiösen Gemeinschaften ["Linguistic traces": Studying the attachment of spiritual leaders in different religious communities]. *European Journal of Mental Health, 3*(2), 171-199. https://doi.org/https://dx.doi.org/10.1556/EJMH.3.2008.2.1	I3, I4, I5
257	Mihandoust, S., Nematollahzadeh, Z., Shirvani, M., Al-Karboolee, N. J. N., Joudi, M., Fouroozandeh, M., & Moghadam, F. A. (2022). The relationship between personality dimensions with resiliency and sense of coherence with respect to the role of spiritual health in the patients' candidate for eye surgery. *Journal of Family Medicine and Primary Care, 11*(10), 6350-6355. https://doi.org/10.4103/jfmpc.jfmpc_198_21	I3
258	Minnie, C. S., & Minnie, F. G. (2017). Perspectives on salutogenesis of scholars writing in Afrikaans. In M. B. Mittelmark, S. Sagy, M. Eriksson, G. F. Bauer, J. M. Pelikan, B. Lindström, & G. A. Espnes (Eds.), *The handbook of salutogenesis* (pp. 351-355). Springer. https://doi.org/10.1007/978-3-319-04600-6_34	I3, I4, I5
259	Milberg, A., & Strang, P. (2007). What to do when 'there is nothing more to do'? A study within a salutogenic framework of family members' experience of palliative home care staff. *Psycho-Oncology, 16*(8), 741-751.	I3, I4, I5
260	Mjosund, N. H., Eriksson, M., Norheim, I., Keyes, C. L. M., Espnes, G. A., & Vinje, H. F. (2015). Mental health as perceived by persons with mental disorders - an interpretative phenomenological analysis study. *International Journal of Mental Health Promotion, 17*(4), 215-233. https://doi.org/10.1080/14623730.2015.1039329	I3, I4, I5
261	Mlonzi, E. N., & Strümpfer, D. J. W. (1998). Antonovsky's sense of coherence scale and 16PF second-order factors. *Social Behavior & Personality, 26*(1), 39-50. https://doi.org/10.2224/sbp.1998.26.1.39	I4, I5
262	Mohangi, K., Ebersohn, L., & Eloff, I. (2011). "I am doing okay": Intrapersonal coping strategies of children living in an institution. *Journal of Psychology in Africa, 21*(3), 397-404. https://doi.org/10.1080/14330237.2011.10820473	I3, I4, I5
263	Moe, A., Hellzen, O., Ekker, K., & Enmarker, I. (2013). Inner strength in relation to perceived physical and mental health among the oldest old people with chronic illness. *Aging & Mental Health, 17*(2), 189-196. https://doi.org/10.1080/13607863.2012.717257	E5
264	Möller, A. (2007). Spiritualität und Religiosität - Sinnfragen als Thema der Medizinpsychologie [Spirituality and religiosity - Fundamental philosophical questions as a topic of medical psychology]. In H. Förstl (Ed.), *Theory of mind* (pp. 163-169). Springer.	I3, I4, I5
265	Möller, A., & Reimann, S. (2004). Religiöse Einstellung und Zukunftssicht in einer studentischen Untersuchungsgruppe [Religious dimensions and optimistic perception of the future in a sample of students]. *PPmP: Psychotherapie Psychosomatik Medizinische Psychologie, 54*(9-10), 383-386. https://doi.org/10.1055/s-2004-828290	I3, I5
266	Moody, B. (2019). *An examination of the relationship between perceived wellness and compliance with the go365 health rewards program*	I3, I5

Number	Reference	Criteria
	for employees of the archdiocese of Louisville. [Doctoral dissertation, Spalding University]. ProQuest Dissertations and Theses Global.	
267	Moon, P. K. (2014). *Resilience and wellbeing in a sample of Singaporean clergy.* [Doctoral dissertation, Fuller Theological Seminary]. ProQuest Dissertations and Theses Global.	I4, I5
268	Moore, J. (2007). R. A. Fisher: A faith fit for eugenics. *Studies in History and Philosophy of Science Part C :Studies in History and Philosophy of Biological and Biomedical Sciences, 38*(1), 110-135. https://doi.org/https://dx.doi.org/10.1016/j.shpsc.2006.12.007	I3, I4, I5
269	Morandi, A., Tosto, C., Roberti di Sarsina, P., & Dalla Libera, D. (2011). Salutogenesis and Ayurveda: Indications for public health management. *The EPMA Journal, 2*(4), 459-465. https://doi.org/10.1007/s13167-011-0132-8	I3, I4, I5
270	Morgenthaler, C. (2002). Von der Pastoralpsychologie zur empirischen Religionspsychologie? Das Beispiel 'religiöses Coping' [From pastoral psychology to empirical psychology of religion? The example of religious coping]. *Wege zum Menschen, 54*(5), 287-300.	I3, I4, I5
271	Mowla, F., Khanjari, S., & Haghani, S. (2020). Effect of the combination of Benson's relaxation technique and brief psychoeducational intervention on religious coping, sense of coherence, and quality of life of family caregivers. *Journal of Education and Health Promotion, 9*(1), 7, Article 117. https://doi.org/10.4103/jehp.jehp_653_19	I5
272	Mueller, S. (2020). Are paradoxes of amoeboid cognition, memristors, and memory mandating a re-conceptualization of actions and behaviors? *Explore, 16*(4), 250-256. https://doi.org/https://dx.doi.org/10.1016/j.explore.2020.04.001	I3, I4, I5
273	Mullen, P. M., Smith, R. M., & Hill, E. W. (1994). Sense of coherence as a mediator of stress for cancer patients and spouses. *Journal of Psychosocial Oncology, 11*(3), 23-46.	I5
274	Murken, S. (1998). *Gottesbeziehung und psychische Gesundheit: Die Entwicklung eines Modells und seine empirische Überprüfung*. Waxmann..	I3, I5
275	Murken, S., & Namini, S. (2004). Selbst gewählte Mitgliedschaft in religiösen Gemeinschaften: Ein Versuch der Lebensbewältigung? In Zwingmann, C., & Moosbrugger, H. (Eds.), *Religiosität: Messverfahren und Studien zur Gesundheit und Lebensbewältigung. Neue Beiträge zur Religionspsychologie* (pp. 299-316). Waxmann.	I3
276	Murray, T. M. (2014). *Faith-based organizations, sense of coherence & stress management among African-American adults*. [Doctoral dissertation, University of Texas at Tyler]. Nursing Theses and Dissertations.	E1
277	Namini, S., & Murken, S. (2009). Self-chosen involvement in new religious movements (NRMs): Well-being and mental health from a longitudinal perspective. *Mental Health, Religion & Culture, 12*(6), 561-585. https://doi.org/10.1080/13674670902897618	I3
278	Nanín, J. E. (2001). *Burnout, sense of coherence, and health status in New York City HIV service providers*. [Doctoral dissertation, Columbia University]. ProQuest Dissertations and Theses Global.	E4
279	Neerland, C. E., Avery, M. D., Looman, W. S., Saftner, M. A., Rockwood, T. H., & Gurvich, O. V. (2020). Development and testing of the preparation for labor and birth instrument. *Journal of Obstetric, Gynecologic, and Neonatal Nursing, 49*(2), 200-211. https://doi.org/10.1016/j.jogn.2019.12.006	I3, I4, I5
280	Neikrug, S. M. (2003). Worrying about a frightening old age. *Aging & Mental Health, 7*(5), 326.	I4, I5

Number	Reference	Criteria
	https://doi.org/10.1080/1360786031000150702	
281	Nissley, G. E., Jr. (2009). *Rural resilience: Developing a model for the role of spirituality and religiosity in the resilience and well-being of rural adults*. [Doctoral dissertation, Capella University]. ProQuest Dissertations and Theses Global.	E1
282	Nothdurft, I. (2008). Gesundheitsförderung und Salutogenese: Patientenzentrierte Beratung in der Pflege [Health promotion and salutary nursing: patient centered counseling in nursing]. *Pflege Zeitschrift, 61*(7), 388-391.	I3, I4, I5
283	Nuccitelli, C., Valentini, A., Caletti, M. T., Caselli, C., Mazzella, N., Forlani, G., & Marchesini, G. (2018). Sense of coherence, self-esteem, and health locus of control in subjects with type 1 diabetes mellitus with/without satisfactory metabolic control. *Journal of Endocrinological Investigation, 41*(3), 307-314. https://doi.org/10.1007/s40618-017-0741-8	I4, I5
284	Nudelman, G., & Shiloh, S. (2011). Who deserves to be sick? An exploration of the relationships between belief in a just world, illness causal attributions and their fairness judgements. *Psychology, Health & Medicine, 16*(6), 675-685. https://doi.org/10.1080/13548506.2011.569730	I4, I5, I3
285	Nygren, B., Aléx, L., Jonsén, E., Gustafson, Y., Norberg, A., & Lundman, B. (2005). Resilience, sense of coherence, purpose in life and self-transcendence in relation to perceived physical and mental health among the oldest old. *Aging & Mental Health, 9*(4), 354-362. https://doi.org/10.1080/1360500114415	E5
286	O'Brien, R., Hunt, K., & Hart, G. (2009). 'The average Scottish man has a cigarette hanging out of his mouth, lying there with a portion of chips': prospects for change in Scottish men's constructions of masculinity and their health-related beliefs and behaviours. *Critical Public Health, 19*(3-4), 363-381. https://doi.org/10.1080/09581590902939774	I3, I4, I5
287	Oliveira, D. V. B., da Silva, J. F., Araujo, T. A. D., & Albuquerque, U. P. (2022). Influence of religiosity and spirituality on the adoption of behaviors of epidemiological relevance in emerging and re-emerging diseases: The case of Dengue fever. *Journal of Religion & Health, 61*(1), 564-585. https://doi.org/10.1007/s10943-021-01436-x	I3, I5
288	Oluyinka, O. (2011). Psychological predictors of attitude towards seeking professional psychological help in a Nigerian university student population. *South African Journal of Psychology, 41*(3), 310-327.	E5
289	Oman, D., & Nuru-Jeter, A. M. (2018). Social identity and discrimination in religious/spiritual influences on health. In D. Oman (Ed.), *Why religion and spirituality matter for public health: Evidence, implications, and resources* (Vol. 2, pp. 111-137). Springer International Publishing. https://doi.org/10.1007/978-3-319-73966-3_6	I3, I4, I5
290	Oman, D., & Syme, S. L. (2018). Social and community-level factors in health effects from religion/spirituality. In D. Oman (Ed.), *Why religion and spirituality matter for public health: Evidence, implications, and resources* (Vol. 2, pp. 81-110). Springer International Publishing. https://doi.org/10.1007/978-3-319-73966-3_5	I3, I4, I5
291	Orth, Z., Moosajee, F., & Van Wyk, B. (2022). Measuring mental wellness of adolescents: A systematic review of instruments. *Frontiers in Psychology, 13*, 835601. https://doi.org/10.3389/fpsyg.2022.835601	I4, I5
292	Orth, Z., & Van Wyk, B. (2022). Discourses of mental wellness among adolescents living with HIV in Cape Town, South Africa. *Psychology Research and Behavior Management, 15*, 1435-1450. https://doi.org/10.2147/prbm.S360145	I3, I4, I5

Number	Reference	Criteria
293	Orth, Z., & Van Wyk, B. (2022). Rethinking mental health wellness among adolescents living with HIV in the African context: An integrative review of mental wellness components. *Frontiers in Psychology, 13*, 955869. https://doi.org/10.3389/fpsyg.2022.955869	I3, I4, I5
294	Oshita, D., Hattori, K., & Iwakuma, M. (2013). A Buddhist-based meditation practice for care and healing: An introduction and its application. *International Journal of Nursing Practice, 19*(Suppl 2), 15-23. https://doi.org/10.1111/ijn.12040	I4, I5
295	Ośmiałowska, E., Staś, J., Chabowski, M., & Jankowska-Polańska, B. (2022). Illness perception and quality of life in patients with breast cancer. *Cancers, 14*(5). https://doi.org/10.3390/cancers14051214	I3, I4, I5
296	Otey-Scott, S. (2008). *A lesson in gratitude: Exploring the salutogenic relationship between gratitude and health.* [Doctoral dissertation, Regent University]. ProQuest Dissertations and Theses Global.	I3, I4, I5
297	Owens, J., & Sami, W. (2016). The Role of the Qur'an and Sunnah in Oral Health. *Journal of Religion & Health, 55*(6), 1954-1967. https://doi.org/10.1007/s10943-015-0095-5	I3, I4, I5
298	Ozaki, M., Kobayashi, K., & Oku, T. (2006). Healthy Spirituality and Genuineness -- From Research on Spirituality with Authenticity and Flow. *Journal of International Society of Life Information Science, 24*(1), 165-169.	I3, I5
299	Pandya, S. P. (2016). Spiritual programmes? For prisoners in India: Insights for criminological social work practice. *Journal of Social Work Practice, 30*(4), 417-430. https://doi.org/10.1080/02650533.2015.1132688	I4, I5
300	Pandya, S. P. (2018). Spirituality to build resilience in primary caregiver parents of children with autism spectrum disorders: A cross-country experiment. *International Journal of Developmental Disabilities, 64*(1), 53-64. https://doi.org/10.1080/20473869.2016.1222722	I4, I5
301	Pandya, S. P. (2020). Meditation to improve the quality of life of community-dwelling ever-single older adults: A multi-city five-year follow-up experiment. *Journal of Religion, Spirituality & Aging, 32*(1), 45-69. https://doi.org/10.1080/15528030.2019.1600631	I4, I5
302	Pandya, S. (2021). Social Work with Environmental Migrants: Exploring the Scope for Spiritually Sensitive Practice. *Social Work, 66*(2), 148-156. https://doi.org/10.1093/sw/swab001	I3, I5
303	Pandya, S. P. (2021). Older women and wellbeing through the pandemic: Examining the effect of daily online yoga lessons. *Health Care for Women International, 42*(11), 1255-1278. https://doi.org/10.1080/07399332.2021.1932897	I3, I4, I5
304	Patel, K., Wall, K., Bott, N. T., Katonah, D. G., & Koopman, C. (2015). A qualitative investigation of the effects of psycho-spiritual integrative therapy on breast cancer survivors' experience of paradox. *Journal of Religion and Health, 54*(1), 253-263. https://doi.org/10.1007/s10943-014-9827-1	I3, I4, I5
305	Paula, J. S. d., Rodrigues, P. A., Mattos, F. F., Abreu, M. H. N. G. d., Chalub, L. L. F. H., & Zina, L. G. (2022). Mother's education and family relations protect children from dental caries experience: a salutogenic approach. *Brazilian Oral Research, 36*, e111. https://doi.org/10.1590/1807-3107bor-2022.vol36.0111	I3, I4, I5
306	Pelters, B., & Roxberg, A. (2018). "Don't stop believing!" From health religiosity to an equality-enhancing hermeneutic of health promotion. *International Journal of Qualitative Studies on Health and Well-Being, 13*, 13, Article 1555420. https://doi.org/10.1080/17482631.2018.1555420	I3, I4, I5

Number	Reference	Criteria
307	Peter, C., Müller, R., Cieza, A., & Geyh, S. (2012). Psychological resources in spinal cord injury: A systematic literature review. *Spinal Cord, 50*(3), 188-201. https://doi.org/10.1038/sc.2011.125	I3, I4, I5
308	Peterman, A. H., Reeve, C. L., Winford, E. C., Cotton, S., Salsman, J. M., McQuellon, R., . . . Campbell, C. (2014). Measuring meaning and peace with the FACIT–Spiritual Well-Being Scale: Distinction without a difference? *Psychological Assessment, 26*(1), 127-137. https://doi.org/10.1037/a0034805	I3, I5
309	Pham, P. N., Vinck, P., Kinkodi, D. K., & Weinstein, H. M. (2010). Sense of coherence and its association with exposure to traumatic events, posttraumatic stress disorder, and depression in eastern Democratic Republic of Congo. *Journal of Traumatic Stress, 23*(3), 313-321. https://doi.org/10.1002/jts.20527	I4, I5
310	Pienaar, J. M., Beukes, R. B. I., & Esterhuyse, K. G. F. (2006). The relationship between conservatism and psychological well-being in adolescents. *South African Journal of Psychology, 36*(2), 391-406. https://doi.org/10.1177/008124630603600211	I5
311	Pienaar, J. M., & De Witte, H. (2016). Work locus of control and sense of coherence as antecedents of job insecurity. *South African Journal of Business Management, 47*(3), 35-43. https://doi.org/10.4102/sajbm.v47i3.66	I4, I5
312	Pierce, L. L. (1997). The framework of systemic organization applied to older adults as family caregivers of persons with chronic illness and disability. *Gastroenterology Nursing, 20*(5), 168-175. /http://dx.doi.org/10.1097/00001610-199709000-00004	I3, I4, I5
313	Pietkiewicz, I. J. (2014). Salutary, pathogenic, and pathoplastic aspects of the Jehovah's Witness culture. *Journal of Family Studies, 20*(2), 148-165. https://doi.org/10.1080/13229400.2014.11082003	I3, I4, I5
314	Podolin-Danner, N., Wenzl, M., Knorr, A., Fuchshuber, J., Silani, G., & Unterrainer, H. F. (2022). The Swedish version of the multidimensional inventory for religious/spiritual well-being–Part II: Development of a four-field typology. *Frontiers in Psychology, 13*, 1029101-1029101.	I5, E2
315	Popp-Baier, U. (2008). Erfahrung, Identität, Religion. Zur psychologischen Analyse individueller Religiosität [Life experience, identity, Religion. A psychological analysis of individual religiosity]. *Journal für Psychologie, 16*(3). https://doi.org/https://www.journal-fuer-psychologie.de/index.php/jfp/article/view/203/145	I3, I4, I5
316	Porritt, J. M., Sufi, F., Barlow, A., & Baker, S. R. (2014). The role of illness beliefs and coping in the adjustment to dentine hypersensitivity. *Journal of Clinical Periodontology, 41*(1), 60-69. https://doi.org/10.1111/jcpe.12177	I3, I4, I5
317	Posadzki, P., Stockl, A., Musonda, P., & Tsouroufli, M. (2010). A mixed-method approach to sense of coherence, health behaviors, self-efficacy and optimism: Towards the operationalization of positive health attitudes. *Scandinavian Journal of Psychology, 51*(3), 246-252.	I4, I5
318	Postolică, R., Enea, V., Dafinoiu, I., Petrov, I., & Azoicăi, D. (2019). Association of sense of coherence and supernatural beliefs with death anxiety and death depression among Romanian cancer patients. *Death Studies, 43*(1), 9-19. https://doi.org/10.1080/07481187.2018.1430083	I5
319	Pretter, S. (2002). *Religiosity, social support, and sense of coherence as psychosocial resources for caregiving spouses of terminally ill cancer patients*. [Doctoral dissertation, City University of New York]. ProQuest Dissertations and Theses Global.	E1

Number	Reference	Criteria
320	Punyoo, J., Pothiban, L., Jintrawet, U., Mesukko, J., & Reungrongrat, S. (2020). Factors Associated with Psychological Well-being among Parents of a Critically Ill Child in Pediatric Intensive Care Unit. *Walailak Journal of Science & Technology, 17*(5), 437-449.	I5
321	Qiu, X., Zhang, N., Pan, S.-J., Zhao, P., & Wu, B.-W. (2020). Sense of coherence and health-related quality of life in patients with brain metastases. *Frontiers in Psychology, 11*. https://doi.org/10.3389/fpsyg.2020.01516	I4, I5
322	Ragger, K., Hiebler-Ragger, M., Herzog, G., Kapfhammer, H.-P., & Unterrainer, H. F. (2019). Sense of coherence is linked to post-traumatic growth after critical incidents in Austrian ambulance personnel. *BMC Psychiatry, 19*(1). https://doi.org/10.1186/s12888-019-2065-z	I5
323	Rashid, M., Kristofferzon, M.-L., Heiden, M., & Nilsson, A. (2018). Factors related to work ability and well-being among women on sick leave due to long-term pain in the neck/shoulders and/or back: A cross-sectional study. *BMC Public Health, 18*(1), 672-672. https://doi.org/10.1186/s12889-018-5580-9	I4, I5
324	Reddemann, L. (2016). *Mitgefühl, Trauma und Achtsamkeit in psychodynamischen Therapien* [Sympathy, trauma, and mindfulness in psychodynamic therapies]. Vandenhoeck & Ruprecht.	I3, I4, I5
325	Richardson Gibson, L. M., & Parker, V. (2003). Inner resources as predictors of psychological well-being in middle-income African American breast cancer survivors. *Cancer Control, 10*(5 Suppl), 52-59. https://doi.org/https://doi.org/10.1177/107327480301005s08	E2
326	Rieforth, J. (2006). *Triadisches Verstehen in sozialen Systemen: Gestaltung komplexer Wirklichkeiten. Ausgewählte Beiträge zur Jahrestagung der Deutschen Gesellschaft für Systemische Therapie und Familientherapie (DGSF) an der Carl von Ossietzky Universität Oldenburg 2005* [Triadic understanding in social systems]. Carl-Auer Verlag.	I3, I4, I5
327	Rigo, D. C. A., Ferreira, J. B. d. S., Costa, L. R., & Freire, M. d. C. M. (2022). Religiosity is associated with caregivers' perception of preschool children's dental health. *Brazilian Oral Research, 36*, e0121. https://doi.org/10.1590/1807-3107bor-2022.vol36.0121	I5
328	Ripsch, J. P. (2002). Development and maintenance of positive sense of self in successful obese women [Doctoral dissertation, Loyola University Chicago]. ProQuest Dissertations and Theses Global.	E1
329	Ritunnano, R., & Bortolotti, L. (2022). Do delusions have and give meaning? *Phenomenology and the Cognitive Sciences, 21*(4), 949-968. https://doi.org/10.1007/s11097-021-09764-9	I3, I4, I5
330	Rohani, C., Abedi, H.-A., Omranipour, R., & Langius-Eklöf, A. (2015). Health-related quality of life and the predictive role of sense of coherence, spirituality and religious coping in a sample of Iranian women with breast cancer: a prospective study with comparative design. *Health and Quality of Life Outcomes, 13*, Article 40. https://doi.org/10.1186/s12955-015-0229-1	I4, I5
331	Rokach, A., Findler, L., Chin, J., Lev, S., & Kollender, Y. (2013). Cancer patients, their caregivers and coping with loneliness. *Psychology, Health & Medicine, 18*(2), 135-144. https://doi.org/10.1080/13548506.2012.689839	I5
332	Ross, L. T., Heming, B., & Lane, A. (2022). Family Unpredictability and Sense of Coherence: Relationships With Anxiety and Depression in Two Samples. *Psychological Reports*, 332941221080409. https://doi.org/10.1177/00332941221080409	I4, I5
333	Rosselli, M., Salimbeni, M. V., Bessi, C., Nesi, E., Caruso, S., Arboretti, D., ... & Stasi, C. (2015). Screening of distress among hospitalized	I4, I5, I3

Number	Reference	Criteria
	patients in a department of internal medicine. *Asian Journal of Psychiatry, 18*, 91-96.	
334	Rumbold, B. D. (2003). Caring for the spirit: Lessons from working with the dying. *Medical Journal of Australia, 179*(6 Suppl.), S11-S13. https://doi.org/https://dx.doi.org/10.5694/j.1326-5377.2003.tb05568.x	I3, I4, I5
335	Rumbold, B. D. (2007). A review of spiritual assessment in health care practice. *The Medical Journal of Australia, 186*(10 Suppl.), S60-S62.	I3, I4, I5
336	Saba, G. W. (1999). What do family physicians believe and value in their work? *The Journal of the American Board of Family Practice / American Board of Family Practice, 12*(3), 206-213. https://doi.org/https://dx.doi.org/10.3122/jabfm.12.3.206	I3, I4, I5
337	Sadati, A. K., Salehzade, H., Hemmati, S., Darvish, M., Heydari, S. T., & Tabrizi, R. (2015). The causal factors associated with the loving care of the mothers of children with multiple disabilities. *International Journal of Community-Based Nursing and Midwifery, 3*(4), 309.	I5
338	Sadati, A. K., Lankarani, K. B., Gharibi, V., Fard, M. E., Ebrahimzadeh, N., & Tahmasebi, S. (2015). Religion as an empowerment context in the narrative of women with breast cancer. *Journal of Religion and Health, 54*(3), 1068-1079. https://doi.org/https://dx.doi.org/10.1007/s10943-014-9907-2	I3, I4, I5
339	Sagberg, S., & Roen, I. (2011). Social practices of encountering death: a discussion of spiritual health in grief and the significance of worldview. *International Journal of Childrens Spirituality, 16*(4), 347-360. https://doi.org/10.1080/1364436x.2011.642854	I3, I4, I5
340	Saint Arnault, D., & Zonp, Z. (2022). Understanding help-seeking barriers after Gender-Based Violence: Validation of the Barriers to Help Seeking-Trauma version (BHS-TR). *Archives of Psychiatric Nursing, 37*, 1-9. https://doi.org/10.1016/j.apnu.2021.12.004	I3, I4, I5
341	Salvatore, A., & Amir-Moazami, S. (2002). Religiöse Diskurstraditionen. Zur Transformation des Islam in kolonialen, postkolonialen und europäischen Öffentlichkeiten [*The transformation of Islam as a discursive tradition in colonial, post-colonial and European public spheres. Berliner Journal für Soziologie, 12*(3), 309-330. https://doi.org/10.1007/BF03204060	I3, I4, I5
342	Sarenmalm, E. K., Browall, M., Persson, L. O., Fall-Dickson, J., & Gaston-Johansson, F. (2013). Relationship of sense of coherence to stressful events, coping strategies, health status, and quality of life in women with breast cancer. *Psycho-Oncology, 22*(1), 20-27. https://doi.org/10.1002/pon.2053	I5
343	Schäfer, S. K., Lass-Hennemann, J., Groesdonk, H., Volk, T., Bomberg, H., Staginnus, M., . . . Michael, T. (2018). Mental mealth in anesthesiology and ICU staff: Sense of coherence matters. *Frontiers in Psychiatry, 9*, Article 440. https://doi.org/10.3389/fpsyt.2018.00440	I4, I5
344	Schmees, P., Braig, J., Nilles, H., Kerkhoff, D., Demir, Z., Rueth, J.-E., Lohaus, A., & Eschenbeck, H. (2022). Wohlbefinden und Ressourcen von Minderjährigen mit Flüchtlingshintergrund im Vergleich zu Minderjährigen mit Migrations- oder einheimischem Hintergrund [Well-being and resources of minors with refugee background in comparison to minors with migration or native background]. *European Journal of Health Psychology, 29*(1), 3-14. https://doi.org/10.1027/2512-8442/a000099	I3, I5
345	Schmuck, J., Hiebel, N., Rabe, M., Schneider, J., Erim, Y., Morawa, E., Jerg-Bretzke, L., Beschoner, P., Albus, C., Hannemann, J., Weidner, K., Steudte-Schmiedgen, S., Radbruch, L., Brunsch, H., & Geiser, F. (2021). Sense of coherence, social support and religiosity as resources for medical personnel during the COVID-19 pandemic: A web-based survey among 4324 health care workers within the German Network University Medicine. *Plos One, 16*(7), 1-18. https://doi.org/10.1371/journal.pone.0255211	I3

Number	Reference	Criteria
346	Schnell, T., Höge, T., & Pollet, E. (2013). Predicting meaning in work: Theory, data, implications. *The Journal of Positive Psychology, 8*(6), 543-554. https://doi.org/10.1080/17439760.2013.830763	I3, I5
347	Schlieter, J. (2017). Buddhist insight meditation (Vipassan) and Jon Kabat-Zinn's "Mindfulness-based Stress Reduction": An example of dedifferentiation of religion and medicine?. *Journal of Contemporary Religion, 32*(3), 447-463. https://doi.org/10.1080/13537903.2017.1362884	I3, I4, I5
348	Schrank, B., Brownell, T., Jakaite, Z., Larkin, C., Pesola, F., Riches, S., . . . Slade, M. (2016). Evaluation of a positive psychotherapy group intervention for people with psychosis: Pilot randomised controlled trial. *Epidemiology and Psychiatric Sciences, 25*(3), 235-246. https://doi.org/10.1017/S2045796015000141	I3, I4, I5
349	Schwarzer, R., Jerusalem, M., & Weber, H. (2002). *Gesundheitspsychologie von A bis Z: Ein Handwörterbuch* [Health psychology from A to Z: A dictionary]. Hogrefe.	I3, I4, I5
350	Seah, B., Espnes, G. A., Hong, W. T., & Wang, W. R. (2022). Salutogenic Healthy Ageing Programme Embracement (SHAPE)- an upstream health resource intervention for older adults living alone and with their spouses only: complex intervention development and pilot randomized controlled trial. *BMC Geriatrics, 22*(1), 18, Article 932. https://doi.org/10.1186/s12877-022-03605-3	I3, I4, I5
351	Seaward, B. L. (2000). Stress and human spirituality 2000: At the cross roads of physics and metaphysics. *Applied Psychophysiology Biofeedback, 25*(4), 241-246. https://doi.org/https://dx.doi.org/10.1023/A:1026458905835	I3, I4, I5
352	Senka, J. (1995). Coping processes in groups at risk in the context of psychological health aspects. *Studia Psychologica, 37*(3), 154-156.	E4
353	Sergooris, A., Verbrugghe, J., De Baets, L., Meeus, M., Roussel, N., Smeets, R., Bogaerts, K., & Timmermans, A. (2023). Are contextual factors associated with activities and participation after total hip arthroplasty? A systematic review. *Annals of Physical and Rehabilitation Medicine, 66*(5), 101712. https://doi.org/10.1016/j.rehab.2022.101712	I3, I4, I5
354	Serrano-Gómez, D., Velasco-González, V., Alconero-Camarero, A. R.,..., & Sarabia-Cobo, C. (2022). COVID-19 Infection among Nursing Students in Spain: The Risk Perception, Perceived Risk Factors, Coping Style, Preventive Knowledge of the Disease and Sense of Coherence as Psychological Predictor Variables: A Cross Sectional Survey. *Nursing Reports, 12*(3), 661-673. https://doi.org/10.3390/nursrep12030066	I4, I5
355	Shakespeare-Finch, J., Schweitzer, R. D., King, J., & Brough, M. (2014). Distress, coping, and posttraumatic growth in refugees from Burma. *Journal of Immigrant & Refugee Studies, 12*(3), 311-330. https://doi.org/10.1080/15562948.2013.844876	I3, I4, I5
356	Shani, E., Ayalon, A., Hammad, I. A., & Sikron, F. (2003). What picture is worth a thousand words? A comparative evaluation of a burn prevention programme by type of medium in Israel. *Health Promotion International, 18*(4), 361-371. https://doi.org/10.1093/heapro/dag416	I4, I5
357	Shin, N. Y., & Lim, Y.-J. (2019). Contribution of self-compassion to positive mental health among Korean university students. *International Journal of Psychology, 54*(6), 800-806. https://doi.org/10.1002/ijop.12527	I3, I4, I5, E5
358	Silva, M. P., Vettore, M. V., Rebelo, M. A. B., Rebelo Vieira, J. M., Herkrath, A., Queiroz, A. C., Herkrath, F. J., & Pereira, J. V. (2020). Clinical Consequences of Untreated Dental Caries, Individual Characteristics, and Environmental Factors on Self-Reported Oral Health	I4, I5

Number	Reference	Criteria
	Measures in Adolescents: A Follow-Up Prevalence Study. *Caries Research, 54*(2), 176-184. https://doi.org/10.1159/000506438	
359	Siqueira, J., Fernandes, N. M., & Moreira-Almeida, A. (2019). Association between religiosity and happiness in patients with chronic kidney disease on hemodialysis. *Jornal Brasileiro de Nefrologia, 41*(1), 22-28. https://doi.org/10.1590/2175-8239-JBN-2018-0096	I5
360	Skrondal, T. F., Bache-Gabrielsen, T., & Aune, I. (2020). All that I need exists within me: A qualitative study of nulliparous Norwegian women's experiences with planned home birth. *Midwifery, 86*, 102705. https://doi.org/10.1016/j.midw.2020.102705	I3, I4, I5
361	Smith, D. F. (2002). Functional salutogenic mechanisms of the brain. *Perspectives in Biology and Medicine, 45*(3), 319-328. https://doi.org/10.1353/pbm.2002.0058	I3, I4, I5
362	Snell, D. L., Martin, R., Surgenor, L. J., Siegert, R. J., & Hay-Smith, E. J. C. (2017). What's wrong with me? Seeking a coherent understanding of recovery after mild traumatic brain injury. *Disability and Rehabilitation, 39*(19), 1968-1975. https://doi.org/10.1080/09638288.2016.1213895	I3, I4, I5
363	Snyder, M. A. (2016). *Sense of coherence and daily spiritual experience among pregnant, post-partum, and parenting women in recovery from substance abuse: An expressive arts group therapy intervention.* [Doctoral dissertation, University of North Carolina at Charlotte]. ProQuest Dissertations and Theses Global.	I5
364	Soares, T. R. C., Lenzi, M. M., Leite, I. M., Muniz Loureiro, J., Leão, A. T. T., Pomarico, L., . . . Maia, L. C. (2020). Oral status, sense of coherence, religious-spiritual coping, socio-economic characteristics, and quality of life in young patients. *International Journal of Paediatric Dentistry, 30*(2), 171-180. https://doi.org/10.1111/ipd.12594	I5
365	Sobo, E. J. (2015). Salutogenic Education? Movement and Whole Child Health in a Waldorf (Steiner) School. *Medical Anthropology Quarterly, 29*(2), 137-156. https://doi.org/10.1111/maq.12140	I3, I4, I5
366	Sokar, S., Greenbaum, C. W., & Haj-Yahia, M. M. (2023). Exposure to parental violence during childhood and later psychological distress among Arab adults in Israel: The role of gender and sense of coherence. *Journal of Interpersonal Violence, 38*(1/2), NP588-NP612. https://doi.org/10.1177/08862605221082741	I4, I5
367	Starnino, V. R., & Sullivan, W. P. (2016). Early trauma and serious mental illness: what role does spirituality play?. *Mental Health, Religion & Culture, 19*(10), 1094-1117. https://doi.org/10.1080/13674676.2017.1320368	I3, I4, I5
368	Stefanaki, I. N., Shea, S., Linardakis, M., Symvoulakis, E. K., Wynyard, R., & Lionis, C. (2014). Exploring the association of sense of coherence, and spiritual and religious beliefs in a rural population group on the Island of Crete, Greece. *International Journal of Psychiatry in Medicine, 47*(3), 207-230. https://doi.org/10.2190/PM.47.3.c	I5
369	Steffen, E., & Coyle, A. (2011). Sense of presence experiences and meaning-making in bereavement: A qualitative analysis. *Death Studies, 35*(7), 579-609.	I3, I4, I5
370	Stephens, N. M., Fryberg, S. A., Markus, H. R., & Hamedani, M. G. (2013). Who explains Hurricane Katrina and the Chilean earthquake as an act of God? The experience of extreme hardship predicts religious meaning-making. *Journal of Cross-Cultural Psychology, 44*(4), 606-619. https://doi.org/10.1177/0022022112454330	I3, I4, I5
371	Stern, A. (2018). Sense of place, sense of self. [Doctoral dissertation, Saybrook University]. ProQuest Dissertations and Theses Global.	I3, I4, I5

Number	Reference	Criteria
372	Stoner, C. R., Orrell, M., & Spector, A. (2015). Review of positive psychology outcome measures for chronic illness, traumatic brain injury and older adults: Adaptability in dementia? *Dementia and Geriatric Cognitive Disorders, 40*(5-6), 340-357. https://doi.org/10.1159/000439044	I3, I4, I5
373	Stoyanova, K., & Stoyanov, D. S. (2021). Sense of Coherence and burnout in healthcare professionals in the COVID-19 era. *Frontiers in Psychiatry, 12*, 709587. https://doi.org/10.3389/fpsyt.2021.709587	I4, I5
374	Strang, S., & Strang, P. (2001). Spiritual thoughts, coping and 'sense of coherence' in brain tumour patients and their spouses. *Palliative Medicine, 15*(2), 127-134.	I3, I4, I5
375	Strauser, D. R., & Lustig, D. C. (2003). The moderating effect of sense of coherence on work adjustment. *Journal of Employment Counseling, 40*(3), 129-140. https://doi.org/10.1002/j.2161-1920.2003.tb00863.x	I4, I5
376	Strhan, A. (2013). The metropolis and evangelical life: Coherence and fragmentation in the "lost city of London". *Religion, 43*(3), 331-352. https://doi.org/10.1080/0048721x.2013.798164	I3, I4, I5
377	Strinnholm, S., Gustafson, Y., & Niklasson, J. (2019). Depressive disorders and religious engagement in very old people. *Gerontology & Geriatric Medicine, 5*, 1-9. https://doi.org/10.1177/2333721419846576	I3, I4, I5
378	Strümpfer, D. J. W., & Bands, J. (1996). Stress among clergy: An exploratory study on South African Anglican priests. *South African Journal of Psychology, 26*(2), 67-75. https://doi.org/10.1177/008124639602600201	I4, I5
379	Sundararajan-Reddy, S. (2005). *The relationship of spirituality to resilience in adolescents.* [Doctoral dissertation, State University of New Jersey]. ProQuest Dissertations and Theses Global.	E5
380	Sullivan, W. P. (1998). Recoiling, regrouping, and recovering: First-person accounts of the role of spirituality in the course of serious mental illness. *New Directions for Mental Health Services, 80*, 25-33. https://doi.org/10.1002/yd.23319988005	I3, I4, I5
381	Suraj, S., & Singh, A. (2011). Study of sense of coherence health promoting behavior in north Indian students. *The Indian Journal of Medical Research, 134*(5), 645-652. https://doi.org/10.4103/0971-5916.90989	I5
382	Świątoniowska-Lonc, N., Tański, W., Polański, J., Jankowska-Polańska, B., & Mazur, G. (2021). Psychosocial determinants of treatment adherence in patients with type 2 diabetes: A Review. *Diabetes, Metabolic Syndrome and Obesity, 14*, 2701-2715. https://doi.org/10.2147/dmso.S308322	I3, I4, I5
383	Tagamets, M. A., Cortes, C. R., Griego, J. A., & Elvevåg, B. (2014). Neural correlates of the relationship between discourse coherence and sensory monitoring in schizophrenia. *Cortex, 55*, 77-87. https://doi.org/10.1016/j.cortex.2013.06.011	I3, I4, I5
384	Tagay, S., Senf, W., Schöpper, N., Mewes, R., Bockisch, A., & Görges, R. (2007). Protektive Faktoren für Angst und Depression bei Schilddrüsenkarzinompatienten [Protective factors for anxiety and depression in thyroid cancer patients]. *Zeitschrift für Psychosomatische Medizin und Psychotherapie, 53*(1), 62-74.	I5
385	Temane, L., Khumalo, I. P., & Wissing, M. P. (2014). Validation of the Meaning in Life Questionnaire in a South African context. *Journal of Psychology in Africa, 24*(1), 51-60.	I5
386	Temane, Q. M., & Wissing, M. P. (2006). The role of spirituality as a mediator for psychological well-being across different contexts. *South*	I5

Number	Reference	Criteria
	African Journal of Psychology, 36(3), 582-597. https://doi.org/10.1177/008124630603600309	
387	Teut, M., Besch, F., Witt, C. M., & Stöckigt, B. (2019). Perceived outcomes of spiritual healing: Results from a prospective case series. *Complementary Medicine Research, 26*(4), 265-275. https://doi.org/10.1159/000496736	I5, E3
388	Teut, M., Stöckigt, B., Holmberg, C., Besch, F., Witt, C. M., & Jeserich, F. (2014). Perceived outcomes of spiritual healing and explanations: A qualitative study on the perspectives of German healers and their clients. *BMC Complementary and Alternative Medicine, 14,* Article 240. https://doi.org/http://dx.doi.org/10.1186/1472-6882-14-240	I3, I4, I5
389	Thelu, M., Webster, B., Jones, K., & Orrell, M. (2022). A cross sectional survey on UK older adult's attitudes to ageing, dementia and positive psychology attributes. *BMC Geriatrics, 22*(1), 837. https://doi.org/10.1186/s12877-022-03539-w	I4, I5
390	Thoma, M. V., Rohner, S. L., Heim, E., Hermann, R. M., Roos, M., Evangelista, K. W. M., Nater, U. M., & Höltge, J. (2022). Identifying well-being profiles and resilience characteristics in ex-members of fundamentalist Christian faith communities. *Stress and Health, 38*(5), 1058-1069. https://doi.org/10.1002/smi.3157	I3, I4, I5
391	Tilles-Tirkkonen, T., Suominen, S., Liukkonen, J., Poutanen, K., & Karhunen, L. (2015). Determinants of a regular intake of a nutritionally balanced school lunch among 10-17-year-old schoolchildren with special reference to sense of coherence. *Journal of Human Nutrition and, 28*(1), 56-63. https://doi.org/10.1111/jhn.12221	I4, I5
392	Tilus, M. R. (2003). *The roles of spirituality and adult romantic attachment in responses to exposure to trauma and the development of post-traumatic stress disorder in nonclinical military couples.* [Doctoral dissertation, Alliant International University]. ProQuest Dissertations and Theses Global.	I3, I5
393	Ullmann, E., Licinio, J., Barthel, A., Petrowski, K., Oratovski, B., Stalder, T., . . . Bornstein, S. R. (2017). Circumcision does not alter long-term glucocorticoids accumulation or psychological effects associated with trauma- and stressor-related disorders. *Translational Psychiatry, 2017*. https://doi.org/10.1038/tp.2017.23	I4, I5
394	Unterrainer, H.-F. (2005). *Das Konstrukt der religiös-spirituellen Befindlichkeit: Testpsychologische Operationalisierung im Kontext von seelischer Gesundheits- und Krankheitsverarbeitung* [Doctoral dissertation].	E2
395	Unterrainer, H.-F. (2010). *Seelenfinsternis?: Struktur und Inhalt der Gottesbeziehung im klinisch-psychiatrischen Feld [Structure and content of a relationship with God in clinical-psychiatric field*] Waxmann.	E2
396	Unterrainer, H. F., & Kapfhammer, H. P. (2014). Religiös/spirituelles Befinden bei psychisch Kranken II: Die Entwicklung einer Kurzskala und Vergleichswerte von klinisch-psychiatrischen Gruppen und gesunden Kontrollpersonen [Religious/spiritual well-being in mentally ill persons II: The development of a short scale and comparison scores for clinical psychiatric groups and healthy controls]. *Neuropsychiatrie, 28*(2), 49-55. https://doi.org/http://dx.doi.org/10.1007/s40211-013-0083-5	I3, I5
397	Unterrainer, H. F., Huber, H. P., Sorgo, I. M., Collicutt, J., & Fink, A. (2011). Dimensions of religious/spiritual well-being and schizotypal personality. *Personality and Individual Differences, 51*(3), 360-364. https://doi.org/10.1016/j.paid.2011.04.007	E2
398	Unterrainer, H. F., Schoeggl, H., Fink, A., Neuper, C., & Kapfhammer, H. P. (2012). Soul darkness? Dimensions of religious/spiritual well-being among mood-disordered inpatients compared to healthy controls. *Psychopathology, 45*(5), 310-316.	I3, I5

Number	Reference	Criteria
	https://doi.org/10.1159/000336050	
399	Unterrainer, H., Lewis, A., & Fink, A. (2014). Religious/Spiritual Well-Being, Personality and Mental Health: A Review of Results and Conceptual Issues. *Journal of Religion & Health, 53*(2), 382-392. https://doi.org/10.1007/s10943-012-9642-5	E2
400	Unterrainer, H.-F., Ladenhauf, K. H., Wallner-Liebmann, S. J., & Fink, A. (2011). Different types of religious/spiritual well-being in relation to personality and subjective well-being. *International Journal for the Psychology of Religion, 21*(2), 115-126. https://doi.org/10.1080/10508619.2011.557003	I5, E2
401	van Den Berg, H. A. (2018). Darwin endures, despite disparagement. *Science Progress, 101*(1), 32-51. https://doi.org/https://dx.doi.org/10.3184/003685018X15166188312386	I3, I4, I5
402	van der Westhuizen, S., de Beer, M., & Bekwa, N. (2011). Psychological strengths as predictors of postgraduate students' academic achievement. *Journal of Psychology in Africa, 21*(3), 473-478.	E5
403	van Leeuwen, C. M. C., Kraaijeveld, S., Lindeman, E., & Post, M. W. M. (2012). Associations between psychological factors and quality of life ratings in persons with spinal cord injury: A systematic review. *Spinal Cord, 50*(3), 174-187. https://doi.org/10.1038/sc.2011.120	I3, I4, I5
404	van Mulukom, V. (2017). Remembering religious rituals: Autobiographical memories of high-arousal religious rituals considered from a narrative processing perspective. *Religion Brain & Behavior, 7*(3), 191-205. https://doi.org/10.1080/2153599x.2016.1232304	I3, I4, I5
405	Ventegodt, S. (2013). On the paradigm of consciousness-based medicine and quality of life as medicine. In J. Merrick (Ed.), *Alternative medicine research yearbook 2012*. (pp. 73-83). Nova Biomedical Books.	I3, I4, I5
406	Ventegodt, S., Kandel, I., & Merrick, J. (2007). Clinical holistic medicine: Factors influencing the therapeutic decision-making. From academic knowledge to emotional intelligence and spiritual "crazy" wisdom. *The Scientific World Journal, 7*, 1932-1949. https://doi.org/10.1100/tsw.2007.303	I3, I4, I5
407	Ventegodt, S., Kandel, I., & Merrick, J. (2007). Clinical holistic medicine (mindful short-term psychodynamic psychotherapy complimented with bodywork) in the treatment of schizophrenia (ICD10-F20/DSM-IV Code 295) and other psychotic mental diseases. *The Scientific World Journal, 7*, Article ID 957859. https://doi.org/10.1100/tsw.2007.298	I4, I5, I3
408	Ventegodt, S., & Merrick, J. (2013). Quality of life as medicine. In J. Merrick (Ed.), *Alternative medicine yearbook, 2011*. (pp. 175-185). Nova Biomedical Books.	I3, I4, I5
409	Ventegodt, S., Solheim, E., Saunte, M. E., Morad, M., Kandel, I., & Merrick, J. (2004). Clinical holistic medicine: Metastatic cancer. *The Scientific World Journal, 4*, 913-935. https://doi.org/10.1100/tsw.2004.189	I3, I4, I5
410	Veronese, G., Dhaouadi, Y., & Afana, A. (2021). Rethinking sense of coherence: Perceptions of comprehensibility, manageability, and meaningfulness in a group of Palestinian health care providers operating in the West Bank and Israel. *Transcultural Psychiatry, 58*(1), 38-51, Article 1363461520941386. https://doi.org/10.1177/1363461520941386	I3, I4, I5
411	Veronese, G., Fiore, F., Castiglioni, M., el Kawaja, H., & Said, M. (2013). Can sense of coherence moderate traumatic reactions? A cross-sectional study of Palestinian helpers operating in war contexts. *British Journal of Social Work, 43*(4), 651-666.	I4, I5

Number	Reference	Criteria
	https://doi.org/10.1093/bjsw/bcs005	
412	Verres, R. (1997). Etüden zur Gesundheit [Health attitudes]. In H. H. Bartsch & J. Bengel (Eds.), *Salutogenese in der Onkologie* (pp. 20-36). Karger.	I3, I4, I5
413	Vogel, R. (2010). *Lebenssinn in schweren Erkrankungen älterer Menschen. Eine empirische Untersuchung über Selbsttranszendenz. Sinnerfüllung, Sinnkrise im Alter* [Meaning of life in severe illness in the elderly: An empirical investigation of self-transcendence, meaning fulfillment, meaning crisis in old age]. [Doctoral dissertation, University of Heidelberg]. HeiDOK: Heidelberger Dokumentenserver. https://archiv.ub.uni-heidelberg.de/volltextserver/10929/	I4, I5, I3
414	von Humboldt, S., & Leal, I. (2017). Correlates of adjustment to aging among the young-old and the oldest-old: A comparative analysis. *Educational Gerontology, 43*(4), 175-185. https://doi.org/10.1080/03601277.2016.1272355	I4, I5
415	von Humboldt, S., Leal, I., & Pimenta, F. (2014a). Living well in later life: The influence of sense of coherence, and socio-demographic, lifestyle and health-related factors on older adults' satisfaction with life. *Applied Research in Quality of Life, 9*(3), 631-642. https://doi.org/10.1007/s11482-013-9262-6	I4, I5
416	von Humboldt, S., Leal, I., & Pimenta, F. (2014b). What predicts older adults' adjustment to aging in later life? The impact of sense of coherence, subjective well-being, and sociodemographic, lifestyle, and health-related factors. *Educational Gerontology, 40*(9), 641-654. https://doi.org/10.1080/03601277.2013.860757	I4, I5
417	von Humboldt, S., Leal, I., & Pimenta, F. (2015). Sense of coherence, sociodemographic, lifestyle, and health-related factors in older adults' subjective well-being. *International Journal of Gerontology, 9*(1), 15-19.	E4
418	von Humboldt, S., Leal, I., Pimenta, F., & Maroco, J. (2014). Assessing adjustment to aging: A validation study for the Adjustment to Aging Scale (AtAS). *Social Indicators Research, 119*(1), 455-472.	E5
419	Wahab, S. N. B. A., Mordiffi, S. Z., Ang, E., & Lopez, V. (2017). Light at the end of the tunnel: New graduate nurses' accounts of resilience: A qualitative study using Photovoice. *Nurse Education Today, 52*, 43-49. https://doi.org/10.1016/j.nedt.2017.02.007	I3, I4, I5
420	Waite, P. J., Hawks, S. R., & Gast, J. A. (1999). The correlation between spiritual well-being and health behaviors. *American Journal of Health Promotion, 13*(3), 159-162. https://doi.org/10.4278/0890-1171-13.3.159	I3, I5
421	Walach, H., Ferrari, M. L. G., Sauer, S., & Kohls, N. (2012). Mind-body practices in integrative medicine. *Religions, 3*(1), 50-81. https://doi.org/10.3390/rel3010050	I3, I4, I5
422	Waldrop, D. P., & Rinfrette, E. S. (2009). Can short hospice enrollment be long enough? Comparing the perspectives of hospice professionals and family caregivers. *Palliative & Supportive Care, 7*(1), 37-47. https://doi.org/10.1017/S1478951509000066	I3, I4, I5
423	Walsh, F. (1996). The concept of family resilience: crisis and challenge. *Family Process, 35*(3), 261-281. https://doi.org/10.1111/j.1545-5300.1996.00261.x	I3, I4, I5
424	Watkins, C. C., Kanu, I. K., Hamilton, J. B., Kozachik, S. L., & Gaston-Johansson, F. (2017). Differences in coping among African American women with breast cancer and triple-negative breast cancer. *Oncology Nursing Forum, 44*(6), 689-702. https://doi.org/10.1188/17.ONF.689-702	E5

Number	Reference	Criteria
425	Webb, C., Smith, A., Orrell, M., & Jones, K. A. (2022). Positive psychology and attitudes to ageing in people aged 50 and over in the United Kingdom. *Aging & Mental Health*, 1-7. https://doi.org/10.1080/13607863.2022.2129587	I4, I5
426	Wenning, B. (2021). An ethnographic perspective of well-being, salutogenesis and meaning making among refugees and asylum seekers in the Gambia and the United Kingdom. *Social Sciences, 10*(9), 16, Article 324. https://doi.org/10.3390/socsci10090324	I3, I4, I5
427	Wiesmann, U., & Hannich, H. J. (2013). The contribution of resistance resources and sense of coherence to life satisfaction in older age. *Journal of Happiness Studies, 14*(3), 911-928. https://doi.org/10.1007/s10902-012-9361-3	I4, I5
428	Wijesinghe, S. (2013). *Role of Buddhist spiritual practice in the lives and health of Buddhist nuns living with a chronic illness in Sri Lanka.* [Doctoral dissertation, University of New Mexico]. ProQuest Dissertations and Theses Global.	I3, I4, I5
429	Wijesinghe, S., & Parshall, M. B. (2016). Impermanence and sense of coherence: Lessons learned from the adaptive behaviors of Sri Lankan Buddhist nuns with a chronic illness. *Journal of Transcultural Nursing, 27*(2), 157-165. https://doi.org/10.1177/1043659614545402	I3, I4, I5
430	Wiklund, L. (2008). Existential aspects of living with addiction - Part II: Caring needs. A hermeneutic expansion of qualitative findings. *Journal of Clinical Nursing, 17*(18), 2435-2443. https://doi.org/https://dx.doi.org/10.1111/j.1365-2702.2008.02357.x	I3, I4, I5
431	Wilson, A. R., Tiwari, T., Thomas, J. F., Henderson, W. G., Braun, P. A., & Albino, J. (2020). Validation of psychosocial measures assessing American Indian parental beliefs related to control over their children's oral health. *International Journal of Environmental Research and Public Health, 17*(2). https://doi.org/10.3390/ijerph17020403	I4, I5
432	Wilson, A., & Mittelmark, M. B. (2013). Resources for adjusting well to work migration: Women from Northern Ghana working in Head Porterage in Greater Accra. *Africa Today, 59*(4), 25-38. https://doi.org/10.2979/africatoday.59.4.25	I3, I4, I5
433	Winger, J. G., Adams, R. N., & Mosher, C. E. (2016). Relations of meaning in life and sense of coherence to distress in cancer patients: A meta-analysis. *Psycho-Oncology, 25*(1), 2-10. https://doi.org/10.1002/pon.3798	I4, I5
434	Wong, I. Y. T., Hawes, D. J., & Dar-Nimrod, I. (2019). Illness representations among adolescents with attention deficit hyperactivity disorder: associations with quality of life, coping, and treatment adherence. *Heliyon, 5*(10), e02705. https://doi.org/10.1016/j.heliyon.2019.e02705	I3, I4, I5
435	Woodbine, L. (2016). *Coming home: Post incarcerated lived experience of a caring community.* [Doctoral dissertation, Fordham University]. ProQuest Dissertations and Theses Global.	I4, I5
436	Wrbsky, P. M. (2000). *Family meaning attribution in the health-illness transition to preterm birth.* [Doctoral dissertation, University of Minnesota]. ProQuest Dissertations and Theses Global.	I3
437	Wyatt, G. (2013). Ein praktischer spiritueller Weg: Das Persönliche, das Berufliche und das Gesellschaftliche verbinden [A practical spiritual path: Connecting the personal, the professional, and the societal]. *Person, 17*(2), 130-138.	I3, I4, I5
438	Xiu, D., Mc Gee, S. L., & Maercker, A. (2018). Sense of coherence and posttraumatic growth: The moderating role of value orientation in Chinese and Swiss bereaved parents. *Journal of Loss & Trauma, 23*(3), 259-270. https://doi.org/10.1080/15325024.2018.1436120	I4, I5, I3
439	Yasuma, N., Watanabe, K., Nishi, D., & Kawakami, N. (2020). Personal values in adolescence and sense of coherence in adulthood: A cross-	I4, I5

Number	Reference	Criteria
	sectional study based on a retrospective recall. *Neuropsychopharmacology Reports, 40*(3), 262-267. https://doi.org/10.1002/npr2.12111	
440	Ying, Y.-W. (2008). Variation in personal competence and mental health between entering and graduating MSW students: The contribution of mindfulness. *Journal of Religion & Spirituality in Social Work: Social Thought, 27*(4), 405-422. https://doi.org/10.1080/15426430802347347	E5
441	Ying, Y.-W. (2009). Contribution of self-compassion to competence and mental health in social work students. *Journal of Social Work Education, 45*(2), 309-323. https://doi.org/10.5175/JSWE.2009.200700072	E5
442	Ying, Y.-W., Akutsu, P. D., Zhang, X. L., & Huang, L. N. (1997). Psychological dysfunction in Southeast Asian refugees as mediated by sense of coherence. *American Journal of Community Psychology, 25*(6), 839-859. https://doi.org/10.1023/a:1022217330005	I4, I5
443	Ziarko, M., Mojs, E., Kaczmarek, L. D., Warchol-Biedermann, K., Malak, R., Lisinski, P., & Samborski, W. (2015). Do urban and rural residents living in Poland differ in their ways of coping with chronic diseases? *European Review for Medical and Pharmacological Sciences, 19*(22), 4227-4234.	I5
444	Zwack, J., Bodenstein, U., Mundle, G., & Schweitzer, J. (2012). Pathogenetische und salutogenetische Aspekte der Ärztegesundheit: Eine qualitative Katamnese betroffener Ärzte [Pathogenetic and salutogenetic aspects of physicians' health]. *Psychiatrische Praxis, 39*(4), 181-188. https://doi.org/10.1055/s-0031-1298975	I3, I4, I5

Note. The abbreviations in the third column "Criteria" refer to the five inclusion (I) and six exclusion criteria (E) of the meta-analysis; for example: I5 = inclusion criteria 5.
