# Supplementary material for: Sense of coherence and religion/spirituality: A systematic review and meta-analysis based on a methodical classification of instruments measuring religion/spirituality
Source: PLoS One. 2023 Aug 3;18(8):e0289203. doi: 10.1371/journal.pone.0289203 (PMC10399782; doi:10.1371/journal.pone.0289203)
Supplement: S9 Table — List of all measurement instruments that cannot be considered as measures of R/S according to our (heuristic) definition of R/S including documentation of the item-wise classification process and a rationale for the decision. (PDF) [file pone.0289203.s013.pdf]

S14 Table. List of Scales that Have not Been Shown to be R/S Measurement Tools According to our Definition.

Scale	Scale author(s)	Study	Classification	Rationale
**Dogmatism**, total scale (40 items)	Rokeach (1956)	Britt et al. (2009)	X, X, X, X, X, X, X, X, R, X, X, X, X, X, X, X, X, X, X, X, X, X, X, X, R, X, X, X, X, X, X, X, X, X, X, X, X, X<br>T: 5%<br>R: 5%<br>S: 0%	The scale primarily measures dogmatic positions in the area of political opinion. Only two of the forty items relate to the religious sphere.
**Functional Assessment of Chronic Illness Therapy—Spiritual Well-Being**, total scale (12 items) with two sub-scales: 1. Meaning / Peace (8 items), 2. Faith (4 items)	Peterman et al. (2002)	Meghani et al. (2012); Watkins et al. (2017); Iani et al. (2019)	(X), (X), X, (X), S, (S), (S), S, SR, SR, SR, (X)<br>T: 58%<br>R: 25%<br>S: 58%	Although more than half of the items refer to spiritual aspects, many items lack references to transcendence and therefore tend to measure emotional well-being in general.
**Meaning / Peace**, sub-scale (8 items) of the Functional Assessment of Chronic Illness Therapy—Spiritual Well-Being	Peterman et al. (2002)	Meghani et al. (2012)	(X), (X), X, (X), S, (S), (S), S<br>T: 50%<br>R: 0%<br>S: 50%	Although half of the items refer to spiritual aspects, the other half do not show clear references to transcendence. Because the sub-scale also focuses strongly on emotional well-being in general, it does not qualify as an R/S measurement instrument.
**Hope Orientation Measure**, total scale (57 items)	Maree et al. (2008)	Van der Westhuizen et al. (2011)	NA	Based on the description of the factors and individual sample items, it is clear that the construct hope is not conceived and measured as a religious / spiritual concept in this measurement instrument.
**Mindful Attention Awareness Scale**, total scale (15 items)	Brown & Ryan (2003)	Ying (2008); Oluyinka (2011)	X, X, X, X, X, X, X, X, X, X, X, X, X, X, X<br>T: 0%<br>R: 0%<br>S: 0%	The scale measures mindfulness as a form of conscious action and being present, but does not include transcendent aspects or frame the concept of mindfulness in religious or spiritual terms.

Scale	Scale author(s)	Study	Classification	Rationale
**Multidimensional Instrument for Measuring Religious / Spiritual Well-Being**, total scale (48 items) with X sub-scales:	Unterrainer et al. (2010)	Unterrainer et al. (2010); Unterrainer et al. (2013); Berger et al. (2016); Wenzl et al. (2021)	RS, X, (X), S, X, X, R, X, (X), RS, X, X, R, X, (X), S, X, X, R, X, (X), S, X, X, R, X, (X), SR, X, X, R, X, (X), (S), (RS), X, R, X, (X), S, (X), X, RS, X, (X), (S), (RS), (S)<br>T: 40%<br>R: 25%<br>S: 27%	The total scale is a good measure of emotional well-being, including both religious / spiritual and existential aspects. However, since only 40% of the items have a reference to something / someone transcendent, the total scale does not qualify as a measurement instrument for R/S in the sense defined here.
**Experiences of Sense and Meaning**, sub-scale (8 items) of the Multidimensional Instrument for Measuring Religious / Spiritual Well-Being	Unterrainer et al. (2010)	Unterrainer et al. (2010); Unterrainer et al. (2013); Berger et al. (2016); Wenzl et al. (2021)	X, X, X, X, X, X, X, (S)<br>T: 13%<br>R: 0%<br>S: 13%	The scale measures positive emotional experiences, but does not ask whether meaning / sense making is related to transcendent concepts.
**Forgiveness**, sub-scale (8 items) of the Multidimensional Instrument for Measuring Religious / Spiritual Well-Being	Unterrainer et al. (2010)	Unterrainer et al. (2010); Unterrainer et al. (2013); Berger et al. (2016); Wenzl et al. (2021)	(X), (X), (X), (X), (X), (X), (X), (X)<br>T: 0%<br>R: 0%<br>S: 0%	As long as respondents do not view the concept of forgiveness as essentially religious / spiritual, the scale measures the ability to forgive without reference to transcendence.
**Hope Immanent**, sub-scale (8 items) of the Multidimensional Instrument for Measuring Religious / Spiritual Well-Being	Unterrainer et al. (2010)	Unterrainer et al. (2010); Unterrainer et al. (2013); Berger et al. (2016); Wenzl et al. (2021)	(X), (X), (X), (X), (X), (X), (X), (X)<br>T: 0%<br>R: 0%<br>S: 0%	The scale is a measure of optimism about the future, but does not include transcendental aspects.
**Hope Transcendent**, sub-scale (8 items) of the Multidimensional Instrument for Measuring Religious / Spiritual Well-Being	Unterrainer et al. (2010)	Unterrainer et al. (2010); Unterrainer et al. (2013); Berger et al. (2016); Wenzl et al. (2021)	X, X, X, X, X, (RS), X, (RS)<br>T: 25%<br>R: 25%<br>S: 25%	The scale primarily measures the emotional handling of dying and death, but only to a small extent religious / spiritual aspects (e.g., postmortem beliefs).
**Spiritual Wellness**, sub-scale (6 items) of the Perceived Wellness Scale	Adams (1995)	Adams (1995); Adams et al. (2000)	(S), X, X, (S), X, (S)<br>T: 50%	The sub-scale is actually a measure of sense of purpose and not a measure of spiritual

Scale	Scale author(s)	Study	Classification	Rationale
			R: 0%<br>S: 50%	wellness. It makes no reference to anything or anyone transcendent. However, some items can be indirectly related to spirituality, as they use terms such as "real purpose" or "sense of mission".
**Purpose in Life Test**, total scale (20 items)	Crumbaugh & Maholick (1964)	Mak et al. (2017); Haugan (2021)	X, X, X, (S), X, X, X, X, X, X, (S), (X), X, (X), (X), (X), (S), (X), X, (S)<br>T: 20%<br>R: 0%<br>S: 20%	In answering the questions, a religious or spiritual interpretation of the items may play a role. This may be reinforced, for example, by the use of the phrase "mission in life". However, references to something or someone transcendent are not made.
**QE Health Scale**, total scale (28 items)	Faull & Hills (2007)	Faull & Hills (2007)	X, (S), X, X, X, X, (S), (S), X, S, X, (X), X, X, SR, S, (S), X, X, S, (S), (S), X, X, X, X, X, X<br>T: 36%<br>R: 4%<br>S: 36%	Few items have a direct or indirect reference to something or someone transcendent. Many items rather ask about social, emotional and personal resources in the disease process and do not show any reference to transcendence.
**Religious Quest**, sub-scale (3 items) of a questionnaire block on R/S	Zehnder Grob (2015)	Zehnder Grob (2015)	(X), (S), R<br>T: 67%<br>R: 33%<br>S: 33%	The first question measures how often people think about suffering in the world. This question does not have to include a transcendental worldview or be related to R/S. The second item, which asks about the meaning of life, can be related to spirituality, but it too does not have to be related to R/S.
**Self-compassion**, total scale (26 items) with six sub-scales: 1. Self-Kindness (5 items), 2. Self-Judgment (5 items), 3. Common Humanity (4 items), 4. Isolation (4 items), 5.	Neff (2003)	Ying (2009); Shin & Lim (2019)	X, X, X, X, X, X, X, X, X, X, X, (X), X, X, X, (X), X, X, X, X, (X), (X), X, X, X, X<br>T: 0%<br>R: 0%	Due to the proximity to Buddhist concepts of self and mindfulness, the scale could have included religious / spiritual aspects. However, none of the items has a reference to transcendence.

Scale	Scale author(s)	Study	Classification	Rationale
Mindfulness (4 items), 6. Over-Identification (4 items)			S: 0%	
**Self-Transcendence Scale**, total scale (15 items)	Reed (2009)	Nygren et al. (2005); Moe et al. (2013); Lundman et al. (2015); Haugan et al. (2019); Haugan (2021)	X, X, X, X, X, (X), (X), (X), X, (X), (X), S, X, X, (X)<br>T: 7%<br>R: 0%<br>S: 7%	Apart from one item that explicitly refers to spiritual beliefs, the questions do not refer to transcendence. The underlying concept of self-transcendence is not placed in a religious / spiritual framework.
**Spiritual Behavior, Attitude and Sensitivity Test**, total scale (29 items) with three sub-scales: 1. Spiritual Behavior (9 items), 2. Spiritual Attitude (10 items), Spiritual Sensivity (11 items)	Ozaki (2005)	Ozaki (2005)	(X), X, (X), X, X, (X), X, (S), (X), (S), X, X, (X), (X), X, X, X, X, (S), SR, S, S, R, (X), (S), SR, S, X, (S)<br>T: 38%<br>R: 10%<br>S: 35%	Only the Spiritual Sensitivity sub-scale can be considered an instrument measuring R/S according to our definition. Since the other two sub-scales do not show any references to something or someone transcendent, the overall scale also disqualifies as a R/S measure in our sense.
**Spiritual Attitude**, sub-scale (9 items) of the Spiritual Behavior, Attitude and Sensitivity Test	Ozaki (2005)	Ozaki (2005)	(S), X, X, (X), (X), X, X, X, X<br>T: 11%<br>R: 0%<br>S: 11%	The scale most closely measures life satisfaction and has no direct references to anything or anyone transcendent.
**Spiritual Behavior**, sub-scale (9 items) of the Spiritual Behavior, Attitude and Sensitivity Test	Ozaki (2005)	Ozaki (2005)	(X), X, (X), X, X, (X), X, (S), (X)<br>T: 11%<br>R: 0%<br>S: 11%	Only one item can be linked to spirituality through the theme of meaning / purpose, the rest of the questions rather measure whether a person is doing what he / she thinks should / must be done.
**Spiritual Transcendence Scale – Short Form**, total scale (9 items) with three sub-scales: 1. Prayer Fulfillment (3 items), 2. Universality (3 items), 3. Connectedness (3 items)	Piedmont (2010)	Wilkins et al. (2012); Piedmont et al. (2014)	SR, (X), (X), SR, (X), S, (X), SR, S<br>T: 56%<br>R: 33%<br>S: 56%	While the 'Prayer Fulfillment' subscale can clearly be classified as a measurement instrument for R/S according to our definition, references to transcendence are missing in many of the items of the other two subscales.

Scale	Scale author(s)	Study	Classification	Rationale
**Connectedness**, sub-scale (3 items) of the Spiritual Transcendence Scale – Short Form	Piedmont (2010)	Wilkins et al. (2012); Piedmont et al. (2014)	(X), (X), (X)<br>T: 0%<br>R: 0%<br>S: 0%	The items measure whether the respondent has a lasting connection to deceased persons. Since the questions do not focus on afterlife concepts, but rather on emotional ties, transcendental aspects are secondary.
**Universality**, sub-scale (3 items) of the Spiritual Transcendence Scale – Short Form	Piedmont (2010)	Wilkins et al. (2012); Piedmont et al. (2014)	S, (X), S<br>T: 67%<br>R: 0%<br>S: 67%	The adjective 'higher' in two of the three items refers to something transcendent. It is missing in the second item, which can also be agreed to without any reference to transcendence.
**Spiritual Well-Being Scale** (20 items) with two sub-scales: 1. Religious Well-Being (10 items), 2. Existential Well-Being (10 items)	Paloutzian & Ellison (1982)	Lee (1998); Darling et al. (2004); Sundararajan-Reddy (2005); Wissing et al. (2008)	R, (X), R, X, R, X, R, (X), R, X, R, X, R, X, R, X, R, (X), R, (S)<br>T: 55%<br>R: 50%<br>S: 5%	Since the sub-scale 'Existential Well-Being' is not assessed as a measurement tool for R/S, the total scale also disqualifies itself as a suitable measure for R/S.
**Existential Well-Being**, sub-scale (10 items) of the Spiritual Well-Being Scale	Paloutzian & Ellison (1982)	Lee (1998); Wissing et al. (2008)	(X), X, X, (X), X, X, X, X, (X), (S)<br>T: 10%<br>R: 0%<br>S: 10%	Except for one item that can possibly be interpreted in terms of a spiritual-religious horizon of meaning ("real purpose for my life"), all other questions measure emotional life satisfaction without reference to transcendence.
**Spirituality at Work**, total scale (18 items) with four sub-scales: 1. Engaging Work (7 items), Mystical Experience (5 items), Spiritual Connection (3 items), 4. Sense of Community (3 items)	Kinjerski (2013)	Zerach & Levin (2018)	(RS), (S), (X), (S), (S), (X), (X), (X), (X), (S), (S), (S), S, S, S, (X), (S), (X)<br>T: 61%<br>R: 6%<br>S: 61%	Although the overall scale covers many aspects of workplace spirituality, it includes too many other aspects (team spirit, flow experience, job satisfaction) that do not have a clear reference to transcendence.
**Engaging Work**, sub-scale (7 items) of the Spirituality at Work scale	Kinjerski (2013)	Zerach & Levin (2018)	(RS), (S), (X), (S), (S), (X), (X)	Some formulations suggest a spiritual / religious dimension to the meaning of work.

Scale	Scale author(s)	Study	Classification	Rationale
			T: 57%<br>R: 14%<br>S: 57%	This interpretation is also suggested by the explanation of the subscale ("meaningful work that has a higher purpose"). However, in our opinion, not all questions point to this transcendent level to a sufficient degree.
**Mystical Experience**, sub-scale (5 items) of the Spirituality at Work scale	Kinjerski (2013)	Zerach & Levin (2018)	(X), (X), (S), (S), (S)<br>T: 60%<br>R: 0%<br>S: 60%	Although some of the words used in the questions (e.g., blissful) can certainly be understood spiritually / religiously, this subscale is more suitable for measuring so-called flow experiences in the work process.
**Sense of Community**, sub-scale (3 items) of the Spirituality at Work scale	Kinjerski (2013)	Zerach & Levin (2018)	(X), (S), (X)<br>T: 33%<br>R: 0%<br>S: 33%	The transcendent dimension of work is not sufficiently evident in the questions. This subscale is more a measure of team spirit and a sense of belonging in the work context.
**Spiritual/Philosophical Resources**, sub-scale (12 items) of the Coping Resources Inventory	Hammer and Marting (1988)	Dåderman and De Colli (2014); Ghazinour et al. (2014)	SR/RS, (X), S, (X), (X), (R), R, RS/SR, S, (RS), (X), (X)<br>T: 58%<br>R: 42%<br>S: 33%	The scale includes some items that clearly refer to religion and/or spirituality. However, as the name of the scale suggests ("philosophical"), almost half of the questions do not refer to transcendence, but deal with general questions of meaning and philosophy.
**Zest and Spirituality**, sub-scale (5 items) of the Adjustment to Aging Scale	von Humboldt et al. (2014)	von Humboldt et al. (2014)	X, RS/SR, X, X, X<br>T: 20%<br>R: 20%<br>S: 20%	Four of the five items measure the aspect 'zest' and only one of the five items measures R/S.

References

Adams, T. B. (1995). *The conceptualization and measurement of wellness.* [Doctoral dissertation, University of Texas at Austin]. ProQuest Dissertations and Theses Global.

Adams, T. B., Bezner, J. R., Drabbs, M. E., Zambarano, R. J., & Steinhardt, M. A. (2000). Conceptualization and measurement of the spiritual and psychological dimensions of wellness in a college population. *Journal of American College Health, 48*(4), 165-173. doi: https://doi.org/10.1080/07448480009595692

Berger, D., Fink, A., Perez Gomez, M. M., Lewis, A., & Unterrainer, H.-F. (2016). The validation of a Spanish version of the multidimensional inventory of religious/spiritual well-being in Mexican college students. *The Spanish Journal of Psychology, 19*(e3), 1-11. doi: https://doi.org/10.1017/sjp.2016.9

Britt, T. W., Millard, M. R., Sundareswaran, P. T., & Moore, D. (2009). Personality variables predict strength-related attitude dimensions across objects. *Journal of Personality, 77*(3), 859-882. doi: https://doi.org/10.1111/j.1467-6494.2009.00567.x

Brown, K. W., & Ryan, R. M. (2003). The benefits of being present: Mindfulness and its role in psychological well-being. *Journal of Personality and Social Psychology, 84*(4), 822-848. doi: https://doi.org/10.1037/0022-3514.84.4.822

Crumbaugh, J., & Maholick, L. (1964). *Manual of instructions for the purpose and meaning in life test*: Psychometric Affiliates.

Dåderman, A. M., & De Colli, D. (2014). The significance of the sense of coherence for various coping resources in stress situations used by police officers in on-the-beat service. *International Journal of Occupational Medicine and Environmental Health, 27*(1), 3-15. doi: https://doi.org/10.2478/s13382-014-0227-2

Darling, C. A., Hill, E. W., & McWey, L. M. (2004). Understanding stress and quality of life for clergy and clergy spouses. *Stress and Health, 20*(5), 261-277.

Faull, K., & Hills, M. D. (2007). A spiritually-based measure of holistic health for those with disabilities: Development, preliminary reliability and validity assessment. *Disability and Rehabilitation, 29*(13), 999-1010. doi: https://doi.org/10.1080/09638280600926637

Ghazinour, M., Richter, J., & Eisemann, M. (2004). Quality of life among Iranian refugees resettled in Sweden. *Journal of Immigrant Health, 6*(2), 71-81.

Hammer, A. L., & Marting, M. S. (1988). *Manual for the coping resources inventory*: Consulting Psychologists Press.

Haugan, G., Rinnan, E., Espnes, G. A., Drageset, J., Rannestad, T., & André, B. (2019). Development and psychometric properties of the Joy-of-Life Scale in cognitively intact nursing home patients. *Scandinavian Journal of Caring Sciences, 33*(4), 801-814. doi: https://doi.org/10.1111/scs.12676

Haugan, G. (2021). Nurse-patient interaction: A vital salutogenic resource in nursing home care. In G. Haugan & M. Eriksson (Eds.), *Health Promotion in Health Care – Vital Theories and Research* (pp. 117-136). Springer. https://doi.org/10.1007/978-3-030-63135-2_10

Iani, L., Quinto, R. M., Porcelli, P., & Abeni, D. (2019). The effect of sense of coherence and positivity on spiritual well-being and distress in individuals with skin diseases. *Psychotherapy and Psychosomatics, 88 (Supplement 1)*, 58. doi: http://dx.doi.org/10.1159/000502467

Kinjerski, V. (2013). The spirit at work scale: Developing and validating a measure of individual spirituality at work. In J. Neal (Ed.), *Handbook of faith and spirituality in the workplace: Emerging research and practice* (pp. 383-402). New York: Springer.

Lee, L. E. (1998). *Internal coping resources as predictive of individual outcome in outpatient drug rehabilitation treatment* [Doctoral dissertation, City University of New York]. ProQuest Dissertations and Theses Global.

Lundman, B., Arestedt, K., Norberg, Å., Norberg, C., Santamaki Fischer, R., & Lövheim, H. (2015). Psychometric properties of the Swedish version of the self-transcendence scale among very old people. *Journal of Nursing Measurement, 23*(1), 96-111. doi: https://doi.org/10.1891/1061-3749.23.1.96

Mak, W. W. S., Ng, I. S. W., Wong, C. C. Y., & Law, R. W. (2017). Resilience style questionnaire: Development and validation among college students and cardiac patients in Hong Kong. *Assessment, 26*(4), 706-725. doi: https://doi.org/10.1177/1073191116683798

Maree, D. J. F., Maree, M., & Collins, C. (2008). Constructing a South African hope measure. *Journal of Psychology in Africa, 18*(1), 167-177. doi: https://doi.org/10.1080/14330237.2008.10820183

Meghani, S. H., Peterson, C., Kaiser, D. H., Rhodes, J., Rao, H., Chittams, J., & Chatterjee, A. (2018). A pilot study of a mindfulness-based art therapy intervention in outpatients with cancer. *American Journal of Hospice & Palliative Medicine, 35*(9), 1195-1200. doi: https://doi.org/10.1177/1049909118760304

Moe, A., Hellzen, O., Ekker, K., & Enmarker, I. (2013). Inner strength in relation to perceived physical and mental health among the oldest old people with chronic illness. *Aging & Mental Health, 17*(2), 189-196. doi: https://doi.org/10.1080/13607863.2012.717257

Neff, K. D. (2003). The development and validation of a scale to measure self-compassion. *Self and Identity, 2*(3), 223-250. doi: https://doi.org/10.1080/15298860309027

Nygren, B., Aléx, L., Jonsén, E., Gustafson, Y., Norberg, A., & Lundman, B. (2005). Resilience, sense of coherence, purpose in life and self-transcendence in relation to perceived physical and mental health among the oldest old. *Aging & Mental Health, 9*(4), 354-362. doi: https://doi.org/10.1080/1360500114415

Oluyinka, O. (2011). Psychological predictors of attitude towards seeking professional psychological help in a Nigerian university student population. *South African Journal of Psychology, 41*(3), 310-327.

Ozaki, M. (2005). Development of an assessment tool on spirituality explained by three domains, Will, joy and sense: From a holistic educational approach. *Journal of International Society of Life Information Science, 23*(2), 364-369.

Paloutzian, R. F., & Ellison, C. (1982). Spiritual well-being scale. In P. C. Hill & R. W. Hood Jr (Eds.), *Measures of religiosity* (pp. 382-385): Religious Education Press.

Peterman, A. H., Fitchett, G., Brady, M. J., Hernandez, L., & Cella, D. (2002). Measuring spiritual well-being in people with cancer: The functional assessment of chronic illness therapy—Spiritual Well-being Scale (FACIT-Sp). *Annals of Behavioral Medicine, 24*(1), 49-58. doi: https://doi.org/10.1207/S15324796ABM2401_06

Piedmont, R. L. (2010). *Assessment of spirituality and religious sentiments technical manual*. Timonium: Author.

Piedmont, R. L., Magyar-Russell, G., DiLella, N., & Matter, S. (2014). Sense of coherence: Big five correlates, spirituality, and incremental validity. *Current Issues in Personality Psychology, 2*(1), 1-9. doi: https://doi.org/10.5114/cipp.2014.43096

Reed, P. G. (2009). Demystifying self-transcendence for mental health nursing practice and research. *Archives of Psychiatric Nursing, 23*(5), 397-400. doi: https://doi.org/10.1016/j.apnu.2009.06.006

Rokeach, M. (1956). Political and religious dogmatism: An alternative to the authoritarian personality. *Psychological Monographs: General and Applied, 70*(18), 1-43. doi: https://doi.org/10.1037/h0093727

Shin, N. Y., & Lim, Y.-J. (2019). Contribution of self-compassion to positive mental health among Korean university students. *International Journal of Psychology, 54*(6), 800-806. doi: https://doi.org/10.1002/ijop.12527

Sundararajan-Reddy, S. (2005). *The relationship of spirituality to resilience in adolescents.* [Doctoral dissertation, Rutgers, The State University of New Jersey]. ProQuest Dissertations and Theses Global.

Unterrainer, H.-F., Huber, H.-P., Ladenhauf, K. H., Wallner-Liebmann, S. J., & Liebmann, P. M. (2010). MI-RSB 48. Die Entwicklung eines multidimensionalen Inventars zum religiös-spirituellen Befinden [MI-RSB 48. The development of a multidimensional inventory of religious-spiritual well-being]. *Diagnostica, 56*(2), 82-93. doi: https://doi.org/10.1026/0012-1924/a000001

Unterrainer, H.-F., Lewis, A., Collicutt, J., & Fink, A. (2013). Religious/spiritual well-being, coping styles, and personality dimensions in people with substance use disorders. *International Journal for the Psychology of Religion, 23*(3), 204-213. doi: https://doi.org/10.1080/10508619.2012.714999

van der Westhuizen, S., de Beer, M., & Bekwa, N. (2011). Psychological strengths as predictors of postgraduate students' academic achievement. *Journal of Psychology in Africa, 21*(3), 473-478.

von Humboldt, S., Leal, I., Pimenta, F., & Maroco, J. (2014). Assessing adjustment to aging: A validation study for the adjustment to aging scale (AtAS). *Social Indicators Research, 119*(1), 455-472.

Watkins, C. C., Kanu, I. K., Hamilton, J. B., Kozachik, S. L., & Gaston-Johansson, F. (2017). Differences in coping among African American women with breast cancer and triple-negative breast cancer. *Oncology Nursing Forum, 44*(6), 689-702. doi: https://doi.org/10.1188/17.ONF.689-702

Wenzl, M., Fuchshuber, J., Podolin-Danner, N., Silani, G., & Unterrainer, H.-F. (2021). The Swedish version of the Multidimensional Inventory for Religious/Spiritual Well-Being: First results from Swedish students. *Frontiers in Psychology*, 12, 783761. https://doi.org/10.3389/fpsyg.2021.783761

Wilkins, T. A., Piedmont, R. L., & Magyar-Russell, G. M. (2012). Spirituality or religiousness: Which serves as the better predictor of elements of mental health? *Research in the Social Scientific Study of Religion, 23*, 53-73.

Wissing, J. A. B., Wissing, M. P., du Toit, M. M., & Temane, Q. M. (2008). Psychometric properties of various scales measuring psychological well-being in a South African context: The FORT 1 Project. *Journal of Psychology in Africa, 18*(4), 511-520. doi: https://doi.org/10.1080/14330237.2008.10820230

Ying, Y.-W. (2008). Variation in personal competence and mental health between entering and graduating MSW students: The contribution of mindfulness. *Journal of Religion & Spirituality in Social Work: Social Thought, 27*(4), 405-422. doi: https://doi.org/10.1080/15426430802347347

Ying, Y.-W. (2009). Contribution of self-compassion to competence and mental health in social work students. *Journal of Social Work Education, 45*(2), 309-323. doi: https://doi.org/10.5175/JSWE.2009.200700072

Zerach, G., & Levin, Y. (2018). Posttraumatic stress symptoms, burn-out, and compassion satisfaction among body handlers: The mediating role of sense of coherence and spirituality at workplace. *Journal of Interpersonal Violence, 33*(12), 1931-1957. doi: https://doi.org/10.1177/0886260515621065
