# Supplementary material for: Sense of coherence and religion/spirituality: A systematic review and meta-analysis based on a methodical classification of instruments measuring religion/spirituality
Source: PLoS One. 2023 Aug 3;18(8):e0289203. doi: 10.1371/journal.pone.0289203 (PMC10399782; doi:10.1371/journal.pone.0289203)
Supplement: S10 Table — List of all scales that can be considered as measures of R/S according to our (heuristic) definition. The table contains information about the item-wise classification of each scale, the dominant R/S aspects, Cronbach’s alpha, and a sample item. (PDF) [file pone.0289203.s014.pdf]

S15 Table. Characteristics and Classification of the Included R/S Measurement Tools Used in the Studies Qualified for Meta-Analysis.

Scale	Scale author(s)	Study	α	Scale description	Items	Item class.	Scale class.	R/S codes	Example item
Australian Sheep Goat Scale	Thalbourne & Delin (1993)	Goulding (2004; 2005)	NA	Total scale with three sub-scales: 1. Beliefs and experiences of extrasensory perception (11 items), 2. Psychokinesis (5 items), 3. Belief in life after death (2 items)	18	S, S, S, S, S, S, S, S, S, S, S, S, S, S, S, S, RS, SR	**S**<br>T: 100%<br>R: 11%<br>S: 100%<br>N: 6%	Afterlife (2), awareness (2), belief (2), communication (1), control (2), **experience** (9), **extrasensory perception** (16), future (4), identity (2), meaning (3), mysticism (2), negative aspects (1), **paranormal beliefs** (16), power (1), retrospection (1), self-image (2), understanding (3)	"I believe I have had personal experience of ESP."
Awareness	Hall & Edwards (2002)	Gavulic (2018)	NA	Sub-scale of the Spiritual Assessment Inventory (47 items)	19	R, R, R, R, R, R, R, R, R, R, R, R, R, R, R, R, R, R, R	**R**<br>T: 100%<br>R: 100%<br>S: 0%<br>N: 0%	**Awareness** (15), centrality (3), **communication** (9), connectedness (3), coping (3), emotion (2), **experience** (5), extraordinary perception (3), frequency (1), **God concept** (14), **God relationship** (15), grandiosity (4), guidance (2), individual practice (4), **influence** (5), inspiration (1), mysticism (4), way of life (4)	"I experience an awareness of God speaking to me personally."
Belief in punishing God[a]	Self-developed scale	Diržytė et al. (2003)	NA	Sub-scale of the Religious Beliefs Questionnaire (40 items)	NA	R, R, (…)	**N-R**	God concept (1), God relationship (1), **negative aspects** (2) (…)	"God punishes for bad behavior."
Beliefs and Practices	Holland et al. (1998)	López et al. (2015)	NA	Sub-scale of the Systems of Belief Inventory (15 items)	10	R, RS/SR, R, R, RS/SR, RS/SR, RS/SR, R, R, RS	**RS**<br>T: 100%<br>R: 100%<br>S: 50%<br>N: 0%	**belief** (6), centrality (1), **coping** (6), development (1), efficacy (2), emotion (2), experience (2), future (1), **God concept** (4), God relationship (2), individual practice (3),	"I believe God protects me from harm."

Scale	Scale author(s)	Study	α	Scale description	Items	Item class.	Scale class.	R/S codes	Example item
								influence (2), meaning (1), optimism (2), trust (2), way of life (1)	
Beliefs and Values	Fetzer Institute & National Institute on Aging Working Group (1999)	Johnson-Migalski (2006)	NA	Items from the Brief Multidimensional Measurement of Religiousness/Spirituality (33 items)	2	R, (S)	**RS**<br>T: 100%<br>R: 50%<br>S: 50%<br>N: 0%	**Belief** (1), **emotion** (1), **God concept** (1), **God relationship** (1), **idealism** (1), **prosociality** (1)	"I believe in a God who watches over me."
Centrality of Religiosity Scale	Huber & Huber (2012)	Zarzycka & Rydz (2014b)	.94	Total scale with five sub-scales: 1. Intellect (3 items), 2. Ideology (3 items), 3. Public practice (3 items), 4. Private practice (3 items), 5. Experience (3 items)	15	RS, RS, R, RS, RS, RS, SR, R, RS, RS, RS, SR, R, SR, RS	**RS**<br>T: 100%<br>R: 100%<br>S: 80%<br>N: 0%	Awareness (2), belief (3), **centrality** (5), communication (1), collective practice (2), intellect (3), emotion (3), experience (3), **frequency** (8), God concept (3), God relationship (3), afterlife (1), **individual practice** (4), influence (1), organized religion (3), power (1), social support (1), way of life (1)	"How often do you experience situations in which you have the feeling that God or something divine is present?"
Centrality of Religiosity Scale (shortened)	Huber (2008)	Zehnder Grob (2015)	.91	Total scale with five sub-scales: 1. Intellect (2 items), 2. Ideology (2 items), 3. Public Practice (2 items), 4. Private Practice (2 items), 5. Experience (2 items)	10	R, RS, RS, SR, R, R, RS, SR, RS, RS	**RS**<br>T: 100%<br>R: 100%<br>S: 70%<br>N: 0%	Afterlife (1), belief (2), **centrality** (3), churchiness (2), collective practice (2), communication (1), experience (1), emotion (2), **frequency** (4), God concept (2), God relationship (1), individual practice (2), intellect (2), mysticism (1), organized religion (2)	"How often do you experience situations in which you have the feeling that God or something divine wants to communicate or to reveal something to you?"
Centrality of Religious Meaning System Scale[a]	Self-developed scale	Dezutter et al. (2010)	.97	Total scale (one open question followed by 12 closed questions)	1+12	RS, RS, RS, RS, RS, RS, (…)	**RS**	**Centrality** (5), identity (1), meaning (1), **way of life** (4), (…)	"My religion / spirituality / faith influences my

Scale	Scale author(s)	Study	α	Scale description	Items	Item class.	Scale class.	R/S codes	Example item
									behavior in my daily life."
Conscious State Expansion	King & DeCicco (2009)	Ebrahimi Meymand et al (2021)	NA	Sub-scale of the Spiritual Intelligence Self-Report Inventory (24 items)	5	S, S, S, S, S	**S**<br>T: 100%<br>R: 0%<br>S: 100%<br>N: 0%	Autonomy (1), **awareness** (5), **control** (2), development (1), frequency (1), **individual practice** (2), understanding (1)	"I can control when I enter higher states of consciousness or awareness."
Connectedness	Unterrainer (2007)	Unterrainer & Ladenhauf (2008); Unterrainer et al. (2010); Unterrainer et al. (2013); Berger et al. (2016)<br>Wenzl et al. (2021)	.80<br><br><br><br><br><br><br><br><br>.81	Sub-scale of the Multidimensional Instrument for the Measurement of Religious-Spiritual Well-Being (48 items)	8	S, SR, S, S, SR, (S), S, (S)	**S**<br>T: 100%<br>R: 25%<br>S: 100%<br>N: 0%	**Afterlife** (3), **belief** (3), emotion (1), **experience** (5), extraordinary perception (2), future (1), grandiosity (1), **mysticism** (3), paranormal beliefs (1), power (1), self-transcendence (1), universality (1)	"I have experienced the feeling of being absorbed into something greater."
Critical Existential Thinking	King & DeCicco (2009)	Ebrahimi Meymand et al (2021)	NA	Sub-scale of the Spiritual Intelligence Self-Report Inventory (24 items)	7	(S), S, S, S, S, S, SR	**S**<br>T: 100%<br>R: 14%<br>S: 100%<br>N: 0%	Afterlife (2), autonomy (1), connectedness (1), development (1), effort (1), frequency (2), higher being (1), **individual practice** (1), **intellect** (7), meaning (1), power (1), quest (1), universality (1)	"I have developed my own theories about such things as life, death, reality, and existence."
Daily Spiritual Experience Scale	Underwood (2011)	Zafar et al. (2019); Zerach (2013)	.80<br>.95	Total scale with no sub-scales	16	R, S, R, RS/SR, RS/SR, (X), R, R, R, R, S, (RS), (S), (X), RS, RS	**RS**<br>T: 81%<br>R: 69%<br>S: 50%<br>N: 0%	Awareness (2), collective practice (1), communication (1), connectedness (3), coping (2), effort (1), **emotion** (12), **experience** (15), **frequency** (15), God concept (2), **God relationship** (8), guidance (1), mysticism (1), nature (1),	"During worship, or at other times when connecting with God, I feel joy which lifts me out of my daily concerns."

Scale	Scale author(s)	Study	α	Scale description	Items	Item class.	Scale class.	R/S codes	Example item
								prosociality (1), self-transcendence (2), social support (1), trust (1), universality (2), way of life (2)	
Daily Spiritual Experience Scale (shortened)	Fetzer Institute & National Institute on Aging Working Group (1999); Underwood & Teresi (2002)	Johnson-Migalski (2006); Büssing et al. (2016)	.91	Items from the Brief Multidimensional Measurement of Religiousness/Spirituality (33 items); short form of the Daily Spiritual Experience Scale (16 items) respectively	6	R, RS/SR, (X), R, S, RS	**RS**<br>T: 83%<br>R: 67%<br>S: 50%<br>N: 0%	**Awareness** (2), connectedness (1), coping (1), effort (1), **emotion** (3), **experience** (6), **frequency** (6), **God concept** (2), **God relationship** (3), mysticism (1), nature (1), social support (1)	"I feel God's love for me, directly or through others."
Degree of Spirituality	Self-developed item	DeBruyn (2001)	NA	One item of 8 items measuring different aspects of religion/spirituality	1	S	**S**<br>T: 100%<br>S: 100%<br>R: 0%<br>N: 0%	**Centrality** (1), **identity** (1), **meaning** (1)	"How spiritual a person do you consider yourself to be? By spiritual, I mean, having a sense of meaning, purpose, and direction?"
Disappointment	Hall & Edwards (2002)	Gavulic (2018)	NA	Sub-scale of the Spiritual Assessment Inventory (47 items)	7	R, R, R, R, R, R, R	**N-R**<br>T: 100%<br>R: 100%<br>S: 0%<br>N: 100%	Communication (1), God concept (4), God relationship (7), individual practice (1), **negative aspects** (7)	"There are times when I feel disappointed with God."
Duke University Religion Index	Koenig et al. (1997)	Curtis (2000)	NA	Total scale with 3 sub-scales: 1. Organizational Religious Activity (1 item), 2. Non-Organizational Religious Activity (1 item), 3. Intrinsic Religiosity (3 items)	5	R, RS, RS, R, R	**R**<br>T: 100%<br>R: 100%<br>S: 40%<br>N: 0%	Awareness (1), belief (1), **centrality** (2), churchiness (1), collective practice (1), effort (1), experience (1), **frequency** (2), God relationship (1), individual practice (1), intellect (1), organized religion (1), **way of life** (2)	"My religious beliefs are what really lie behind my whole approach to life."

Scale	Scale author(s)	Study	α	Scale description	Items	Item class.	Scale class.	R/S codes	Example item
Everyday Relevance of Religiosity	Self-developed items based on Dubach & Campiche (1993) and Huber (2008)	Zehnder Grob (2015)	.75	Sub-scale of the questionnaire block Religiosity as Personality Factor	2	R, R	**R**<br>T: 100%<br>R: 100%<br>S: 0%<br>N: 0%	**Centrality** (2), **compliance** (2), **ethics** (2), **way of life** (2)	"How much do you live by religious precepts in your daily life?"[d]
Experience	Huber & Huber (2012)	Zarzycka & Rydz (2014b)		Sub-scale of the Centrality of Religiosity Scale (15 items)	3	RS, RS, RS	**RS**<br>T: 100%<br>R: 100%<br>S: 100%<br>N: 0%	Awareness (1), communication (1), **emotion** (3), **experience** (3), **frequency** (3), **God concept** (3), **God relationship** (3), influence (1)	"How often do you experience situations in which you have the feeling that God or something divine intervenes in your life?"
External Critique	Hutsebaut (1996)	Zarzycka and Rydz (2014a)	.87	Sub-scale of the Post-Critical Belief Scale (39 items)	11	RS, R, R, R, RS, RS, R, RS, R, RS, R	**N-RS**<br>T: 100%<br>R: 100%<br>S: 55%<br>N:	Belief (1), experience (1), God concept (2), intellect (4), **negative aspects** (9), power (1), understanding (1)	"In the end, faith is nothing more than a safety net for human fears."
Extrinsic Orientation	Allport & Ross (1967)	Littlejohn (1994); Hossack (1997)	.73	Sub-scale of the Religious Orientation Scale (20 items)	6	R, RS, R, RS, R, R	**R**<br>T: 100%<br>R: 100%<br>S: 33%<br>N: 0%	Affiliation (1), centrality (1), **churchiness** (2), compliance (1), **coping** (3), **individual practice** (3), **social interaction** (2), **organized religion** (2), **social support** (2)	"The primary purpose of prayer is to gain relief and protection."
Extrinsic Religious Orientation (shortened)	Feagin (1964); Batson & Schoenrade (1991)	Strümpfer (1997)	.59	Sub-scale of the Intrinsic/Extrinsic Scale (21 items); only the 6 highest loading items were used	6	RS, R, RS, R, R, RS	**RS**<br>T: 100%<br>R: 100%<br>S: 50%<br>N: 0%	Affiliation (1), **churchiness** (3), compliance (1), **coping** (2), **efficacy** (2), **individual practice** (3), intellect (1), **social interaction** (3), socialization (1), **social support** (3)	"The Church is most important as a place to formulate good social relationships."
Faith	Peterman et al. (2002)	Meghani et al. (2018)	NA	Sub-scale of the Functional Assessment of Chronic Illness	4	SR, SR, SR, (X)	**SR**<br>T: 75%<br>R: 75%	**Belief** (3), **coping** (3), development (1), **emotion** (2),	"I find strength in my faith or spiritual beliefs."

Scale	Scale author(s)	Study	α	Scale description	Items	Item class.	Scale class.	R/S codes	Example item
				Therapy—Spiritual Well-Being			S: 75%<br>N: 0%	future (1), optimism (1), trust (1)	
General Religiosity	Unterrainer (2007)	Unterrainer & Ladenhauf (2008); Unterrainer et al. (2010); Unterrainer et al. (2013); Berger et al. 2016<br>Wenzl et al. (2021)	.94<br><br>.97	Sub-scale of the Multidimensional Instrument for the Measurement of Religious-Spiritual Well-Being (48 items)	8	RS, RS, RS, RS, RS, RS, R, SR	**RS**<br>T: 100%<br>R: 100%<br>S: 88%<br>N: 0%	Awareness (1), belief (1), collective practice (1), communication (1), connectedness (1), coping (2), **emotion** (5), experience (1), future (2), **God concept** (4), **God relationship** (5), nature (1), optimism (2), organized religion (1), social support (1)	"At certain moments in my life, I feel very close to God."
General Spiritual Scale[b]	McIlwain (1990)	Uren & Wastell (2002)	.72	Sub-scale of the Spiritual Orientation Scale	6	NA	**NA**	NA	NA
Global Religiosity[b]	Grabner (1998); Trinker (2002)	Unterrainer & Ladenhauf (2008); Unterrainer et al. (2010); Unterrainer et al. (2013)	.97	Short form of the Global Religiosity Scale (71 items)	20	NA	**NA**	NA	NA
God concepts	Huber (2008)	Zehnder Grob (2015)	.81	Sub-scale of the questionnaire block Religiosity as Personality Factor	3	SR, R, RS	**RS**<br>T: 100%<br>R: 100%<br>S: 67%<br>N: 0%	Communication (1), **God concept** (3), God relationship (1), higher being (1), mysticism (1), power (1)	"God or the divine is like… a higher power."[e]
Grandiosity	Hall & Edwards (2002)	Gavulic (2018)	NA	Sub-scale of the Spiritual Assessment Inventory (47 items)	7	R, R, R, R, R, R, R	**R**<br>T: 100%<br>R: 100%<br>S: 0%<br>N: 0%	Centrality (1), communication (1), coping (1), **efficacy** (3), extraordinary perception (1), **God concept** (3), **God relationship** (7), **grandiosity**	"I seem to have a unique ability to influence God through my prayers."

Scale	Scale author(s)	Study	α	Scale description	Items	Item class.	Scale class.	R/S codes	Example item
								(7), identity (1), **individual practice** (3), **self-image** (5)	
Ideology	Huber & Huber (2012)	Zarzycka & Rydz (2014b)		Sub-scale of the Centrality of Religiosity Scale (15 items)	3	R, RS/SR, S	**RS/SR**<br>T: 100%<br>R: 67%<br>S: 67%<br>N: 0%	Afterlife (1), **belief** (3), God concept (1), power (1)	"To what extent do you believe that God or something divine exists?"
Importance of Religion	Self-developed item	Tagay, et al. (2006)	NA	Single item	1	R	**R**<br>T: 100%<br>R: 100%<br>S: 0%<br>N: 0%	**Centrality** (1), **way of life** (1)	"How important is religion for your life?"
Impression Management	Hall & Edwards (2002)	Gavulic (2018)	NA	Sub-scale of the Spiritual Assessment Inventory (47 items)	5	R, R, R, RS, RS	**R**<br>T: 100%<br>R: 100%<br>S: 40%<br>N: 0%	**Centrality** (2), **churchiness** (2), **emotion** (2), **frequency** (4), God relationship (1), **grandiosity** (3), guidance (1), **individual practice** (2), **organized religion** (2), **prosociality** (2), **self-image** (3), social interaction (1), way of life (1)	"I am always as kind at home as I am at church."
Instability	Hall & Edwards (2002)	Gavulic (2018)	NA	Sub-scale of the Spiritual Assessment Inventory (47 items)	9	R, R, R, R, R, R, R, R, R	**N-R**<br>T: 100%<br>R: 100%<br>S: 0%<br>N: 89 %	Connectedness (3), ethics (3), God concept (8), God relationship (8), **negative aspects** (9)	"When I sin, I am afraid of what God will do to me."
Intellect	Huber & Huber (2012)	Zarzycka & Rydz (2014b)		Sub-scale of the Centrality of Religiosity Scale (15 items)	3	R, R, R	**R**<br>T: 100%<br>R: 100%<br>S: 0%<br>N: 0%	Centrality (1), **frequency** (2), individual practice (1), **intellect** (3)	"How often do you think about religious issues?"
Intensity of Religious Attitude Scale	Prężyna (1981)	Skowroński & Talik (2021)	.96	Total scale with two sub-scales: 1. Positive attitude (17 items), 2.	30	NA	NA	NA	NA

Scale	Scale author(s)	Study	α	Scale description	Items	Item class.	Scale class.	R/S codes	Example item
				Negative attitude (13 items)					
Intrinsic Orientation	Allport & Ross (1967)	Littlejohn (1994)	NA	Sub-scale of the Religious Orientation Scale (20 items)	14	RS, RS, R, R, R, R, R, R, R, R, R, R, R, R	**R**<br>T: 100%<br>R: 100%<br>S: 14%<br>N: 0%	Affiliation (1), autonomy (2), awareness (1), belief (2), **centrality** (6), **churchiness** (4), **collective practice** (4), effort (1), emotion (2), ethics (1), frequency (1), God relationship (1), higher being (1), identity (1), individual practice (3), **intellect** (4), meaning (2), organized religion (3), **way of life** (4)	"My religious beliefs are what really lie behind my whole approach to life."
Intrinsic Orientation (shortened)	Allport & Ross (1967)	Hossack (1997)	.93	Sub-scale of the Religious Orientation Scale (20 items); items 1, 3 and 11 were eliminated, order of items was changed, wording of some items was modified	11	RS, R, R, R, R, R, RS, R, R, R, R	**R**<br>T: 100%<br>R: 100%<br>S: 18%<br>N: 0%	Affiliation (1), autonomy (1), awareness (1), belief (1), **centrality** (4), **churchiness** (4), **collective practice** (4), effort (1), emotion (1), frequency (1), God relationship (1), higher being (1), individual practice (3), **intellect** (4), meaning (2), organized religion (3), way of life (2)	"A primary reason for my interest in religion is that my church is a congenial social activity."
Intrinsic Religiosity	King & Hunt (1975)	Chamberlain & Zika (1988)	.97	Two sub-scales were used as a single measure for Intrinsic Religiosity: 1. Orientation: Growth and Striving (6 items), 2. Salience: Cognition (8 items). The two sub-scales have 3 overlapping items that were excluded.	11	R, RS, RS, R, R, R, R, R, R, R, R	**R**<br>T: 100%<br>R: 100%<br>S: 18%<br>N: 0%	Belief (2), **centrality** (4), churchiness (1), collective practice (1), connectedness (1), development (1), effort (2), emotion (1), experience (1), forgiveness (1), **frequency** (5), God concept (1), **God relationship** (4), identity (1), individual practice (3), intellect (3), meaning (1), self-image	"I try hard to grow in understanding of what it means to live as a child of God."

Scale	Scale author(s)	Study	α	Scale description	Items	Item class.	Scale class.	R/S codes	Example item
								(2), trust (1), understanding (2), **way of life** (5)	
Intrinsic Religiosity	Koenig, et al. (1997)	Curtis (2000)	NA	Sub-scale of the Duke University Religion Index (5 items)	3	RS, R, R	**R**<br>T: 100%<br>R: 100%<br>S: 33%<br>N: 0%	Awareness (1), belief (1), **centrality (2)**, effort (1), experience (1), God relationship (1), **way of life (3)**	"I try to carry my religion over into all other dealings in life."
Intrinsic Religiosity	Gorsuch & McPherson (1989)	Abel et al. (2014)	.63	Sub-scale of the Intrinsic/Extrinsic Religiosity Scales (14 items)	8	R, RS, SR, R, R, R, R, R	**R**<br>T: 100%<br>R: 100%<br>S: 25%<br>N: 0%	Awareness (1), centrality (4), effort (1), **emotion** (3), ethics (1), frequency (1), God relationship (1), **individual practice** (4), intellect (2), **way of life** (4)	"I enjoy reading about my religion."
Intrinsic Religious Motivation (shortened)	Hoge (1972)	Strümpfer (1997)	.78	Total scale with no sub-scales; item 11 was deleted	9	RS, R, RS, R, R, R, RS, R, R	**R**<br>T: 100%<br>R: 100%<br>S: 33%<br>N: 0%	Awareness (1), belief (2), **centrality** (6), compliance (1), **effort** (3), ethics (1), experience (1), **God relationship** (3), guidance (1), identity (1), influence (1), quest (1), **way of life** (6)	"Nothing is as important to me as serving God as best as I know."
Intrinsic/Extrinsic Religiosity Scales	Gorsuch & McPherson (1989)	Bradbury et al. (2009)	.75	Total scale with two sub-scales: 1. Intrinsic Religiosity (8 items), 2. Extrinsic Religiosity (6 items)	14	R, R, RS, SR, R, RS, R, R, RS, R, R, R, R, R	**R**<br>T: 100%<br>R: 100%<br>S: 29%<br>N: 0%	Awareness (1), belief (2), **centrality** (4), churchiness (3), coping (3), collective practice (3), compliance (1), effort (1), emotion (3), ethics (1), experience (1), frequency (1), God relationship (1), identity (1), **individual practice** (4), intellect (2), organized religion (3), social support (3), **way of life** (4)	"My whole approach to life is based on my religion."
Negative Religious Coping	Pargament et al. (2000)	Khanjari et al. (2012);	.76	Sub-scale of the Brief Religious Coping Scale (14 items)	7	R, R, R, R, R, R, R	**N-R**<br>T: 100%<br>R: 100%	Churchiness (1), God concept (5), God relationship (4), **negative aspects** (7),	"Questioned God's love for me."

Scale	Scale author(s)	Study	α	Scale description	Items	Item class.	Scale class.	R/S codes	Example item
		Rohani et al. (2010)<br>Skalski-Bednarz et al. (2022)	.79<br><br>.81				S: 100%<br>N: 100%	organized religion (1), self-image (3), social support (1)	
Non-Organizational Religious Activity	Koenig, et al. (1997)	Schonder (2016)	.84	Sub-scale of the Duke University Religion Index (5 items)	1	RS	**RS**<br>T: 100%<br>R: 100%<br>S: 100%<br>N: 0%	**Individual practice** (1), **intellect** (1), **frequency** (1)	"How often do you spend time in private religious activities, such as prayer, meditation or Bible study?"
Organizational Religious Activity	Koenig, et al. (1997)	Schonder (2016)	.84	Sub-scale of the Duke University Religion Index (5 items)	1	R	**R**<br>T: 100%<br>R: 100%<br>S: 0%<br>N: 0%	**Churchiness** (1), **collective practice** (1), **frequency** (1), **organized religion** (1)	"How often do you attend church or other religious meetings?"
Original Spiritual Fitness Scale	Peterson et al. (2011)	Hammer et al. (2013)	.71 - .76	Sub-scale of the Global Assessment Tool (105 items)	5	S, (S), (S), S, S	**S**<br>T: 100%<br>R: 0%<br>S: 100%<br>N: 0%	**Belief** (2), connectedness (1), identity (1), **meaning** (3), self-transcendence (1), universality (1), way of life (1), workplace (1)	"I believe there is a purpose for my life."
Orthodoxy	Hutsebaut (1996)	Zarzycka and Rydz (2014a)	.71	Sub-scale of the Post-Critical Belief Scale (39 items)	8	R, R, R, R, R, R, RS, R	**R**<br>T: 100%<br>R: 100%<br>S: 13%<br>N: 0%	**Belief** (3), churchiness (1), **compliance** (4), God concept (1), God relationship (1), meaning (2), organized religion (2), **truth** (5), way of life (2)	"Ultimately, there is only one correct answer to each religious question."
Personal Faith	O'Brien (1999); Rego (2008)	Encarnação et al. (2018)	NA	Sub-scale of the Spiritual Assessment Scale (21 items)	7	R, R, R, S, R, R, RS	**R**<br>T: 100%<br>R: 86%<br>S: 29%<br>N: 0%	**Belief** (4), coping (1), **emotion** (3), future (1), **God concept** (4), **God relationship** (5), higher being (1), **optimism** (2), self-image (1), **trust** (2), **universality** (2)	"I trust that God will take care of the future."

Scale	Scale author(s)	Study	α	Scale description	Items	Item class.	Scale class.	R/S codes	Example item
Personal Meaning Production	King & DeCicco (2009)	Ebrahimi Meymand et al (2021)	NA	Sub-scale of the Spiritual Intelligence Self-Report Inventory (24 items)	5	S, S, S, S, S	**S**<br>T: 100%<br>R: 0%<br>S: 100%<br>N: 0%	Coping (1), experience (1), **meaning** (5), **way of life** (2)	"I am able to find meaning and purpose in my everyday experiences."
Positive Religious Coping	Pargament et al. (2000)	Khanjari et al. (2012);<br>Rohani, et al. (2010);<br>Skalski-Bednarz et al. (2022)	.86<br>.89<br>.92	Sub-scale of the Brief Religious Coping Scale (14 items)	7	R, R, R, R, R, R, R	**R**<br>T: 100%<br>R: 100%<br>S: 100%<br>N: 14%	**Coping** (7), communication (1), connectedness (1), **effort** (5), emotion (1), future (1), **God concept** (2), **God relationship** (5), **individual practice** (3), negative aspects (1), quest (1)	"Sought help from God in letting go of my anger."
Positive Spiritual Experiences (Mystic positive)	Self-developed scale	Kohls et al. (2008)	NA	Sub-scale of the Exceptional Experiences Questionnaire (57 items)	7	SR, (S), SR, S, S, S, S	**S**<br>T: 100%<br>R: 29%<br>S: 100%<br>N: 0%	**Awareness** (3), coping (1), connectedness (1), **experience** (3), **extraordinary perception** (3), God concept (1), higher being (1), influence (1), inspiration (1), meaning (1), mysticism (1), paranormal beliefs (1), power (1), universality (1), workplace (1)	"I am illumined by divine light and divine strength."
Prayer Fulfillment (shortened)	Piedmont (2010)	Piedmont et al. (2014);<br>Wilkins et al. (2012)	.91	Sub-scale of the Spiritual Transcendence Scale and from the Assessment of Spirituality and Religious Sentiments-Short Form (13 items)	3	SR, SR, SR	**SR**<br>T: 100%<br>S: 100%<br>R: 100%<br>N: 0%	**Coping** (2), **efficacy** (3), **emotion** (3), **individual practice** (3), mysticism (1), self-transcendence (1)	"My prayers and/or meditations provide me with a sense of emotional support."
Private Practice	Huber & Huber (2012)	Zarzycka and Rydz (2014b)		Sub-scale of the Centrality of Religiosity Scale (15 items)	3	RS, RS, RS	**RS**<br>T: 100%<br>R: 100%<br>S: 100%<br>N: 0%	Centrality (1), **frequency** (2), **individual practice** (3), inspiration (1), way of life (1)	"How often do you pray?"

Scale	Scale author(s)	Study	α	Scale description	Items	Item class.	Scale class.	R/S codes	Example item
Psychopathology	Self-developed scale	Kohls et al. (2008)	NA	Sub-scale of the Exceptional Experiences Questionnaire (57 items)	7	(S), SR, (SR), (SR), (S), (X), (S)	**N-S**<br>T: 86%<br>S: 86%<br>R: 43%<br>N: 100%	Communication (3), extraordinary perception (3), influence (4), **negative aspects** (7), paranormal beliefs (6), power (2)	"I am cursed."
Public Practice	Huber & Huber (2012)	Zarzycka and Rydz (2014b)		Sub-scale of the Centrality of Religiosity Scale (15 items)	3	R, R, R	**R**<br>T: 100%<br>R: 100%<br>S: 0%<br>N: 0%	Affiliation (1), **centrality** (2), **churchiness** (2), **collective practice** (2), frequency (1), **organized religion** (3)	"How important is to take part in religious services?"
Quest Scale	Batson & Schoenrade (1991)	Bossick (2008)	.80	Total scale with three sub-scales: 1. Readiness to face existential complexities without reducing their complexity (4 items), 2. Self-criticism and perceptions of religious doubts as positive (4 items), 3. Openness to change (4 items)	12	RS, RS, R, RS, R, R, R, RS, RS, RS, RS, RS	**RS**<br>T: 100%<br>R: 100%<br>S: 67%<br>N: 8%	Awareness (1), belief (1), centrality (3), **development** (4), experience (2), God relationship (1), idealism (1), identity (2), **intellect** (4), meaning (2), negative aspects (1), **quest** (9), retrospection (1)	"As I grow and change, I expect my religion also to grow and change."
Realistic Acceptance	Hall & Edwards (2002)	Gavulic (2018)	NA	Sub-scale of the Spiritual Assessment Inventory (47 items)	7	R, R, R, R, R, R, R	**R**<br>T: 100%<br>R: 100%<br>S: 0%<br>N: 0%	Awareness (1), communication (1), connectedness (1), coping (1), **effort** (3), **emotion** (3), **God concept** (3), **God relationship** (7), individual practice (1), **quest** (3), **trust** (2)	"When this happens, my trust in God is not completely broken."
Relativism	Hutsebaut (1996)	Zarzycka and Rydz (2014a)	.72	Sub-scale of the Post-Critical Belief Scale (39 items)	11	R, R, R, R, (X), R, (RS), (RS), (RS), (S), R	**R**<br>T: 91%<br>R: 82%<br>S: 36%<br>N: 0%	Churchiness (1), experience (1), God concept (2), God relationship (1), **intellect** (4), social interaction (1), organized religion (1), **plurality** (4), quest (3), meaning (2)	"I am well aware that my ideology is only one possibility among so many others."

Scale	Scale author(s)	Study	α	Scale description	Items	Item class.	Scale class.	R/S codes	Example item
Reliance on God's help	Büssing et al. (2015)	Kerksieck et al. (2016)	.78	Total scale with no sub-scales	5	SR, R, RS, RS, R	**RS**<br>T: 100%<br>R: 100%<br>S: 60%<br>N: 0%	Belief (1), compliance (1), **coping** (4), effort (1), **God concept** (2), **God relationship** (2), individual practice (1), influence (1), **optimism** (2), power (1), **trust** (2), way of life (1)	"I have a strong belief that God will help me."
Religion	Self-developed scale	Gabrielsen et al. (2012); Gabrielsen et al. (2013)	.74 - .75	Sub-scale of the Adolescent Life Goal Profile Scale (21+21 items)	2+2	R, SR, R, SR	**RS**<br>T: 100%<br>R: 100%<br>S: 50%<br>N: 0%	**Afterlife** (2), **belief** (4), **centrality** (2), **God concept** (2)	"To believe in a God."
Religion as a Means	Batson & Ventis (1982)	Hossack (1997)	.80	Sub-scale of the Religious Life Inventory (35 items)	6	R, R, R, R, R, R	**R**<br>T: 100%<br>R: 100%<br>S: 100%<br>N: 0%	**Centrality** (3), **churchiness** (3), coping (1), **development** (5), emotion (1), family resource (1), identity (1), pastoral care (1), **socialization** (5), social support (1)	"The church has been very important for my religious development."
Religion as a Quest	Batson & Ventis (1982)	Hossack (1997)	.82	Sub-scale of the Religious Life Inventory (35 items); item 17 was dropped	11	R, RS, R, RS, R, R, R, R, R, RS, SR	**R**<br>T: 100%<br>R: 100%<br>S: 36%<br>N: 0%	**Autonomy** (4), awareness (1), belief (1), centrality (3), compliance (1), coping (1), **development** (4), experience (1), God relationship (1), identity (1), intellect (2), meaning (2), **quest** (9), retrospection (1), truth (1)	"I am constantly questioning my religious beliefs."
Religion as an End	Batson & Ventis (1982)	Hossack (1997)	.91	Sub-scale of the Religious Life Inventory (35 items)	8	R, R, R, R, R, R, RS, R	**R**<br>T: 100%<br>R: 100%<br>S: 13%<br>N: 0%	Belief (1), **centrality** (5), development (1), emotion (1), God relationship (2), guidance (1), **identity** (4), individual practice (1), quest (1), trust (1), truth (1), way of life (2)	"I have found it essential to have faith."
Religion/Spiritua lity	Heppner et al. (2006)	Ahmad (2012)	.92	Sub-scale of the Collectivist Coping	4	RS, R, R, R	**R**<br>T: 100%	Belief (1), collective practice (1), **coping** (4), **efficacy** (4),	"Found guidance from my religion."

Scale	Scale author(s)	Study	α	Scale description	Items	Item class.	Scale class.	R/S codes	Example item
				Styles Inventory (30 items)			R: 100%<br>S: 25%<br>N: 0%	guidance (1), individual practice (1), intellect (1), meaning (1)	
Religiosity	Self-developed scale	Kibour (2002)	NA	Two items: 1. Self-Rated Religiosity (1 item), 2. Ceremony Attendance (1 item)	2	R, R	**R**<br>T: 100%<br>R: 100%<br>S: 0%<br>N: 0%	**Collective practice** (1), **frequency** (1), **identity** (1), **organized religion** (1)	"On a scale of 1-7 how often do you attend religious services?"
Religiosity	Self-developed scale	Dyer (2022)	NA	Three items: 1. Affiliation (1 item), 2. Salience (1 item), 3. Religious activity (1 item)	3	R, RS/SR, R	**R**<br>T: 100%<br>R: 100%<br>S: 33%<br>N: 0%	**Affiliation (2)**, belief (1), centrality (1), collective practice (1), identity (1), organized religion (1)	"What is your religious or spiritual preference?"
Religiosity and Search for Meaning	Muthny (1989)	Unterrainer & Ladenhauf (2008)	NA	Sub-scale of the Freiburg Questionnaire of Illness Coping (35 items)	5	(SR), R, (S), (SR), (X)	**SR**<br>T: 80%<br>R: 60%<br>S: 60%<br>N: 0%	**Coping** (4), **effort** (2), emotion (1), **meaning** (2), prosociality (1), quest (1)	"Accepting the disease as fate."
Religiosity[a]	Kecskes & Wolf (1993)	Renner et al. (2004)	.97	Sub-scale of the Austrian Value Questionnaire (54 items); the sub-scale is based on two sub-scales of the Christian Religiosity Scales: 1. Religious Experience (6 items), 2. Religious Belief (15 items)	12	R, R, R, R, R, R, R, R, R, R, R, R, R, R, R, R, R, RS, R, R, R, R, SR, RS, R; based on the 21 items of Kecskes & Wolf (1993)	**R**<br>T: 100%<br>R: 100%<br>S: 14%<br>N: 0%	Afterlife (2), **belief** (15), coping (3), emotion (3), experience (1), future (1), **God concept** (9), **God relationship** (7), meaning (2), way of life (1)	"God rewards good deeds."
Religiosity[a]	Katz (2000)	Israelashvili et al. (2011)	.81	Total scale with no sub-scales (8 items for	8-10	R, R, (…)	**R**<br>NA	Centrality (1), compliance (1), identity (1), way of life (1), (…)	"To which extent do you wish to preserve the

Scale	Scale author(s)	Study	α	Scale description	Items	Item class.	Scale class.	R/S codes	Example item
				Druze, 10 items for Jews)					traditional Druze/Jewish life style?"
Religious / Spiritual Coping	Self-developed scale	Aderhold et al. (2019)	.91	Sub-scale of the Patient Competence in Coping with Cancer Questionnaire (18 items)	4	RS/SR, RS/SR, RS, RS	**RS**<br>T: 100%<br>R: 100%<br>S: 100%<br>N: 0%	**Coping** (4), effort (1), emotion (1), God concept (1), **God relationship** (2), **power** (2), **trust** (2)	"I seek comfort in faith."[k]
Religious Commitment	Fetzer Institute & National Institute on Aging Working Group (1999)	Johnson-Migalski (2006)	NA	Items from the Brief Multidimensional Measurement of Religiousness/Spirituality (33 items)	3	R, R, RS	**R**<br>T: 100%<br>R: 100%<br>S: 33%<br>N: 0%	Affiliation (1), belief (1), **centrality** (2), **churchiness** (2), collective practice (1), effort (1), frequency (1), individual practice (1), **organized religion** (2), prosociality (1), **way of life** (2)	"During the last year about how much was the average monthly contribution of your household to your congregation or to religious causes?"
Religious Coping	Self-developed scale with items based on Klaghofer & Oser (1987); Pargament et al. (2000)	Zehnder Grob (2015)	.85 - .86	Sub-scale of the questionnaire block Religion as a Means to Well-Being and Problem Solving	3	R, RS, RS	**RS**<br>T: 100%<br>R: 100%<br>S: 67%<br>N: 33%[c]	**Coping** (3), **God concept** (2), **God relationship** (2), **higher being** (2), negative aspects (1)[c], **power** (2), quest (1), trust (1)	"Religion helps me not to despair in difficult situations."[f]
Religious Hope	Self-developed scale	Magnano (2003)	.86	Sub-scale of the Hope Scale (68 items)	7	R, R, R, R, R, R, R	**R**<br>T: 100%<br>R: 100%<br>S: 0%<br>N: 14%	Awareness (1), centrality (1), control (1), **coping** (2), **emotion** (3), experience (1), **future** (2), **God concept** (4), **God relationship** (6), meaning (1), nature (1), negative aspects (1), optimism (1), self-image (1), **trust** (2), **way of life** (2)	"Only in God do we have hope."

Scale	Scale author(s)	Study	α	Scale description	Items	Item class.	Scale class.	R/S codes	Example item
Religious Identity	Self-developed items based on Dubach & Campiche (1993); Saroglou & Galand (2004)	Zehnder Grob (2015)	.81	Sub-scale of the questionnaire block Religiosity as Personality Factor	2	R, R	**R**<br>T: 100%<br>R: 100%<br>S: 0%<br>N: 0%	**Affiliation** (2), **churchiness** (1), **identity** (1), **organized religion** (2)	"How connected do you feel to your church/religious community?"[g]
Religious Importance Scale	Self-developed scale	Racklin (1999)	.89	Total scale with no sub-scales	8	R, R, R, R, R, R, R, R	**R**<br>T: 100%<br>R: 100%<br>S: 0%<br>N: 0%	Centrality (1), **coping** (4), emotion (1), identity (1), **meaning** (3), **organized religion** (7)	"I look to organized religion for answers to life's difficult questions."
Religious Involvement	Piedmont (2010)	Piedmont et al. (2014); Wilkins et al. (2012)	.84 - .86	Sub-scale of the Spiritual Transcendence Scale and from the Assessment of Spirituality and Religious Sentiments-Short Form (13 items)	4	R, R, RS, R	**R**<br>T: 100%<br>S: 25%<br>R: 100%<br>N: 0%	Collective practice (1), **frequency** (4), **individual practice** (3), **intellect** (2), organized religion (1)	"How often do you read the Bible/Torah/Koran/ Geeta?"
Religious Life Inventory (shortened)	Batson & Ventis (1982)	Hossack (1997)	NA	Total scale with 3 sub-scales and 7 filler items: 1. Religion as a Means (6 items), 2. Religion as an End (10 items), 3. Religion as a Quest (12 items); item 17 (Quest) and item 22 (filler) were dropped due to problematic factor loadings	33	R, R, R, R, RS, R, R, RS, R, R, R, R, R, R, R, R, R, R, R, R, SR, R, R, R, R, RS, R, RS, R, R, SR, R, R	**R**<br>T: 100%<br>R: 100%<br>S: 18%<br>N: 0%	Affiliation (1), autonomy (4), belief (2), **centrality** (12), churchiness (3), compliance (3), coping (1), **development** (12), emotion (1), experience (1), family resource (1), frequency (1), future (1), God relationship (3), guidance (1), idealism (2), identity (5), individual practice (2), influence (1), intellect (6), meaning (2), organized religion (4), pastoral care (1), **quest** (11), retrospection (5), self (1), social interaction (2),	"As I grow and change, I expect my religion also to grow and change."

Scale	Scale author(s)	Study	α	Scale description	Items	Item class.	Scale class.	R/S codes	Example item
								social support (3), socialization (5), trust (1), truth (2), way of life (2)	
Religious Maturity Scale	Dudley & Cruise (1990)	Hossack (1997)	.71	Total scale with no sub-scales	11	RS, RS, R, S, R, (RS), R, RS, RS, R, R	**RS**<br>T: 100%<br>R: 91%<br>S: 55%<br>N: 0%	**Autonomy** (4), belief (3), centrality (1), **development** (4), emotion (1), God relationship (1), **intellect** (5), meaning (3), prosociality (1), **quest** (6), social interaction (1), social support (1), **truth** (4), understanding (3)	"As best as I can determine, my religion is true, but I recognize that I could be mistaken on some points."
Religious Meaning Questionnaire	Self-developed scale	Krok (2016)	.92	Total scale with two sub-scales: 1. Orientation (10 items), 2. Meaningfulness (10 items)	20	R, RS, R, RS, RS, RS, R, R, R, R, R, R, R, R, RS, R, R, R, R, R	**R**<br>T: 100%<br>R: 100%<br>S: 25%<br>N: 0%	Autonomy (2), belief (2), **centrality** (10), compliance (1), collective practice (1), coping (2), effort (3), emotion (2), ethics (2), frequency (1), God concept (1), God relationship (2), idealism (1), individual practice (4), **meaning** (5), quest (1), retrospection (1), social support (1), **intellect** (5), understanding (1), **way of life** (6)	"I rarely think about religion in the midst of my daily affairs and tasks."[h]
Religious Pluralism (summarized)	Self-developed items based on Saroglou & Hanique (2006); Ziebertz et al. (2008)	Zehnder Grob (2015)	NA	The sub-scales 1. Acceptance of Religious Pluralism (2 items) and 2. Religious Endogamy (2 items) are summarized in this review to the scale "Religious Pluralism".	4	R, R, R, R	**R**<br>T: 100%<br>R: 100%<br>S: 0%<br>N: 0%	Autonomy (1), **compliance** (2), **ethics** (2), **plurality** (4)	"I would never marry someone with a different religion."[i]
Religious Practice	O'Brien (1999); Rego (2008)	Encarnação et al. (2018)	NA	Sub-scale of the Spiritual Assessment Scale (21 items)	7	R, R, R, R, RS, R, RS	**R**<br>T: 100%<br>R: 100%	Belief (1), centrality (1), churchiness (1), **collective practice** (2), communication	"I am supported by relationships with friends or family

Scale	Scale author(s)	Study	α	Scale description	Items	Item class.	Scale class.	R/S codes	Example item
							S: 29%<br>N: 0%	(1), **coping** (2), efficacy (1), **emotion** (2), family resource (1), **God relationship** (2), **individual practice** (3), intellect (1), **organized religion** (2), pastoral care (1), prosociality (1), **social support** (4), way of life (1)	members who share my religious beliefs."
Religious Practice Dimension	De Jong et al. (1976)	Edwards and Besseling (2001)	NA	Sub-scale of the Religiosity Scale (29 items)	5	R, R, R, R, R	**R**<br>T: 100%<br>R: 100%<br>S: 0%<br>N: 0%	**Affiliation** (2), **churchiness** (4), **collective practice** (2), ethics (1), **frequency** (3), individual practice (1), intellect (1), **organized religion** (4), prosociality (1)	"How often do you attend Sabbath worship services?"
Religious Socialization (summarized)	Self-developed scale with items based on Dillen & Pollefeyt (2005); Huber (2008); Klaghofer & Oser (1987)	Zehnder Grob (2015)	.74 - .93	The sub-scales 1) Religious Socialization through Mother (3 items), 2) Religious Socialization through Father (3 items), 3. Religious Family Climate (5 items) and 4. Religious Autonomy (2 items) are combined in this review to the scale "Religious Socialization".	13	R, RS, R, R, RS, R, R, R, R, R, (X), RS, RS	**R**<br>T: 92%<br>R: 92%<br>S: 31%<br>N: 0%	Autonomy (2), centrality (1), churchiness (3), collective practice (3), compliance (1), coping (1), **family resource** (6), **frequency** (6), individual practice (2), intellect (2), organized religion (3), prosociality (1), social interaction (2), **socialization** (11), way of life (2)	"In my family, religion helps us to cope with difficult situations such as illness, misfortune or quarrels."[j]
Religious Well-Being	Paloutzian & Ellison (1982)	Lee (1998); Wissing et al. (2008); Vosloo et al. (2009)	NA	Sub-scale of the Spiritual Well-Being Scale (20 items)	10	R, R, R, R, R, R. R, R, R, R	**R**<br>T: 100%<br>R: 100%<br>S: 0%<br>N: 20%	**Belief** (3), connectedness (2), **coping** (4), emotion (2), **God concept** (3), **God relationship** (8), **meaning** (4), individual practice (1), mysticism (1), negative aspects (2), self-image (2), trust (1)	"My relationship with God contributes to my sense of well-being."

Scale	Scale author(s)	Study	α	Scale description	Items	Item class.	Scale class.	R/S codes	Example item
Revised Paranormal Belief Scale	Tobacyk (2004)	Bradbury et al. (2009)	.91	Total scale with six sub-scales: 1. Psi (4 items), 2. Witchcraft (4 items), 3. Superstition (3 items), 4. Spiritualism (4 items), 5. Extraordinary Life Forms (3 items), 6. Precognition (4 items)	22	S, (S), X, S, X, (S), (S), S, X, S, X, (S), (S), S, X, SR, X, S, (S), S, S, (S)	**N-S**<br>T: 73%<br>R: 5%<br>S: 73%<br>N: 41%	Afterlife (4), paranormal beliefs (16), belief (21), communication (1), control (3), extraordinary perception (6), future (3), God concept (1), **negative aspects** (9), power (1)	"Black magic really exists."
Santa Clara Strength of Religious Faith Questionnaire	Plante & Boccaccini (1997)	Schonder (2016)	.94 - .95	Total scale with no sub-scales	10	R, RS, RS/SR, SR, RS, RS/SR, R, RS/SR, RS/SR, RS/SR	**RS**<br>T: 100%<br>R: 100%<br>S: 80%<br>N: 0%	**Centrality** (5), churchiness (1), collective practice (1), coping (1), emotion (1), frequency (1), God relationship (1), identity (1), individual practice (1), inspiration (1), meaning (1), organized religion (1), self-image (1), social support (1), way of life (2)	"My religious faith is extremely important to me."
Second Naiveté	Hutsebaut (1996)	Zarzycka and Rydz (2014a)	.72	Sub-scale of the Post-Critical Belief Scale (39 items)	9	R, R, R, R, R, R, R, R, R	**R**<br>T: 100%<br>R: 100%<br>S: 0%<br>N: 18%	Belief (2), centrality (1), God concept (1), **God relationship** (3), guidance (2), identity (1), **intellect** (4), negative aspects (2), **quest** (3), retrospection (1), truth (2), understanding (1)	"The Bible is a guide, full of signs in the search for God, and not a historical account."
Seeking Spiritual Support	Olson (1983); Corcoran & Fischer (2013)	Mirsoleymani et al. (2021)	NA	Sub-scale of the Family Crisis Oriented Personal Evaluation Scales (30 items)	4	R, R, R, R	**R**<br>T: 100%<br>R: 100%<br>S: 0%<br>N: 0%	**Churchiness** (3), **collective practice** (2), **coping** (4), **family resource** (4), **frequency** (4), God relationship (1), **organized religion** (3), pastoral care (1), trust (1)	"When we face problems or crises in our family, we respond by: Participating in church activities."
Social Support	Holland et al. (1998)	López et al. (2015)	NA	Sub-scale of the Systems of Belief Inventory (15 items)	5	RS, RS/SR, RS/SR, RS/SR, RS/SR	**RS**<br>T: 100%<br>R: 100%<br>S: 100%	affiliation (1), collective practice (1), **coping** (3), **frequency** (2), organized	"When I feel lonely, I rely on people who share my spiritual or religious

Scale	Scale author(s)	Study	α	Scale description	Items	Item class.	Scale class.	R/S codes	Example item
							N: 0%	religion (1), **social interaction** (3), **social support** (3)	beliefs for support."
Spiritual Assessment Scale	O'Brien (1999); Rego (2008)	Encarnação et al. (2018)	NA	Total scale with three sub-scales: (1) Personal Faith (7 items), (2) Religious Practice (7 items), (3) Spiritual Contentment (7 items)	21	R, R, R, S, R, R, RS, R, R, R, R, RS, R, RS, S, R, R, R, R, R, R	**R**<br>T: 100%<br>R: 91%<br>S: 24%<br>N: 33%[c]	**Belief** (6), centrality (1), churchiness (1), collective practice (2), communication (1), connectedness (1), coping (4), efficacy (1), **emotion** (12), ethics (1), experience (1), family resource (1), future (1), **God concept** (9), **God relationship** (13), higher being (1),), individual practice (3), intellect (1), negative aspects (7)[c], **optimism** (5), organized religion (2), pastoral care (1), prosociality (1), self-image (1), social support (4), **trust** (5), universality (2), way of life (1)	"I am at peace with God."
Spiritual Change	Tedeschi & Calhoun (1996)	Brockhouse et al. (2011); Forstmeier et al. (2009); Nishi et al. (2010); Arya & Davidson (2015); Ragger et al. (2019) Farhadi et al. (2022)	.85	Sub-scale of the Posttraumatic Growth Inventory (21 items)	2	S, R	**RS/SR**<br>T: 100%<br>R: 50%<br>S: 50%<br>N: 0%	**Belief** (1), **development** (2), **understanding** (1)	"I have a stronger religious faith."
Spiritual Connection	Kinjerski (2013)	Zerach and Levin (2018)	NA	Sub-scale of the Spirituality at Work Scale (18 items)	3	S, S, S	**S**<br>T: 100%<br>S: 100%<br>R: 0%	Belief (1), centrality (1), connectedness (1), experience (1), inspiration (1), influence (1), power (1), **self-**	"I experience a connection with a greater source that

Scale	Scale author(s)	Study	α	Scale description	Items	Item class.	Scale class.	R/S codes	Example item
							N: 0%	**transcendence** (2), way of life (1), **workplace** (3)	has a positive effect on my work."
Spiritual Contentment	O'Brien (1999); Rego (2008)	Encarnação et al. (2018)	NA	Sub-scale of the Spiritual Assessment Scale (21 items)	7	S, R, R, R, R, R, R	**R**<br>T: 100%<br>R: 86%<br>S: 14%<br>N: 100%[c]	Belief (1), connectedness (1), coping (1), **emotion** (7), ethics (1), experience (1), **God concept** (5), **God relationship** (6), negative aspects (7)[c], **optimism** (3), **trust** (3)	"I feel that I have lost God's love."
Spiritual Dryness Scale	Büssing et al. (2013)	Kerksieck et al. (2016)	.86 - .87	Total scale with no sub-scales	6+3	R, R, S, S, RS, (X), (RS), (RS), SR	**N-RS**<br>T: 89%<br>R: 67%<br>S: 67%<br>N: 67%	Connectedness (1), coping (1), development (1), efficacy (1), effort (2), emotion (8), experience (2), frequency (6), God concept (1), God relationship (3), individual practice (1), **negative aspects** (6), quest (1)	"I have the feeling that God is distant from me, regardless of my efforts to draw close to him."
Spiritual Dryness Scale (shortened)	Büssing et al. (2013)	Büssing et al. (2013)	NA	Total scale with no sub-scales; without item 6	5+3	R, R, S, S, RS, (RS), (RS), SR	**N-RS**<br>T: 100%<br>R: 67%<br>S: 67%<br>N: 63%	Connectedness (1), coping (1), development (1), efficacy (1), effort (1), emotion (7), experience (2), frequency (6), God concept (1), God relationship (3), individual practice (1), **negative aspects** (5), quest (1)	"I have the feeling that God is distant from me, regardless of my efforts to draw close to him."
Spiritual Growth	Pakenham and Cox (2008)	Pakenham and Cox (2008)	.87	Sub-scale of the Benefit Finding in Multiple Sclerosis Caregiving scale (37 items)	4	S, S, RS, RS	**SR**<br>T: 100%<br>R: 50%<br>S: 100%<br>N: 0%	**Belief** (2), coping (1), **development** (4), emotion (1), identity (1), intellect (1), understanding (1)	"…become more spiritual."
Spiritual Intelligence Self-Report Inventory	King & DeCicco (2009)	Ebrahimi Meymand et al. (2021)	.93	Total scale with four sub-scales: 1. Critical existential thinking (7 items), 2. Personal meaning production (5	24	(S), S, S, S, S, S, S, S, S, S, S, S, S, S, S, S,	**S**<br>T: 100%<br>R: 4%<br>S: 100%<br>N: 0%	Afterlife (2), autonomy (2), **awareness** (9), connectedness (2), control (2), coping (1), development (2), effort (1), emotion (1), experience (2),	"I have spent time contemplating the purpose or reason for my existence."

Scale	Scale author(s)	Study	α	Scale description	Items	Item class.	Scale class.	R/S codes	Example item
				items), 3. Transcendental awareness (7 items), 4. Conscious state expansion (5 items).		S, S, S, S, SR, S, S		extraordinary perception (4), frequency (3), higher being (1), individual practice (5), **intellect** (7), **meaning** (7), power (1), quest (1), self (3), self-transcendence (2), understanding (1), universality (1), way of life (2)	
Spiritual Involvement and Beliefs Scale	Hatch et al. (1998)	Ferreira et al. (2020)	NA	Total scale with no sub-scales	26	X, (X), S, X, S, S, S, (S), RS, SR, S, S, S, S, S, S, SR, S, S, X, X, S, X, RS, S, SR	S<br>T: 77%<br>S: 77%<br>R: 19%<br>N: 4%	Autonomy (1), belief (4), collective practice (2), connectedness (3), control (1), coping (3), development (3), efficacy (1), emotion (5), experience (1), **frequency** (7), future (2), God relationship (1), idealism (2), identity (1), **individual practice** (8), influence (2), meaning (4), negative aspects (1), optimism (1), power (2), quest (1), retrospection (3), self (1), self-transcendence (3), social interaction (4), understanding (1), way of life (3)	"Participating in spiritual activities helps me forgive other people."
Spiritual Orientation Inventory	Elkins et al. (1988)	Littlejohn (1994); Racklin, (1999)	.97	Total scale with nine sub-scales: 1. Transcendent Dimension (13 items), 2. Meaning and Purpose in Life (10 items), 3. Mission in Life (9 items), 4. Sacredness of Life (15 items), 5.	85	S, (S), (S), SR, X, (S), X, (S), S, S, (S), SR, SR, S, S, S, S, S, S, S, S, X, S, (S), SR, X, S, S, S, S, S, X,	S<br>T: 80%<br>S: 80%<br>R: 9%<br>N: 0%	Autonomy (3), awareness (1), belief (13), centrality (3), communication (10), connectedness (5), coping (7), development (4), efficacy (2), effort (1), **emotion** (20), ethics (4), **experience** (18), extraordinary perception (1), family resource (1), frequency	"I have had experiences in which I felt very close to the transcendent, spiritual dimension."

Scale	Scale author(s)	Study	α	Scale description	Items	Item class.	Scale class.	R/S codes	Example item
				Material Values (6 items), 6. Altruism (7 items), 7. Idealism (10 items), 8. Awareness of the Tragic (5 items), 9. Fruits of Spirituality (10 items)		S, X, X, S, S, S, S, SR, S, S, X, X, S, S, S, S, (S), S, X, X, S, S, S, S, S, X, X, S, S, S, S, S, (S), S, S, S, X, SR, X, S, S, (S), S, X, S, S, SR, S, SR, S, S, X, S		(2), future (2), **idealism** (20), identity (3), intellect (1), **meaning** (21), mysticism (6), nature (1), optimism (6), power (1), prosociality (3), quest (5), retrospection (3), sacredness (7), self-transcendence (4), social interaction (2), trust (1), truth (1), universality (8), way of life (3)	
Spiritual Perspective	Pakenham (2007)	Pakenham (2007)	.87	Sub-scale of the Sense Making Scale (38 items)	8	R, SR, S, S, SR, S, S, (SR)	**SR**<br>T: 100%<br>R: 50%<br>S: 87.5%<br>N: 0%	**Coping** (7), development (2), **future** (3), God concept (1), **meaning** (8)	"My getting MS was destiny or fate."
Spiritual Perspective Scale	Reed (1986)	Conway-Phillips and Janusek (2014); Gibson (2000); Khanjari et al. (2012); Rohani et al. (2010)	.87<br>.91	Total scale with two sub-scales: 1. Behaviors (4 items), 2. Beliefs (6 items)	10	S, S, S, SR, S, S, S, RS, S, S	**S**<br>T: 100%<br>R: 20%<br>S: 100%<br>N: 0%	Belief (1), **centrality** (6), collective practice (1), compliance (1), connectedness (1), coping (1), effort (1), emotion (2), family resource (1), forgiveness (1), **frequency** (5), God relationship (1), guidance (1), **individual practice** (3), influence (1), intellect (1), meaning (1), power (1), quest (2), retrospection (1), social interaction (2), **way of life** (5)	"My spirituality is a significant part of my life."
Spiritual Sensitivity	Self-developed scale	Ozaki (2005)	.73	Sub-scale of the Spiritual Behavior,	11	(S), SR, S, S, R, (X),	**S**<br>T: 82%	Afterlife (1), awareness (1), belief (2), effort (1), **emotion**	"I feel that there is eternal time that

Scale	Scale author(s)	Study	α	Scale description	Items	Item class.	Scale class.	R/S codes	Example item
				Attitude, and Sensitivity Test (29 items)		(S), SR, S, X, (S)	S: 73%<br>R: 27%<br>N: 18%	(5), extraordinary perception (1), frequency (1), God concept (1), idealism (1), individual practice (2), intellect (3), meaning (1), mysticism (1), nature (2), negative aspects (2), paranormal beliefs (1), power (1), self (1), self-image (2), self-transcendence (3)	goes on before I was born, and after I have died."
Spiritual Suffering	Self-developed scale	Encarnação et al. (2018)	.55	Sub-scale of the Suffering Assessment Questionnaire (12 items)	3	S, S, R	**S**<br>T: 100%<br>R: 33%<br>S: 67%<br>N: 67%[c]	**Connectedness** (3), **coping** (2), **emotion** (2), God relationship (1), negative aspects (2)[c]	"After being diagnosed with the disease, I feel more connected to spirituality."
Spiritual Transcendence Scale	Piedmont (1999)	Delgado (2007)	.72	Total scale with three sub-scales: 1. Universality, 2. Prayer Fulfillment, 3. Connectedness	23	SR, S, S, S, S, S, (S), S, X, (X), S, SR, (S), X, S, S, S, SR, SR, S, RS, X, X	**S**<br>T: 78%<br>R: 22%<br>S: 78%<br>N: 0%	Afterlife (3), belief (4), centrality (2), connectedness (2), coping (2), development (3), efficacy (4), **emotion** (9), ethics (1), experience (1), God relationship (1), higher being (1), idealism (3), **individual practice** (6), influence (2), meaning (2), mysticism (2), prosociality (2), self-transcendence (4), social support (2), understanding (1), **universality** (6), way of life (1)	"I feel that on a higher level all of us share a common bond."
Spirituality	Self-developed scale	Kim et al. (2020)	.97	Sub-scale of the Personal Growth Scale in Partners of Persons With Multiple Sclerosis (32 items)	2	RS, RS	**RS**<br>T: 100%<br>R: 100%<br>S: 100%<br>N: 0%	**Coping** (2), development (1), **efficacy** (2), **frequency** (2), individual practice (1)	"Prayer has been valuable to me."

Scale	Scale author(s)	Study	α	Scale description	Items	Item class.	Scale class.	R/S codes	Example item
Spirituality	WHO Group (1995)	Ghazinour et al. (2004)	NA	Sub-scale of the WHO Quality of Life scale (100 items)	4	(SR/RS), (S), (RS/SR), (RS/SR)	**SR**<br>T: 100%<br>R: 75%<br>S: 100%<br>N: 0%	**belief** (3), **coping** (2), emotion (1), **meaning** (2), understanding (1)	"Do your personal beliefs give meaning to your life?"
Spirituality Index	Self-developed scale	Post-White et al. (1996)	.78	Total scale with no sub-scales	6	R, SR/RS, RS, X, SR, S	**SR/RS**<br>T: 83%<br>R: 75%<br>S: 75%<br>N: 17%	Awareness (1), belief (1), **coping** (4), **frequency** (6), **emotion** (2), meaning (1), **individual practice** (2), intellect (1), negative aspects (1)	"I use prayer or meditation to give me strength."
Subjective Religiosity	Self-developed item	Tagay et al. (2006)	NA	Single item	1	R	**R**<br>T: 100%<br>R: 100%<br>S: 0%<br>N: 0%	**Centrality** (1), **identity** (1)	"To what extent are you religious?"
Traditional Religious Beliefs	Tobacyk (2004)	Bradbury et al. (2009)	.78	Independent sub-scale of the Revised Paranormal Belief Scale (26 items)	4	RS, R, R, R	**R**<br>T: 100%<br>R: 100%<br>S: 25%<br>N: 25%	**Afterlife** (2), **belief** (4), God concept (1), negative aspects (1)	"There is a heaven and a hell."
Transcendental Awareness	King & DeCicco (2009)	Ebrahimi Meymand et al. (2021)	NA	Sub-scale of the Spiritual Intelligence Self-Report Inventory (24 items)	7	S, S, S, S, S, S, S	**S**<br>T: 100%<br>R: 0%<br>S: 100%<br>N: 0%	**Awareness** (3), connectedness (1), emotion (1), experience (1), **extraordinary perception** (3), **self** (3), self-transcendence (2)	"I define myself by my deeper, non-physical self."
Transpersonal Trust	Belschner (2003)	Kohls et al. (2008)	NA	Total scale with no sub-scales	11	SR, S, SR, SR, SR, S, R, S, (S), SR, S	**SR**<br>T: 100%<br>R: 50%<br>S: 91%<br>N: 0%	Afterlife (1), belief (2), **connectedness** (5), coping (2), efficacy (1), effort (1), emotion (3), experience (1), God relationship (3), guidance (2), higher being (3), identity (1), individual practice (1), influence (2), mysticism (3),	"Sometimes in my life I have the impression that I am led by a higher insight."

Scale	Scale author(s)	Study	α	Scale description	Items	Item class.	Scale class.	R/S codes	Example item
								**self-transcendence** (4), **trust** (4), universality (3)	
Turning to Religion	Carver et al. (1989)	Basson and Rothmann (2002); Herbst et al. (2007); Fernández-Martínez et al. (2019); Rothmann and Van Rensburg (2002); Van der Colff & Rothmann (2009)	.83 - .93	Sub-scale of the COPE Scales (53 items)	4	R, R, R, RS	**R**<br>T: 100%<br>R: 100%<br>S: 25%<br>N: 0%	**Coping** (3), **effort** (2), **frequency** (4), **God relationship** (2), individual practice (1), **trust** (2)	"I put my trust in God."
Turning to Religion (shortened)	Carver (1997)	Nahlén & Saboonchi 2010; Verouli et al. (2016); Reguera-García et al. (2020); Diaconescu et al. (2021); Zamanian et al. (2021); Nolvi et al. (2022); Torinomi et al. (2022)	.88	Sub-scale of the Brief COPE Inventory (28 items)	2	RS/SR, RS/SR	**RS/SR**<br>T: 100%<br>R: 100%<br>S: 100%<br>N: 0%	**Coping** (2), **frequency** (2), **individual practice** (1)	"I've been trying to find comfort in my religion or spiritual beliefs."
Universality (shortened)	Piedmont (2010)	Piedmont et al. (2014);	.56 -	Sub-scale of the Spiritual Transcendence	3	S, (S), S	**S**<br>T: 100%	Belief (1), **Emotion** (2), **universality** (3)	"I feel that on a higher level all of us

Scale	Scale author(s)	Study	α	Scale description	Items	Item class.	Scale class.	R/S codes	Example item
		Wilkins et al. (2012)	.58	Scale and from the Assessment of Spirituality and Religious Sentiments-Short Form (13 items)			R: 0%<br>S: 100%<br>N: 0%		share a common bond.”

Note. N= negative aspects of religiosity/spirituality; N-R = negative religiosity; N-RS = negative religiosity with strong spiritual components; N-SR = negative spirituality with strong religious components; N-S = negative spirituality; R = primarily religiosity; (R) = not clearly religiosity, but can be interpreted religiously; RS = religiosity with strong spiritual components; (RS) = not clearly religiosity/spirituality, but can be interpreted as religiosity with strong spiritual components; RS/SR = spiritual and religious aspects in equal proportions; SR = spirituality with strong religious components; (SR) = not clearly spirituality/religiosity, but can be interpreted as spirituality with strong religious components; S = primarily spirituality; (S) = not clearly spirituality, but can be interpreted spiritually; T = The proportion of items in the total scale that measure religious or spiritual aspects (%); X = neither religious nor spiritual aspects; (X) = neither religious nor spiritual aspects, but the item could possibly be interpreted religiously/spiritually.

R/S aspects that are in **bold** are classified as dominant dimensions of R/S in the respective measurement instrument. Dominance was calculated as follows: If the scale included 1-4 items, 50% of the items had to address the same R/S aspect for that R/S aspect to be classified as dominant; if the scale included 5-14 items, 33% of the items had to address the same R/S aspect for that R/S aspect to be classified as dominant; if the scale included 14-25 items, 25% of the items had to address the same R/S aspect for this R/S aspect to be considered dominant; if the scale included more than 26 items, 20% of the items had to address the same R/S aspect for this R/S aspect to be considered dominant.

[a] The classification of this scale could only be done in a rudimentary way, because the exact wording of all items was not known. [b] The measuring instrument or the items used in the study could not be determined with certainty, so that a classification of the items and the scale was not possible. [c] Although one-third of the items on this scale address negative aspects of R/S, the measurement tool is not classified as a negative measure of R/S. This is because this scale is designed as a positive measure of R/S and accordingly all negatively worded items are reverse scored. [d] Translated by first author. Wording of the original German item: “Wie stark lebst du in deinem Alltag nach religiösen Geboten?“. [e]

Translated by first author. Wording of the original German item: “Gott oder das Göttliche ist wie… eine höhere Macht“. [f] Translated by first author. Wording of the original German item: “Die Religion hilft mir, in schwierigen Situationen nicht zu verzweifeln“. [g] Translated by first author. Wording of the original German item: “Wie sehr fühlst du dich deiner Kirche/Religionsgemeinschaft verbunden?“. [h] Translated by FJ. Wording of the original Polish item: “Rzadko pośród codziennych spraw i zadań myślę o religii.” [i] Translated by first author. Wording of the original German item: “Ich würde nie jemanden mit einer anderen Religion heiraten“. [j] Translated by first author. Wording of the original German item: “In meiner Familie hilft uns Religion, schwierige Situationen wie Krankheit, Unglücksfälle oder Streit zu meistern“. [k] Translated by first author. Wording of the original German item: “Ich suche Trost im Glauben.“

References

Abel, L., Walker, C., Samios, C., & Morozow, L. (2014). Vicarious posttraumatic growth: Predictors of growth and relationships with adjustment. *Traumatology: An International Journal, 20*(1), 9-18. doi: 10.1037/h0099375

Aderhold, C., Morawa, E., Paslakis, G., & Erim, Y. (2019). Entwicklung und Validierung eines Fragebogens zur Patientenkompetenz im Umgang mit einer Krebserkrankung (PUK). *Zeitschrift für Psychosomatische Medizin und Psychotherapie, 65*(3), 239-256. doi: https://doi.org/10.13109/zptm.2019.65.3.239

Ahmad, S. (2012). *Coping with cultural conflict: Strategies used by South Asian American women* [Doctoral dissertation, University of La Verne]. ProQuest Dissertations and Theses Global.

Basson, M., & Rothmann, S. (2002). Sense of coherence, coping and burnout of pharmacists. *South African Journal of Economic and Management Sciences, 5*(1), 35-62.

Belschner, W. (2003). Ergebnisse empirischer Forschung zur Transpersonalen Psychotherapie [Results of empirical research on transpersonal psychotherapy]. In J. Galuska (Ed.), *Den Horizont erweitern: Die transpersonale Dimension in der Psychotherapie* (pp. 93-135). Berlin: Ulrich Leutner Verlag.

Berger, D., Fink, A., Perez Gomez, M. M., Lewis, A., & Unterrainer, H.-F. (2016). The validation of a Spanish version of the multidimensional inventory of religious/spiritual well-being in Mexican college students. *The Spanish Journal of Psychology, 19*(e3), 1-11. doi: https://doi.org/10.1017/sjp.2016.9

Bossick, B. E. (2008). *An empirical examination of the relationship between posttraumatic growth and the personality traits of hardiness, sense of coherence, locus of control, self-efficacy, resilience, and optimism* [Doctoral dissertation, University of Akron]. ProQuest Dissertations and Theses Global.

Bradbury, D. A., Stirling, J., Cavill, J., & Parker, A. (2009). Psychosis-like experiences in the general population: An exploratory factor analysis. *Personality and Individual Differences, 46*(7), 729-734. doi: https://doi.org/10.1016/j.paid.2009.01.035

Brockhouse, R., Msetfi, R. M., Cohen, K., & Joseph, S. (2011). Vicarious exposure to trauma and growth in therapists: The moderating effects of sense of coherence, organizational support, and empathy. *Journal of Traumatic Stress, 24*(6), 735-742. doi: https://doi.org/10.1002/jts.20704

Büssing, A., Baumann, K., Jacobs, C., & Frick, E. (2016). Spiritual dryness in Catholic priests: Internal resources as possible buffers. *Psychology of Religion and Spirituality, 9*(1), 46-55. doi: https://psycnet.apa.org/doi/10.1037/rel0000063

Büssing, A., Günther, A., Baumann, K., Frick, E., & Jacobs, C. (2013). Spiritual dryness as a measure of a specific spiritual crisis in catholic priests: Associations with symptoms of burnout and distress. *Evidence-Based Complementary and Alternative Medicine, 2013*, Article ID 246797. doi: http://dx.doi.org/10.1155/2013/246797

Carver, C. S. (1997). You want to measure coping but your protocol's too long: Consider the brief COPE. *International Journal of Behavioral Medicine, 4*(1), 92-100. doi: https://dio.org/10.1207/s15327558ijbm0401_6

Carver, C. S., Scheier, M. F., & Weintraub, J. K. (1989). Assessing coping strategies: A theoretically based approach. *Journal of Personality and Social Psychology, 56*(2), 267-283. doi: https://doi.org/10.1037/0022-3514.56.2.267

Chamberlain, K., & Zika, S. (1988). Religiosity, life meaning and wellbeing: Some relationships in a sample of women. *Journal for the Scientific Study of Religion, 27*(3), 411-420. doi: https://doi.org/10.2307/1387379

Conway-Phillips, R., & Janusek, L. (2014). Influence of sense of coherence, spirituality, social support and health perception on breast cancer screening motivation and behaviors in African American women. *The ABNF Journal, 25*(3), 72-79.

Corcoran, K., & Fischer, J. (2013). *Measures for clinical practice and research: A Sourcebook. Volume 1: Couples, families, and children*. New York: Oxford University Press.

Curtis, R. C. (2000). *Coping with prostrate cancer: The effects of family strength, sense of coherence, and spiritual resources* [Doctoral dissertation, University of North Carolina at Greensboro]. ProQuest Dissertations and Theses Global.

De Jong, G. F., Faulkner, J. E., & Warland, R. H. (1976). Dimensions of religiosity reconsidered: Evidence from a cross-cultural study. *Social Forces, 54*(4), 866-889. doi: https://doi.org/10.2307/2576180

DeBruyn, J. C. (2001). *Binge drinking and salutogenesis: Sense of coherence, stress, religiousness and spirituality* [Doctoral dissertation, Western Michigan University Kalamazoo]. ProQuest Dissertations and Theses Global.

Delgado, C. (2007). Sense of coherence, spirituality, stress and quality of life in chronic illness. *Journal of Nursing Scholarship, 39*(3), 229-234. doi: https://doi.org/10.1111/j.1547-5069.2007.00173.x

Dezutter, J., Robertson, L. A., Luyckx, K., & Hutsebaut, D. (2010). Life satisfaction in chronic pain patients: The stress-buffering role of the centrality of religion. *Journal for the Scientific Study of Religion, 49*(3), 507-516. doi: https://doi.org/10.1111/j.1468-5906.2010.01525.x

Diaconescu, L. V., Gheorghe, I. R., Chesches, T., & Popa-Velea, O. (2021). Psychological variables associated with HPV vaccination intent in Romanian academic settings. *International Journal of Environmental Research and Public Health, 18*(17), 15, Article 8938. https://doi.org/10.3390/ijerph18178938

Dillen, A., & Pollefeyt, D. (2005). Family education and adolescents' approaches to religion. *Journal of Empirical Theology, 18*(2), 205-234. doi: http://dx.doi.org/10.1163/157092505774649216

Diržytė, A., Patapas, A., & Limantaitė, E. (2003). Religious beliefs, sense of coherence and well being of Lithuanian students. *Socialinis Darbas, 1*(3), 15-22.

Dudley, R. L., & Cruise, R. J. (1990). Measuring religious maturity: A proposed scale. *Review of Religious Research, 32*(2), 97-109. doi: https://doi.org/10.2307/3511758

Dyer, W. J. (2022). Refining research on the intersection between sexual orientation, suicide, and religiosity. *Psychology of Religion and Spirituality, 14*(2), 179-188. doi: https://doi.org/10.1037/rel0000451

Ebrahimi Meymand, H. A., Askarizadeh, G., Bagheri, M., & Arabnejad, M. (2021). The role of spiritual intelligence, sense of coherence, and cognitive flexibility as internal resources in predicting perceived stress in patients with multiple sclerosis. *Quarterly Horizon of Medical Sciences, 27*(1), 114-129. https://doi.org/10.32598/hms.27.1.2986.2

Edwards, D., & Besseling, E. (2001). Relationship between depression, anxiety, sense of coherence, social support and religious involvement in a small rural community affected by industrial relations conflict. *South African Journal of Psychology, 31*(4), 62-71. doi: https://doi.org/10.1177/008124630103100408

Elkins, D. N., Hedstrom, L. J., Hughes, L. L., Leaf, J. A., & Saunders, C. (1988). Toward a humanistic-phenomenological spirituality: Definition, description, and measurement. *Journal of Humanistic Psychology, 28*(4), 5-18. doi: https://doi.org/10.1177/0022167888284002

Encarnação, P., Oliveira, C. C., & Martins, T. (2018). Psychometric properties of the suffering assessment questionnaire in adults with chronic diseases or life-threatening illness. *Scandinavian Journal of Caring Sciences, 32*(4), 1279-1287. doi: https://doi.org/10.1111/scs.12569

Farhadi, A., Bahreini, M., Moradi, A., Mirzaei, K., & Nemati, R. (2022). The predictive role of coping styles and sense of coherence in the post-traumatic growth of mothers with disabled children: a cross-sectional study. *BMC Psychiatry, 22*(1), 708. https://doi.org/10.1186/s12888-022-04357-5

Fernandez-Martinez, E., Lopez-Alonso, A. I., Marques-Sanchez, P., Martinez-Fernandez, M. C., Sanchez-Valdeon, L., & Liebana-Presa, C. (2019). Emotional intelligence, sense of coherence, engagement and coping: A cross-sectional study of university students' health. *Sustainability, 11*(24), 6953. doi: 10.3390/su11246953

Ferreira, D. C., Gonçalves, T. R., Celeste, R. K., Olinto, M. T. A., & Pattussi, M. P. (2020). Psychosocial aspects and the impact of oral health on quality of life of Brazilian adults. *Brazilian Journal of Epidemiology, 23*(e200049), 1-13. doi: https://doi.org/10.1590/1980-549720200049

Forstmeier, S., Kuwert, P., Spitzer, C., Freyberger, H. J., & Maercker, A. (2009). Posttraumatic growth, social acknowledgment as survivors, and sense of coherence in former German child soldiers of World War II. *The American Journal of Geriatric Psychiatry, 17*(12), 1030-1039. doi: https://doi.org/10.1097/jgp.0b013e3181ab8b36

Gabrielsen, L. E., Ulleberg, P., & Watten, R. G. (2012). The Adolescent Life Goal Profile Scale: Development of a new scale for measurements of life goals among young people. *Journal of Happiness Studies, 13*(6), 1053-1072. doi: https://doi.org/10.1007/s10902-011-9306-2

Gabrielsen, L. E., Watten, R. G., & Ulleberg, P. (2013). Differences on Adolescent Life Goal Profile Scale between a clinical and non-clinical adolescent sample. *International Journal of Psychiatry in Clinical Practice, 17*(4), 244-252. doi: https://doi.org/10.3109/13651501.2012.745573

Gavulic, A. M. (2018). *Examining the effect of parental attachment, emotional maturity, spiritual maturity, and view of suffering on sense of coherence* [Doctoral dissertation, Liberty University]. ProQuest Dissertations and Theses Global.

Ghazinour, M., Richter, J., & Eisemann, M. (2003). Sense of coherence and psychological health among Iranian refugees resettled in Sweden.

Gibson, L. M. (2000). *Inner resources (sense of coherence, hope, and spiritual perspective) as predictors of psychological well-being in African American breast cancer survivors* [Doctoral dissertation, University of South Carolina]. ProQuest Dissertations and Theses Global.

Goulding, A. (2004). Schizotypy models in relation to subjective health and paranormal beliefs and experiences. *Personality and Individual Differences, 37*(1), 157-167. doi: https://doi.org/10.1016/j.paid.2003.08.008

Goulding, A. (2005). Healthy schizotypy in a population of paranormal believers and experients. *Personality and Individual Differences, 38*(5), 1069-1083. doi: 10.1016/j.paid.2004.07.006

Hammer, J. H., Cragun, R. T., & Hwang, K. (2013). Measuring spiritual fitness: Atheist military personnel, veterans, and civilians. *Military Psychology, 25*(5), 438-451. doi: https://doi.org/10.1037/mil0000010

Hatch, R. L., Burg, M. A., Naberhaus, D. S., & Hellmich, L. K. (1998). The spiritual involvement and beliefs scale: Development and testing of a new instrument. *Journal of Family Practice, 46*(6), 476-486.

Heppner, P. P., Heppner, M. J., Lee, D.-g., Wang, Y.-W., Park, H.-j., & Wang, L.-f. (2006). Development and validation of a collectivist coping styles inventory. *Journal of Counseling Psychology, 53*(1), 107-125. doi: https://doi.org/10.1037/0022-0167.53.1.107

Herbst, L., Coetzee, S., & Visser, D. (2007). Personality, sense of coherence and the coping of working mothers. *SA Journal of Industrial Psychology, 33*(3), 57-67. doi: https://hdl.handle.net/10520/EJC89274

Holland, J. C., Kash, K. M., Passik, S., Gronert, M. K., Sison, A., Lederberg, M., . . . Fox, B. (1998). A brief spiritual beliefs inventory for use in quality of life research in life-threatening illness. *Psycho-Oncology, 7*(6), 460-469. doi: https://doi.org/10.1002/(SICI)1099-1611(199811/12)7:6<460::AID-PON328>3.0.CO;2-R

Hossack, R. C. (1997). *Salutogenic and pathogenic orientations to life: Attachment, personality, sense of coherence and well-being in late adolescence: A structural equation model* [Doctoral dissertation, University of Manitoba]. ProQuest Dissertations and Theses Global.

Israelashvili, M., Taubman-Ben-Ari, O., & Hochdorf, Z. (2011). A multidimensional approach to explore cross-cultural differences in coping behavior: comparing Druze and Jews in Israel. *The Journal of Social Psychology, 151*(1), 31-50. doi: https://doi.org/10.1080/00224540903366651

Johnson-Migalski, L. (2006). *Levels and correlations of sense of coherence, depression and spirituality/religiousness in the medically stable and unstable elderly* [Doctoral dissertation, Adler School of Professional Psychology]. ProQuest Dissertations and Theses Global.

Katz, E. (2000). *The relationship between religious and Israeli identities and attitudes towards the military service among Jewish and Druze adolescents* [Unpublished Master's thesis]. Tel Aviv University.

Kecskes, R., & Wolf, C. (1993). Christliche Religiosität: Konzepte, Indikatoren, Meßinstrumente [Christian religiosity: Concepts, indicators, measurement instruments]. *Kölner Zeitschrift für Soziologie und Sozialpsychologie, 45*(2), 270-287.

Kerksieck, P., Büssing, A., Frick, E., Jacobs, C., & Baumann, K. (2016). Reduced sense of coherence due to neuroticism: Are transcendent beliefs protective among Catholic pastoral workers? *Journal of Religion and Health, 56*(6), 1956-1970.

Khanjari, S., Oskouie, F., & Langiu-Eklöf, A. (2012). Lower sense of coherence, negative religious coping, and disease severity as indicators of a decrease in quality of life in Iranian family caregivers of relatives with breast cancer during the first 6 months after diagnosis. *Cancer Nursing, 35*(2), 148-156. doi: https://doi.org/10.1097/ncc.0b013e31821f1dda

Kibour, Y. (2002). *The moderating role of sense of coherence between migration stress and ajustment among Ethiopian immigrants* [Doctoral dissertation, Howard University]. ProQuest Dissertations and Theses Global.

Kim, S., Zemon, V., & Foley, F. W. (2020). Measuring personal growth in partners of persons with multiple sclerosis: A new scale. *Rehabilitation Psychology, 65*(3), 219-230. doi: https://doi.org/10.1037/rep0000299

King, D. B., & DeCicco, T. L. (2009). A viable model and self-report measure of spiritual intelligence. *International Journal of Transpersonal Studies, 28*(1), 68-85.

Kinjerski, V. (2013). The spirit at work scale: Developing and validating a measure of individual spirituality at work. In J. Neal (Ed.), *Handbook of faith and spirituality in the workplace: Emerging research and practice* (pp. 383-402). New York: Springer.

Klaghofer, R., & Oser, F. (1987). Dimensionen und Erfassung des religiösen Familienklimas [Dimensions and assessment of the religious family climate]. *Unterrichtswissenschaft, 15*(2), 190-206.

Kohls, N., Hack, A., & Walach, H. (2008). Measuring the unmeasurable by ticking boxes and opening Pandora's box? Mixed methods research as a useful tool for investigating exceptional and spiritual experiences. *Archive for the Psychology of Religion, 30*(1), 155-187. doi: https://doi.org/10.1163/157361208X317123

Krok, D. (2016). Sense of coherence mediates the relationship between the religious meaning system and coping styles in Polish older adults. *Aging & Mental Health, 20*(10), 1002-1009. doi: https://doi.org/10.1080/13607863.2015.1056772

Lee, L. E. (1998). *Internal coping resources as predictive of individual outcome in outpatient drug rehabilitation treatment* [Doctoral dissertation, City University of New York]. ProQuest Dissertations and Theses Global.

Littlejohn, E. M. (1994). *The relationship between the components of Black feminism and psychological health in African American women* [Doctoral dissertation, Ohio State University]. ProQuest Dissertations and Theses Global.

López, J., Camilli, C., & Noriega, C. (2015). Posttraumatic growth in widowed and non-widowed older adults: Religiosity and sense of coherence. *Journal of Religion and Health, 54*(5), 1612-1628. doi: 10.1007/s10943-014-9876-5

Magnano, P. A. (2003). *Hope: Building a schema.* Doctoral dissertation, University of Washington.

Meghani, S. H., Peterson, C., Kaiser, D. H., Rhodes, J., Rao, H., Chittams, J., & Chatterjee, A. (2018). A pilot study of a mindfulness-based art therapy intervention in outpatients with cancer. *American Journal of Hospice & Palliative Medicine, 35*(9), 1195-1200. doi: https://doi.org/10.1177/1049909118760304

Mirsoleymani, S., Matbouei, M., Vasli, P., Marzaleh, M. A., & Rohani, C. (2021). The role of family caregiver's sense of coherence and family adaptation determinants in predicting distress and caregiver burden in families of cancer patients. *Indian Journal of Palliative Care, 27*(1), 47-53.

Muthny, F. A. (1989). *Freiburger Fragebogen zur Krankheitsverarbeitung (FKV)* [Freiburg quesstionnaire of coping with illness] Weinheim: Beltz.

Nishi, D., Matsuoka, Y., & Kim, Y. (2010). Posttraumatic growth, posttraumatic stress disorder and resilience of motor vehicle accident survivors. *Biopsychosocial Medicine, 4*(7), 1-6. doi: https://dx.doi.org/10.1186%2F1751-0759-4-7

Nolvi, M., Brogårdh, C., Jacobsson, L., & Lexell, J. (2022). Sense of coherence and coping behaviours in persons with late effects of polio. *Annals of Physical and Rehabilitation Medicine, 65*(3), 101577. https://doi.org/10.1016/j.rehab.2021.101577

Olson, D. H. L. (1983). *Families: What makes them work*: Sage Publications.

Ozaki, M. (2005). Development of an assessment tool on spirituality explained by three domains, Will, joy and sense: From a holistic educational approach. *Journal of International Society of Life Information Science, 23*(2), 364-369.

Pakenham, K. I. (2007). Making sense of caregiving for persons with multiple sclerosis (MS): The dimensional structure of sense making and relations with positive and negative adjustment. *International Journal of Behavioral Medicine, 15*(3), 241-252. doi: 10.1080/10705500802222345

Pakenham, K. I., & Cox, S. (2008). Development of the benefit finding in multiple sclerosis (MS) caregiving scale: A longitudinal study of relations between benefit finding and adjustment. *British Journal of Health Psychology, 13*(4), 583-602. doi: 10.1348/135910707X250848

Paloutzian, R. F., & Ellison, C. (1982). Spiritual well-being scale. In P. C. Hill & R. W. Hood Jr (Eds.), *Measures of religiosity* (pp. 382-385): Religious Education Press.

Piedmont, R. L. (1999). Does spirituality represent the sixth factor of personality? Spiritual transcendence and the five-factor model. *Journal of Personality, 67*(6), 985-1013. doi: https://doi.org/10.1111/1467-6494.00080

Piedmont, R. L. (2010). *Assessment of spirituality and religious sentiments technical manual*. Timonium: Author.

Piedmont, R. L., Magyar-Russell, G., DiLella, N., & Matter, S. (2014). Sense of coherence: Big five correlates, spirituality, and incremental validity. *Current Issues in Personality Psychology, 2*(1), 1-9. doi: https://doi.org/10.5114/cipp.2014.43096

Plante, T. G., & Boccaccini, M. T. (1997). The Santa Clara strength of religious faith questionnaire. *Pastoral Psychology, 45*(5), 375-387. doi: https://doi.org/10.1007/BF02230993

Prężyna, W. (1981). *Funkcja postawy religijnej w osobowości człowieka* [Function of religious attitude in human personality]. Lublin Katol. Uniw.

Post-White, J., Ceronsky, C., Kreitzer, M. J., Nickelson, K., Drew, D., Mackey, K. W., . . . Gutknecht, S. (1996). Hope, spirituality, sense of coherence, and quality of life in patients with cancer. *Oncology Nursing Forum, 23*(10), 1571-1579.

Racklin, J. M. (1999). *The roles of sense of coherence, spirituality, and religion in responses to trauma* [Doctoral dissertation, California School of Professional Psychology at Alameda]. ProQuest Dissertations and Theses Global.

Ragger, K., Hiebler-Ragger, M., Herzog, G., Kapfhammer, H.-P., & Unterrainer, H.-F. (2019). Sense of coherence is linked to post-traumatic growth after critical incidents in Austrian ambulance personnel. *BMC Psychiatry, 19*(89), 1-11. doi: https://doi.org/10.1186/s12888-019-2065-z

Reed, P. G. (1986). Religiousness among terminally ill and healthy adults. *Research in Nursing & Health, 9*(1), 35-41. doi: https://doi.org/10.1002/nur.4770090107

Reguera-García, M. M., Liébana-Presa, C., Álvarez-Barrio, L., Alves Gomes, L., & Fernández-Martínez, E. (2020). Physical activity, resilience, sense of coherence and coping in people with multiple sclerosis in the situation derived from COVID-19. *International Journal of Environmental Research and Public Health, 17*(21), 1-13. doi: https://doi.org/10.3390/ijerph17218202

Renner, W., Salem, I., & Alexandrowicz, R. (2004). Human values as predictors for political, religious and health-related attitudes: A contribution towards validating the Austrian Value Questionnaire (AVQ) by structural equation modeling. *Social Behavior and Personality, 32*(5), 477-490. doi: https://doi.org/10.2224/sbp.2004.32.5.477

Rohani, C., Khanjari, S., Abedi, H.-A., Oskouie, F., & Langius-Eklöf, A. (2010). Health index, sense of coherence scale, brief religious coping scale and spiritual perspective scale: Psychometric properties. *Journal of Advanced Nursing, 66*(12), 2796-2806. doi: https://doi.org/10.1111/j.1365-2648.2010.05409.x

Rothmann, S., & Van Rensburg, P. (2002). Psychological strengths, coping and suicide ideation in the South African Police Services in the North West Province. *SA Journal of Industrial Psychology, 28*(3), 39-49. doi: https://hdl.handle.net/10520/EJC88918

Saroglou, V., & Galand, P. (2004). Identities, values, and religion: A study among Muslim, other immigrant, and native Belgian young adults after the 9/11 attacks. *Identity, 4*(2), 97-132. doi: https://doi.org/10.1207/s1532706xid0402_1

Saroglou, V., & Hanique, B. (2006). Jewish identity, values, and religion in a globalized world: A study of late adolescents. *Identity, 6*(3), 231-249. doi: https://doi.org/10.1207/s1532706xid0603_2

Schonder, M. (2016). *Sense of coherence among religious and non-religious students from Germany and Poland* [Doctoral dissertation, Technische Universität Carolo-Wilhelmina zu Braunschweig].]. LeoPARD TU Braunschweig Publications And Research Data.

Skalski-Bednarz, S. B., Konaszewski, K., Muszyńska, J., Maier, K., & Surzykiewicz, J. (2022). Negative situation appraisal and mental well-being among refugees in Germany: Serial mediation by religious coping and sense of coherence. *International Migration, 00*, 1-13. https://doi.org/10.1111/imig.13087

Skowroński, B., & Talik, E. (2021). Quality of life and its correlates in people serving prison sentences in penitentiary institutions. *International Journal of Environmental Research and Public Health, 18*(4), 1655.

Strümpfer, D. J. W. (1997). The relation between religious motivation and work-related variables amongst agricultural workers. *South African Journal of Psychology, 27*(3), 134-142. doi: https://psycnet.apa.org/doi/10.1177/008124639702700302

Tagay, S., Erim, Y., Brähler, E., & Senf, W. (2006). Religiosity and sense of coherence–Protective factors of mental health and well-being? *Zeitschrift für Medizinische Psychologie, 15*(4), 165-171.

Tedeschi, R. G., & Calhoun, L. G. (1996). The Posttraumatic Growth Inventory: Measuring the positive legacy of trauma. *Journal of Traumatic Stress, 9*(3), 455-471. doi: https://dio.org/10.1007/BF02103658

Tobacyk, J. J. (2004). A revised paranormal belief scale. *The International Journal of Transpersonal Studies, 23*(23), 94-98.

Torinomi, C., Lindenberg, K., Möltner, A., Herpertz, S. C., & Holm-Hadulla, R. M. (2022). Predictors of students' mental health during the COVID-19 pandemic: The impact of coping strategies, sense of coherence, and social support. *International Journal of Environmental Research and Public Health, 19*(24), 16423.

Unterrainer, H.-F., & Ladenhauf, K. H. (2008). Religiös-spirituelles Befinden im Kontext seelischer Gesundheit und Krankheitsverarbeitung: Ergebnisse eines interdisziplinären Forschungsprojekts [Religious-spiritual well-being in the context of mental health and coping with illness: Results of an interdisciplinary research project]. *Psychologie in Österreich, 28*(1), 54-61.

Unterrainer, H.-F., Lewis, A., Collicutt, J., & Fink, A. (2013). Religious/spiritual well-being, coping styles, and personality dimensions in people with substance use disorders. *International Journal for the Psychology of Religion, 23*(3), 204-213. doi: https://doi.org/10.1080/10508619.2012.714999

Uren, T. H., & Wastell, C. A. (2002). Attachment and meaning-making in perinatal bereavement. *Death Studies, 26*(4), 279-308. doi: https://doi.org/10.1080/074811802753594682

Van der Colff, J. J., & Rothmann, S. (2009). Occupational stress, sense of coherence, coping, burnout and work engagement of registered nurses in South Africa. *SA Journal of Industrial Psychology, 35*(1), 1-10. doi: http://dx.doi.org/10.4102/sajip.v35i1.423

Verouli, P., Siafaka, V., & Ageli, A. (2016). Association between the fear of pain, the response strategies and the sense of coherence in workers in primary health care. *International Journal of Caring Sciences, 9*(3), 1106-1116.

Vosloo, C., Wissing, M. P., & Temane, Q. M. (2009). Gender, spirituality and psychological well-being. *Journal of Psychology in Africa, 19*(2), 153-159. doi: https://doi.org/10.1080/14330237.2009.10820274

Wenzl, M., Fuchshuber, J., Podolin-Danner, N., Silani, G., & Unterrainer, H.-F. (2021). The Swedish version of the Multidimensional Inventory for Religious/Spiritual Well-Being: First results from Swedish students. *Frontiers in Psychology, 12*, 783761. https://doi.org/10.3389/fpsyg.2021.783761

Wilkins, T. A., Piedmont, R. L., & Magyar-Russell, G. M. (2012). Spirituality or religiousness: Which serves as the better predictor of elements of mental health? *Research in the Social Scientific Study of Religion, 23*, 53-73.

Wissing, J. A. B., Wissing, M. P., du Toit, M. M., & Temane, Q. M. (2008). Psychometric properties of various scales measuring psychological well-being in a South African context: The FORT 1 Project. *Journal of Psychology in Africa, 18*(4), 511-520. doi: https://doi.org/10.1080/14330237.2008.10820230

Zafar, H., Khan, S. H., Bhatti, M. I., & Hussain, M. M. (2019). Spirituality, sense of coherence, resilience, and stress among earthquake survivors of Azad Jammu and Kashmir. *Pakistan Journal of Social Sciences, 39*(4), 1511-1519.

Zamanian, H., Amini-Tehrani, M., Mahdavi Adeli, A., Daryaafzoon, M., Arsalani, M., Enzevaei, A., & Farjami, M. (2021). Sense of coherence and coping strategies: How they influence quality of life in Iranian women with breast cancer. *Nursing Open, 8*, 1731-1740. doi: https://doi.org/10.1002/nop2.814

Zarzycka, B., & Rydz, E. (2014a). Centrality of religiosity and sense of coherence: A cross-sectional study with polish young, middle and late adults. *International Journal of Social Science Studies, 2*(2), 126-136. doi: http://dx.doi.org/10.11114/ijsss.v2i2.346

Zarzycka, B., & Rydz, E. (2014b). Explaining the relationship between post-critical beliefs and sense of coherence in Polish young, middle, and late adults. *Journal of Religion & Health, 53*(3), 834-848. doi: https://doi.org/10.1007/s10943-013-9680-7

Zehnder Grob, S. (2015). *Religiosität, psychische Gesundheit und Kohärenzsinn: Eine empirische Befragungsstudie Adoleszenter [Religiosity, mental health, and sense of coherence: An empirical survey study of adolescents]* [Doctoral dissertation, Technische Universität Dortmund]. Eldorado - Repositorium der TU Dortmund.

Zerach, G., & Levin, Y. (2018). Posttraumatic stress symptoms, burn-out, and compassion satisfaction among body handlers: The mediating role of sense of coherence and spirituality at workplace. *Journal of Interpersonal Violence, 33*(12), 1931-1957. doi: https://doi.org/10.1177/0886260515621065

Ziebertz, H.-G., Riegel, U., & Heil, S. (2008). *Letzte Sicherheiten: Eine empirische Studie zu Weltbildern Jugendlicher [Last securities: An empirical study of adolescents' worldviews]*. Freiburg-Basel-Wien: Herder.
