# Supplementary material for: Sense of coherence and religion/spirituality: A systematic review and meta-analysis based on a methodical classification of instruments measuring religion/spirituality
Source: PLoS One. 2023 Aug 3;18(8):e0289203. doi: 10.1371/journal.pone.0289203 (PMC10399782; doi:10.1371/journal.pone.0289203)
Supplement: S11 Table — The inductive coding system for coding the dominant aspects of R/S captured by the individual questionnaire items. For intersubjective comprehensibility, the table also includes trigger words, code descriptions, and anchor examples. Parallel to the codes, the more abstract deductive category system resulting from the inductive coding of the items is also presented. (PDF) [file pone.0289203.s015.pdf]

S16 Table. Coding Schema for Classifying Items of the R/S Measures Used in the Studies Included for Meta-Analysis.

| Category | Code | Code description | Trigger words | Anchor example |
|---|---|---|---|---|
| Beliefs & Concepts | Afterlife | *a.* This code should be selected when the item explicitly asks for postmortem concepts.<br>*b.* This code should also be assigned if the wording of the item indirectly presupposes a belief in continued life after death (e.g., communication with deceased). | Afterlife, death, deceased, heaven, hell, immortality, reincarnation, resurrection, soul, spirit | "To what extent do you believe in an afterlife – e.g., immortality of the soul, resurrection of the dead or reincarnation?"<br>Centrality of Religiosity Scale (Huber & Huber, 2012) |
| Beliefs & Concepts | Belief | This code should be selected when the item refers either to a specific religious / spiritual belief or when the question targets religious / spiritual beliefs in general as a more cognitive dimension of R/S. | Beliefs, concepts, faith | "To believe in a God."<br>Adolescent Life Goal Profile Scale (Gabrielsen et al. 2012) |
| Beliefs & Concepts | God concept | *a.* This code should be selected if the item addresses a specific image of God, i.e., if the item directly or indirectly attributes certain characteristics or behaviors to God.<br>*b.* This code should also be selected if the item asks about the belief in the existence of God. | Almighty, existence, father, merciful | "I believe in God as a Heavenly Father who watches over me and to whom I am accountable."<br>Salience: Cognition (King & Hunt, 1975) |
| Beliefs & Concepts | Higher being | This code should be chosen if the transcendent counterpart is explicitly addressed in the item with the somewhat more religiously neutral formulation "higher being" or "supreme being" (and not with Christian terms such as God or divine being). | Higher being, supreme being | "A higher being protects or helps me."<br>Exceptional Experiences Questionnaire (Kohls et al. 2008) |
| Beliefs & Concepts | Paranormal beliefs | This code should be selected when the item refers to religious / spiritual beliefs, practices, or experiences that relate to paranormal phenomena and are often classified within the alternative spectrum of R/S (e.g., psychokinesis, spiritism, witchcraft, etc.). | Esoteric concepts, magic, magical thinking, New Age beliefs, occultism, spiritism, superstitious beliefs | "Witches do exist."<br>Revised Paranormal Beliefs Scale (Tobacyk, 2004) |
| Beliefs & Concepts | Power | *a.* This code should be chosen when the item uses phrases like "divine force", "supreme power" or | Divine power, energy, force, power, higher power, psychic power | "I'm doing my best to deal with the disease and leave the rest to God / a higher power."[e] |

| Category | Code | Code description | Trigger words | Anchor example |
|---|---|---|---|---|
| | | "higher power" to talk about something or someone transcendent in a religion-neutral way.<br>*b.* This code should also be assigned if the item refers in a narrower sense to energy concepts with an affinity to R/S. | | Patient Competence in Coping with Cancer Questionnaire (Aderhold et al. 2019) |
| Beliefs & Concepts | Sacredness | This code should be chosen if the item refers directly or indirectly to the conceptual pair sacred vs. profane or if certain things, places, persons or experiences are described as "sacred" or "holy" in the item. | Holy, profane, sacred, secular | "I do not divide life into sacred and secular; I believe all of life is infused with sacredness."<br>Spiritual Orientation Inventory (Elkins et al. 1988) |
| Beliefs & Concepts | Self-image | This code should be chosen when the item describes religious / spiritual ideas, practices or experiences that contribute to a positive self-image of the believer.<br>- If the item is formulated in such a way that the self-image appears exaggerated or unrealistic, the code *grandiosity* can also be assigned. | Appreciation, esteem, self-concept, self-confidence | "I try hard to grow in understanding of what it means to live as a child of God."<br>Orientation to Growth and Striving (King & Hunt, 1975) |
| Beliefs & Concepts | Truth | This code should be chosen if the item deals with the truth claims of religious / spiritual beliefs.<br>- If the item focuses on the search for the truth in the area of R/S, the code *quest* can also be selected. | Certainty, sure, true, truth, truthful, untrue | "While we can never be quite sure that what we believe is absolutely true, it is worth acting on the probability that it may be."<br>Religious Maturity Scale (Dudley & Cruise, 1990) |
| Coping & Support | Coping | This code should be selected when the item describes an attempt to overcome a problem and / or achieve a positive state through the use of religious / spiritual resources. | Care, comfort, help, needs, relief, strength, stronghold, support | "Prayed to get my mind off of my problems."<br>Brief Religious Coping Scale (Pargament et al. 2000) |
| Coping & Support | Efficacy | *a.* This code should be selected if the item describes a person's feeling, belief, or experience that the use of religious / spiritual resources was effective and / or crowned with success. | Effective, finding, healing, helpful, resolution, successful, wonder | "Found comfort from my religion or spirituality." |

| Category | Code | Code description | Trigger words | Anchor example |
|---|---|---|---|---|
| | | *b.* This code should also be chosen when the item describes a person's feeling, belief, or experience that the intervention of a transcendent power contributed to the success or resolution.<br>- Often this code occurs in the context of religious / spiritual coping strategies rated as successful. In this case, it overlaps with the code *coping*. | | Collectivist Coping Styles Inventory (Heppner et al. 2006) |
| Coping & Support | Pastoral care | This code should be chosen when the item refers to the positive and supportive role of pastoral care.<br>- Depending on the wording of the item, the codes *social support*, *coping* and / or *church* may also be considered. | Chaplaincy, counselling, minister, pastoral care, priest | "I receive comfort and support from a spiritual companion, for example, a pastoral caregiver or friend."<br>Spiritual Assessment Scale (O'Brien, 2004) |
| Coping & Support | Social support | *a.* This code should be given when the item describes a person's feeling, belief or experience of being part of a social community of believers and like-minded people and thereby feeling supported, safe and secure.<br>*b.* This code should also be used when the item describes the social support a person receives from other religiously or spiritually motivated people.<br>- If the item only asks about religious affiliation or identification with the community and does not focus on the support function, the code *affiliation* should be assigned.<br>- If the item describes social support as a religious / spiritual coping strategy, the code *coping* can also be chosen. | Belonging, communality, community, companion, faith group, fellowship, friends, involvement, relationships | "How often do you share with others the problems and joys of living according to your spiritual beliefs?"<br>Spiritual Perspective Scale (Reed, 1986) |
| Emotion & Meaning | Emotion | This code should be selected if religious / spiritual feelings are described in the item or R/S is associated with positive feelings. | Awe, harmony, hope, humility, joy, peace, thankfulness, etc. | "I have had transcendent, spiritual experiences in which I was overcome with a sense of awe, wonder, and reverence." |

| Category | Code | Code description | Trigger words | Anchor example |
|---|---|---|---|---|
| | | - If the item focuses on the religious / spiritual feeling of connectedness, the code *connectedness* should be selected.<br>- If the item focuses on the religious / spiritual concept of forgiveness, select the code *forgiveness*.<br>- If the item focuses on trust in something / someone transcendent, select the code *trust*. | | Spiritual Orientation Inventory (Elkins et al. 1988) |
| Emotion & Meaning | Forgiveness | This code should be chosen when the feeling of forgiveness is understood as a religious / spiritual concept in the item and / or the act of forgiveness is religiously / spiritually motivated. | Apology, forgiveness | "Forgiveness is an important part of my spirituality."<br>Spiritual Perspective Scale (Reed, 1986) |
| Emotion & Meaning | Future | This code should be selected if the item describes religious / spiritual concepts, feelings or thoughts that relate to the future.<br>- Depending on the wording of the item, the codes *optimism* and / or *trust* may also be used. | Destiny, fate, future, hope, plan | "Faith in God gives me confidence in looking at the future."<br>Religious Hope (Magnano, 2003) |
| Emotion & Meaning | Guidance | This code should be chosen when the item describes a person's feeling, belief or experience of being led by something / someone transcendent. | Guidance, leading | "I seek spiritual guidance in making decisions in my everyday life."<br>Spiritual Perspective Scale (Reed, 1986) |
| Emotion & Meaning | Inspiration | This code should be chosen when the item describes a person's feeling, belief or experience of being inspired by something or someone transcendent. | Creativity, insight, inspiration | "I look to my faith as a source of inspiration."<br>Santa Clara Strength of Religious Faith Questionnaire (Plante & Boccaccini, 1997) |
| Emotion & Meaning | Meaning | This code should be chosen when the item describes R/S as a source of fulfillment and meaning in life. This | Answers, calling, fulfillment, meaning, mission, not just | "My spirituality is especially important to me because it |

| Category | Code | Code description | Trigger words | Anchor example |
|---|---|---|---|---|
| | | may be related to a person's feeling or belief that he / she is called or is following a mission. | coincidence, order, purpose, satisfaction, sense | answers many questions about the meaning of life.” Spiritual Perspective Scale (Reed, 1986) |
| Emotion & Meaning | Optimism | This code should be chosen if the item describes a religiously / spiritually based attitude characterized by a positive outlook on life and confidence.<br>- If the item refers in a narrower sense to trust in a transcendent power, the code *trust* should be assigned. | Assurance, confidence, hopeful, positivity, sanguine | “While there is much evil in the world, I believe goodness, integrity and love also abound.” Spiritual Orientation Inventory (Elkins et al. 1988) |
| Emotion & Meaning | Trust | This code should be chosen when the item describes trust in something or someone transcendent.<br>- If the trust does not refer to a transcendent source in the narrower sense, but rather describes a religious / spiritually determined positive attitude, the code *optimism* is appropriate. | Confidence, faith, reliance, trust | “Whatever happens, I will trust in a higher power that carries me through.” Reliance on God’s help (Büssing et al. 2015) |
| Emotion & Meaning | Understanding | This code should be selected if the item describes a person's feeling, belief or experience of understanding life, the environment or him / herself better through her / his religious or spiritual beliefs.<br>- If the item focuses on meaning-oriented understanding, the code *meaning* can also be assigned.<br>- If the item does not focus on emotional or meaning-oriented understanding, but on intellectual comprehension promoted by religious / spiritual knowledge, the code *intellect* should be assigned instead. | Information (not rational), meaning, understanding | “Some experiences can be understood only through one’s spiritual beliefs.” Spiritual Involvement and Belief Scale (Hatch et al. 1998) |
| Connectedness & Experience | Awareness | *a.* This code should be selected when the item describes the feeling, belief or experience of becoming aware of something / someone transcendent. | Awareness, consciousness, presence, recognition, sense | “I am aware of God’s presence in times of need.” |

| Category | Code | Code description | Trigger words | Anchor example |
|---|---|---|---|---|
| | | *b.* This code should also to be selected if the item addresses higher states of consciousness. | | Spiritual Assesment Inventory (Hall & Edwards, 2002) |
| Connectedness & Experience | Communication | This code should be chosen when the item describes the feeling, belief or experience of being in communication with something or someone transcendent. | Asking, communicating, contact, listening, responding, revealing, speaking, telling | "Listening to God is an essential part of my life." Spiritual Assesment Inventory (Hall & Edwards, 2002) |
| Connectedness & Experience | Connectedness | This code should be chosen when the item describes a person's feeling, belief, or experience of being connected to something or someone transcendent.<br>- If the connectedness refers to the person's inner self, the code *self* should also be selected.<br>- If the connection is to God or something divine, the code *God relationship* should also be selected.<br>- If the connectedness refers to humanity or the universe or other abstract entities, additionally select the code *universality*. | Close, connection, in touch, near | "I am in touch with everything." Exceptional Experiences Questionnaire (Kohls et al. 2008) |
| Connectedness & Experience | Experience | This code should be chosen if the item explicitly describes an experience that is religiously / spiritually interpreted or connoted. | experience | "How often do you experience situations in which you have the feeling that God or something divine wants to communicate or to reveal something to you?" Centrality of Religiosity Scale (Huber & Huber, 2012) |
| Connectedness & Experience | Extraordinary perception | This code should be selected if the item describes an experience that was accompanied by extraordinary sensory perceptions and can be interpreted in religious / spiritual terms. | Audition, discern, inner voice, light, perceive, vision | "Benign light surrounds me." Exceptional Experiences Questionnaire (Kohls et al. 2008) |

| Category | Code | Code description | Trigger words | Anchor example |
|---|---|---|---|---|
| | | - If the religious / spiritual experience described does not include an extraordinary sensory perception, the code *experience* should be assigned instead. | | |
| Connectedness & Experience | God relationship | This code should be chosen if the item directly or indirectly addresses the nature, quality and / or strength of the human-God relationship.<br>- If the feeling of God-connectedness is in the foreground, the code *connectedness* should be chosen instead. | Relation, relationship | "I have a personally meaningful relationship with God."<br>Spiritual Well-Being Scale (Paloutzian & Ellison, 1982) |
| Connectedness & Experience | Influence | This code should be chosen when the item describes a person's feeling, belief or experience that an external transcendent power is interfering with her / his internal or external life. | Impact, influence, interference, intervention | "A spiritual force influences the events in my life."<br>Spiritual Involvement and Belief Scale (Hatch et al. 1998) |
| Connectedness & Experience | Mysticism | *a.* This code should be chosen when the item describes a person's feeling or experience of merging or being one with something or someone transcendent.<br>*b.* This code should also be chosen if the item describes the opinion that certain religious / spiritual experiences are beyond rational explanation or description in words. | Beyond intellect, beyond words, communion, inexplicability, mystery, oneness, union, wholeness | "I have had transcendent, spiritual experiences which seem almost impossible to put into words."<br>Spiritual Orientation Inventory (Elkins et al. 1988) |
| Connectedness & Experience | Nature | This code should be chosen when the item describes a person's feeling, belief or experience of being in contact with something or someone transcendent in nature. | Creation, nature, outdoors | „In the great outdoors I feel the presence of God".[d]<br>Multidimensional Instrument for the Measurement of Religious-Spiritual Well-Being (Unterrainer, 2007) |
| Connectedness & Experience | Self-transcendence | This code should be given when the item describes a person's feeling, belief, or experience that there is something greater outside of his / her limited self and | Ego loss, deconstruction, greater source, selfless | "I have had transcendent, spiritual experiences in which I |

| Category | Code | Code description | Trigger words | Anchor example |
|---|---|---|---|---|
| | | that this greater power removes the boundaries of oneself.<br>- If the item refers to the inexplicability of this experience and / or emphasizes the ineffable feeling of oneness, the code *mysticism* should be given. | | 'let go' and surrendered my life to something higher."<br>Spiritual Orientation Inventory (Elkins et al. 1988) |
| Connectedness & Experience | Universality | Select this code if the item describes a religiously or spiritually interpreted connection to an abstract larger entity such as humanity or the universe.<br>- If this feeling or belief of connectedness relates to a transcendent being, the codes *connectedness*, *higher being*, *God relationship* or *power* should also be considered - depending on the formulation of the item.<br>- If this universal connectedness represents a unity experience that cannot be expressed in words, the code *mysticism* can also be chosen.<br>- If the focus is on transcending one's own ego boundaries, *self-transcendence* can also be coded. | All creatures, all life, all of us, common bond, God's family, humankind, humanity, universe, world | "I feel that on a higher level all of us share a common bond.<br>Assessment of Spirituality and Religious Sentiments (Piedmont, 2010) |
| Autonomy & Quest | Autonomy | *a.* This code should be selected when the item addresses independence in matters of faith and / or autonomous decision-making in religious / spiritual matters.<br>*b.* This code should also be selected when the item emphasizes religious / spiritual individualism as opposed to collective forms of organized R/S. | Autonomy, choice, decision, independence, individualism | "My father lets me make my own decisions about religion and faith."[a]<br>Religious Autonomy (Zehnder Grob, 2015) |
| Autonomy & Quest | Control | This code should be chosen when the item describes a person's feeling, belief, or experience of being in control of herself / himself, external circumstances, and / or someone / something transcendent. | Control, impact, influence, manage, manipulation, regulation, responsibility, self-control | "I seem to have a unique ability to influence God through my prayers." |

| Category | Code | Code description | Trigger words | Anchor example |
|---|---|---|---|---|
| | | - This code should not be selected if the item describes the opposite experience of a person, namely the feeling or belief that something or someone transcendent controls one's life. In this case, the code *influence* should be selected. | | Spiritual Assessment Inventory (Hall & Edwards, 2002) |
| Autonomy & Quest | Development | *a.* This code should be chosen when the item describes a person's desire, willingness, or experience to develop through faith.<br>*b.* This code should also be selected if the item describes a person's desire, willingness, or experience to develop her / his religious / spiritual belief system over time. | Growth, change, evolvement, development, transformation | "As I grow older and change, I expect my religion also to grow and change."<br>Religious Life Inventory (Batson & Ventis, 1982) |
| Autonomy & Quest | Grandiosity | *a.* This code should be selected when the item deals with the (unrealistic or exaggerated) conviction of being superior to other people in the field of R/S.<br>*b.* This code should also be selected if the item deals with the conviction of being able to exert an extraordinarily strong influence on the transcendental realm.<br>- If the conviction that one has an influence on transcendent realities does not seem excessive, the code *control* can also be chosen. | Unique, more than, special, better than, superior | "God understands that my needs are more important than most people's."<br>Spiritual Assesment Inventory (Hall & Edwards, 2002) |
| Autonomy & Quest | Individual practice | This code should be selected if the item formulation shows that the religious / spiritual practice described is primarily carried out individually or in the private sphere. | Devotion, meditation, private, prayer, reading, study, use of media | "How often do you spend time in private religious activities, such as prayer, meditation or Bible study?"<br>Duke University Religion Index (Koenig et al. 1997) |
| Autonomy & Quest | Self | This code should be chosen when the item describes a person's feeling, belief or experience that he / she is in contact with his / her deeper inner core or self. | Core, higher self, inner spirit, inner voice | "Meditation does not help me feel more in touch with my inner spirit." |

| Category | Code | Code description | Trigger words | Anchor example |
|---|---|---|---|---|
| | | | | Spiritual Involvement and Belief Scale (Hatch et al. 1998) |
| Autonomy & Quest | Intellect | This code should be chosen if the item focuses on intellectual preoccupation with religious / spiritual issues. | Knowledge, information, interest, insight, learning, opinion, reading, study, thinking | "How often do you think about religious issues?" Centrality of Religiosity Scale (Huber & Huber, 2012) |
| Autonomy & Quest | Quest | This code should be chosen if the item describes a searching and questioning attitude that not only includes change, criticism, doubt and uncertainty, but also values them positively as part of a mature and reflexive R/S.<br>- If the item clearly emphasizes the developmental aspect, the code *development* can be assigned instead.<br>- If the item emphasizes intellectual processes, i.e., less the searching and questioning attitude and more the acquisition of information and knowledge about R/S, the code *intellect* should be chosen.<br>- If the religious/spiritual struggle associated with a quest orientation is not positively evaluated, but the burdening factor is the focus, the code *negative aspects* should be assigned instead. | Flexibility, openness, problems and joys, questioning, readiness for change, rethinking, search, struggle (positive) | "For me, doubting is an important part of what it means to be religious." Quest Scale (Batson & Schoenrade, 1991) |
| Negativity | Negative aspects | *a.* This code should be chosen when the item describes negative feelings such as anger, disappointment or doubt that are related to R/S.<br>*b.* This code should also be chosen if the item emphasizes religious / spiritual concepts that can | Anger, bad luck, black magic, demons, devil, disappointment, dryness, frustration, hell, irritation, pain, pressure, sin, | "Believed the devil was responsible for my situation." Religious Coping Scale (Pargament et al. 2000) |

| Category | Code | Code description | Trigger words | Anchor example |
|---|---|---|---|---|
| | | trigger negative feelings or thoughts in believers (e.g., sin or hell).<br>*c.* This code should also be chosen if the item focuses on negative religious / spiritual beings or forces (e.g., devil or black magic). | struggle (negative), theodicy, witchcraft | |
| Institutionality & Collectivity | Affiliation | This code should be selected when the item asks whether a person belongs to a church or religious / spiritual community. | Affiliation, belonging, denomination, membership | "Do you presently belong to a church (or synagogue)?"<br>Religious Practice Dimension (De Jong et al. 1976) |
| Institutionality & Collectivity | Churchiness | This code should be selected if the item addresses the topic of church in any way or places R/S in church contexts. | Church, ecclesiastic | "How often do you attend church or other religious meetings?"<br>Duke University Religion Index (Koenig et al. 1997) |
| Institutionality & Collectivity | Collective practice | This code should be selected if the item addresses religious / spiritual practices or religion-related activities that are performed collectively or communally. | Attendance, collective, group, meetings, organizations, participation, public, ritual, services, worship | "In how many religious affiliated organizations, groups, or activities (such as choir, youth groups, committees, and boards, etc.) do you participate?"<br>Religious Practice Dimension (De Jong et al. 1976) |
| Institutionality & Collectivity | Organized religion | This code should be chosen if the item refers to institutionalized forms of R/S in a positive and generalized way.<br>- If the item refers to a church community according to Christian understanding, the code *churchiness* should be assigned.<br>- If the item focuses on common religious/spiritual activities, the code *collective practice* should be assigned. | Denomination, institution, organization, organized | "Organized religion is important in my life."<br>Religious Importance Scale (Racklin, 1999) |

| Category | Code | Code description | Trigger words | Anchor example |
|---|---|---|---|---|
| | | - If the item refers to the social support a person receives through organized forms of R/S, the code *social support* can additionally be selected. | | |
| Centrality & Frequency | Centrality | *a.* This code should be selected if the item explicitly addresses the personal or general importance of R/S. *b.* This code should also be selected when the item asks how much the person identifies with his / her church or religious / spiritual community. | Central, essential, identification, important, loyalty, profound, significant | "My spirituality is a significant part of my life." Spiritual Perspective Scale (Reed, 1986) |
| Centrality & Frequency | Compliance | *a.* This code should be selected when the item describes beliefs and behaviors that are consistent with the religious / spiritual convictions of the person and/or the observances of the religious / spiritual community. *b.* This code should also be selected if the item describes a person's desire or attitude to follow the will of something or someone transcendent. | Accordance, compliance, convictions, dogmatism, God's will, observance, orthodoxy, requirements, rules, subordination, surrender | "How much do you live by religious precepts in your daily life?"[b] Everyday Relevance of Religiosity (Zehnder Grob, 2015) |
| Centrality & Frequency | Effort | This code should be selected when the item describes a person who makes efforts to achieve religious / spiritual goals or to live up to her / his religious / spiritual values and ideals. | Attempt, desire, effort, focus, try, will | "I try hard to carry my religion over into all other dealings in life." Duke University Religion Index (Koenig et al. 1997) |
| Centrality & Frequency | Family resource | This code should be selected if the item represents R/S as a family resource or if the item is about the R/S of family members. | Family, kindred, parents, relatives | "When we face problems or crises in our family, we respond by: Attending church services..." Family Crisis-Oriented Personal Evaluation Scales (McCubbin et al. 2013) |
| Centrality & Frequency | Frequency | This code should be selected if the item asks how frequently a religious / spiritual behavior occurs or a religious / spiritual practice is performed. Special | Always, day, frequently, occasionally, often, month, never, rarely, | "How frequently do you attend religious services?" |

| Category | Code | Code description | Trigger words | Anchor example |
|---|---|---|---|---|
| | | attention should be paid not only to the wording of the question (e.g., How often...?), but also to the response format (e.g., always, often, rarely, never). | regularly, seldom, week, year | Assessment of Spirituality and Religious Sentiments (Piedmont, 2010) |
| Centrality & Frequency | Identity | *a.* This code should be given if the item describes R/S as an essential part of identity and / or personality development.<br>*b.* This code is also chosen when an item asks for a self-description as a religious or spiritual person. | Identity, person, personality | "My religious development has emerged out of my growing sense of personal identity."<br>Religious Life Inventory (Batson & Ventis, 1982) |
| Centrality & Frequency | Socialization | This code should be given if the item describes that and how a person has been shaped and socialized in her / his faith by other people or a religious / spiritual community.<br>- If the educational aspect is in the foreground, i.e., the acquisition of religious / spiritual knowledge, the code *intellect* can also be selected.<br>- If the item emphasizes the developmental aspect, the code *development* can also be assigned.<br>- Some item formulations also suggest choosing the code *family resource* as well. | Education, parents, teaching, role models | "A major factor in my religious development has been the importance of religion for my parents."<br>Religious Life Inventory (Batson & Ventis, 1982) |
| Centrality & Frequency | Way of life | This code should be chosen if the item describes R/S as a way of life, i.e., as a factor that permeates everyday activities and decisions.<br>- If the question is obviously aimed at finding out how central R/S is in a person's life, the code *centrality* should also be assigned. | Approach to life, daily affairs, everyday activity, life, life style, way of life | "My religious beliefs are what really lie behind my whole approach to life."<br>Duke University Religion Index (Koenig et al. 1997) |
| Centrality & Frequency | Workplace | Choose this code when the item asks to what extent R/S plays a role in a person's everyday work or workplace. | Employment, job, labor, occupation, profession, work, workplace | "I experience a connection with a greater source that has a positive effect on my work." |

| Category | Code | Code description | Trigger words | Anchor example |
|---|---|---|---|---|
| | | | | Spirit at Work Scale (Kinjerski, 2013) |
| Social Affairs & Values | Ethics | *a.* This code should be selected if the item describes moral-ethical feelings or behaviors that are religiously / spiritually motivated.<br>*b.* This code should also be selected if the item describes the view that R/S is a value system that provides guidance in making ethical decisions. | Evil, good, highest good, morality, norms, right, wrong | "Religion provides answers to the question of what is right and wrong."[c]<br>Religious Meaning Questionnaire (Krok, 2016) |
| Social Affairs & Values | Idealism | This code should be chosen if the item describes a religiously / spiritually motivated attitude or behavior that critically reflects, relativizes or rejects materialistic goods or attitudes. | Ideals, opposite of materialism, values | "It is much more important to pursue spiritual goals than to pursue money and possessions."<br>Spiritual Orientation Inventory (Elkins et al. 1988) |
| Social Affairs & Values | Plurality | This code should be selected if the item addresses an attitude of openness and tolerance towards other religions, worldviews and values. | Differences, tolerance, openness, acceptance | "Do you think it's good that people with different religions live here?"[d]<br>Religious Pluralism (Zehnder Grob, 2015) |
| Social Affairs & Values | Prosociality | *a.* This code should be chosen if the item describes a religiously or spiritually motivated behavior that aims to contribute to the well-being of another person or the community.<br>*b.* This code should also be selected when an item asks about a person's willingness to donate or about financial contributions to a religious community. | Altruism, charity, donations, helping behavior, kindness, social or political engagement, volunteering | "I find satisfaction in religiously motivated activities other than attending worship services, for example, volunteer work or being kind to others."<br>Spiritual Assessment Scale (O'Brien, 1999) |
| Social Affairs & Values | Retrospection | *a.* This code should be chosen if the item deals with past attitudes and experiences and it is primarily about a retrospective evaluation of one's own life history from a religious / spiritual point of view. | Assessment, evaluation, past, previous, re-examination, reflection | "I examine my actions to see if they reflect my values."<br>Spiritual Involvement and Belief Scale (Hatch et al. 1998) |

| Category | Code | Code description | Trigger words | Anchor example |
|---|---|---|---|---|
| | | *b.* This code should also be chosen if the item asks for a retrospective evaluation or assessment of previous religious / spiritual beliefs and experiences. | | |
| Social Affairs & Values | Social interaction | This code should be given when the item describes social interactions that are either religious / spiritually motivated, have R/S as topic, or take place in a religious / spiritual domain.<br>- If the focus is on the help that a person expects or receives through these social activities, the code *social support* can also be selected.<br>- If the core of the social interactions consists of religious / spiritual practices carried out together, then the code *collective practice* should be assigned instead. | Sharing, social activity, talking | "The church is most important as a place to formulate good social relationships."<br>Religious Orientation Scale (Allport & Ross, 1967) |

[a] Translated by first author. Wording of the original German item: "Mein Vater lässt mich meine eigenen Entscheidungen treffen über Religion und Glauben." [b] Translated by first author. Wording of the original German item: "Wie stark lebst du in deinem Alltag nach religiösen Geboten?" [c] Translated by first author. Wording of the original Polish item: "Religia dostarcza odpowiedzi na pytanie, co jest dobre, a co złe." [d] Translated by first author. Wording of the original Austrian item: "In der freien Natur spüre ich die Gegenwart Gottes." [e] Translated by first author. Wording of the original German item: "Ich tue mein Bestes, um mit der Erkrankung fertig zu werden, und überlasse den Rest Gott / einer höheren Macht."

## References

Aderhold, C., Morawa, E., Paslakis, G., & Erim, Y. (2019). Entwicklung und Validierung eines Fragebogens zur Patientenkompetenz im Umgang mit einer Krebserkrankung (PUK). *Zeitschrift für Psychosomatische Medizin und Psychotherapie, 65*(3), 239-256. doi: https://doi.org/10.13109/zptm.2019.65.3.239

Allport, G. W., & Ross, J. M. (1967). Personal religious orientation and prejudice. *Journal of Personality and Social Psychology, 5*(4), 432-443. doi: https://doi.org/10.1037/0022-3514.5.4.432

Batson, C. D., & Schoenrade, P. A. (1991). Measuring religion as quest: 1) Validity concerns. *Journal for the Scientific Study of Religion, 30*(4), 416-429. doi: https://doi.org/10.2307/1387277

Batson, C. D., & Ventis, W. L. (1982). *The religious experience: A social-psychological perspective*: Oxford University Press.

Büssing, A., Recchia, D. R., & Baumann, K. (2015). Reliance on God's Help Scale as a measure of religious trust: A summary of findings. *Religions, 6*(4), 1358-1367. doi: https://doi.org/10.3390/rel6041358

De Jong, G. F., Faulkner, J. E., & Warland, R. H. (1976). Dimensions of religiosity reconsidered: Evidence from a cross-cultural study. *Social Forces, 54*(4), 866-889. doi: https://doi.org/10.2307/2576180

Dudley, R. L., & Cruise, R. J. (1990). Measuring religious maturity: A proposed scale. *Review of Religious Research, 32*(2), 97-109. doi: https://doi.org/10.2307/3511758

Elkins, D. N., Hedstrom, L. J., Hughes, L. L., Leaf, J. A., & Saunders, C. (1988). Toward a humanistic-phenomenological spirituality: Definition, description, and measurement. *Journal of Humanistic Psychology, 28*(4), 5-18. doi: https://doi.org/10.1177/0022167888284002

Gabrielsen, L. E., Ulleberg, P., & Watten, R. G. (2012). The Adolescent Life Goal Profile Scale: Development of a new scale for measurements of life goals among young people. *Journal of Happiness Studies, 13*(6), 1053-1072. doi: https://doi.org/10.1007/s10902-011-9306-2

Hall, T. W., & Edwards, K. J. (2002). The Spiritual Assessment Inventory: A theistic model and measure for assessing spiritual development. *Journal for the Scientific Study of Religion, 41*(2), 341-357. doi: https://doi.org/10.1111/1468-5906.00121

Hatch, R. L., Burg, M. A., Naberhaus, D. S., & Hellmich, L. K. (1998). The spiritual involvement and beliefs scale: Development and testing of a new instrument. *Journal of Family Practice, 46*(6), 476-486.

Heppner, P. P., Heppner, M. J., Lee, D.-g., Wang, Y.-W., Park, H.-j., & Wang, L.-f. (2006). Development and validation of a collectivist coping styles inventory. *Journal of Counseling Psychology, 53*(1), 107-125. doi: https://doi.org/10.1037/0022-0167.53.1.107

Huber, S., & Huber, O. W. (2012). The centrality of religiosity scale (CRS). *Religions, 3*(3), 710-724. doi: https://doi.org/10.3390/rel3030710

King, M. B., & Hunt, R. A. (1975). Measuring the religious variable: National replication. *Journal for the Scientific Study of Religion, 14*(1), 13-22. doi: https://doi.org/10.2307/1384452

Kinjerski, V. (2013). The spirit at work scale: Developing and validating a measure of individual spirituality at work. In J. Neal (Ed.), *Handbook of faith and spirituality in the workplace: Emerging research and practice* (pp. 383-402). New York: Springer.

Koenig, H. G., Parkerson Jr., G. R., & Meador, K. G. (1997). Religion index for psychiatric research. *American Journal of Psychiatry, 154*(6), 885-886. doi: https://doi.org/10.1176/ajp.154.6.885b

Kohls, N., Hack, A., & Walach, H. (2008). Measuring the unmeasurable by ticking boxes and opening Pandora's box? Mixed methods research as a useful tool for investigating exceptional and spiritual experiences. *Archive for the Psychology of Religion, 30*(1), 155-187. doi: https://doi.org/10.1163/157361208X317123

Krok, D. (2016). Sense of coherence mediates the relationship between the religious meaning system and coping styles in Polish older adults. *Aging & Mental Health, 20*(10), 1002-1009. doi: https://doi.org/10.1080/13607863.2015.1056772

Magnano, P. A. (2003). *Hope: Building a schema* [Doctoral dissertation, University of Washington]. ProQuest Dissertations and Theses Global.

McCubbin, H. I., Olson, D. H. L., & Larsen, A. S. (2013). Family crisis-oriented personal evaluation scales *Handbook of Measurements for Marriage and Family Therapy* (pp. 199-203): Routledge.

O'Brien, M. E. (1999). *Spirituality in nursing: Standing on holy ground*. Sudbury: Jones & Bartlett Learning.

Paloutzian, R. F., & Ellison, C. (1982). Spiritual well-being scale. In P. C. Hill & R. W. Hood Jr (Eds.), *Measures of religiosity* (pp. 382-385): Religious Education Press.

Pargament, K. I., Koenig, H. G., & Perez, L. M. (2000). The many methods of religious coping: Development and initial validation of the RCOPE. *Journal of Clinical Psychology, 56*(4), 519-543. doi: https://doi.org/10.1002/(sici)1097-4679(200004)56:4<519::aid-jclp6>3.0.co;2-1

Piedmont, R. L. (2010). *Assessment of spirituality and religious sentiments technical manual*. Timonium: Author.

Plante, T. G., & Boccaccini, M. T. (1997). The Santa Clara strength of religious faith questionnaire. *Pastoral Psychology, 45*(5), 375-387. doi: https://doi.org/10.1007/BF02230993

Racklin, J. M. (1999). *The roles of sense of coherence, spirituality, and religion in responses to trauma* [Doctoral dissertation, California School of Professional Psychology at Alameda]. ProQuest Dissertations and Theses Global.

Reed, P. G. (1986). Religiousness among terminally ill and healthy adults. *Research in Nursing & Health, 9*(1), 35-41. doi: https://doi.org/10.1002/nur.4770090107

Tobacyk, J. J. (2004). A revised paranormal belief scale. *The International Journal of Transpersonal Studies, 23*(23), 94-98.

Unterrainer, H.-F. (2007). *Spiritualität und psychische Gesundheit: Glaube als Ressource in der Krankheitsverarbeitung [Spirituality and mental health: faith as a resource in coping with illness]*. Saarbrücken: VDM Verlag.

Zehnder Grob, S. (2015). Religiosität, psychische Gesundheit und Kohärenzsinn: Eine empirische Befragungsstudie Adoleszenter [Religiosity, mental health, and sense of coherence: An empirical survey study of adolescents] [Doctoral dissertation, Technische Universität Dortmund]. Eldorado - Repositorium der TU Dortmund. doi: http://dx.doi.org/10.17877/DE290R-17714
