# Supplementary material for: Sense of coherence and religion/spirituality: A systematic review and meta-analysis based on a methodical classification of instruments measuring religion/spirituality
Source: PLoS One. 2023 Aug 3;18(8):e0289203. doi: 10.1371/journal.pone.0289203 (PMC10399782; doi:10.1371/journal.pone.0289203)
Supplement: S1 Tool — Self-developed critical appraisal tool to check how well a study meets the methodological requirements or how well it fits the context of our research question. (PDF) [file pone.0289203.s016.pdf]

S6 Tool. Critical Appraisal Tool.

Name of appraiser		Date:	
Study (author/s and year)			

Each question is phrased in such a way that "yes" is a positive answer and "no" is a negative answer. However, to answer the questions more precisely a Likert scale ranging from 0-2 is used. If you would answer with a clear "no", then you tick the box "0". If you would answer with "to some extent" or "partly" then you tick the box "1". And if you would answer with an explicit "yes" then you tick the box "2". You can also choose "don't know" if you are not able to answer the question. At the end of each sub-scale you add the points of the ticked boxes to a total score. A high score indicates high fit with the research focus of the meta-analysis.

**Item**	**Source**	**Question**	**0**	**1**	**2**	**Don't know**
1st Domain: Study population						
1.1	SURE	Were participants fairly selected? Consider: eligibility criteria; sources & selection of participants.				
1.2	BIOCROSS	Were the study population characteristics (i.e., demographic, clinical and social) presented?				
1.3	own	Was the religious affiliation of the participants reported?				
1.4	AXIS	If appropriate, was a satisfactory response rate achieved and/or was information about non-responders described?				
1.5	own + BIOCROSS	Was the sample size sufficiently large (>100 = 0, 100-300 = 1, <300 = 2)? Or: Was sample size justification or power description provided?				
**Total score for the 1st domain:**						(range: 0-10)
2nd Domain: R/S measure						
2.1	own	Were the key concepts (i.e. religiosity, spirituality) defined, sufficiently explained and/or differentiated?				
2.2	own	Did the author/s use a multi-item scale to measure R/S? (single-item = 0, two items = 1, at least three items = 2)				
2.3	own	Did the scale show satisfying reliability? (Cronbach's α, test-retest and/or split-half reliability: <.6 = 0, .6 to .7 = 1, <.7 = 2)				
2.4	own	Are other indicators of validity reported besides reliability? In this study and/or in psychometric evaluations published elsewhere?				
**Total score for the 2nd domain:**						(range: 0-8)
3rd Domain: Data analysis						
3.1	BIOCROSS	Was the raw effect size estimate (correlation coefficient, beta coefficient) or measure of study precision provided (e.g., confidence intervals, precise (!) *p*-value*)?				
3.2	own	Did the author/s discuss the R/S-SOC association in any way (beyond the mere notification of an effect size)?				
3.3	own	Did the author/s report any funding sources and/or exclude conflicts of interest that may affect the interpretation of the results?				
**Total score for the 3rd domain:**						(range: 0-6)

There are two ways to report the total score of this appraisal tool. Either you add the scores of the three sub-scales to one total score (ranging from 0-24) or you use a tripartite structure so that the sub-scores remain reproducible (e.g., 4/3/6). **Please note the total score here: ____________________**

Total score of 2nd appraiser		Interrater agreement	
