# Supplementary material for: Sense of coherence and religion/spirituality: A systematic review and meta-analysis based on a methodical classification of instruments measuring religion/spirituality
Source: PLoS One. 2023 Aug 3;18(8):e0289203. doi: 10.1371/journal.pone.0289203 (PMC10399782; doi:10.1371/journal.pone.0289203)
Supplement: S1 Text — A text explaining the methodological subtleties of classifying measures to be considered as measurement instruments for R/S in the context of our meta-analysis. (PDF) [file pone.0289203.s017.pdf]

S8 Text. Methodological Intricacies in the Classification of Potential R/S Measurement Instruments.

Even with the help of a coding aid, which makes it easier to look for "trigger words" in the R/S questionnaires, the item-specific classification process – like every procedure of qualitative content analysis – remains a time-consuming and subjective undertaking. To make the decision-making process more transparent, each item is classified independently by two authors. If different opinions prevail in the classification, the author team discusses until a consensus is reached.

Often the decisions are made quickly and do not need discussion. This is the case, when a questionnaire item explicitly contains terms such as “religious” or “spiritual”. For example, DeBruyn (2001, p. 49) uses the following single-item measure in her study: “How spiritual a person do you consider yourself to be? By spiritual, I mean, having a sense of meaning, purpose, and direction?” This item is classified as S (“spirituality”) and since DeBruyn’s *Degree of Spirituality* scale consists of only this one item, the overall measure is also classified as an S-scale.

Rather debatable is the fact that we treat, for example, a polysemic and context-dependent concept such as “God” as a “trigger word” for “religion” (R). However, it has been shown that the concept of God (especially with the usually capitalized G) is based on Judeo-Christian presuppositions and is often structurally or referentially related to Christian conceptions of institutionalized religion (Pyysiäinen & Ketola, 1999, pp. 208-209). In terms of cognitive science, the concept of God activates certain religious/theological concepts or mental representations for most Christian Westerners or monotheistically oriented people (Barrett, 1999; Lindeman, Pyysiäinen, & Saariluoma, 2002). If this linkage with the Judeo-Christian tradition is to be deliberately avoided in a questionnaire, then the term “God” is often flanked or even replaced with an alternative concept. This is the reason why in our classification scheme the term “God” rather points to the pole “religion” and terms like “divine being”, “higher being” or “higher power” with increasing tendency point to the pole “spirituality”.

If an item contains both “trigger words” that can be assigned to the religion pole and “trigger words” that belong more to the spirituality pole, careful consideration must be given to whether one concept predominates. Otherwise, the item can of course be coded as a mixed item (RS/SR or SR/RS).

For example, the *Daily Spiritual Experience Scale* (Underwood & Teresi, 2002) contains the following item: “I am spiritually touched by the beauty of creation”. While the phrase "spiritually touched" clearly suggests an S-coding, the expression “beauty of creation” could also argue for an R-coding, since the idea of creation is a traditionally religious concept that presupposes an (often divinely conceived) creator. However, since the item does not speak of God's creation, and the religious aspect therefore only implicitly plays a role, the emphasis is on the adverb “spiritually”. Consequently, the item would be coded as SR-item. If the scale authors had used the phrase "beauty of nature" instead of "beauty of creation," the item would have been unambiguously classified as an S-item. In all these individual decisions, the entire wording of the item and the explanatory context of the questionnaire must always be taken into account in order to be able to make an assignment, which is why an intensive qualitative and intersubjectively designed interpretation process appeared to be of higher quality than a mechanized assignment of items.

The classification of the items is recorded in a table. The classification of the items is noted consecutively, i.e., the table entry “RS, R, S” means that questionnaire item 1 was classified as RS-item, item 2 as R-item and item 3 as S-item. In this way, the reader can take the measuring instrument at hand and check the classification of each item.

After each item is classified, the percentage of the items that were assigned one of the five labels (R, RS, RS/SR or SR/RS, SR, S) in the total number of items is calculated. If the items marked with a label account for more than two-thirds (> 67%) of the total number of questionnaire items, the measure is classified as an R/S scale or sub-scale and is included in the meta-analysis. However, if the percentage of items marked with a label is 67% or less, the scale or sub-scale is excluded. If a study uses only one measurement instrument that did not qualify as an R/S measure according to our classification, the entire study is excluded (exclusion criteria 5). The percentage of items classified is also tabulated and indicated by the capital letter T, which stands for “total”. The discarded scales and sub-scales that did not qualify as R/S measures are recorded in a separate table.

To determine whether the scale or sub-scale as a whole should be considered an R-measure, RS-measure, RS/SR-measure, SR/RS-measure, SR-measure, or S-measure, the percentages of S-items and R-items within the total questionnaire items are also calculated. In this calculation, those items that received one of the three middle labels (RS, RS/SR or SR/RS, SR) count as both R-items and S-items. For a scale or sub-scale to be classified as a (partial) measure of "religion", at least 50 % of the items must receive a label containing an R (i.e., R, RS, RS/SR, SR/RS, SR). Accordingly, for scales or sub-scales to be recognized as instruments for the (partial) measurement of "spirituality", at least 50 % of the items must have received a label containing an S (i.e., S, SR, SR/RS, RS/SR, RS). If both percentages, i.e., the relative proportion of R-items and the relative proportion of S-items, account for more than 50 % of the total scale, it is a mixed scale. If the percentages are equal, it is an RS/SR scale (if the R-labels were somewhat more dominant in the classification of the individual items, e.g., there were more RS-items than SR-items), or an SR/RS scale (if there was a certain preponderance of S in the classification of the individual items). If either percentage is higher than the other, but both are above 50 %, the scale or sub-scale as a whole becomes an RS-scale (if the percentage of R-items is higher) or an SR-scale (if the percentage of S-items is higher). If one percentage is above 50 % but the other is below 50 %, it is either an R-scale (R-items above 50 % and S-items below 50 %) or an S-scale (S-items above 50 % and R-items below 50 %).

Let's illustrate the classification process with an invented example. A scale has five items, which were classified as follows: R, RS, X, SR, (R). Four of the five questionnaire items have been given a label, but in item 3 no reference to R/S could be found (which is indicated by the symbol X). One label (in this case the last R-item) is in brackets, which indicates that the item can be interpreted religiously, but has no unequivocal religious reference. Thus, the percentage of items with label in relation to the total scale is 80 % (4 of 5 items). The exemplary scale is therefore an R/S measure that meets our criteria (over 67 %). The percentage of R-items is also 80 % (4 of the 5 items contain a label with R), and the percentage of S-items is 40 % (2 of the 5 items contain a label with S). Since the R-items of the scale predominate and less than half of the items are related to spirituality, the sample

scale is classified as an R-measure. Tabulation of this result is as follows: **R**, T: 80%, R: 80%, S: 40%. The bold label (R in this case) indicates how the scale was classified overall. The letter T acts as an abbreviation for the percentage of items with label in the total scale, the letter R represents the percentage of R items in the total scale, and the letter S represents the percentage of S items.

References

Barrett, J. L. (1999). Theological correctness: Cognitive constraint and the study of religion. *Method & Theory in the Study of Religion, 11*(4), 325-339. doi: https://doi.org/10.1163/157006899X00078

DeBruyn, J. C. (2001). *Binge drinking and salutogenesis: Sense of coherence, stress, religiousness and spirituality* [Doctoral dissertation, Western Michigan University Kalamazoo]. ProQuest Dissertations and Theses Global.

Lindeman, M., Pyysiäinen, I., & Saariluoma, P. (2002). Representing God. *Papers on Social Representations, 11*, 1.1-1.13.

Pyysiäinen, I., & Ketola, K. (1999). Rethinking 'God': The concept of 'God'as a category in comparative religion. *Scripta Instituti Donneriani Aboensis, 17*(1), 207-214. doi: https://doi.org/10.30674/scripta.67254

Underwood, L. G., & Teresi, J. A. (2002). The daily spiritual experience scale: Development, theoretical description, reliability, exploratory factor analysis, and preliminary construct validity using health-related data. *Annals of Behavioral Medicine, 24*(1), 22-33. doi: https://doi.org/10.1207/s15324796abm2401_04
