# Supplementary material for: Sense of coherence and religion/spirituality: A systematic review and meta-analysis based on a methodical classification of instruments measuring religion/spirituality
Source: PLoS One. 2023 Aug 3;18(8):e0289203. doi: 10.1371/journal.pone.0289203 (PMC10399782; doi:10.1371/journal.pone.0289203)
Supplement: S1 Code — The code used for the meta-analytical procedures for the RStudio software, which can be copied and used to replicate the calculations we perform. (PDF) [file pone.0289203.s018.pdf]

S11 Code. R Code for Meta-Analytical Procedures.

#Opening the R packages

install.packages(c("robumeta", "metafor", "dplyr"))

library("robumeta")

library("metafor")

library("dplyr")

library(xlsx)

#Meta-analysis of the subgroup of negative R/S measures

name of subgroup <- escalc(measure="ZCOR", ri=r, ni=n, data= name of subgroup, slab=paste(authors, year, sep=", "))

res <- rma(yi, vi, data= name of subgroup)

res

predict(res, digits=3, transf=transf.ztor)

confint(res)

b_res <- rma(yi, vi, data= name of subgroup, slab=ID)

baujat(b_res)

forest(res, xlim=c(-1.6,1.6), atransf=transf.ztor,

at=transf.rtoz(c(-.4,-.2,0,.2,.4,.6)), digits=c(2,1), cex=.8)

text(-1.6, 18, "Author(s), Year", pos=4, cex=.8)

text(1.6, 18, "Correlation [95% CI]", pos=2, cex=.8)

funnel(res, xlab = "Correlation coefficient")

regtest(res)

ranktest(res)
